# Burdens of type 2 diabetes and cardiovascular disease attributable to sugar-sweetened beverages in 184 countries

**DOI:** 10.1038/s41591-024-03345-4

**Published:** 2025-01-06

**Authors:** Laura Lara-Castor, Meghan O’Hearn, Frederick Cudhea, Victoria Miller, Peilin Shi, Jianyi Zhang, Julia R. Sharib, Sean B. Cash, Simon Barquera, Renata Micha, Dariush Mozaffarian, Global Dietary Database, Antonia Trichopoulou, Murat Bas, Jemal Haidar Ali, Tatyana El-Kour, Anand Krishnan, Puneet Misra, Nahla Hwalla, Chandrashekar Janakiram, Nur Indrawaty Lipoeto, Abdulrahman Musaiger, Farhad Pourfarzi, Iftikhar Alam, Celine Termote, Anjum Memon, Marieke Vossenaar, Paramita Mazumdar, Ingrid Rached, Alicia Rovirosa, María Elisa Zapata, Roya Kelishadi, Tamene Taye Asayehu, Francis Oduor, Julia Boedecker, Lilian Aluso, Emanuele Marconi, Laura D’Addezio, Raffaela Piccinelli, Stefania Sette, Johana Ortiz-Ulloa, J. V. Meenakshi, Giuseppe Grosso, Anna Waskiewicz, Umber S. Khan, Kenneth Brown, Lene Frost Andersen, Anastasia Thanopoulou, Reza Malekzadeh, Neville Calleja, Anca Ioana Nicolau, Cornelia Tudorie, Marga Ocke, Zohreh Etemad, Mohannad Al Nsour, Lydiah M. Waswa, Maryam Hashemian, Eha Nurk, Joanne Arsenault, Patricio Lopez-Jaramillo, Abla Mehio Sibai, Albertino Damasceno, Pulani Lanerolle, Carukshi Arambepola, Carla Lopes, Milton Severo, Nuno Lunet, Duarte Torres, Heli Tapanainen, Jaana Lindstrom, Suvi Virtanen, Cristina Palacios, Noel Barengo, Eva Roos, Irmgard Jordan, Charmaine Duante, Corazon Cerdena, Imelda Angeles-Agdeppa, Josie Desnacido, Mario Capanzana, Anoop Misra, Ilse Khouw, Swee Ai Ng, Edna Gamboa Delgado, Mauricio T. Caballero, Johanna Otero, Hae-Jeung Lee, Eda Koksal, Idris Guessous, Carl Lachat, Stefaan De Henauw, Ali Reza Rahbar, Alison Tedstone, Annie Ling, Beth Hopping, Catherine Leclercq, Christian Haerpfer, Christine Hotz, Christos Pitsavos, Coline van Oosterhout, Debbie Bradshaw, Dimitrios Trichopoulos, Dorothy Gauci, Dulitha Fernando, Elzbieta Sygnowska, Erkki Vartiainen, Farshad Farzadfar, Gabor Zajkas, Gillian Swan, Guansheng Ma, Hajah Masni Ibrahim, Harri Sinkko, Isabelle Sioen, Jean-Michel Gaspoz, Jillian Odenkirk, Kanitta Bundhamcharoen, Keiu Nelis, Khairul Zarina, Lajos Biro, Lars Johansson, Leanne Riley, Mabel Yap, Manami Inoue, Maria Szabo, Marja-Leena Ovaskainen, Meei-Shyuan Lee, Mei Fen Chan, Melanie Cowan, Mirnalini Kandiah, Ola Kally, Olof Jonsdottir, Pam Palmer, Philippos Orfanos, Renzo Asciak, Robert Templeton, Rokiah Don, Roseyati Yaakub, Rusidah Selamat, Safiah Yusof, Sameer Al-Zenki, Shu-Yi Hung, Sigrid Beer-Borst, Suh Wu, Widjaja Lukito, Wilbur Hadden, Xia Cao, Yi Ma, Yuen Lai, Zaiton Hjdaud, Jennifer Ali, Ron Gravel, Tina Tao, Jacob Lennert Veerman, Mustafa Arici, Demosthenes Panagiotakos, Yanping Li, Gülden Pekcan, Karim Anzid, Anuradha Khadilkar, Veena Ekbote, Irina Kovalskys, Arlappa Nimmathota, Avula Laxmaiah, Balakrishna Nagalla, Brahmam Ginnela, Hemalatha Rajkumar, Indrapal Meshram, Kalpagam Polasa, Licia Iacoviello, Marialaura Bonaccio, Simona Costanzo, Yves Martin-Prevel, Nattinee Jitnarin, Wen-Harn Pan, Yao-Te Hsieh, Sonia Olivares, Gabriela Tejeda, Aida Hadziomeragic, Le Tran Ngoan, Amanda de Moura Souza, Daniel Illescas-Zarate, Inge Huybrechts, Alan de Brauw, Mourad Moursi, Augustin Nawidimbasba Zeba, Maryam Maghroun, Nizal Sarrafzadegan, Noushin Mohammadifard, Lital Keinan-Boker, Rebecca Goldsmith, Tal Shimony, Gudrun B. Keding, Shivanand C. Mastiholi, Moses Mwangi, Yeri Kombe, Zipporah Bukania, Eman Alissa, Nasser Al-Daghri, Shaun Sabico, Rajesh Jeewon, Martin Gulliford, Tshilenge S. Diba, Kyungwon Oh, Sihyun Park, Sungha Yun, Yoonsu Cho, Suad Al-Hooti, Chanthaly Luangphaxay, Daovieng Douangvichit, Latsamy Siengsounthone, Pedro Marques-Vidal, Peter Vollenweider, Constance Rybak, Amy Luke, Nipa Rojroongwasinkul, Noppawan Piaseu, Kalyana Sundram, Jeremy Koster, Donka Baykova, Parvin Abedi, Sandjaja Sandjaja, Fariza Fadzil, Noriklil Bukhary Ismail Bukhary, Pascal Bovet, Yu Chen, Norie Sawada, Shoichiro Tsugane, Lalka Rangelova, Stefka Petrova, Vesselka Duleva, Ward Siamusantu, Lucjan Szponar, Hsing-Yi Chang, Makiko Sekiyama, Khanh Le Nguyen Bao, Sesikeran Boindala, Jalila El Ati, Ivonne Ramirez Silva, Juan Rivera Dommarco, Luz Maria Sanchez-Romero, Simon Barquera, Sonia Rodríguez-Ramírez, Nayu Ikeda, Sahar Zaghloul, Anahita Houshiar-rad, Fatemeh Mohammadi-Nasrabadi, Morteza Abdollahi, Khun-Aik Chuah, Zaleha Abdullah Mahdy, Alison Eldridge, Eric L. Ding, Herculina Kruger, Sigrun Henjum, Milton Fabian Suarez-Ortegon, Nawal Al-Hamad, Veronika Janská, Reema Tayyem, Bemnet Tedla, Parvin Mirmiran, Almut Richter, Gert Mensink, Lothar Wieler, Daniel Hoffman, Benoit Salanave, Shashi Chiplonkar, Anne Fernandez, Androniki Naska, Karin De Ridder, Cho-il Kim, Rebecca Kuriyan, Sumathi Swaminathan, Didier Garriguet, Anna Karin Lindroos, Eva Warensjo Lemming, Jessica Petrelius Sipinen, Lotta Moraeus, Saeed Dastgiri, Sirje Vaask, Tilakavati Karupaiah, Fatemeh Vida Zohoori, Alireza Esteghamati, Sina Noshad, Suhad Abumweis, Elizabeth Mwaniki, Simon G. Anderson, Justin Chileshe, Sydney Mwanza, Lydia Lera Marques, Samuel Duran Aguero, Mariana Oleas, Luz Posada, Angelica Ochoa, Alan Martin Preston, Khadijah Shamsuddin, Zalilah Mohd Shariff, Hamid Jan Bin Jan Mohamed, Wan Manan, Bee Koon Poh, Pamela Abbott, Mohammadreza Pakseresht, Sangita Sharma, Tor Strand, Ute Alexy, Ute Nöthlings, Indu Waidyatilaka, Ranil Jayawardena, Julie M. Long, K. Michael Hambidge, Nancy F. Krebs, Aminul Haque, Liisa Korkalo, Maijaliisa Erkkola, Riitta Freese, Laila Eleraky, Wolfgang Stuetz, Laufey Steingrimsdottir, Inga Thorsdottir, Ingibjorg Gunnarsdottir, Lluis Serra-Majem, Foong Ming Moy, Corina Aurelia Zugravu, Elizabeth Yakes Jimenez, Linda Adair, Shu Wen Ng, Sheila Skeaff, Regina Fisberg, Carol Henry, Getahun Ersino, Gordon Zello, Alexa Meyer, Ibrahim Elmadfa, Claudette Mitchell, David Balfour, Johanna M. Geleijnse, Mark Manary, Laetitia Nikiema, Masoud Mirzaei, Rubina Hakeem

**Affiliations:** 1 Food Is Medicine Institute, Friedman School of Nutrition Science and Policy, Tufts University, Boston, MA USA; 2 Institute of Health Metrics and Evaluation, University of Washington, Seattle, WA USA; 3 Food Systems for the Future Institute, Chicago, IL USA; 4 Department of Medicine, McMaster University, Hamilton, Ontario Canada; 5 Population Health Research Institute, Hamilton, Ontario Canada; 6 Center for Surgery and Public Health, Brigham and Women’s Hospital, Boston, MA USA; 7 Friedman School of Nutrition Science and Policy, Tufts University, Boston, MA USA; 8 Research Center on Nutrition and Health, National Institute of Public Health, Cuernavaca, Mexico; 9 University of Thessaly, Volos, Greece; 10 Tufts University School of Medicine, Boston, MA USA; 11 Department of Medicine, Tufts Medical Center, Boston, MA USA; 12 Academy of Athens, Athens, Greece; 13 Acibadem University, Istanbul, Turkey; 14 Addis Ababa University, Addis Ababa, Ethiopia; 15 Aga Khan Foundation, Geneva, Switzerland; 16 All India Institute of Medical Sciences, New Delhi, India; 17 American University of Beirut, Beirut, Lebanon; 18 Amrita School of Dentistry, Eranakulum, India; 19 Andalas University, Padang, Indonesia; 20 Arab Center for Nutrition, Manama, Bahrain; 21 Ardabil University of Medical Sciences, Ardabil, Iran; 22 Bacha Khan University, Charsadda, Pakistan; 23 Biodiversity International, Nairobi, Kenya; 24 Brighton and Sussex Medical School, Brighton, UK; 25 Center for Studies of Sensory Impairment Aging and Metabolism (CeSSIAM), Guatemala City, Guatemala; 26 Centre For Media Studies, New Delhi, India; 27 Centro de Atencion Nutricional Antimano (CANIA), Miami, FL USA; 28 Centro de Estudios sobre Nutrición Infantil (CESNI), Buenos Aires, Argentina; 29 Child Growth and Development Research Center, Isfahan University of Medical Sciences, Isfahan, Iran; 30 College of Applied Sciences, Department of Food Science and Applied Nutrition, Addis Ababa Science and Technology University, Addis Ababa, Ethiopia; 31 Consultative Group on International Agricultural Research (CGIAR), Montpellier, France; 32 Council for Agricultural Research and Economics, Research Centre for Food and Nutrition, Rome, Italy; 33 Cuenca University, Cuenca, Ecuador; 34 Delhi School of Economics, University of Delhi, Delhi, India; 35 Department of Biomedical and Biotechnological Sciences, University of Catnia, Catania, Italy; 36 Department of CVD Epidemiology, Prevention and Health Promotion, Institute of Cardiology, Warsaw, Poland; 37 Department of Community Health Sciences, Aga Khan University, Karachi, Pakistan; 38 Department of Nutrition and Institute for Global Nutrition, University of California Davis, Davis, CA USA; 39 Department of Nutrition, University of Oslo, Oslo, Norway; 40 Diabetes Center, 2nd Department of Internal Medicine, Athens University, Athens, Greece; 41 Digestive Disease Research Institute, Tehran University of Medical Sciences, Tehran, Iran; 42 Directorate for Health Information and Research, Pietà, Malta; 43 Dunarea de Jos University of Galati, Galati, Romania; 44 Dutch National Institute for Public Health and the Environment (RIVM), Bilthoven, Netherlands; 45 Eastern Mediterranean Public Health Network (EMPHNET), Amman, Jordan; 46 Egerton University, Njoro, Kenya; 47 Epidemiology and Community Health Branch, National Heart, Lung, and Blood Institute, National Institutes of Health, Bethesda, MD USA; 48 Estonian National Institute for Health Development, Tallinn, Estonia; 49 FHI360, Washington DC, USA; 50 FOSCAL and UDES, Bucaramanga, Colombia; 51 Faculty of Health Sciences, American University of Beirut, Beirut, Lebanon; 52 Faculty of Medicine, Eduardo Mondlane University, Maputo, Mozambique; 53 Faculty of Medicine, University of Colombo, Colombo, Sri Lanka; 54 Faculty of Medicine/ Institute of Public Health, University of Porto, Porto, Portugal; 55 Faculty of Nutrition and Food Sciences, University of Porto, Porto, Portugal; 56 Finnish Institute for Health and Welfare, Helsinki, Finland; 57 Florida International University, Miami, FL USA; 58 Folkhälsan Research Center, Helsinki, Finland; 59 Food Envrionment Consumer Behaviour Lever of the Alliance Bioversity International and CIAT, Nairobi, Kenya; 60 Food and Nutrition Research Institute (DOST-FNRI), Manila, Philippines; 61 Food and Nutrition Research Institute (DOST-FNRI), Taguig City, Philippines; 62 Fortis CDOC Center for Excellence for Diabetes, New Delhi, India; 63 National Diabetes, Obesity and Cholesterol Foundation (N-DOC), Diabetes Foundation (India), Fortis CDOC Hospital for Diabetes and Allied Sciences, New Delhi, India; 64 FrieslandCampina, Amersfoort, The Netherlands; 65 Fundacion Cardiovascular de Colombia, Bucaramanga, Colombia; 66 Fundacion INFANT and Consejo Nacional De Investigaciones Cientificas y Tecnicas (CONICET), Buenos Aires, Argentina; 67 Fundacion Oftalmologica de Santander (FOSCAL), Floridablanca, Colombia; 68 Gachon University, Seongnam-si, South Korea; 69 Gazi University, Ankara, Turkey; 70 Geneva University Hospitals, Geneva, Switzerland; 71 Ghent University, Ghent, Belgium; 72 Global Dietary Database Consortium, Boston, MA USA; 73 Statistics Canada, Government of Canada, Ottawa, Ontario Canada; 74 Griffith University, Gold Coast, Queensland Australia; 75 Faculty of Medicine, Hacettepe University, Ankara, Turkey; 76 Harokopio University, Athens, Greece; 77 Harvard School of Public Health, Cambridge, MA USA; 78 Department of Nutrition and Dietetics, Hasan Kalyoncu University, Gaziantep, Turkey; 79 Higher Institute of Nursing Professions and Health Techniques, Marrakesh, Morocco; 80 Hirabai Cowasji Jehangir Medical Research Institute, Pune, India; 81 ICCAS (Instituto para la Cooperacion Científica en Ambiente y Salud), Buenos Aires, Argentina; 82 ICMR-National Institute of Nutrition, Hyderabad, India; 83 IRCCS Neuromed, Pozzilli, Italy; 84 LUM University “Giuseppe Degennaro”, Casamassima, Italy; 85 Institut de Recherche pour le Developpement, Montpellier, France; 86 Institute for International Investigation, NDRI-USA, New York, NY USA; 87 Institute of Biomedical Sciences, Academia Sinica, Taipei, Taiwan; 88 Institute of Nutrition and Food Technology (INTA), University of Chile, Santiago, Chile; 89 Institute of Nutrition in Central America and Panama (INCAP), Guatemala City, Guatemala; 90 Institute of Public Health of Federation of Bosnia and Herzegovina, Sarajevo, Bosnia and Herzegovina; 91 Institute of Research and Development, Duy Tan University, Da Nang City, Vietnam; 92 School of Preventive Medicine and Public Health, Hanoi City, Vietnam; 93 Institute of Studies in Public Health, Federal University of Rio de Janeiro (UFRJ), Rio de Janeiro, Brazil; 94 Instituto Nacional de Ciencias Médicas y Nutrición Salvador Zubirán, Mexico City, Mexico; 95 Tecnologico de Monterrey, , Escuela de Medicina y Ciencias de la Salud, Mexico City, Mexico; 96 International Agency for Research on Cancer, Lyon, France; 97 International Food Policy Research Institute (IFPRI), Washington DC, USA; 98 Intitut de Recherche en Sciences de la Sante, Bobo Dioulasso, Burkina Faso; 99 Isfahan Cardiovascular Research Center, Cardiovascular Research Institute, Isfahan University of Medical Sciences, Isfahan, Iran; 100 Israel Center for Disease Control, Tel-Hashomer, Israel; 101 Institute of Nutritional Sciences, Justus Liebig University of Giessen, Giessen, Germany; 102 KLE Academy of Higher Education and Research (Deemed-to-be-University) Jawaharlal Nehru Medical College, Belagavi, India; 103 Kenya Medical Research Institute, Nairobi, Kenya; 104 King Abdulaziz University, Jeddah, Saudi Arabia; 105 King Saud University, Riyadh, Saudi Arabia; 106 University of Mauritius, Reduit, Mauritius; 107 King’s College London, London, UK; 108 Kinshasa School of Public Health, Kinshasa, Democratic Republic of Congo; 109 Korea Disease Control and Prevention Agency (KDCA), Cheongju-si, South Korea; 110 Korea University, Seoul, South Korea; 111 Kuwait Institute for Scientific Research, Safat, Kuwait; 112 Lao Tropical and Public Health Institute, Vientiane, Lao People’s Democratic Republic; 113 Lausanne University Hospital (CHUV) and University of Lausanne, Lausanne, Switzerland; 114 Leibniz Centre for Agricultural Landscape Research, Muncheberg, Germany; 115 Loyola University Chicago, Chicago, IL USA; 116 Mahidol University, Pathom, Thailand; 117 Malaysian Palm Oil Council (MPOC), Kelana Jaya, Malaysia; 118 Max Planck Institute for Evolutionary Anthropology, Leipzig, Germany; 119 Medical Center Markovs, Sofia, Bulgaria; 120 Menopause Andropause Research Center, Ahvaz Jundishapur University of Medical Sciences, Ahvaz, Iran; 121 Ministry of Health, Jakarta, Indonesia; 122 Ministry of Health, Kuala Lumpur, Malaysia; 123 Ministry of Health, Sungai Besar, Malaysia; 124 Ministry of Health, Victoria, Seychelles; 125 University Center for Primary Care and Public Health (Unisanté), Lausanne, Switzerland; 126 NYU School of Medicine, New York, NY USA; 127 National Cancer Center Institute for Cancer Control, Tokyo, Japan; 128 National Centre of Public Health and Analyses (NCPHA), Sofia, Bulgaria; 129 National Food and Nutrition Commission, Lusaka, Zambia; 130 National Food and Nutrition Institute, Warsaw, Poland; 131 National Health Research Institutes, Zhunan Township, Taiwan, Republic of China; 132 Health and Environmental Risk Division, National Institute for Environmental Studies, Tsukuba, Japan; 133 National Institute of Nutrition, Hanoi, Vietnam; 134 National Institute of Nutrition, Hyderabad, India; 135 National Institute of Nutrition and Food Technology & SURVEN RL, Tunis, Tunisia; 136 National Institute of Public Health (INSP), Cuernavaca, Mexico; 137 National Institutes of Biomedical Innovation Health and Nutrition, Osaka, Japan; 138 National Nutrition Institute, Cairo, Egypt; 139 National Nutrition and Food Technology Research Institute (NNFTRI): SBMU, Tehran, Iran; 140 National University of Malaysia (UKM), Kuala Lumpur, Malaysia; 141 Nestlé Research, Lausanne, Switzerland; 142 New England Complex Systems Institute, Cambridge, MA USA; 143 North-West University, Potchefstroom South Africa, Potchefstroom, South Africa; 144 Oslo Metropolitan University (OsloMet), Oslo, Norway; 145 Pontificia Universidad Javeriana Seccional Cali, Cali, Colombia; 146 Public Authority For Food and Nutrition, Sabah Al Salem, Kuwait; 147 Public Health Authority of the Slovak Republic, Bratislava, Slovak Republic; 148 Qatar University and University of Jordan, Doha, Qatar; 149 Queensland University of Technology, Brisbane, Queensland Australia; 150 Research Institute for Endocrine Sciences, Shahid Beheshti University of Medical Sciences, Tehran, Iran; 151 Robert Koch Institute, Berlin, Germany; 152 Rutgers University, New Brunswick, NJ USA; 153 Santé publique France, the French Public Health Agency, Saint Maurice, France; 154 Savitribai Phule Pune University (SPPU), Pune, India; 155 School of Medicine, Deakin University, Geelong, Victoria Australia; 156 School of Medicine, National and Kapodistrian University of Athens, Athens, Greece; 157 Sciensano (Belgian Public Health Institute), Brussels, Belgium; 158 Seoul National University, Seoul, South Korea; 159 St John’s Research Institute, Bangalore, India; 160 Statistics Canada, Ottawa, Ontario Canada; 161 Swedish Food Agency, Uppsala, Sweden; 162 Tabriz University of Medical Sciences, Tabriz, Iran; 163 Tallinn University, Tallinn, Estonia; 164 Taylor’s University, Subang Jaya, Malaysia; 165 Teesside University, Middlesbrough, UK; 166 Tehran University of Medical Sciences, Tehran, Iran; 167 The Hashemite University, Az Zarqa, Jordan; 168 The Technical University of Kenya, Nairobi, Kenya; 169 The University of the West Indies, Kingston, Jamaica; 170 Tropical Diseases Research Centre, Ndola, Zambia; 171 Unidad de Nutricion Publica, Macul, Chile; 172 Universidad San Sebastian, Providencia, Chile; 173 Universidad Tecnica del Norte, Ibarra, Ecuador; 174 Universidad de Antioquia, Medellin, Colombia; 175 Universidad de Cuenca, Cuenca, Ecuador; 176 Medical Sciences Department of Biochemistry, Universidad de Puerto Rico, San Juan, Puerto Rico; 177 Universiti Kebangsaan Malaysia Medical Centre, Kuala Lumpur, Malaysia; 178 Universiti Putra Malaysia, Serdang, Malaysia; 179 Universiti Sains Malaysia, Kubang Kerian, Malaysia; 180 University Kebangsaan Malaysia, Bangi, Malaysia; 181 University of Aberdeen, Aberdeen, UK; 182 University of Alberta, Edmonton, Alberta Canada; 183 University of Bergen, Bergen, Norway; 184 Department of Nutrition and Food Sciences, University of Bonn, Bonn, Germany; 185 University of Colombo, Colombo, Sri Lanka; 186 University of Colorado School of Medicine, Aurora, CO USA; 187 University of Dhaka, Dhaka, Bangladesh; 188 Department of Food and Nutrition, University of Helsinki, Helsinki, Finland; 189 University of Hohenheim, Stuttgart, Germany; 190 University of Iceland, Reykjavik, Iceland; 191 University of Las Palmas de Gran Canaria (ULPGC), Las Palmas, Spain; 192 University of Malaya, Kuala Lumpur, Malaysia; 193 University of Medicine and Pharmacy Carol Davila, Bucharest, Romania; 194 University of New Mexico Health Sciences Center, Albuquerque, NM USA; 195 University of North Carolina at Chapel Hill, Chapel Hill, NC USA; 196 University of Otago, Dunedin, New Zealand; 197 University of Sao Paulo, Sao Paulo, Brazil; 198 University of Saskatchewan, Saskatoon, Saskatchewan Canada; 199 University of Vienna, Vienna, Austria; 200 University of the Southern Caribbean, Port of Spain, Trinidad and Tobago; 201 Wageningen University, Wageningen, Netherlands; 202 Washington University in St. Louis, St. Louis, MO USA; 203 World Health Organization (WHO), Geneva, Switzerland; 204 Yazd Cardiovascular Research Centre, Shahid Sadoughi University of Medical Sciences, Yazd, Iran; 205 Ziauddin University Karachi, Karachi City, Pakistan

**Keywords:** Risk factors, Epidemiology

## Abstract

The consumption of sugar-sweetened beverages (SSBs) is associated with type 2 diabetes (T2D) and cardiovascular diseases (CVD). However, an updated and comprehensive assessment of the global burden attributable to SSBs remains scarce. Here we estimated SSB-attributable T2D and CVD burdens across 184 countries in 1990 and 2020 globally, regionally and nationally, incorporating data from the Global Dietary Database, jointly stratified by age, sex, educational attainment and urbanicity. In 2020, 2.2 million (95% uncertainty interval 2.0–2.3) new T2D cases and 1.2 million (95% uncertainty interval 1.1–1.3) new CVD cases were attributable to SSBs worldwide, representing 9.8% and 3.1%, respectively, of all incident cases. Globally, proportional SSB-attributable burdens were higher among men versus women, younger versus older adults, higher- versus lower-educated adults, and adults in urban versus rural areas. By world region, the highest SSB-attributable percentage burdens were in Latin America and the Caribbean (T2D: 24.4%; CVD: 11.3%) and sub-Saharan Africa (T2D: 21.5%; CVD: 10.5%). From 1990 to 2020, the largest proportional increases in SSB-attributable incident T2D and CVD cases were in sub-Saharan Africa (+8.8% and +4.4%, respectively). Our study highlights the countries and subpopulations most affected by cardiometabolic disease associated with SSB consumption, assisting in shaping effective policies and interventions to reduce these burdens globally.

## Abstract (web summary)

Estimates from the Global Dietary Database indicated that 2.2 million new type 2 diabetes and 1.2 million new cardiovascular disease cases were attributable to sugar-sweetened beverages worldwide in 2020, with the highest burdens in sub-Saharan Africa, Latin America and the Caribbean.

## Main

Sugar-sweetened beverages (SSBs) contribute to excess weight gain and cardiometabolic diseases such as type 2 diabetes (T2D) and cardiovascular disease (CVD), both directly and mediated by weight gain^1,2^. Despite progress in elucidating the role of SSBs in health, an updated and comprehensive assessment of the global disease burden attributed to SSBs remains scarce. Our previous study estimated that, in 2010, intake of SSBs was responsible for 184,000 global deaths^3^. More recent analyses looking at 87 different risk factors in 2019, including SSB intake^4^, relied primarily on national per capita estimates of added sugar availability or sales data^5^, rather than individual-level dietary data^6,7^, limiting the validity and precision of estimates across population subgroups.

Due to their liquid form, SSBs are rapidly consumed and digested, resulting in lower satiety, higher caloric intake and weight gain^8^. High doses of rapidly digested glucose also activate insulin and other regulatory pathways, which can result in visceral fat production, hepatic and skeletal muscle insulin resistance and weight gain. High doses of rapidly digested fructose directly activate hepatic fat synthesis, leading to ectopic fat deposition and metabolic dysfunction in liver and muscle^9^. SSBs may also replace other healthier foods in the diet, contributing to harms through their absence. Excess adiposity and metabolic dysfunction activate inflammatory cytokines and increase risk of hypertension, dyslipidemia and diabetes^10^. All these risk factors accelerate atherosclerosis and plaque instability, contributing to ischemic cardiovascular events^11^. Hence, both direct and adiposity-mediated effects of SSBs are relevant to assessing their health effects.

Both SSB intake and cardiometabolic risk also can vary substantially by key demographic factors within nations. For example, we recently reported that SSB intakes were higher among more versus less educated adults in sub-Saharan Africa, South Asia and Latin America and the Caribbean, while the inverse pattern was observed in Middle East and North Africa^12^. By area of residence, intakes were higher in urban versus rural areas in sub-Saharan Africa and South Asia, whereas the inverse was true in the Middle East and North Africa. Yet, assessments of the global disease burden attributable to SSBs by key demographics such as educational attainment and urban versus rural residence have yet to be reported at a global scale.

This study aims to estimate the burdens of cardiometabolic diseases attributable to SSBs and the changes over time, in nations worldwide as well as subnationally, by key sociodemographic factors, as highlighted by the substantial public health challenge of SSB intake in most world regions^12^. The findings would inform national, subnational and multinational surveillance and policy actions to address SSBs and their disease burdens, including inequities across nations and population subgroups (Table 1).

**Table 1:** Policy summary

Background	An updated and comprehensive assessment of the global, regional and national disease burdens attributable to SSBs remains scarce, particularly by key demographics such as education and urban or rural residence.
Main findings and limitations	In 2020, 2.2 million (95% UI 2.0–2.3) new T2D cases and 1.2 million (1.1–1.3) new CVD cases were attributable to SSBs worldwide, representing 9.8% and 3.1%, respectively, of all incident cases. By world region, the highest absolute cases per million adults (20+ years) were in Latin America and the Caribbean (T2D: 1,263; CVD: 522) and Middle East and North Africa (T2D: 1,001; CVD: 815), while the highest proportional SSB-attributable cases were in Latin America and the Caribbean (T2D: 24.4%; CVD: 11.3%) and sub-Saharan Africa (T2D: 21.5%; CVD: 10.5%). In 2020, the greatest absolute number of new T2D cases attributable to SSBs per million adults among the 30 most populous countries were in Mexico (2,007 per million adults; 30% of all T2D incidence cases), Colombia (1,971; 48.1%) and South Africa (1,258; 27.6%). For CVD, the greatest numbers were in Colombia (1,084; 23.0%), South Africa (828; 14.6%) and Mexico (721; 13.5%). Jointly considering education, urbanicity and world region, the highest proportions of incident T2D attributable to SSBs were among high-educated (31.9%) and mid-educated (34.2%) adults in urban sub-Saharan Africa, followed by high- and medium-educated adults in both urban and rural Latin America and the Caribbean (~26% each). Findings were similar for CVD, with the largest SSB-attributable proportions among higher-educated (19.5%) and mid-educated (17.6%) adults from urban areas in sub-Saharan Africa, but also among higher-educated and mid-educated adults from rural areas in sub-Saharan Africa and both urban and rural areas in Latin America and the Caribbean (~12–13% each). From 1990 to 2020, sub-Saharan Africa had the largest proportional increases in incident T2D and CVD (12.7–21.5% and 6.1–10.5%, respectively), while Latin America and the Caribbean experienced slight decreases. The findings represent estimates based on available data and reasoned assumptions and do not prove cause and effect.
Policy implications	Our study offers a comprehensive analysis of the global burden of SSB-attributable T2D and CVD burdens, incorporating sociodemographic disparities and regional nuances. While some policies to curb SSB intakes are currently in place in some countries, our study suggests that more work is needed. In Latin America and the Caribbean, for instance, several nations have implemented policies to curb SSB intakes, yet this region had the largest SSB-attributable cardiometabolic burdens in 2020. Mid- and high-educated adults in rural and urban Latin America and the Caribbean and rural sub-Saharan Africa should be given particular attention. The increasing burdens in sub-Saharan Africa shed light on the necessity of acting quickly in this region. By highlighting the countries and subpopulations most affected, our research can assist in shaping effective policies and interventions to efficiently reduce the burden of cardiometabolic diseases attributed to SSB consumption globally.

## Results

### Distributions of SSB intakes

SSBs were defined as any beverage with added sugars and ≥50 kcal per 8 oz serving, including commercial or homemade beverages, soft drinks, energy drinks, fruit drinks, punch, lemonade and aguas frescas. This definition excluded 100% fruit and vegetable juices, noncaloric artificially sweetened drinks and sweetened milk. We derived SSB intakes from the Global Dietary Database (GDD)^7,12,13^, including 450 surveys with data on SSBs, totaling 2.9 million individuals from 118 countries representing 87.1% of the global population (Supplementary Tables 1 and 2) and Bayesian hierarchical modeling. Globally in 2020, consistent with findings reported in 2018^12^, adults consumed an average of 2.6 8 oz (248 g) servings per week (95% uncertainty interval (UI) 2.4–2.8). This ranged regionally from 0.7 (95% UI 0.5–1.1) in South Asia to 7.3 (95% UI 6.7–8.1) in Latin America and the Caribbean (Supplementary Table 3), and nationally among the 30 most populous countries from 17.4 in Colombia (95% UI 13.2–22.7), 9.6 in South Africa (95% UI 7.5–12.5), 8.5 in Mexico (95% UI 7.8–9.4) and 6.9 in Ethiopia (95% UI 5.5–8.7) to 0.2 in India, China and Bangladesh (Supplementary Table 4).

Globally, regionally and nationally, men had modestly higher energy-adjusted SSB intake than women. By age, SSB intakes were higher at younger compared with older ages in all world regions, though with varying absolute magnitudes of intakes and differences by region (Supplementary Table 3). The largest variations by age were observed in Latin America and the Caribbean, and the lowest in South Asia. By region and education, intakes were higher among more versus less educated adults in sub-Saharan Africa, South Asia and Latin America and the Caribbean, but lower among more versus less educated adults in the Middle East and North Africa, with smaller differences by education in other regions^12^.

### Global T2D and CVD burdens attributable to SSBs

SSB intakes and cardiometabolic disease rates were incorporated into a comparative risk assessment (CRA) model to assess risk. The CRA framework does not use ecologic correlations but is based on independent lines of evidence^14^, including age-adjusted etiologic effects of SSBs on T2D, ischemic heart disease and ischemic stroke, both directly and mediated by body mass index (BMI), from previous meta-analyses and pooled analyses of prospective cohorts, supported by evidence from randomized controlled trials^2,15–17^. For each model, we ran 1,000 Monte Carlo simulations and report the median and 2.5th and 97.5th values (95% UI).

**Extended Data Fig. 1:**
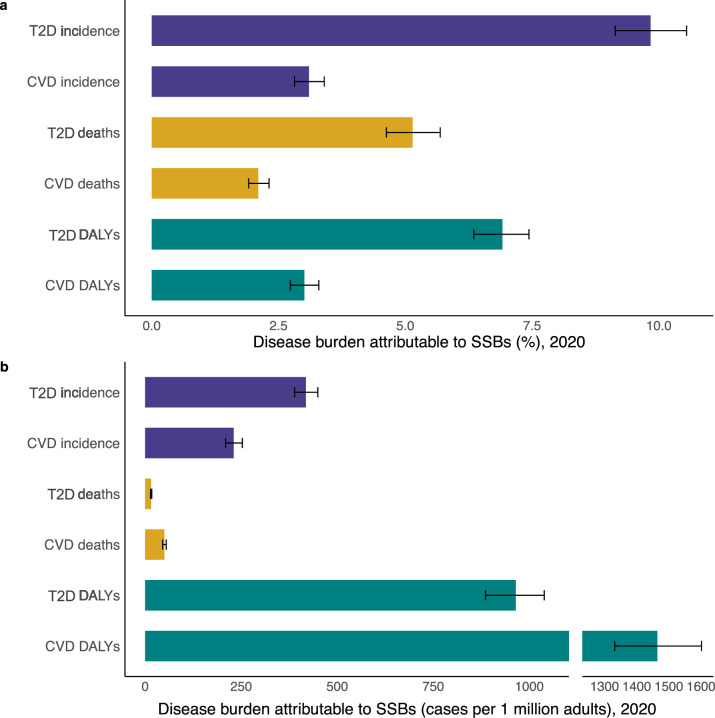
Global disease burden attributable to intake of sugar-sweetened beverages among adults (20+ years) in 2020. Bars represent the estimated proportional SSB-attributable burden in the top panel (**a**) and the estimated SSB-attributable absolute cases per 1 million adults in the bottom panel (**b**) of T2D and CVD incidence, deaths, and DALYs. Data are presented as central estimate (median) and the corresponding 95% UI, derived from the 2.5^th^ and 97.5^th^ percentiles of 1,000 multiway probabilistic Monte Carlo model simulations. The SSB-attributable absolute burden per 1 million adults was calculated by dividing the absolute number of SSB-attributable cases by the total adult population (20+ years) in that year and multiplying it by 1 million. Source data are provided in Source Data file 6. CVD, cardiovascular disease; SSBs, sugar sweetened beverages; T2D, type 2 diabetes; UIs, uncertainty intervals. Source data

In 2020, an estimated 2.2 million (95% UI 2.0–2.3) new T2D cases and 1.2 million (95% UI 1.1–1.3) new CVD cases were attributable to intake of SSBs globally, corresponding to 9.8% (95% UI 9.1–10.5) and 3.1% (95% UI 2.8–3.4) of total incident cases, respectively (Supplementary Data 1 and Extended Data Fig. 1). SSBs contributed to 12.5 million cardiometabolic disability-adjusted life years (DALYs), including 5.0 million (95% UI 4.6–5.4) from T2D (6.9% (6.4–7.4) of all T2D DALYs) and 7.6 million (95% UI 6.9–8.3) from CVD (3.0% (2.7–3.3) of all CVD DALYs). SSBs were estimated to cause 80,278 (72,297–88,824) deaths from T2D (5.1% (4.6–5.7) of all T2D deaths) and 257,962 (235,059–283,798) deaths from CVD (2.1% (1.9–2.3) of all CVD deaths).

**Fig. 1:**
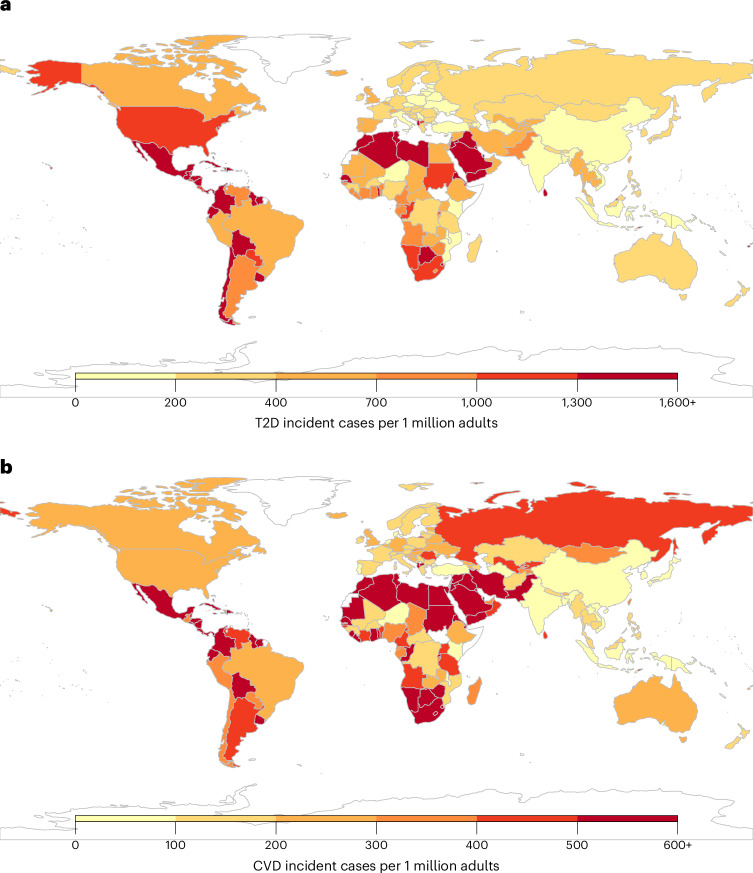
Incidence of T2D and CVD per 1 million adults attributable to SSB intake among adults (20+ years) in 184 countries in 2020. **a**,**b**, Absolute SSB-attributable T2D incidence (**a**) and absolute SSB-attributable CVD incidence (**b**). The SSB-attributable absolute burden per 1 million adults was calculated by dividing the country absolute number of SSB-attributable cases by the country adult population (20+ years) in that same year and multiplying by 1 million. Values were truncated at 1,600 for **a** and at 600 for **b** to better reflect the absolute case distribution globally for T2D and CVD. The analysis of the data was done using the rworldmap package (v1.3-6). Source data are provided in Source Data Fig. 1. Source data

Among the 30 most populous countries, the greatest absolute numbers of new T2D cases attributable to SSBs were in Mexico (2,007 per million adults (1,754–2,338)), Colombia (1,971 (1,612–2,354)) and South Africa (1,258 (1,005–1,575)) (Fig. 1 and Supplementary Data 1). For CVD, the greatest numbers were in Colombia (1,084 (832–1,381)), South Africa (828 (645–1,083)) and Mexico (721 (612–889)). As a proportion of all new cases, the highest SSB burdens of T2D were in Colombia (48.1% (39.3–57.3)), Mexico (30.0% (26.4–35.0)) and South Africa (27.6% (22.1–34.6)), and those of CVD were in Colombia (23.0% (18.0–29.2)), South Africa (14.6% (11.4–19.2)) and Mexico (13.5% (11.5–16.7)). Findings on cardiometabolic deaths and DALYs attributable to SSBs in 184 nations are presented in Supplementary Figs. 1 and 2 and Supplementary Data 1.

### Regional T2D and CVD burdens attributable to SSBs

**Fig. 2:**
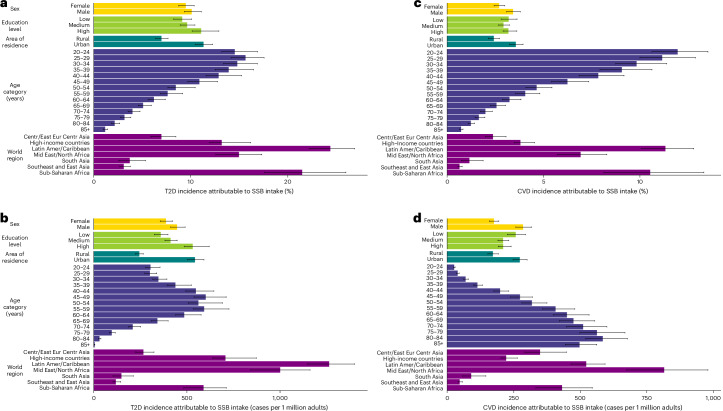
Incidence of T2D and CVD attributable to SSB intake by key sociodemographic factors at the global level and by world region in 2020. **a**–**d**, The bars represent the central estimate (median) of the proportional SSB-attributable T2D incidence (**a**), the absolute SSB-attributable T2D incidence per 1 million adults (**b**), the proportional SSB-attributable CVD incidence (**c**) and the absolute SSB-attributable CVD incidence per 1 million adults (**d**). The error bars represent the 95% UI derived from the 2.5th and 97.5th percentiles of 1,000 multiway probabilistic Monte Carlo model simulations. The SSB-attributable absolute burden per 1 million adults was calculated by dividing the stratum absolute number of SSB-attributable cases by the stratum adult population (20+ years) in that same year and multiplying by 1 million. In previous GDD reports, the region ‘Central and Eastern Europe and Central Asia’ was referred to as ‘Former Soviet Union’, and ‘Southeast and East Asia’ was referred to as ‘Asia’. See Supplementary Table 2 for a list of countries included in each world region. Source data are provided in Source Data Fig. 2. Centr/East Eur Centr Asia, Central or Eastern Europe and Central Asia; Latin Amer/Caribbean, Latin America and the Caribbean; Mid East/North Africa, Middle East and North Africa. Source data

By world region, Latin America and the Caribbean had the highest absolute and proportional T2D incidence due to SSBs (1,263 new cases per 1 million (1,146–1,400); 24.4% (22.3.0–26.9)), and Southeast and East Asia had the lowest (119 new cases per 1 million (103–145); 3.1% (2.7–3.8)) in 2020 (Fig. 2 and Supplementary Table 5). SSB-attributable CVD incidence ranged from 815 new cases per 1 million (674–980) in the Middle East and North Africa to 46.8 new cases per 1 million (41.0–57.1) in Southeast and East Asia (Fig. 2 and Supplementary Table 6). SSBs were estimated to have caused more than 1 in 10 new CVD cases in Latin America and the Caribbean (11.3% (10.1–12.8)) and sub-Saharan Africa (10.5% (8.1–13.3)), compared with less than 1 in 100 cases in South Asia (0.60% (0.6–0.8)). Cardiometabolic mortality and DALYs from SSBs in different world regions are presented in Supplementary Figs. 3–6 and Supplementary Tables 5 and 6.

### Global heterogeneity by age, sex, education and urbanicity

Globally, SSBs were estimated to cause more T2D cases in men (447 per 1 million adults (413–491); 10.1% (9.3–11.10) of total cases) versus women (388 per 1 million adults (358–422); 9.5% (8.8–10.4)), higher-educated (531 per 1 million adults (486–620); 11.1% (10.1–12.9)) versus lower-educated adults (360 per 1 million adults (326–398); 9.1% (8.2–10.1)) and urban (543 per 1 million adults (502–592); 11.3% (10.5–12.2)) versus rural adults (244 per 1 million adults (233–267); 7.0% (6.4–7.7)) in 2020 (Fig. 2 and Supplementary Table 5). By age, absolute burdens of SSB-attributable T2D cases were highest at ages 45–49 years (601 per 1 million adults (539–711); 10.9% (9.7–12.8)), while proportional risk was highest at ages 25–29 years (301 per 1 million adults (274–338); 15.6% (14.2–17.6)).

For CVD, absolute SSB-attributable incident cases per 1 million adults were higher among men (285 per 1 million adults (258–317)) than women (176 per 1 million adults (159–194)) in 2020, owing to both higher SSB intake and higher baseline CVD risk (Fig. 2 and Supplementary Table 6). SSB-attributable CVD incidence was also higher in urban adults (273 per 1 million adults (247–300)) than in rural adults (172 per 1 million adults (153–192)), for similar reasons. By contrast, global SSB-attributable CVD incidence was similar across education levels. Absolute incidence of SSB-attributable CVD increased with age, while proportion risk decreased with age. For example, SSBs were estimated to contribute to 585 new CVD cases (520–677) per 1 million adults among adults aged 80–84 years (1.2% (1.1–1.4) of total incident CVD in this age group) versus 26.2 new CVD cases (23.6–29.8) per 1 million among adults aged 20–24 years (12.0% (10.6–13.5) of total incident CVD in this age group). T2D and CVD mortality and DALYs attributable to SSBs followed similar patterns as for T2D and CVD incidence (Supplementary Figs. 3–6 and Supplementary Tables 5 and 6).

### Regional and national heterogeneity by age, education and urbanicity

**Extended Data Fig. 2:**
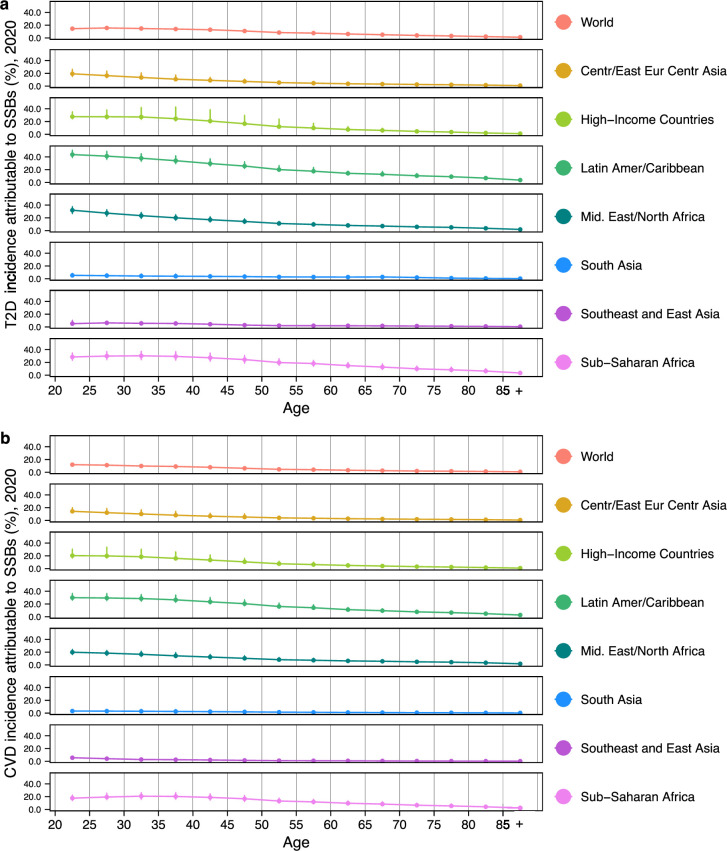
Proportional incidence of T2D and CVD attributable to SSBs intake among adults (20+ years) jointly stratified by world region and age in 2020. The filled circles represent the central estimate (median) of the proportional SSB-attributable diabetes incidence in the top panel (**a**) CVD incidence in the bottom panel (**b**). The error bars represent the 95% UI derived from the 2.5^th^ and 97.5^th^ percentiles of 1,000 multiway probabilistic Monte Carlo model simulations. The age groups are 20-24, 25-29, 30-34, 35-39, 40-44, 45-49, 50-54, 55-59, 60-64, 65-69, 70-74, 75-79, 80-84, 85+ years. In prior GDD reports, the region Central/ Eastern Europe and Central Asia was referred as Former Soviet Union, and Southeast and East Asia was referred as Asia. See Supplementary Table 1 for a list of countries included in each world region. Source data are provided in Source Data file 7. Centr/East Eur Centr Asia, Central/Eastern Europe and Central Asia; CVD, cardiovascular disease; GDD, Global Dietary Database; Latin Amer/Caribbean, Latin America/Caribbean; SSBs, sugar sweetened beverages; T2D, type 2 diabetes; UIs, uncertainty intervals. Source data

In all world regions, the proportion of SSB-attributable T2D and CVD cases was highest at the youngest ages (Extended Data Fig. 2), with most pronounced variations by age in high-income countries, Latin America and the Caribbean, Middle East and North Africa, and sub-Saharan Africa. By world region and age, the highest proportional incidence due to SSBs was seen among younger adults in Latin America and the Caribbean: 43.7% (39.0–50.4) in 20–24-year-olds and 41.2% (36.1–48.8) in 25–30-year-olds. Patterns were similar for proportions of CVD attributable to SSBs (Extended Data Fig. 2). Cardiometabolic deaths and DALYs by age and world region are shown in Supplementary Figs. 7 and 8.

**Fig. 3:**
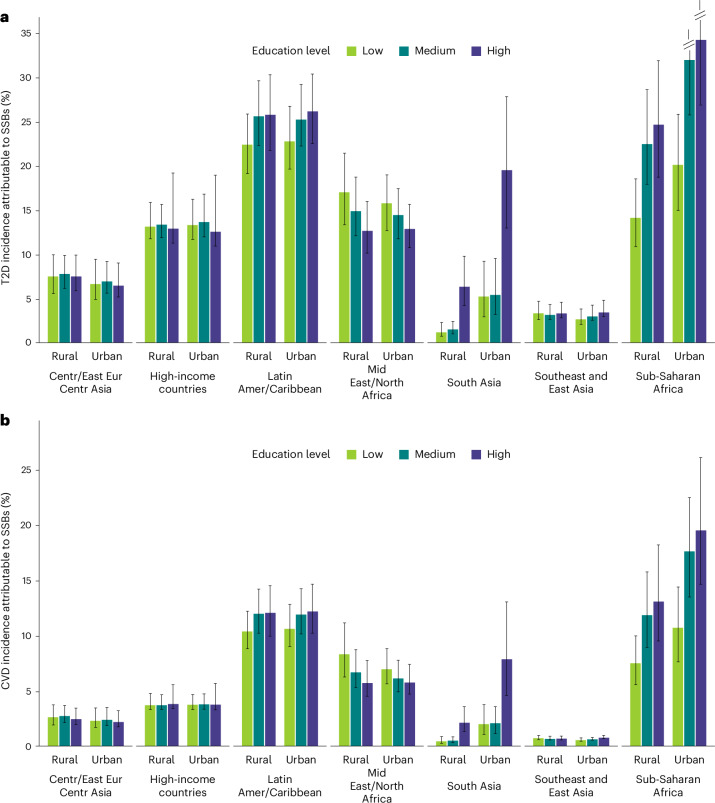
Proportional incidence of T2D and CVD attributable to SSB intake among adults (20+ years) jointly stratified by world region, area of residence and education level in 2020. **a**,**b**, The bars represent the central estimate (median) of the proportional SSB-attributable T2D incidence (**a**) and CVD incidence (**b**). The error bars represent the 95% UI derived from the 2.5th and 97.5th percentiles of 1,000 multiway probabilistic Monte Carlo model simulations. Values were truncated at 35 for **a**, and 95% UIs above 35 are shown with diagonal lines. Source data are provided in Source Data Fig. 3. Source data

Cardiometabolic burdens due to SSB varied by education and urban or rural residence across world regions (Fig. 3). When education, urbanicity and world region were jointly considered, it was revealed that the highest proportions of incident T2D attributable to SSBs were among high-educated (34.2% (26.8–42.7)) and mid-educated (31.9% (25.7–38.4)) adults in urban sub-Saharan Africa, followed by high- and medium-educated adults in both urban and rural Latin America and the Caribbean (~26% each). Our findings were similar for CVD, with the largest SSB-attributable proportions among higher-educated (19.5% (14.6–26.1)) and mid-educated (17.6 % (13.5–22.4)) adults from urban areas in sub-Saharan Africa, but also among higher-educated and mid-educated adults from rural areas in sub-Saharan Africa and both urban and rural areas in Latin America and the Caribbean (~12–13% each). Patterns for SSB-attributable deaths and DALYs were consistent with these results (Supplementary Figs. 9 and 10).

### Changes over time in T2D and CVD attributable to SSBs

**Fig. 4:**
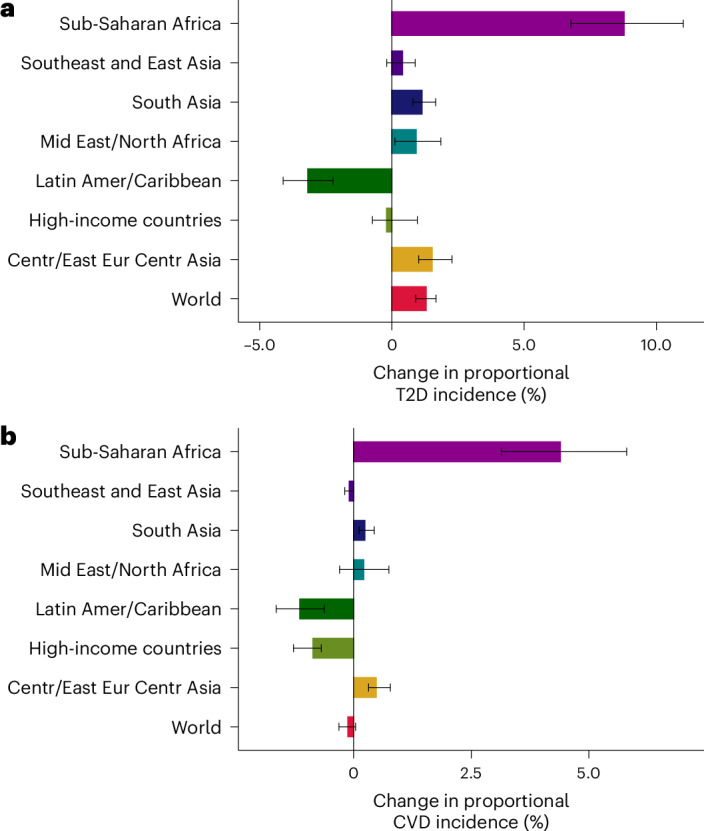
Change in proportional incidence of T2D and CVD attributable to intake of SSBs among adults (20+ years) from 1990 to 2020 by world region. **a**,**b**, The bars represent the central estimate (median) of the difference between 1990 and 2020 for the proportional T2D incidence (**a**) and CVD incidence (**b**) attributable to SSB. The error bars represent the 95% UI derived from the 2.5th and 97.5th percentiles of 1,000 multiway probabilistic Monte Carlo model simulations. Source data are provided in Source Data Fig. 4. Source data

Globally from 1990 to 2020, the proportion of T2D incidence attributable to SSBs increased by 1.3% absolute percentage points (0.9–1.7), and that of CVD decreased by −0.1% (−0.3 to 0.0). By region, the greatest percentage increase in T2D and CVD burdens due to SSBs was in sub-Saharan Africa (Fig. 4 and Supplementary Data 2), where SSB-attributable T2D increased by 8.8 percentage points (6.8–11.0) and CVD by 4.4 percentage points (3.1–5.8). More moderate increases were also identified in the Middle East and North Africa and Central or Eastern Europe and Central Asia. By contrast, T2D and CVD proportional burdens were generally stable over time in other regions, while Latin America and the Caribbean and high-income countries experienced a slight decrease. Similar patterns were identified in SSB-attributable deaths and DALYs (Supplementary Fig. 11).

**Fig. 5:**
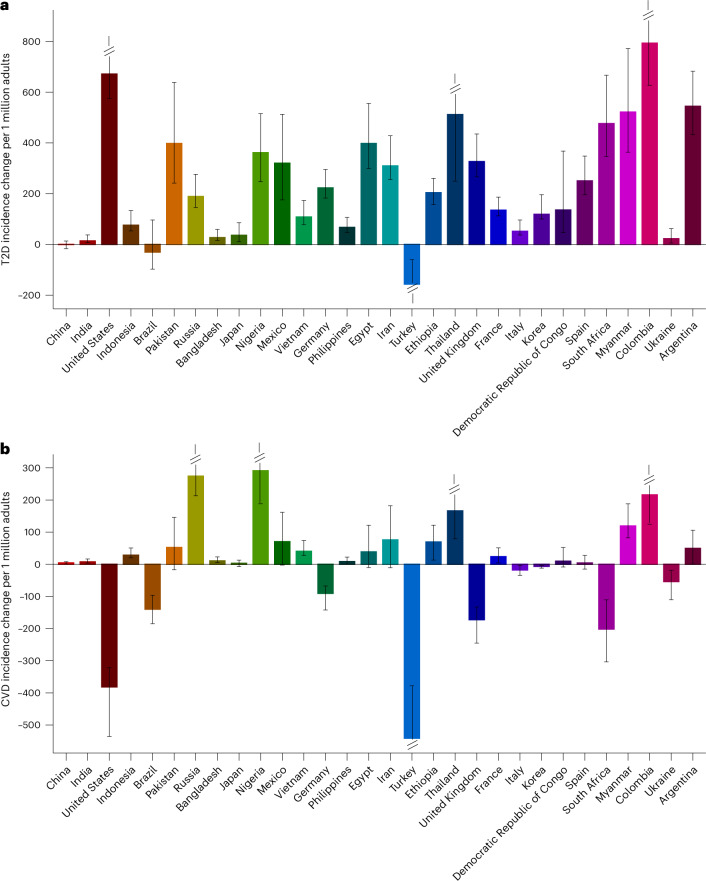
Change in incident cases per 1 million adults of T2D and CVD attributable to SSB intake among adults (20+ years) from 1990 to 2020 among the 30 most populous countries. **a**,**b**, The bars represent the central estimate (median) of the difference between 1990 and 2020 of the absolute SSB-attributable T2D incidence (**a**) and CVD incidence (**b**) per 1 million adults. The error bars represent the 95% UI derived from the 2.5th and 97.5th percentiles of 1,000 multiway probabilistic Monte Carlo model simulations. Values were truncated from −200 to 870 for T2D (**a**) and from −545 to 300 for CVD (**b**). The 95% UIs above or below these values are shown with diagonal lines. The SSB-attributable absolute burden per 1 million adults was calculated by dividing the country absolute number of SSB-attributable cases by the country adult population (20+ years) in that same year and multiplying by 1 million. The difference in the absolute burden per 1 million adults was calculated by subtracting per 1 million adult burdens in 1990 from per 1 million adult burdens in 2020. From left to right, the countries are ordered from most to least populous based on 2020 adult (20+ years) population data. Source data are provided in Source Data Fig. 5. Source data

Among the 30 most populous countries, the largest increase over time in SSB-attributable new T2D cases per 1 million adults was in Colombia with 793 more cases (627–972), followed by the United States (671 (576–985)), Argentina (544 (432–682)), Myanmar (522 (364–772)) and Thailand (512 (249–982)) (Fig. 5). Incident SSB-attributable CVD cases increased most in Nigeria (291 (188–464)), Russia (274 (213–414)), Colombia (216 (125–335)) and Thailand (166 (79.6–340)). By contrast, Turkey (−156 (−234, −59.7)) experienced the largest decrease in incident T2D due to SSBs, while the largest reductions in incident CVD due to SSBs were in Turkey (−541 (−789, −378)), the United States (−382 (−536, −322)), South Africa (−202 (−304, −111)) and the United Kingdom (−172 (−245, −133)). Changes over time in SSB-attributable T2D and CVD deaths and DALYs among the 30 most populous countries are presented in Supplementary Figs. 12 and 13.

**Extended Data Fig. 3:**
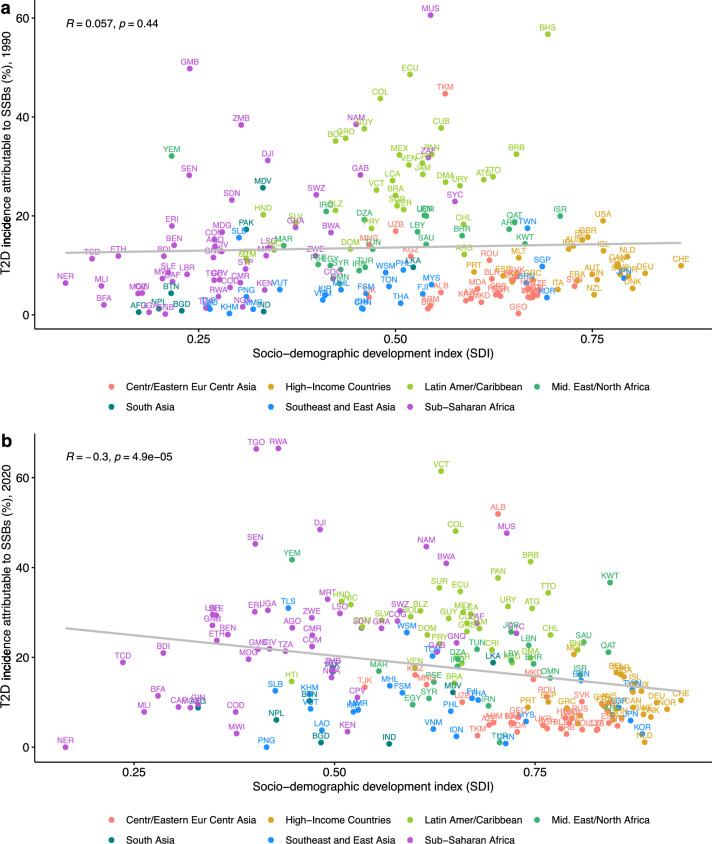
National correlation of proportional SSB-attributable T2D incidence and SDI in 1990 and 2020. **(a)** 1990 and **(b)** 2020. Points represent the 184 countries included in this analysis (labeled with their ISO3 code and colored based on world region). The gray line represents the overall linear association, with Spearman correlation coefficient and associated P value (two-tailed) provided. No adjustments were made for multiple comparisons. SDI is a measure of a nation’s development expressed on a scale of 0 to 1 sourced from the Global Burden of Disease study, based on a compositive average of the rankings of income per capita, average educational attainment and fertility rates. In prior GDD reports, the region Central/ Eastern Europe and Central Asia was referred as Former Soviet Union, and Southeast and East Asia was referred to as Asia. See Supplementary Table 1 for a list of countries included in each world region. Source data are provided in Source Data file 8. Source data

**Extended Data Fig. 4:**
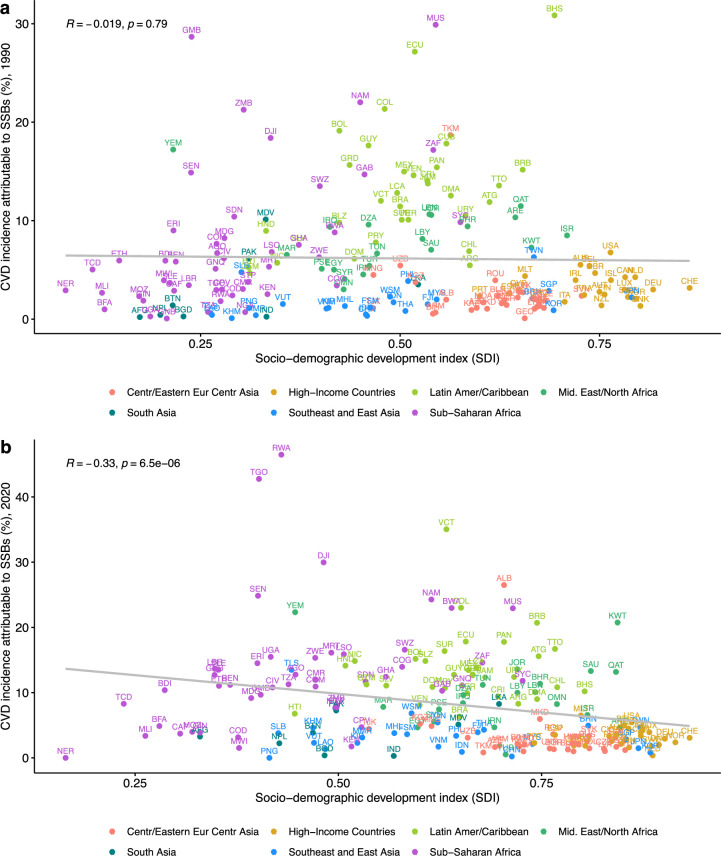
National correlation of proportional SSB-attributable CVD incidence and SDI at the national level in 1990 and 2020. **(a)** 1990 and **(b)** 2020. Points represent the 184 countries included in this analysis (labeled with their ISO3 code and colored based on world region). The gray line represents the overall linear association, with Spearman correlation coefficient and associated p value (two-tailed) provided. No adjustments were made for multiple comparisons. SDI is a measure of a nation’s development expressed on a scale of 0 to 1 sourced from the Global Burden of Disease study, based on a compositive average of the rankings of income per capita, average educational attainment and fertility rates. In prior GDD reports, the region Central/ Eastern Europe and Central Asia was referred as Former Soviet Union, and Southeast and East Asia was referred to as Asia. See Supplementary Table 1 for a list of countries included in each world region. Source data are provided in Source Data file 8. Centr/Eastern Eur Centr Asia, Central/Eastern Europe and Central Asia; CVD, cardiovascular disease; GDD, Global Dietary Database; Latin Amer/Caribbean, Latin America/Caribbean; SDI, sociodemographic development index; SSBs, sugar sweetened beverages; UIs, uncertainty intervals. Source data

To better understand relationships of these trends with national development, we investigated findings by sociodemographic index (SDI), a composite measure of national development based on fertility rate under age 25, mean education level among people aged 15+ years and income per capita^18^. In 1990, the national SDI was not correlated with SSB-attributable T2D or CVD disease burdens. However, by 2020, the national SDI was inversely correlated with both incident T2D (*r* = −0.30, *P* < 0.01) and CVD (*r* = −0.33, *P* < 0.01), with higher SSB-attributable health burdens occurring among nations with lower SDI (Extended Data Figs. 3 and 4, Supplementary Figs. 14–17 and Supplementary Data 3).

## Discussion

This investigation provides updated estimates of T2D and CVD health burdens attributable to SSBs worldwide, with stratification by age, sex, education, and rural or urban residence^3^. Globally, we found that 2.2 million new cases of T2D and 1.2 million new cases of CVD in 2020 were attributable to SSBs—representing about 1 in 10 new T2D and 1 in 30 new CVD cases. In addition, we estimated that about 340,000 people died in 2020 from SSB-related T2D and CVD. Important trends over time were identified by world region and demographic subgroups.

These SSB-attributable health burdens are informed by independently derived etiologic effects incorporated into our CRA model, identified from published meta-analyses of prospective cohort studies and randomized controlled trials of SSBs and cardiometabolic outcomes. A larger global effect on T2D than on CVD can be explained by the greater impact of adiposity on T2D versus CVD, as well as T2D onset generally occurring at younger ages (when SSB intakes are much higher) compared with CVD. Also, competing risk factors for CVD, such as smoking, LDL cholesterol and blood pressure, lower the relative impact of SSBs on CVD compared with T2D^19^.

We identified important heterogeneity in these cardiometabolic disease burdens. Regionally, sub-Saharan Africa experienced the largest increases in SSB-attributable burdens between 1990 and 2020, while Latin America and the Caribbean, despite modest declines over this period, retained significant burdens compared with other regions. We also identified relatively high SSB-related health burdens among individuals with higher educational attainment in Latin America and the Caribbean, South Asia and sub-Saharan Africa; lower educational attainment in the Middle East and North Africa; and both urban and rural residence in South Asia and sub-Saharan Africa. Proportional burdens were largest among younger versus older adults in most world regions, whereas absolute burdens were more substantial among middle-aged and older adults. Proportional SSB-attributable burdens were lower among older adults owing to other competing risk factors for cardiometabolic disease later in life, absolute numbers of cases and deaths were substantial.

The large SSB-attributable burdens in sub-Saharan Africa are consistent with reports of substantial increases in SSB intakes and, separately, in cardiometabolic disease rates in the region between 1990 and 2020^12,20^. Our findings provide a clarion call that the ‘nutrition transition’ from traditional toward Western diets has already occurred in much of the region, requiring urgent policy and public health attention. Yet, many sub-Saharan African nations have not implemented any measures to curb SSB intakes, perhaps owing to both industry opposition and previous lack of credible country-specific data^21,22^. As SSB intakes have leveled or started to decline in high-income nations^12^, the beverage industry has turned to emerging markets where populations are highly susceptible to marketing appeal of aspirational ‘Western’ lifestyles^23,24^. Critically, we found in many such regions that SSB-attributable heath burdens are now largest among more educated adults, in line with regional economic growth and burgeoning middle classes^23,25^. In South Africa, for example, SSB advertising is mainly directed at wealthy males under 45 years old, who also show the highest SSB consumption in the country^26^. Although South Africa has implemented an SSB tax, the beverage industry increased their advertising to offset negative effects on sales^27^. Independent advertising regulations—that is, not designed or policed by industry—have been largely missing^26,27^. Our findings highlight the need to address high and increasing health burdens from SSBs in these nations. Our results further suggest, given higher intakes and health burdens among more versus less educated adults in many regions, that general education alone is unlikely to effectively reduce SSB intakes (and could even augment intakes and health burdens).

The modest decreases in SSB-related cardiometabolic burdens identified in Latin America and the Caribbean are consistent with slowly decreasing consumption of SSBs in this region^12^. Nations in this region have implemented several policy efforts targeting SSBs, including taxes, marketing regulations, front-of-package warnings and education campaigns^28,29^. Yet, SSB-attributable health burdens remain high in the region, and absolute burdens per million adults continue to rise owing to continuing increased rates in obesity, T2D and CVD. While declining SSB intake may have slowed regional growth in obesity, SSB intake remains high, while other risks such as high refined grain intake and physical inactivity remain prevalent^30^. Given the lag between obesity and development of T2D and CVD, as well as the persistent impact of early life influences on later cardiometabolic risk, concerted multigenerational efforts over many years may be needed to reverse these challenges.

Compared with other regions, South Asia and Southeast and East Asia had the lowest SSB-attributable cardiometabolic burdens, consistent with their much lower intakes^12^. Other competing dietary risks, such as large T2D burdens attributable to refined rice^31^, may also lower relative SSB-attributable burdens. In line with these findings, a set of prospective cohorts across 21 countries found that incident T2D associated with refined rice intake was highest in South Asia^32^. Our lack of data on sweetened teas could also partly underestimate burdens attributable to these beverages, given their expanding market in Asia^33^.

In nations with high SSB intakes and T2D rates among younger adults, resulting attributable burdens will be greater. Too large or too low nation-specific findings should be interpreted within their estimated uncertainty ranges. For example, Colombia had high central estimates of SSB-attributable burdens (48.1% for T2D incidence; 23.0% for CVD incidence), and these estimates’ lower bounds (39.3% and 18.0%, respectively) are also plausible SSB-attributable burdens in this country. Given imperfections of global modeling, a reasonable conclusion is that Colombia’s SSB-attributable T2D harms are at least as high as in other high-burden nations such as Mexico (30.0%) or South Africa (27.6%).

Between 1990 and 2020, SSB-attributable T2D incidence increased 1.3% globally while SSB-attributable CVD incidence remained stable, although with wide variations by region and nation. For instance, sub-Saharan Africa experienced an increase in T2D incidence due to SSBs of 8.8 percentage points, while Latin America and the Caribbean experienced a decrease of −3.2 percentage points. Among largely populated nations, the largest increases in SSB-related T2D incidence was in Colombia, USA and Argentina, and in CVD incidence, Nigeria, Russia, Colombia and Thailand. These changes generally align with rises in SSB consumption in these nations, except in the US where slight declines in SSB consumption were offset by increased burdens of diabetes^12^. Similarly, declining SSB-related cardiometabolic burdens in Turkey, Brazil, and the United States and the United Kingdom for CVD are consistent with their decreasing SSB consumption from 1990 to 2020^12^.

Commercial interests of multinational and local SSB manufacturers, who invest in supply chain and marketing efforts to increase sales while opposing policy efforts to curb intakes, are probably drivers of increased SSB intakes and attributable cardiometabolic burdens in many nations^34^. Mexico faces industry opposition to its soda tax, including industry-supported reports questioning the efficacy of the tax to reduce intakes and suggesting harms to jobs and the national economy, as well as amplified marketing through advertising, price reductions and bonus products^34^. Colombia’s 2016 efforts to pass an SSB tax were blocked by industry opposition, although renewed efforts were successful in 2022^35^. In Nigeria, increased SSB intake has motivated studies looking into potential SSB taxation and other policies to curb intakes^36^. However, increasing SSB sales in Nigeria have likewise motivated the expansion of the soda industry, supported by the vulnerability of the youthful population and the availability of relevant natural resources for SSB production^37^. The case of Nigeria highlights a reality that growth in SSB sales frequently greatly outpaces consideration, development and implementation of countering policies. When Thailand implemented an SSB tax in 2017 in response to high intakes, promising effects were seen among older and lower-income individuals, but not in youth^38^, which may relate to heavy advertising targeting youth in Thailand^39^. In the United Kingdom, a 2018 SSB graduated tax encouraged industry reformulation to lower sugar content, but the total volume of SSBs purchased went up^40^, potentially owing to counteracting industry marketing strategies^41^. Beyond commercial interests, insufficient availability of safe drinking water can contribute to rising SSB-related health burdens in many nations, such as among rural areas in Colombia, Mexico and Thailand^42–44^. Clean water scarcity and commercial interests can go hand in hand: in one Mexican town, water scarcity was partly attributable to water concessions for soda companies^45^. Our findings show the downstream health consequences, which are high and often rising, of these realities.

Addressing cardiometabolic diseases is crucial to several United Nations 2030 Sustainable Development Goals, including promoting good health and well-being, reducing inequalities, promoting responsible consumption and reducing poverty^46^. Our research contributes to these endeavors by quantifying SSB-attributable cardiometabolic burdens, including population subgroups with the largest risk and increases over time, to more accurately inform national strategies. Policies targeting the food environment, including taxes and food labeling, are gaining traction and can influence consumer behavior^47–49^. Taxation based on sugar density also influences product reformulation, promoting the availability of lower-sugar options^50^. Currently, a higher proportion of people in low- and lower-middle-income countries is exposed to SSB taxes compared with those living in upper-middle-income and high-income countries^51^. Yet, these taxes are generally new (many implemented after 2018) and often authorized at low levels to raise revenue, rather than higher levels needed to reduce consumption. Our findings also reveal that lower-SDI nations are more likely to have higher T2D and CVD SSB-attributable burdens than higher-SDI nations—a worrisome change since 1990, and consistent with other recent reports^52^. Lower-SDI countries can face significant constraints in policy implementation, including lower tax rates, implementation oversight and administration capacity^53^, as well as challenging population-wide access to clean water, which may hinder the declines in SSB consumption^54^. Our findings suggest the need for national and multilateral design, implementation and evaluation of policy to reduce SSBs, such as taxes, front-of-package labeling, marketing regulations, school food regulations and water sanitation efforts^55,56^, with particular focus on nations and subgroups with the largest SSB-attributable cardiometabolic burdens.

Some industry segments have initiated sugar reduction in certain products and regions^57,58^. In Europe, Coca-Cola and PepsiCo have cut sugar contents by 30–50% in some products including Sprite and 7-Up^58^. SSB marketing and distribution are often replaced by those for beverages with nonnutritive sweeteners. While short-term weight effects of the latter may be less problematic, growing evidence suggests that such compounds are not innocuous and may have harms on the microbiome and glucose tolerance^59–61^. Given the widespread and increasing use of nonnutritive sweeteners, additional research on their health effects is critically needed, as well as public health messaging for avoidance whenever possible.

The GBD recently estimated, among other risk factors, SSB-attributable cardiometabolic burdens globally^52,62,63^, although incidence was not reported, which is the primary focus of our study. Considering deaths, the GBD estimated 52,882 SSB-attributable T2D deaths in 2020, representing 3.4% of total T2D deaths^63^. In comparison, we estimated 80,278 SSB-attributable T2D deaths, or 5.1% of the total. The GBD estimated only 13,691 SSB-attributable CVD deaths in 2020, compared with 257,962 in our analysis. Important methodologic differences between these estimates include (1) our use of a much larger number of individual-level dietary surveys with data on SSBs (GDD: 450; GBD: 44)^64^; (2) individual dietary data derived from more countries (GDD: 118; GBD: 17); (3) our use of individual-level surveys as the primary input to estimate global SSB intakes, rather than sales data and Food and Agriculture Organization (FAO) food balance estimates of national per capita added sugar availability used by GBD^12,65^; and (4) our incorporation of updated findings on both direct and BMI-mediated etiologic effects of SSBs, compared with GBD that did not include mediation by BMI^65^. We consider the latter to be important given effects of SSBs on adiposity, contributions of overweight and obesity to cardiometabolic risk, and the fact that SSB–disease estimates in the literature generally adjust for BMI^2,17^. Ultimately, differing careful global investigations such as these are critical to help triangulate the reality on the ground in the absence of uniformly available data in all nations, while similarities and differences between studies can help identify how differences in reasoned assumptions and methods might influence results.

Our study has several strengths. To our knowledge, previous investigations have not reported global, regional and national estimates of T2D and CVD SSB-attributable burdens jointly stratified by age, sex, education, and urban or rural residence. About 85% of dietary inputs were derived from individual-level dietary surveys (that is, 24 h recalls, food records and food frequency questionaries), and uncertainty in the individual data sources, their methods and representativeness is incorporated into our hierarchical Bayesian model to help address potential bias from less reliable dietary collection methods, variability in representativeness and survey level stratification, and sampling and model uncertainties. Compared with previous studies, our final GDD dietary estimates are stratified by education and area of residence, in addition to sex and age. Our CRA incorporated etiologic effects of SSBs from meta-analyses of prospective cohorts or randomized trials that facilitate inference of independence and temporality, rather than performing cross-sectional correlational analyses that can be strongly limited by reverse causation and cross-national confounding. The cohort-derived effects were multivariable adjusted for sociodemographic, lifestyle and other dietary factors and are consistent with randomized trials of SSBs, representing reasonable estimates of long-term health effects. Studies of etiologic effects with increased risk of bias, such as retrospective or cross-sectional studies, were excluded^2^. We incorporated sampling and modeling uncertainty from each of our model inputs, providing central estimates and measures of uncertainty representing the 95% most probable values^12^.

Limitations should be considered. Our estimates are based on best available data and reasoned assumptions, and do not prove cause and effect. The CRA framework is not a microsimulation estimating the impact of a specific intervention to reduce future SSB intakes, but a counterfactual approach that estimates the health effects of current SSB intakes compared with the scenario in which such an exposure was not present. While etiologic effects of SSBs on weight gain, T2D and CVD were obtained from multiple cohorts across world regions, these were mostly from high-income countries especially for weight gain that used pooled US studies, which could imperfectly represent other populations if health effects of SSBs are in the future shown to biologically vary by world region or over time. While we varied such estimates by age and baseline BMI, current evidence is likewise insufficient to vary such risk estimates by other population characteristics. Although etiologic effects were obtained from multivariable-adjusted studies and, where available, were consistent with findings from trials, measurement error, residual confounding and publication bias cannot be ruled out, which could alter findings in unpredictable directions. We did not incorporate other likely SSB-related health harms, such as dental caries, other effects of adiposity, hepatic steatosis or microbiome dysfunction; thus, our findings probably underestimate the full health burdens of SSBs. Despite extensive efforts of the GDD, dietary data were limited for several time periods and countries, particularly lower-income nations^12^. Accordingly, estimated burdens in countries with fewer individual-level surveys have higher uncertainty. All dietary assessments include some error; however, validated methods such as multiple 24 h recalls, food records and food frequency questionaries included in the GDD are considered realistic and reliable tools for individual-level dietary collection in large-scale demographic studies^66^. Our SSB definition did not include 100% fruit juices or sugar-sweetened milk, which have shown inconsistent evidence for cardiometabolic effects^2,9^. Global dietary surveys often did not collect information on sugar-sweetened tea or coffee, highlighting a future surveillance need, particularly in Asia^33^.

In summary, our study offers a comprehensive analysis of the global burden of SSB-attributable T2D and CVD, incorporating sociodemographic disparities and regional nuance. The largest proportional T2D and CVD attributable burdens in 2020 were in Latin America and the Caribbean and sub-Saharan Africa, and the largest increases from 1990 to 2020 were in sub-Saharan Africa. These findings emphasize the need for targeted interventions, accounting for social inequities and aligned with global health objectives. While some policies to curb SSB intakes are currently in place in some countries, our study suggests that more work is needed. By highlighting the countries and subpopulations most affected, our research can assist in shaping effective policies and interventions to ultimately reduce the cardiometabolic heath burdens of SSBs globally.

## Methods

### Inclusion and ethics statement

Data informing the GDD modeling estimates for this study, including from LMICs (low- and middle-income countries), were collected between 1980 and 2018 from GDD consortium members and publicly available sources in the form of dietary intake surveys. If nationally representative surveys were not available for a country, we also considered national surveys without representative sampling, followed by regional, urban or rural surveys, and finally large local cohorts, provided that selection and measurement biases were not apparent limitations (for example, excluding studies focused on a selected population with a specific disease, a certain profession or following a particular dietary pattern). For countries with no surveys identified, other sources of potential data were considered, including the WHO Infobase, the STEP database and household budget survey data. As of August 2021, we identified and retrieved 1,634 eligible survey years of data from public and private sources. Of these, 1,224 were checked, standardized and included in the GDD model, including 450 surveys informing SSB intake estimates^12^.

Most surveys identified were privately held or, if public, not available in relevant format for GDD modeling (for example, not jointly stratified by age, sex, education, and urban or rural status). We thus relied almost entirely on direct consortium member contacts for each survey to provide us with exposure data directly. Roles and responsibilities of GDD consortium members were determined and agreed upon before data sharing as part of a standardized data sharing agreement. The draft manuscript was shared with all GDD consortium members before submission for peer review, and all members are included as coauthors of this work. We endorse the Nature Portfolio journals’ guidance on LMIC authorship and inclusion and are committed to the inclusion of researchers from LMICs in publications from the GDD. We share the GDD data with the entire consortium, encourage authors from LMICs to take the lead on analyses and papers, and can provide technical and writing support to LMIC authors. For more details on the collaborative GDD data collection process, please visit our website at https://www.globaldietarydatabase.org/methods/summary-methods-and-data-collection.

This research is locally relevant to all countries included, given that it disaggregates findings nationally and subnationally by key demographic factors such as age, sex, education level and urbanicity, providing decision-makers with the CVD and diabetes risk associated with SSB intakes over time.

This modeling investigation was exempt from ethical review board approval because it was based on published data and nationally representative, de-identified datasets, without personally identifiable information. Individual surveys underwent ethical review board approval required for the applicable local context.

### Study design

A CRA model^14^ estimated the numbers, proportions and uncertainty of global T2D and CVD incidence, DALYs and mortality attributable to intake of SSBs among adults aged 20+ years. Importantly, the CRA framework does not use ecologic correlations to estimate risk, but incorporates independently derived input parameters and their uncertainties on sociodemographics, population size, risk factor (that is, SSBs) their multivariable-adjusted estimated etiologic effects on disease based on external studies, and background disease incidence, mortality and DALYs^14^. These parameters are entered into the model to estimate the disease burdens and their uncertainties. Specifically for this investigation, we leveraged input data and corresponding uncertainty in 184 countries including (1) population SSB intake distributions based on individual-level survey data from the GDD (https://www.globaldietarydatabase.org/)^7,12,13^; (2) optimal SSB intake levels from previous analyses^67^; (3) direct age-adjusted etiologic effects of SSBs on T2D, ischemic heart disease and ischemic stroke adjusted for BMI^2,68–70^, and of weight gain on T2D^15^, ischemic heart disease^16^ and ischemic stroke^15^ from previous meta-analyses and pooled analyses of prospective cohorts, as well as linear, BMI-stratified effects of SSBs on weight gain or loss^17^; (4) population overweight (BMI ≥ 25 kg m^−^^2^) and underweight (BMI < 18.5 kg m^−2^) distributions from the (non-communicable disease) NCD Risk Factor Collaboration (NCD-RisC)^71^; (5) total T2D, ischemic heart disease, and ischemic stroke incidence, DALYs and mortality estimate distributions from the GBD study^72,73^; and (6) population demographic data from the United Nations Population Division^74,75^ and the Barro and Lee Educational Attainment Dataset 2013^76^, as previously reported^31^ (Supplementary Table 7).

Bias and reliability were addressed in each of the independent data sources used in our model. The GDD selected national and subnational dietary surveys without apparent measurement or selection biases^7^, and leveraged a Bayesian model to incorporate differences in data comparability and sampling uncertainty. In GBD, bias adjustment of underlying rates of T2D and CVD not specifically meeting the gold-standard definition of these causes was done using network meta-regression before estimation in DisMod, while implausible or unspecified causes of death were redistributed to valid underlying causes of death using reclassification algorithms^73,77^. Etiologic effects were obtained from published meta-analyses or pooled analyses of prospective cohorts and randomized control trials including multivariable adjustment for age, sex, BMI and other risk factors to reduce bias from confounding^2,68–70^. Studies with increased risk of bias such as retrospective or cross-sectional studies were excluded^2^. Underlying adiposity rates were obtained from the NCD-RisC, which used national or subnational surveys that collected measured height and weight data to avoid bias in self-reported data^71^.

The GBD study uses a different approach to dietary assessment, primarily relying on adjusted United Nations (UN) and FAO national per capita availability of sugar as primary data to estimate SSBs, with a limited set of individual-level dietary surveys (*N* = 44). In comparison, the GDD uses a much more comprehensive database of largely individual-level dietary surveys to estimate SSB intake (*N* = 450), with other data (such as UN FAO sugar) used as covariates rather than as primary data. Thus, in addition to novel stratification by educational level and area of residence, the GDD dietary estimates may be more valid and informative. Our investigation leverages published diet–disease etiologic effects from extensive meta-analyses identified through reviews conducted by our team, includes both direct and BMI-mediated effects, and incorporates new data on prevalence of overweight and obesity from the NCD-RisC. Our study also estimates incident cases, which is not a measure reported in previous global studies.

Compared with our previous 2010 estimates^3^, our present investigation includes major expansion of individual-level dietary surveys and global coverage through 2018; updated modeling methods, covariates and validation to improve estimates of stratum-specific mean intakes and uncertainty; inclusion of updated dietary and disease data that are jointly stratified subnationally by age, sex, education level, and urban or rural residence; and updated SSB etiologic effect estimates on T2D, ischemic stroke and ischemic heart disease. This present analysis focused on adults aged 20+ years given the low rates of T2D and CVD globally at younger ages.

### Global distributions of SSB intakes

The GDD systematically searched for and compiled representative data on individual-level dietary intakes from national surveys and subnational surveys^7,12^. The final GDD model incorporated 1,224 dietary surveys representing 185 countries from 7 world regions and 99.0% of the global population in 2020. Of these, 450 surveys reported data on SSBs, totaling 2.9 million individuals from 118 countries representing 86.8% of the global population. Most surveys were nationally or subnationally representative (94.2%), collected at the individual level (84.7%), and included estimates in both urban and rural area of residence (61.6%). Further details on characteristics of surveys with data on SSBs, including availability of surveys per world region, are available in Supplementary Table 1. The world region classification used in our study was based on groupings that are likely to have consistent exposures to disease risk and rates of disease outcomes, and this or similar classifications have been previously used by our team and others^73^. Countries included in each world region are listed in Supplementary Table 2. Global, regional and national estimates among the 30 most populous countries, by population characteristics in 2020, are available in Supplementary Tables 3 and 4.

SSBs were defined as any beverages with added sugars and ≥50 kcal per 8 oz serving, including commercial or homemade beverages, soft drinks, energy drinks, fruit drinks, punch, lemonade and aguas frescas. This definition excluded 100% fruit and vegetable juices, noncaloric artificially sweetened drinks and sweetened milk. All included surveys used this definition. We used an average sugar content per SSB serving, an assumption that probably has little influence on large-scale demographic estimates such as these but could be a problem for more focused local studies. Home-sweetened teas and coffees (which often would have less than 50 kcal per serving) were not explicitly excluded from the SSB definition at the time of data collection, but total tea and coffee intake were separately collected in the dietary surveys and by the GDD as separate variables. Compared with soda and other industrial SSBs, 100% fruit juices and sugar-sweetened milk, coffee and tea have shown inconsistent evidence for health effects, and were therefore excluded from our definition of SSBs^2,9^. Differences in health effects may relate to additional nutrients in those drinks, such as calcium, vitamin D, fats, and protein in milk, caffeine and polyphenols in coffee and tea, and fiber and vitamins in 100% juice, or to differences in the rapidity of consumption and/or drinking patterns of these beverages. Notably, each of these other beverages is also generally excluded in policy and surveillance efforts around SSBs^12^. At high intakes, alcoholic beverages have been associated with T2D and CVD in prospective cohorts and genome-wide association studies^78,79^. However, the effect of alcoholic beverages on T2D and CVD differs from the effect of SSBs on these diseases, and thus, alcohol and SSB should be analyzed separately^2,79,80^. Moreover, the exclusion of alcoholic beverages ensures comparability across diverse populations, given variations in alcohol consumption due to religious and cultural factors^81^. Regulatory shortcomings in labeling 100% fruit and vegetable juices may have led to underestimations in SSB intake and attributable burdens for certain populations^82,83^.

For our present analysis, we updated SSB intake estimates for 2020 using similar methodology as previously reported^12^, but with updated food availability data released by FAO for 2014–2020 as covariates. Because FAO updated its methodology for these new estimates, the FAO estimates from this period versus their estimates from earlier years are not directly comparable (for example, a ‘step change’ in FAO estimates was noted comparing 2013 versus 2014 data for most countries). To account for this and retain the relative ranking between nations, we calculated a nation-specific adjustment factor for each FAO covariate, based on the ratio of that nation’s 2013 versus 2014 data, and applied this to each nation’s FAO estimates from 2014 to 2020.

A Bayesian model with a nested hierarchical structure (with random effects by country and region) was used to estimate the mean consumption level of SSBs and its statistical uncertainty for each of 264 population strata across 185 countries from 1990 through 2020, incorporating and addressing differences in data comparability and sampling uncertainty^12,84^. The model then estimated intakes jointly stratified by age (22 age categories from 0 to 6 months through 95+ years), sex (female, male), education (≤6 years of education, >6 years to 12 years, >12 years) and urbanicity (urban, rural). Although this analysis focuses only on adults aged 20+ years, the model used all age data to generate the strata estimates.

Of the 188 countries with survey data, 3 were dropped from the GDD estimation model owing to unavailability of FAO food availability data (Andorra, Democratic People’s Republic of Korea and Somalia), an important covariate in the estimation model. Uncertainty of each stratum-specific estimate was quantified using 4,000 iterations to determine posterior distributions of mean intake jointly by country, year and sociodemographic subgroup. The median intake and the 95% UI for each stratum were computed at the 50th, 2.5th and 97.5th percentiles of the 4,000 draws, respectively.

Global, regional, national and within-country population subgroup intakes of SSBs and their uncertainty were calculated as population-weighted averages using all 4,000 posterior estimates for each of the 264 demographic strata in each country–year. Population weights for each year were derived from the United Nations Population Division^74,75^, supplemented with data for education and urban or rural status from a previous study^85^. Intakes were calculated as 8 oz (248 g) servings per week. For our present analysis, GDD SSB estimates were collapsed for adults aged 85+ years using the 4,000 simulations corresponding to the stratum-level intake data derived from the Bayesian model. In this study, regression-based methods were used to estimate the standard deviation corresponding to each estimated, stratum-specific mean from the dietary survey input data. These mean–standard deviation pairs were then used to generate gamma distribution parameters for usual dietary intake as detailed in the following section.

### Estimation of gamma parameters for the distribution of usual intake

Dietary intakes cannot be negative, and the usual intake distributions tend to be skewed to the right^86,87^. Gamma distributions were shown to be more appropriate than normal distributions for SSBs based on the analysis of GDD input data (for example, NHANES data) in a previous study^88^ and other research on assessment of population dietary intake^89,90^, as it is nonnegative and includes a wide range of shapes with varying degrees of skewness^91^. Standard deviation (s.d.) needed to be obtained to construct the gamma distribution of intakes. Parameters for gamma distribution were generated using the mean estimate from the GDD estimation model and the estimated s.d. for the mean estimate from 1,000 simulations.

#### Standard deviation estimates for the distribution of usual dietary intake

Stratum-level GDD input survey data were used to fit a linear regression of the s.d. of intake on mean intake (both adjusted for total energy). To determine the appropriate transformation of the input data used for fitting the linear regression, scatter plots of energy-adjusted means versus energy-adjusted s.d. were created. Using this approach, we concluded that a natural log transformation for both mean and s.d. was most appropriate. We also explored excluding demographic and health surveys, household surveys and outlier data owing to the potential unreliability of such surveys for estimating s.d., but determined that no one dietary assessment method contributed unevenly to the observed linear trend. Thus, all available data were included, allowing for the largest possible sample size and greatest generalizability. We also investigated whether the log mean and log s.d. relationship differed by world region, but did not find strong evidence for such heterogeneity. A regression model was used for each individual diet factor to calculate the s.d.:

$${Y}_{i}=\,{\beta }_{0}+{\beta }_{1}{x}_{i}+{\varepsilon }_{i}$$

where *i* refers to each survey stratum, *Y*_*i*_ is the natural log of the s.d. of stratum-specific intake, *x*_*i*_ is the natural log of the mean of stratum-specific intake and *ε*_*i*_ is the random error that follows *N*(0, *σ*^2^).

#### Monte Carlo simulations for generating standard deviation distributions

Estimates for *β*_0_ and *β*_1_ were used to predict 1,000 ln(s.d.) values corresponding to 1,000 iterations (*k*) of the predicted mean intake for each population stratum (*j*) using Monte Carlo simulations.

$${\widehat{Y}}_{{jk}}=\,{\widehat{\beta}}_{0}+{\widehat{\beta}}_{1}{\widehat{X}}_{jk}$$

in which $\widehat{{X}_{{jk}}}$ is the *k*th sample draw of the posterior distribution for mean intake for population stratum *j*. We propagated uncertainty from the model estimates, as well as variation within the sampling data itself, by randomly drawing from a *t-*distribution with *n* *−* 1 degrees of freedom using the following equation:

$$\mathrm{ln}\left(\widehat{{\rm{{s.d.}}}_{{jk}}}\right)=\,{\hat{Y}}_{{jk}}+\,\widehat{{\rm{\sigma }}}\sqrt{1+\left(\frac{1}{n}\right)\times}\, {t}_{k}^{n-1},$$

in which $\hat{\sigma }$ is the estimate for *σ, n* is the number of survey strata, ${t}_{k}^{n-1}$ is the *k*th sample drawn from a *t*-distribution with *n* − 1 degrees of freedom and $\widehat{{s.d.}_{{jk}}}$ is the *k*th sample draw of the predicted s.d. distribution for population stratum *j*.

#### Estimation of gamma parameters for the distribution of usual intake

The posterior distributions for each stratum-specific s.d. were used to generate 1,000 corresponding shape and rate gamma parameters for the distribution of usual intake, a primary input in the CRA model, using the following equations:

$$\widehat{{\rm{Shape}}_{{jk}}}={\left(\frac{\widehat{{X}_{{jk}}}}{\widehat{{\rm{s.d.}}_{{jk}}}}\right)}^{2}$$

$$\widehat{{\rm{Rate}}_{{jk}}}=\,\frac{\widehat{{X}_{{jk}}}}{\widehat{{\rm{s.d.}}_{{jk}}^{2}}}$$

### Estimated SSB–disease relationships

The direct risk estimates between SSB intake and T2D, ischemic heart disease and ischemic stroke were obtained from published systematic reviews and evidence grading, based on published meta-analyses of prospective cohort studies and randomized controlled trials including multivariable adjustment for age, sex, BMI and other risk factors to reduce bias from confounding (Supplementary Table 8)^2,68–70^. The methods and results for the review, identification and assessment of evidence for the SSB–disease relationships have been described^2,67^. Briefly, evidence for each SSB–disease relationship was first evaluated by grading the quality of evidence according to nine different Bradford Hill criteria for causation: strength, consistency, temporality, coherence, specificity, analogy, plausibility, biological gradient and experiment. This evidence grading was completed independently and in duplicate by two expert investigators. Based on these assessments, probable or convincing evidence was determined independently and in duplicate, in accordance with the criteria of the FAO and World Health Organization^92^ and with consideration of consistency with similar criteria of the World Cancer Research Fund and the American Institute for Cancer Research^93^. SSBs had at least probable association for direct etiologic effects (BMI independent) on T2D, ischemic heart disease and ischemic stroke risk, as well as on weight gain. See Supplementary Table 9 for further details on the evidence grading criteria and results of this evaluation. All SSB–disease estimates were standardized from the originally reported 250 ml serving size to 8 oz servings (248 g), the unit used in our analysis.

Given that these studies adjusted for BMI, we separately assessed the BMI-mediated effects (BMI change in kg m^−^^2^) based on pooled analyses from long-term prospective cohort studies of changes in diet and changes in BMI (Supplementary Table 8)^17^. Specifically, we used data from three separate prospective cohort studies: the Nurses’ Health Study (1986–2006), involving 50,422 women with 20 years of follow-up; the Nurses’ Health Study II (1991–2003), including 47,898 women with 12 years of follow-up; and the Health Professionals Follow-up Study (1986–2006) with 22,557 men with 20 years of follow-up. Participants included in these analyses were initially free of obesity (that is, BMI < 30 kg m^−^^2^) or chronic diseases and had complete baseline data on weight and lifestyle habits. The associations between SSBs and weight gain were estimated separately for overweight and obese (BMI ≥ 25 kg m^−^^2^) and non-overweight adults (BMI < 25 kg m^−^^2^), given observed effect modification by baseline BMI status^17^. We used linear regression with robust variance, accounting for within-person repeated measures, to assess the independent relationships between changes in SSB intake and changes in BMI over 4 year periods. Women who became pregnant during follow-up were excluded from the analysis. BMI-mediated effects did not specifically differentiate between overweight and obesity, which could have led to an underestimation in the BMI-mediated effects among adults with obesity.

To examine the BMI-mediated associations, we assessed the impact of differences in BMI on the risk of T2D, ischemic heart disease and ischemic stroke (Supplementary Table 8)^15,16^. These relationships were obtained from pooled analyses of multiple cohort studies investigating the quantitative effects of BMI on T2D^15^, ischemic heart disease^16^ and ischemic stroke^15^. The risk estimates were transformed from the originally reported 5 kg m^−^^2^ to 1 kg m^−2^.

### Heterogeneity in diet–disease relationships using age-specific relative risks

Age-specific relative risks were calculated for each SSB–disease etiologic relationship based on evidence showing decreasing proportional effects of metabolic risk factors on cardiometabolic disease incidence at older ages (for example, due to other competing risk factors)^15,67^. The age-specific relative risks were calculated based on the age at event and were assumed to have a log-linear age association, although the true age relationship may differ.

To calculate the age at event for each SSB–disease pair, we obtained relevant data from the original studies. This included the average age at baseline in years, the follow-up time in years, the type of follow-up time reported (for example, maximum, median or mean) and the study weight for each study in each meta-analysis (Supplementary Tables 10–12 and Supplementary Data 4). In cases in which the age at baseline was reported as a range rather than as the average, we used the central value to estimate the mean. If follow-up time to events was not reported, we estimated it based on the duration of the study. For studies that reported maximum follow-up time, we estimated the mean time to event as half of the maximum follow-up, and for studies that reported mean or median follow-up times, as two-thirds of the mean or median follow-up. The unweighted mean age at event for each study was calculated by summing the mean age at baseline and the appropriate mean time to event, and the weighted mean age at event for the meta-analysis as the weighted age at event across all studies. In cases in which specific studies were excluded from the meta-analysis owing to limitations in study quality, or when the meta-analysis was conducted for multiple outcomes, the weights were adjusted accordingly. When study weights were not reported, we assigned equal weights to each study when calculating the mean overall age at event.

Given limited evidence of significant effect modification by sex, we incorporated similar proportional effects of risk factors by sex^67^. In previous research, we evaluated the proportional differences in relative risk for key diet-related cardiometabolic risk factors, including systolic blood pressure, BMI, fasting plasma glucose and total cholesterol, across six 10 year age groups from 25–34 years to 75+ years^67^. Given similarities across these four risk factors, the mean proportional differences in relative risk across all risk factors were applied to the SSB–disease relative risks. In this study, we disaggregated the mean proportional differences into 14 5 year age groups from 20–24 years to 85+ years. This was achieved by linearly scaling between each 10 year mean proportional difference in log relative risk, anchoring at the calculated mean age at event for each SSB–disease.

We used Monte Carlo simulations to estimate the uncertainty in the age-distributed log relative risk, sampling from the distribution of log relative risk at the age at event. On the basis of 1,000 simulations, we used the 2.5th and 97.5th percentiles to derive the 95% UI.

### Global distributions of adiposity

Prevalence of overweight (BMI ≥ 25 kg m^−^^2^) and underweight (BMI < 18.5 kg m^−^^2^) in each country–year–age–sex–urbanicity stratum and their uncertainty was obtained from the NCD-RisC. The NCD-RisC collected data from 1,820 population-based studies encompassing national, regional and global trends in mean BMI, with measurements of height and weight taken from over 97 million adults^71,94^. Surveys were excluded if they relied solely on self-report data, focused on specific subsets of the population or involved pregnancy. The NCD-RisC used a Bayesian hierarchical model to estimate age-specific mean BMI and prevalence of overweight and obesity by country, year and sex. The model incorporated data-driven fixed effects to account for differences in BMI by rural and urban area of residence. A Gibbs sampling Markov Chain Monte Carlo algorithm was used to fit the model, producing 5,000 samples of the posterior distributions of the model parameters. These samples were then used to generate posterior distributions of mean BMI and prevalence of overweight and obesity for each stratum. Estimates were age standardized using age weights from the WHO standard population. Weighting was also used at the global, regional and national levels, taking into account the respective age-specific population proportions by country, year and sex. The estimates of mean BMI and overweight and obesity prevalence were presented along with their respective 95% credible intervals, representing the uncertainty around the estimates. To further stratify the NCD-RisC overweight and obesity prevalence estimates by education level and urbanicity, we assumed that the prevalence did not vary across different education levels or between urban and rural residences. In addition, it was assumed that these estimates remained constant between 2016 and 2020 (as NCD-RisC reports only through 2016, but this CRA analysis assesses estimates for 2020), a conservative assumption that probably underestimates the prevalence of overweight and obesity and, thus, SSB-attributable burdens.

### Characterization of optimal intake

The optimal intake level of SSBs served as the counterfactual in our CRA modeling analysis, allowing the quantification of impacts of SSBs on disease risk at the population level. We determined the optimal intake level based on probable or convincing evidence for effects of SSBs on cardiometabolic outcomes. The methodology for defining the optimal intake level has been described^67^. Briefly, it was determined primarily based on disease risk (observed consumption levels associated with lowest disease risk in meta-analyses) with further considerations of feasibility (observed national mean consumption levels in nationally representative surveys worldwide)^95,96^, and consistency with existing major food-based dietary guidelines^97,98^. The term ‘optimal intake’ can be considered analytically analogous to what has been referred to as the ‘theoretical minimum risk exposure level’ in other analyses^99,100^. We prefer the former term as it is more relevant to dietary risks, which can serve as a benchmark for quantifying disease risk, informing dietary guidance and informing policy priorities.

### Global distributions of T2D, ischemic heart disease and ischemic stroke

The estimates of underlying cardiometabolic disease burdens at global, regional and national levels were obtained from the GBD 2021. The GBD collected data from censuses, household surveys, civil registration, vital statistics and other relevant records to estimate incidence, prevalence, mortality, years lived with disability (YLDs), years of life lost (YLLs) and DALYs for 371 diseases and injuries^73^. These estimates were stratified by 204 countries and territories, 23 age groups and sex, yearly from 1990 to 2021. For this analysis, we used GBD estimates of incidence, mortality and DALYs for T2D, ischemic heart disease and ischemic stroke for 1990 and 2020. The GBD defined T2D as fasting plasma glucose greater than or equal to 126 mg dl^−1^ (7 mmol l^−1^) or reporting the use of diabetes medication^73^. Estimated cases of type 1 diabetes were subtracted from the overall diabetes cases at the most stratified level of age, sex, location and year to estimate T2D cases. Ischemic heart disease was estimated in the GBD as the aggregate of myocardial infraction (heart attack), angina (chest pain) or ischemic cardiomyopathy (heart failure due to ischemic heart disease). Ischemic stroke was defined as rapidly developing clinical signs of (usually focal) cerebral function disturbance lasting over 24 h, or leading to death, according to the WHO criterion of sudden occlusion of arteries supplying the brain due to a thrombus^101^.

GBD mortality estimates were generated using the Cause of Death Ensemble Model framework, which incorporated various models including different sets of covariates testing the predictive validity, and generating cause-specific mortality estimates^73,102,103^. Cause of Death Ensemble Model estimates were scaled among all causes such that the sum of cause-specific deaths did not exceed all-cause mortality. YLLs were calculated as the product of the number of deaths for each cause by age, sex, location and year times the standard life expectancy. Life expectancy was first decomposed by cause of death, location and year to represent the cause-specific effects on life expectancy^102^. Then, the sum across age groups was taken to estimate the impact of a given cause on the at-birth life expectancy from 1990 to 2021. Incidence was modeled using DisMod, a meta-regression tool that used epidemiologic data to estimate the occurrence disease within a population and determines whether cases remain prevalent, go into remission or result in death. YLDs were calculated by splitting the prevalence of each cause into mutually exclusive sequela, each defined by a health state; each health state was then weighted by the corresponding disability weight^73^. Finally, DALYs were calculated as the sum of YLLs and YLDs.

### Disaggregation of T2D and CVD burdens by education level and urbanicity

The GBD provides underlying disease estimates at global, regional and national levels for 1990 to 2021, jointly stratified by age and sex. Extensive previous evidence shows that T2D and CVD outcomes vary by educational attainment and urbanicity^104–122^. We further stratified the 1990 and 2020 GBD estimates by education level (low, medium, high) and area of residency (urban, rural) to examine potential variations in risk within these subpopulations and to align with the demographic and GDD dietary data stratifications available. This approach required assumptions on distributions of disease burdens by these demographic factors and potentially underestimated uncertainty in our results stratified by these factors.

To stratify the GBD estimates, we conducted a search of scientific literature to identify recent meta-analysis, pooled analyses and large surveys evaluating the association between educational attainment and urbanicity with the risk of T2D and CVD. Because we hypothesized that country income level was a potential effect modifier for the relationships of educational attainment and urbanicity with T2D and CVD risk, we further collected and collated risk estimates stratified by country income level. We limited our analysis to studies adjusting only for age and sex, when possible, to avoid the attenuating effects of adjusting for additional covariates^104–122^.

We conducted fixed-effects meta-analysis of collated effect sizes (associations between education or urbanicity and disease rates), stratified by country income level. Published estimates were standardized to high versus low education level, matched as closely as possible to the GDD definitions (low: 0–6 years of education; high: >12 years of education), as well as to urban versus rural residence. We pooled estimates within studies when (1) multiple estimates were reported for different CVD outcomes, (2) separate estimates were provided for men and women, (3) estimates were reported for different locations (except by country income) or (4) an intermediate category matched our definitions for education level or area of residence. The characteristics of the studies used to calculate the effect estimates, including their original and calculated effect sizes, can be found in Supplementary Data 5 and 6 for education level and area of residence, respectively.

We conducted a separate fixed-effect meta-analysis for the relationship of education or urbanicity to T2D and CVD, stratified by country income level. We distributed the central estimate of our meta-analyzed risk estimate equally for high versus low education, and urban versus rural residence, by taking its square root and inverse square root (Supplementary Table 13). This approach assumed similar differences from high to medium education as from medium to low education. We also explored distributing the central estimate by incorporating information on the actual distance (for example, grade years) from high to medium education and medium to low education, when such information was available. As the results did not appreciably differ, we used the square root and inverse square root approach to maintain consistency across studies, particularly given heterogeneity in categorizations of education levels. The final calculated effect estimates for the association between education level and area of residence with T2D and CVD, by income country level, can be found in Supplementary Table 13.

The T2D, ischemic heart disease and ischemic stroke estimates for each year–country–age–sex stratum (mean and 95% UI) were multiplied by their respective population proportion, education effect and urban effect. This process created six de novo strata with the raw (unscaled) fully proportioned burden estimates and their uncertainty. The global population proportions for each year were derived from the United Nations Population Division^75^, supplemented with data on education attainment from a previous study^76^. Finally, to prevent under- or overestimation of the absolute number of T2D, ischemic heart disease and ischemic stroke cases globally, the raw fully proportioned burden estimates were scaled to match the total burden estimate for each stratum. This scaling ensured that the overall burden estimates remained consistent. Supplementary Table 14 provides a fictitious, illustrative example of how 1,000 T2D cases in a single age–sex population stratum (low-income country) in a given year were disaggregated into the 6 finer education–urbanicity strata using the central estimate of the meta-analyzed education and urban effects. The population proportioned only burden estimates is also provided as a comparison. While uncertainty was incorporated in all the modeling parameters, we were unable to include uncertainty in the stratification of T2D and CVD cases by educational attainment and urban or rural residence as rigorous data to do so were not available.

### Statistical analysis: CRA analysis

The CRA framework incorporated the data inputs and their uncertainty to estimate the absolute number, rate (per million adults 20+ years) and proportion of T2D, ischemic heart disease and ischemic stroke cases attributable to intake of SSBs in 1990 and 2020 (Supplementary Fig. 18). For each stratum, the model calculated the percentage (population attributable fraction (PAF)) of total T2D, ischemic heart disease and ischemic stroke incidence, mortality and DALYs attributable to intake of SSBs. For BMI-mediated effects, the model considered the associations between observed SSB intakes and changes in BMI at the stratum level. This association was weighted by the prevalence of overweight (BMI ≥ 25), normal weight (BMI >18.5 to <25) and underweight (BMI < 18.5; assumed to have no effect) in each stratum. The resulting weighted BMI change was combined with the relative risk (RR) of BMI change and T2D or CVD using the same continuous PAF formula. Further details on each calculation for the PAF can be found in the sections below.

Given that summing direct and BMI-mediated PAFs would overestimate the combined effect, for each disease stratum (that is, country–year–age–sex–education–residence), the PAF was calculated using proportional multiplication of the direct and BMI-mediated PAFs as follows:

$${\rm{PAF}}=1-\left(\left(1-{\rm{direct}}\; {\rm{PAF}}\right)\times\left(1-{{\text{BMI-mediated}}\, {\text{PAF}}}\right)\right)$$

The resulting PAF was then multiplied by the corresponding number of disease cases to calculate the attributable burden in each stratum. Findings were evaluated globally, regionally and nationally, and by specific population subgroups of age, sex, education and urbanicity. The results are presented as proportional attributable burden (percentage of cases) and attributable rate (per one million adults). This representation of the proportional multiplication for a single risk factor (that is, SSBs) is equivalent to the formula commonly reported for several risk factors: ${\rm{PAF}}=1-\,\mathop{\prod}\nolimits_{i=1}^{n}1-{\rm{PAF}}_{i}$

#### Direct-effect PAF

The PAF formula is used to quantify the burden of disease attributable to a particular exposure. It involves comparing the disease cases associated with the observed exposure levels in the population to a counterfactual scenario with an optimal intake distribution, given a known etiologic exposure–disease risk relationship.

In this analysis, we aimed to estimate the burden of incidence, mortality and DALYs for T2D, ischemic heart disease and ischemic stroke attributable to intake of SSBs.

$${\rm{PAF}}=\frac{{\int }_{\!x=0}^{m}{\rm{RR}}\left(x\right)P\left(x\right){dx}-1}{{\int }_{\!x=0}^{m}{\rm{RR}}\left(x\right)P\left(x\right){dx}}\,,$$

The PAF formula used is as follows:

$${\rm{PAF}}=\frac{{\int }_{\!x=0}^{m}{\rm{RR}}\left(x\right)P\left(x\right){dx}-1}{{\int }_{\!x=0}^{m}{\rm{RR}}\left(x\right)P\left(x\right){dx}}\,,$$

where *P*(*x*) is the usual SSB intake distribution in a specific population stratum, assumed to follow a gamma distribution as used in previous analyses^3,31,88^; RR(*x*) is the age-specific relative risk function for T2D or CVD risk; and *m* is the maximum exposure level.

RR(*x*) is defined as:

$$\left\{\begin{array}{cc}\exp (\beta (x-y(x))) & :x-y(x)\ge 0\\ 1 & :x-y(x) < 0\end{array}\right.$$

where *β* is the stratum-specific change in log relative risk per unit of exposure, *x* is the current exposure level and *y*(*x*) is the optimal exposure level. *y*(*x*) is defined to be ${F}_{\rm{optimal}}({F}_{x}^{-1}\left(x\right))$, where *F*_optimal_ is the cumulative distribution function of the optimal intake and ${F}_{x}^{-1}$ is the inverse cumulative distribution function of the current exposure distribution. Implicit in how we characterize the relative risk function are certain assumptions, including a linear relationship between the log relative risk (beta) and the unit of exposure. This model assumes that no further risk is associated with exposure beyond the optimal intake level, and that both *x* and the optimal intake level for an individual at exposure level *x* are the *q*th quantile of their respective distributions (the observed exposure distribution and the optimal intake distribution, respectively).

#### PAF calculation

In practice, simple numerical integration using Riemann sums can be used to compute the integrals in the PAF formula^88^.

$${\rm{PAF}}=\frac{{\sum }_{i=1}^{n}{P}_{i}({\rm{RR}}_{i}-1)}{{\sum }_{i=1}^{n}{P}_{i}({\rm{RR}}_{i}-1)+1}$$

$${\rm{PAF}}=\frac{{\sum }_{i=1}^{n}{P}_{i}({\rm{RR}}_{i}-1)}{{\sum }_{i=1}^{n}{P}_{i}({\rm{RR}}_{i}-1)+1}$$

*n* categories are determined by dividing the exposure range (chosen here to be 0, ${F}_{x}^{-1}\left.\left(\varPhi \left(-6\right)\right)\right)$ into 121 intervals, each of length 0.1 when converted to the standard normal scale (except for the first one). *Φ* is defined as the cumulative distribution function of the standard normal distribution (*N*(0,1)). More precisely, the range of exposure groups *i* can be described as:

$$\left({0,\,F}_{x}^{-1}\left(\varPhi \left(-6\right)\right)\right)\,\qquad\qquad:i=1$$

$$\left({F}_{x}^{-1}\left(\varPhi \left(-6+0.1\left(i-2\right)\right)\right),{F}_{x}^{-1}\left(\varPhi \left(-6+0.1\left(i-1\right)\right)\right)\right)\,\qquad:i=1$$

#### BMI-mediated effects PAF

The association of change in BMI with change in SSB intake was assessed in three pooled US cohorts using multivariate linear regression accounting for within-person repeated measures, as described in an earlier study^17^. Separate linear relationships were estimated for underweight (BMI < 18.5), normal weight (BMI > 18.5 to <25) and overweight (BMI ≥ 25 to <30), given observed effect modification by baseline BMI status^17^. Because individuals with obesity were excluded in these previous analyses, we used the risk estimate for individuals with overweight for individuals with obesity, which could underestimate the full effects of SSB on weight change.

To assess the BMI-mediated effects of SSB intake on incidence, mortality and DALYs of T2D, ischemic heart disease and ischemic stroke, we first calculated the monotonic effect of SSB intake on BMI change for each population stratum by weighting the baseline BMI-specific effect by the respective prevalence of underweight, normal weight and overweight (including obesity) within each stratum. We obtained overweight and underweight population distributions from the NCD-RisC^71^ and calculated the prevalence of normal weight as 1 minus the sum of these prevalences^71^. The NCD-RisC estimates go up to 2016, and thus, for our 2020 analysis, we used data from 2016 as a proxy for 2020. Given increasing adiposity globally, this assumption could result in underestimation of disease burdens due to SSBs in 2020. We assumed that individuals with underweight did not experience increased risk of T2D, ischemic heart disease or ischemic stroke with increased consumption of SSBs. As such, the monotonic effect for this population segment was set at 0:

$$\begin{array}{l}{\text{SSB}}-{\text{to}}-{\text{BMI}} {\text{effect}}={\beta }_{\rm{BMI}\ge 25}\,\times \,\left({\rm{overweight}}\; {\text{prevalence}}\right)+\,{\beta }_{{\rm{BMI}}18.5-25}\,\\\qquad\qquad\qquad\qquad\qquad\times \,\left({\rm{normal}}\; {\text{weight}}\; {\text{prevalence}}\right)+0\,\\\qquad\qquad\qquad\qquad\qquad\times \,\left({\rm{underweight}}\; {\text{prevalence}}\right)\end{array}$$

We then estimated the BMI-mediated log(RR) by multiplying the log(RR) per unit increase in BMI and the SSB-to-BMI effect (associated increase in BMI per one-unit-associated increase in SSB intake).

#### Quantification of uncertainty using Monte Carlo simulations

We used Monte Carlo simulations to quantify the uncertainty around the PAF estimate. In this calculation, we incorporated uncertainty of multiple key parameters, including the usual intake distribution of SSBs in each stratum; underlying T2D, CVD and DALY burden estimates in each stratum; the etiologic estimates (RR) for SSB–BMI, SSB–T2D and SSB–CVD relationships; and the prevalence of individuals with underweight, normal weight or overweight in each stratum. For each SSB–disease combination and stratum, we drew randomly 1,000 times from the respective probability distributions. This included drawing randomly from the normal distribution of the estimate of disease-specific changes in the log(RR) of BMI-mediated and direct etiologic effects for a one-unit increase in SSB intake, the posterior distributions for shape and rate parameters for usual dietary intake and the normal distribution of the estimate for the prevalence of underweight, normal weight and overweight. Draws of proportions that were less than 0 or greater than 1 were truncated at 0 or 1, respectively, and draws of mean intake that were 0 or less were truncated at 0.00001. Each set of random draws was used to calculate the PAFs and, multiplied by the stratum-specific disease rates, the associated absolute attributable disease burden. Corresponding 95% UIs were derived from the 2.5th and 97.5th percentiles of 1,000 estimated models.

### Sociodemographic development index

We assessed national-level findings by SDI in 1990 and 2020. The SDI is a composite measure of a nation’s development based on factors such as income per capita, educational attainment and fertility rates^18^.

### Changes in SSB-attributable burdens over time

To compare estimates across different years (1990 and 2020), we calculated differences for absolute and proportional burdens from 1990 to 2020 (that is, 2020 minus 1990). We performed this calculation for each simulation resulting in a distribution of differences, and we report the median and 95% UIs for each difference. We did not formally standardize comparisons over time by age or sex. This decision was made to ensure that findings would reflect the actual population differences in attributable burdens that are relevant to policy decisions. However, we also performed analyses stratified by age and sex, taking into account changes in these demographics over time. All analyses were conducted using R statistical software, R version 4.4.0 (ref. ^123^) on the Tufts High Performance Cluster.

### Reporting summary

Further information on research design is available in the Nature Portfolio Reporting Summary linked to this article.

## Online content

Any methods, additional references, Nature Portfolio reporting summaries, source data, extended data, supplementary information, acknowledgements, peer review information; details of author contributions and competing interests; and statements of data and code availability are available at 10.1038/s41591-024-03345-4.

## Supplementary information

Supplementary Information Supplementary Tables 1–14 and Figs. 1–18.

Reporting Summary

Supplementary Data 1 Proportional and absolute T2D and CVD burdens attributable to SSBs in 1990 and 2020 globally, regionally and nationally.

Supplementary Data 2 Differences in proportional and absolute CVD and T2D burdens attributable to SSBs from 1990 to 2020 globally, regionally and nationally.

Supplementary Data 3 Sociodemographic development index and proportional T2D and CVD burdens attributable to SSBs in 1990 and 2020.

Supplementary Data 4 Age-at-event calculation for the etiologic effect of BMI on ischemic heart disease.

Supplementary Data 5 Study characteristics and effect estimates from studies assessing the association between education level with T2D and CVD risk for disaggregating GBD disease estimates.

Supplementary Data 6 Study characteristics and effect estimates from studies assessing the association between area of residence with T2D and CVD risk for disaggregating GBD disease estimates.

## Source data

Source Data Fig. 1 Source data.

Source Data Fig. 2 Source data.

Source Data Fig. 3 Source data.

Source Data Fig. 4 Source data.

Source Data Fig. 5 Source data.

Source Data Extended Data Fig. 1 Source data.

Source Data Extended Data Fig. 2 Source data.

Source Data Extended Data Fig. 3 and 4 Source data.

## Data Availability

Data used in this analysis are publicly available from the following sources: (1) population SSB intake distributions based on individual-level survey data from the GDD (https://www.globaldietarydatabase.org/data-download)^7,12^; (2) optimal SSB intake levels from previous analyses^67^; (3) direct age-adjusted etiologic effects of SSBs on T2D, ischemic heart disease and ischemic stroke adjusted for BMI^2^, and of weight gain on T2D^15^, ischemic heart disease^16^ and ischemic stroke^15^ from previous meta-analyses and pooled analyses of prospective cohorts, as well as linear, BMI-stratified effects of SSBs on weight gain or loss^17^; (4) population overweight (BMI ≥ 25 kg m^−^^2^) and underweight (BMI < 18.5 kg m^−^^2^) distributions from the NCD-RisC (https://ncdrisc.org/data-downloads.html)^71^; (5) total T2D, ischemic heart disease and ischemic stroke incidence, DALYs, and mortality estimate distributions from the GBD study (https://vizhub.healthdata.org/gbd-results/)^72,73^; and (6) population demographic data from the United Nations Population Division (UN, https://population.un.org/wpp/)^74,75^, the Barro and Lee Educational Attainment Dataset 2013 (10.3386/w15902)^76^ and SDI data (Global Health Data Exchange: GBD, https://ghdx.healthdata.org/record/ihme-data/gbd-2019-socio-demographic-index-sdi-1950-2019). The GDD SSB intake data were collapsed for 85+ years using the 4,000 simulations corresponding to the stratum-level intake data derived from the Bayesian model. These data were used to obtain the gamma parameters of the SSB intake distribution used in the model. The 4,000 simulation files can be made available to researchers upon request. Eligibility criteria for such requests include utilization for nonprofit purposes only, for appropriate scientific use based on a robust research plan and by investigators from an academic institution. If you are interested in requesting access to the data, please submit the following documents: (1) proposed research plan (please download and complete the proposed research plan form: https://www.globaldietarydatabase.org/sites/default/files/manual_upload/research-proposal-template.pdf), (2) data sharing agreement (please download this form: https://www.globaldietarydatabase.org/sites/default/files/manual_upload/tufts-gdd-data-sharing-agreement.docx and complete the highlighted fields; have someone who is authorized to enter your institution into a binding legal agreement with outside institutions sign the document; note that this agreement does not apply when protected health information or personally identifiable information is shared), and (3) email items 1 and 2 to info@globaldietarydatabase.org. Please use the subject line ‘GDD Data Access Request’. Once all documents have been received, the GDD team will be in contact with you within 2–4 weeks regarding subsequent steps. Data will be shared as .csv or .xlsx files, using a compressed format when appropriate. Source data are provided with this paper.

## Code availability

Custom code was developed using R (version 4.4.0) for this research including calculation of age-adjusted relative risks, SSB intake gamma parameters, CRA analysis (including the PAF and attributable mortality for each stratum) and data visualizations. Given the computational size, calculation of the SSB intake gamma parameters, CRA modeling, absolute and relative differences, and summary statistics were run on the Tufts University High Performance Computing Cluster (https://it.tufts.edu/high-performance-computing), supported by the National Science Foundation (grant: 2018149, https://www.nsf.gov/awardsearch/showAward?AWD_ID=2018149&HistoricalAwards=false) under active development by Research Technology, Tufts Technology Services (https://it.tufts.edu/researchtechnology.tufts.edu). The statistical code can be made available to researchers upon request. Eligibility criteria for such requests include utilization for nonprofit purposes only, for appropriate scientific use based on a robust research plan and by investigators from an academic institution. GDD will nominate coauthors to be included in any papers generated using GDD-generated statistical code. If you are interested in requesting access to the statistical code, please submit the following documents: (1) proposed research plan (please download and complete the proposed research plan form: https://www.globaldietarydatabase.org/sites/default/files/manual_upload/research-proposal-template.pdf), (2) data sharing agreement (please download this form: https://www.globaldietarydatabase.org/sites/default/files/manual_upload/tufts-gdd-data-sharing-agreement.docx and complete the highlighted fields; have someone who is authorized to enter your institution into a binding legal agreement with outside institutions sign the document; note that this agreement does not apply when protected health information or personally identifiable information is shared) and (3) email items 1 and 2 to info@globaldietarydatabase.org. Please use the subject line ‘GDD Code Access Request’. Once all documents have been received, the GDD team will be in contact with you within 2–4 weeks regarding subsequent steps. Data will be shared as .csv or .xlsx files, using a compressed format when appropriate.
