# Author Correction: Burdens of type 2 diabetes and cardiovascular disease attributable to sugar-sweetened beverages in 184 countries

**DOI:** 10.1038/s41591-025-03524-x

**Published:** 2025-01-28

**Authors:** Laura Lara-Castor, Meghan O’Hearn, Frederick Cudhea, Victoria Miller, Peilin Shi, Jianyi Zhang, Julia R. Sharib, Sean B. Cash, Simon Barquera, Renata Micha, Dariush Mozaffarian, Global Dietary Database, Antonia Trichopoulou, Murat Bas, Jemal Haidar Ali, Tatyana El-Kour, Anand Krishnan, Puneet Misra, Nahla Hwalla, Chandrashekar Janakiram, Nur Indrawaty Lipoeto, Abdulrahman Musaiger, Farhad Pourfarzi, Iftikhar Alam, Celine Termote, Anjum Memon, Marieke Vossenaar, Paramita Mazumdar, Ingrid Rached, Alicia Rovirosa, María Elisa Zapata, Roya Kelishadi, Tamene Taye Asayehu, Francis Oduor, Julia Boedecker, Lilian Aluso, Emanuele Marconi, Laura D’Addezio, Raffaela Piccinelli, Stefania Sette, Johana Ortiz-Ulloa, J. V. Meenakshi, Giuseppe Grosso, Anna Waskiewicz, Umber S. Khan, Kenneth Brown, Lene Frost Andersen, Anastasia Thanopoulou, Reza Malekzadeh, Neville Calleja, Anca Ioana Nicolau, Cornelia Tudorie, Marga Ocke, Zohreh Etemad, Mohannad Al Nsour, Lydiah M. Waswa, Maryam Hashemian, Eha Nurk, Joanne Arsenault, Patricio Lopez-Jaramillo, Abla Mehio Sibai, Albertino Damasceno, Pulani Lanerolle, Carukshi Arambepola, Carla Lopes, Milton Severo, Nuno Lunet, Duarte Torres, Heli Tapanainen, Jaana Lindstrom, Suvi Virtanen, Cristina Palacios, Noel Barengo, Eva Roos, Irmgard Jordan, Charmaine Duante, Corazon Cerdena, Imelda Angeles-Agdeppa, Josie Desnacido, Mario Capanzana, Anoop Misra, Ilse Khouw, Swee Ai Ng, Edna Gamboa Delgado, Mauricio T. Caballero, Johanna Otero, Hae-Jeung Lee, Eda Koksal, Idris Guessous, Carl Lachat, Stefaan De Henauw, Ali Reza Rahbar, Alison Tedstone, Annie Ling, Beth Hopping, Catherine Leclercq, Christian Haerpfer, Christine Hotz, Christos Pitsavos, Coline van Oosterhout, Debbie Bradshaw, Dimitrios Trichopoulos, Dorothy Gauci, Dulitha Fernando, Elzbieta Sygnowska, Erkki Vartiainen, Farshad Farzadfar, Gabor Zajkas, Gillian Swan, Guansheng Ma, Hajah Masni Ibrahim, Harri Sinkko, Isabelle Sioen, Jean-Michel Gaspoz, Jillian Odenkirk, Kanitta Bundhamcharoen, Keiu Nelis, Khairul Zarina, Lajos Biro, Lars Johansson, Leanne Riley, Mabel Yap, Manami Inoue, Maria Szabo, Marja-Leena Ovaskainen, Meei-Shyuan Lee, Mei Fen Chan, Melanie Cowan, Mirnalini Kandiah, Ola Kally, Olof Jonsdottir, Pam Palmer, Philippos Orfanos, Renzo Asciak, Robert Templeton, Rokiah Don, Roseyati Yaakub, Rusidah Selamat, Safiah Yusof, Sameer Al-Zenki, Shu-Yi Hung, Sigrid Beer-Borst, Suh Wu, Widjaja Lukito, Wilbur Hadden, Xia Cao, Yi Ma, Yuen Lai, Zaiton Hjdaud, Jennifer Ali, Ron Gravel, Tina Tao, Jacob Lennert Veerman, Mustafa Arici, Demosthenes Panagiotakos, Yanping Li, Gülden Pekcan, Karim Anzid, Anuradha Khadilkar, Veena Ekbote, Irina Kovalskys, Arlappa Nimmathota, Avula Laxmaiah, Balakrishna Nagalla, Brahmam Ginnela, Hemalatha Rajkumar, Indrapal Meshram, Kalpagam Polasa, Licia Iacoviello, Marialaura Bonaccio, Simona Costanzo, Yves Martin-Prevel, Nattinee Jitnarin, Wen-Harn Pan, Yao-Te Hsieh, Sonia Olivares, Gabriela Tejeda, Aida Hadziomeragic, Le Tran Ngoan, Amanda de Moura Souza, Daniel Illescas-Zarate, Inge Huybrechts, Alan de Brauw, Mourad Moursi, Augustin Nawidimbasba Zeba, Maryam Maghroun, Nizal Sarrafzadegan, Noushin Mohammadifard, Lital Keinan-Boker, Rebecca Goldsmith, Tal Shimony, Gudrun B. Keding, Shivanand C. Mastiholi, Moses Mwangi, Yeri Kombe, Zipporah Bukania, Eman Alissa, Nasser Al-Daghri, Shaun Sabico, Rajesh Jeewon, Martin Gulliford, Tshilenge S. Diba, Kyungwon Oh, Sihyun Park, Sungha Yun, Yoonsu Cho, Suad Al-Hooti, Chanthaly Luangphaxay, Daovieng Douangvichit, Latsamy Siengsounthone, Pedro Marques-Vidal, Peter Vollenweider, Constance Rybak, Amy Luke, Nipa Rojroongwasinkul, Noppawan Piaseu, Kalyana Sundram, Jeremy Koster, Donka Baykova, Parvin Abedi, Sandjaja Sandjaja, Fariza Fadzil, Noriklil Bukhary Ismail Bukhary, Pascal Bovet, Yu Chen, Norie Sawada, Shoichiro Tsugane, Lalka Rangelova, Stefka Petrova, Vesselka Duleva, Ward Siamusantu, Lucjan Szponar, Hsing-Yi Chang, Makiko Sekiyama, Khanh Le Nguyen Bao, Sesikeran Boindala, Jalila El Ati, Ivonne Ramirez Silva, Juan Rivera Dommarco, Luz Maria Sanchez-Romero, Simon Barquera, Sonia Rodríguez-Ramírez, Nayu Ikeda, Sahar Zaghloul, Anahita Houshiar-rad, Fatemeh Mohammadi-Nasrabadi, Morteza Abdollahi, Khun-Aik Chuah, Zaleha Abdullah Mahdy, Alison Eldridge, Eric L. Ding, Herculina Kruger, Sigrun Henjum, Milton Fabian Suarez-Ortegon, Nawal Al-Hamad, Veronika Janská, Reema Tayyem, Bemnet Tedla, Parvin Mirmiran, Almut Richter, Gert Mensink, Lothar Wieler, Daniel Hoffman, Benoit Salanave, Shashi Chiplonkar, Anne Fernandez, Androniki Naska, Karin De Ridder, Cho-il Kim, Rebecca Kuriyan, Sumathi Swaminathan, Didier Garriguet, Anna Karin Lindroos, Eva Warensjo Lemming, Jessica Petrelius Sipinen, Lotta Moraeus, Saeed Dastgiri, Sirje Vaask, Tilakavati Karupaiah, Fatemeh Vida Zohoori, Alireza Esteghamati, Sina Noshad, Suhad Abumweis, Elizabeth Mwaniki, Simon G. Anderson, Justin Chileshe, Sydney Mwanza, Lydia Lera Marques, Samuel Duran Aguero, Mariana Oleas, Luz Posada, Angelica Ochoa, Alan Martin Preston, Khadijah Shamsuddin, Zalilah Mohd Shariff, Hamid Jan Bin Jan Mohamed, Wan Manan, Bee Koon Poh, Pamela Abbott, Mohammadreza Pakseresht, Sangita Sharma, Tor Strand, Ute Alexy, Ute Nöthlings, Indu Waidyatilaka, Ranil Jayawardena, Julie M. Long, K. Michael Hambidge, Nancy F. Krebs, Aminul Haque, Liisa Korkalo, Maijaliisa Erkkola, Riitta Freese, Laila Eleraky, Wolfgang Stuetz, Laufey Steingrimsdottir, Inga Thorsdottir, Ingibjorg Gunnarsdottir, Lluis Serra-Majem, Foong Ming Moy, Corina Aurelia Zugravu, Elizabeth Yakes Jimenez, Linda Adair, Shu Wen Ng, Sheila Skeaff, Regina Fisberg, Carol Henry, Getahun Ersino, Gordon Zello, Alexa Meyer, Ibrahim Elmadfa, Claudette Mitchell, David Balfour, Johanna M. Geleijnse, Mark Manary, Laetitia Nikiema, Masoud Mirzaei, Rubina Hakeem

**Affiliations:** 1 Food Is Medicine Institute, Friedman School of Nutrition Science and Policy, Tufts University, Boston, MA USA; 2 Institute of Health Metrics and Evaluation, University of Washington, Seattle, WA USA; 3 Food Systems for the Future Institute, Chicago, IL USA; 4 Department of Medicine, McMaster University, Hamilton, Ontario Canada; 5 Population Health Research Institute, Hamilton, Ontario Canada; 6 Center for Surgery and Public Health, Brigham and Women’s Hospital, Boston, MA USA; 7 Friedman School of Nutrition Science and Policy, Tufts University, Boston, MA USA; 8 Research Center on Nutrition and Health, National Institute of Public Health, Cuernavaca, Mexico; 9 University of Thessaly, Volos, Greece; 10 Tufts University School of Medicine, Boston, MA USA; 11 Department of Medicine, Tufts Medical Center, Boston, MA USA; 12 Academy of Athens, Athens, Greece; 13 Acibadem University, Istanbul, Turkey; 14 Addis Ababa University, Addis Ababa, Ethiopia; 15 Aga Khan Foundation, Geneva, Switzerland; 16 All India Institute of Medical Sciences, New Delhi, India; 17 American University of Beirut, Beirut, Lebanon; 18 Amrita School of Dentistry, Eranakulum, India; 19 Andalas University, Padang, Indonesia; 20 Arab Center for Nutrition, Manama, Bahrain; 21 Ardabil University of Medical Sciences, Ardabil, Iran; 22 Bacha Khan University, Charsadda, Pakistan; 23 Biodiversity International, Nairobi, Kenya; 24 Brighton and Sussex Medical School, Brighton, UK; 25 Center for Studies of Sensory Impairment Aging and Metabolism (CeSSIAM), Guatemala City, Guatemala; 26 Centre For Media Studies, New Delhi, India; 27 Centro de Atencion Nutricional Antimano (CANIA), Miami, FL USA; 28 Centro de Estudios sobre Nutrición Infantil (CESNI), Buenos Aires, Argentina; 29 Child Growth and Development Research Center, Isfahan University of Medical Sciences, Isfahan, Iran; 30 College of Applied Sciences, Department of Food Science and Applied Nutrition, Addis Ababa Science and Technology University, Addis Ababa, Ethiopia; 31 Consultative Group on International Agricultural Research (CGIAR), Montpellier, France; 32 Council for Agricultural Research and Economics, Research Centre for Food and Nutrition, Rome, Italy; 33 Cuenca University, Cuenca, Ecuador; 34 Delhi School of Economics, University of Delhi, Delhi, India; 35 Department of Biomedical and Biotechnological Sciences, University of Catnia, Catania, Italy; 36 Department of CVD Epidemiology, Prevention and Health Promotion, Institute of Cardiology, Warsaw, Poland; 37 Department of Community Health Sciences, Aga Khan University, Karachi, Pakistan; 38 Department of Nutrition and Institute for Global Nutrition, University of California Davis, Davis, CA USA; 39 Department of Nutrition, University of Oslo, Oslo, Norway; 40 Diabetes Center, 2nd Department of Internal Medicine, Athens University, Athens, Greece; 41 Digestive Disease Research Institute, Tehran University of Medical Sciences, Tehran, Iran; 42 Directorate for Health Information and Research, Pietà, Malta; 43 Dunarea de Jos University of Galati, Galati, Romania; 44 Dutch National Institute for Public Health and the Environment (RIVM), Bilthoven, Netherlands; 45 Eastern Mediterranean Public Health Network (EMPHNET), Amman, Jordan; 46 Egerton University, Njoro, Kenya; 47 Epidemiology and Community Health Branch, National Heart, Lung, and Blood Institute, National Institutes of Health, Bethesda, MD USA; 48 Estonian National Institute for Health Development, Tallinn, Estonia; 49 FHI360, Washington DC, USA; 50 FOSCAL and UDES, Bucaramanga, Colombia; 51 Faculty of Health Sciences, American University of Beirut, Beirut, Lebanon; 52 Faculty of Medicine, Eduardo Mondlane University, Maputo, Mozambique; 53 Faculty of Medicine, University of Colombo, Colombo, Sri Lanka; 54 Faculty of Medicine/ Institute of Public Health, University of Porto, Porto, Portugal; 55 Faculty of Nutrition and Food Sciences, University of Porto, Porto, Portugal; 56 Finnish Institute for Health and Welfare, Helsinki, Finland; 57 Florida International University, Miami, FL USA; 58 Folkhälsan Research Center, Helsinki, Finland; 59 Food Envrionment Consumer Behaviour Lever of the Alliance Bioversity International and CIAT, Nairobi, Kenya; 60 Food and Nutrition Research Institute (DOST-FNRI), Manila, Philippines; 61 Food and Nutrition Research Institute (DOST-FNRI), Taguig City, Philippines; 62 Fortis CDOC Center for Excellence for Diabetes, New Delhi, India; 63 National Diabetes, Obesity and Cholesterol Foundation (N-DOC), Diabetes Foundation (India), Fortis CDOC Hospital for Diabetes and Allied Sciences, New Delhi, India; 64 FrieslandCampina, Amersfoort, The Netherlands; 65 Fundacion Cardiovascular de Colombia, Bucaramanga, Colombia; 66 Fundacion INFANT and Consejo Nacional De Investigaciones Cientificas y Tecnicas (CONICET), Buenos Aires, Argentina; 67 Fundacion Oftalmologica de Santander (FOSCAL), Floridablanca, Colombia; 68 Gachon University, Seongnam-si, South Korea; 69 Gazi University, Ankara, Turkey; 70 Geneva University Hospitals, Geneva, Switzerland; 71 Ghent University, Ghent, Belgium; 72 Global Dietary Database Consortium, Boston, MA USA; 73 Statistics Canada, Government of Canada, Ottawa, Ontario Canada; 74 Griffith University, Gold Coast, Queensland Australia; 75 Faculty of Medicine, Hacettepe University, Ankara, Turkey; 76 Harokopio University, Athens, Greece; 77 Harvard School of Public Health, Cambridge, MA USA; 78 Department of Nutrition and Dietetics, Hasan Kalyoncu University, Gaziantep, Turkey; 79 Higher Institute of Nursing Professions and Health Techniques, Marrakesh, Morocco; 80 Hirabai Cowasji Jehangir Medical Research Institute, Pune, India; 81 ICCAS (Instituto para la Cooperacion Científica en Ambiente y Salud), Buenos Aires, Argentina; 82 ICMR-National Institute of Nutrition, Hyderabad, India; 83 IRCCS Neuromed, Pozzilli, Italy; 84 LUM University “Giuseppe Degennaro”, Casamassima, Italy; 85 Institut de Recherche pour le Developpement, Montpellier, France; 86 Institute for International Investigation, NDRI-USA, New York, NY USA; 87 Institute of Biomedical Sciences, Academia Sinica, Taipei, Taiwan; 88 Institute of Nutrition and Food Technology (INTA), University of Chile, Santiago, Chile; 89 Institute of Nutrition in Central America and Panama (INCAP), Guatemala City, Guatemala; 90 Institute of Public Health of Federation of Bosnia and Herzegovina, Sarajevo, Bosnia and Herzegovina; 91 Institute of Research and Development, Duy Tan University, Da Nang City, Vietnam; 92 School of Preventive Medicine and Public Health, Hanoi City, Vietnam; 93 Institute of Studies in Public Health, Federal University of Rio de Janeiro (UFRJ), Rio de Janeiro, Brazil; 94 Instituto Nacional de Ciencias Médicas y Nutrición Salvador Zubirán, Mexico City, Mexico; 95 Tecnologico de Monterrey, , Escuela de Medicina y Ciencias de la Salud, Mexico City, Mexico; 96 International Agency for Research on Cancer, Lyon, France; 97 International Food Policy Research Institute (IFPRI), Washington DC, USA; 98 Intitut de Recherche en Sciences de la Sante, Bobo Dioulasso, Burkina Faso; 99 Isfahan Cardiovascular Research Center, Cardiovascular Research Institute, Isfahan University of Medical Sciences, Isfahan, Iran; 100 Israel Center for Disease Control, Tel-Hashomer, Israel; 101 Institute of Nutritional Sciences, Justus Liebig University of Giessen, Giessen, Germany; 102 KLE Academy of Higher Education and Research (Deemed-to-be-University) Jawaharlal Nehru Medical College, Belagavi, India; 103 Kenya Medical Research Institute, Nairobi, Kenya; 104 King Abdulaziz University, Jeddah, Saudi Arabia; 105 King Saud University, Riyadh, Saudi Arabia; 106 University of Mauritius, Reduit, Mauritius; 107 King’s College London, London, UK; 108 Kinshasa School of Public Health, Kinshasa, Democratic Republic of Congo; 109 Korea Disease Control and Prevention Agency (KDCA), Cheongju-si, South Korea; 110 Korea University, Seoul, South Korea; 111 Kuwait Institute for Scientific Research, Safat, Kuwait; 112 Lao Tropical and Public Health Institute, Vientiane, Lao People’s Democratic Republic; 113 Lausanne University Hospital (CHUV) and University of Lausanne, Lausanne, Switzerland; 114 Leibniz Centre for Agricultural Landscape Research, Muncheberg, Germany; 115 Loyola University Chicago, Chicago, IL USA; 116 Mahidol University, Pathom, Thailand; 117 Malaysian Palm Oil Council (MPOC), Kelana Jaya, Malaysia; 118 Max Planck Institute for Evolutionary Anthropology, Leipzig, Germany; 119 Medical Center Markovs, Sofia, Bulgaria; 120 Menopause Andropause Research Center, Ahvaz Jundishapur University of Medical Sciences, Ahvaz, Iran; 121 Ministry of Health, Jakarta, Indonesia; 122 Ministry of Health, Kuala Lumpur, Malaysia; 123 Ministry of Health, Sungai Besar, Malaysia; 124 Ministry of Health, Victoria, Seychelles; 125 University Center for Primary Care and Public Health (Unisanté), Lausanne, Switzerland; 126 NYU School of Medicine, New York, NY USA; 127 National Cancer Center Institute for Cancer Control, Tokyo, Japan; 128 National Centre of Public Health and Analyses (NCPHA), Sofia, Bulgaria; 129 National Food and Nutrition Commission, Lusaka, Zambia; 130 National Food and Nutrition Institute, Warsaw, Poland; 131 National Health Research Institutes, Zhunan Township, Taiwan, Republic of China; 132 Health and Environmental Risk Division, National Institute for Environmental Studies, Tsukuba, Japan; 133 National Institute of Nutrition, Hanoi, Vietnam; 134 National Institute of Nutrition, Hyderabad, India; 135 National Institute of Nutrition and Food Technology & SURVEN RL, Tunis, Tunisia; 136 National Institute of Public Health (INSP), Cuernavaca, Mexico; 137 National Institutes of Biomedical Innovation Health and Nutrition, Osaka, Japan; 138 National Nutrition Institute, Cairo, Egypt; 139 National Nutrition and Food Technology Research Institute (NNFTRI): SBMU, Tehran, Iran; 140 National University of Malaysia (UKM), Kuala Lumpur, Malaysia; 141 Nestlé Research, Lausanne, Switzerland; 142 New England Complex Systems Institute, Cambridge, MA USA; 143 North-West University, Potchefstroom South Africa, Potchefstroom, South Africa; 144 Oslo Metropolitan University (OsloMet), Oslo, Norway; 145 Pontificia Universidad Javeriana Seccional Cali, Cali, Colombia; 146 Public Authority For Food and Nutrition, Sabah Al Salem, Kuwait; 147 Public Health Authority of the Slovak Republic, Bratislava, Slovak Republic; 148 Qatar University and University of Jordan, Doha, Qatar; 149 Queensland University of Technology, Brisbane, Queensland Australia; 150 Research Institute for Endocrine Sciences, Shahid Beheshti University of Medical Sciences, Tehran, Iran; 151 Robert Koch Institute, Berlin, Germany; 152 Rutgers University, New Brunswick, NJ USA; 153 Santé publique France, the French Public Health Agency, Saint Maurice, France; 154 Savitribai Phule Pune University (SPPU), Pune, India; 155 School of Medicine, Deakin University, Geelong, Victoria Australia; 156 School of Medicine, National and Kapodistrian University of Athens, Athens, Greece; 157 Sciensano (Belgian Public Health Institute), Brussels, Belgium; 158 Seoul National University, Seoul, South Korea; 159 St John’s Research Institute, Bangalore, India; 160 Statistics Canada, Ottawa, Ontario Canada; 161 Swedish Food Agency, Uppsala, Sweden; 162 Tabriz University of Medical Sciences, Tabriz, Iran; 163 Tallinn University, Tallinn, Estonia; 164 Taylor’s University, Subang Jaya, Malaysia; 165 Teesside University, Middlesbrough, UK; 166 Tehran University of Medical Sciences, Tehran, Iran; 167 The Hashemite University, Az Zarqa, Jordan; 168 The Technical University of Kenya, Nairobi, Kenya; 169 The University of the West Indies, Kingston, Jamaica; 170 Tropical Diseases Research Centre, Ndola, Zambia; 171 Unidad de Nutricion Publica, Macul, Chile; 172 Universidad San Sebastian, Providencia, Chile; 173 Universidad Tecnica del Norte, Ibarra, Ecuador; 174 Universidad de Antioquia, Medellin, Colombia; 175 Universidad de Cuenca, Cuenca, Ecuador; 176 Medical Sciences Department of Biochemistry, Universidad de Puerto Rico, San Juan, Puerto Rico; 177 Universiti Kebangsaan Malaysia Medical Centre, Kuala Lumpur, Malaysia; 178 Universiti Putra Malaysia, Serdang, Malaysia; 179 Universiti Sains Malaysia, Kubang Kerian, Malaysia; 180 University Kebangsaan Malaysia, Bangi, Malaysia; 181 University of Aberdeen, Aberdeen, UK; 182 University of Alberta, Edmonton, Alberta Canada; 183 University of Bergen, Bergen, Norway; 184 Department of Nutrition and Food Sciences, University of Bonn, Bonn, Germany; 185 University of Colombo, Colombo, Sri Lanka; 186 University of Colorado School of Medicine, Aurora, CO USA; 187 University of Dhaka, Dhaka, Bangladesh; 188 Department of Food and Nutrition, University of Helsinki, Helsinki, Finland; 189 University of Hohenheim, Stuttgart, Germany; 190 University of Iceland, Reykjavik, Iceland; 191 University of Las Palmas de Gran Canaria (ULPGC), Las Palmas, Spain; 192 University of Malaya, Kuala Lumpur, Malaysia; 193 University of Medicine and Pharmacy Carol Davila, Bucharest, Romania; 194 University of New Mexico Health Sciences Center, Albuquerque, NM USA; 195 University of North Carolina at Chapel Hill, Chapel Hill, NC USA; 196 University of Otago, Dunedin, New Zealand; 197 University of Sao Paulo, Sao Paulo, Brazil; 198 University of Saskatchewan, Saskatoon, Saskatchewan Canada; 199 University of Vienna, Vienna, Austria; 200 University of the Southern Caribbean, Port of Spain, Trinidad and Tobago; 201 Wageningen University, Wageningen, Netherlands; 202 Washington University in St. Louis, St. Louis, MO USA; 203 World Health Organization (WHO), Geneva, Switzerland; 204 Yazd Cardiovascular Research Centre, Shahid Sadoughi University of Medical Sciences, Yazd, Iran; 205 Ziauddin University Karachi, Karachi City, Pakistan

**Keywords:** Risk factors, Epidemiology

Correction to: *Nature Medicine* 10.1038/s41591-024-03345-4, published online 6 January 2025.

In the version of the article initially published, in the eighth paragraph of the Discussion, the text “Among large nations, the largest increases in SSB-related T2D burdens were in Mexico, Thailand and the United Kingdom, and in CVD burdens, Colombia, Nigeria, Thailand and Russia. These changes align with rises in SSB consumption in these nations^12^. Similarly, declining SSB-related cardiometabolic burdens in Brazil, the United States and the United Kingdom (for CVD) are consistent with their decreasing SSB consumption from 1990 to 2020^12^” was incorrect and has now been updated to “Among largely populated nations, the largest increases in SSB-related T2D incidence was in Colombia, USA and Argentina; and in CVD incidence, Nigeria, Russia, Colombia and Thailand. These changes generally align with rises in SSB consumption in these nations, except in the US where slight declines in SSB consumption were offset by increased burdens of diabetes ^12^. Similarly, declining SSB-related cardiometabolic burdens in Turkey, Brazil, and the United States and the United Kingdom for CVD are consistent with their decreasing SSB consumption from 1990 to 2020^12^.” Additionally, Supplementary Data 1 and 2 have been updated to remove decimals in values greater than 100. These corrections have been made to the HTML and PDF versions of the article.

