# Supplementary material for: Burdens of type 2 diabetes and cardiovascular disease attributable to sugar-sweetened beverages in 184 countries
Source: Nat Med. 2025 Jan 6;31(2):552–64. doi: 10.1038/s41591-024-03345-4 (PMC11835746; doi:10.1038/s41591-024-03345-4)
Supplement: Supplementary file 1 — Supplementary Tables 1–14 and Figs. 1–18. [file 41591_2024_3345_MOESM1_ESM.pdf]

nature medicine

Supplementary information https://doi.org/10.1038/s41591-024-03345-4

Burdens of type 2 diabetes and cardiovascular disease attributable to sugar-sweetened beverages in 184 countries

In the format provided by the authors and unedited

Burdens of type 2 diabetes and cardiovascular disease burdens to sugar-sweetened beverages in 184 countries

Supplementary Information

Table of Contents

Supplementary Table 1. Characteristics of global data sources of sugar-sweetened beverage intakes. 4

Supplementary Table 2. Countries by world region (superregion) in GDD 2020... 5

Supplementary Table 3. Global and regional mean (95% UI) sugar-sweetened beverage intakes (8 oz serving/week) in adults (20+ years) by age, sex, education, and area of residence across 185 countries in 2020............................ 6

Supplementary Table 4. National mean (95% UI) sugar-sweetened beverage intakes (8 oz servings/week) in adults (20+ years) by sex, education, and area of residence in the 30 most populous countries in 2020.* 7

Supplementary Figure 1. Deaths of T2D and CVD per 1 million adults attributable to intake of SSBs among adults (20+ years) in 184 countries in 2020.. 8

Supplementary Figure 2. DALYs of T2D and CVD per 1 million adults attributable to intake of SSBs among adults (20+ years) in 184 countries in 2020.. 9

Supplementary Figure 3. Deaths of T2D attributable to SSBs intake by key sociodemographic factors at the global level and by world region in 2020.. 10

Supplementary Figure 4. Deaths of CVD attributable to SSBs intake by key sociodemographic factors at the global level and by world region in 2020.. 11

Supplementary Figure 5. DALYs of T2D attributable to SSBs intake by key sociodemographic factors at the global level and by world region in 2020.. 12

Supplementary Figure 6. DALYs of CVD attributable to SSBs intake by key sociodemographic factors at the global level and by world region in 2020.. 13

Supplementary Table 5. T2D burdens attributable to SSBs as per 1 million adults and proportional in 2020........... 14

Supplementary Table 6. CVD burdens attributable to SSBs as per 1 million adults and proportional in 2020.¥ 15

Supplementary Figure 7. Proportional deaths of T2D and CVD attributable to SSBs intake among adults (20+ years) jointly stratified by world region and age in 2020. 16

Supplementary Figure 8. Proportional DALYs of T2D and CVD attributable to SSBs intake among adults (20+ years) jointly stratified by world region and age in 2020. 17

Supplementary Figure 9. Proportional deaths of T2D and CVD attributable to intake of SSBs among adults (20+ years) jointly stratified by world region, area of residence, and education level in 2020....................................... 18

Supplementary Figure 10. Proportional DALYs of T2D and CVD attributable to intake of SSBs among adults (20+ years) jointly stratified by world region, area of residence, and education level in 2020....................................... 19

Supplementary Figure 12. Change in deaths per 1 million adults of T2D and CVD attributable to intake of SSBs among adults (20+ years) from 1990 to 2020 among the 30 most populous countries. 21

Supplementary Figure 13. Change in DALYs per 1 million adults of T2D and CVD attributable to intake of SSBs among adults (20+ years) from 1990 to 2020 among the 30 most populous countries. 22

Supplementary Figure 14. National correlation of proportional SSB-attributable T2D deaths and SDI at the national level in 1990 and 2020.. 23

Supplementary Figure 15. National correlation of proportional SSB-attributable CVD deaths and SDI at the national level in 1990 and 2020.. 24

Supplementary Figure 16. National correlation of proportional SSB-attributable T2D DALYs and SDI at the national level in 1990 and 2020.. 25

Supplementary Figure 17. National correlation of proportional SSB-attributable CVD DALYs and SDI at the national level in 1990 and 2020. 26

Supplementary Table 7. Primary data inputs and associated data sources for comparative risk assessment analysis. 27

Supplementary Table 8. Etiologic effects of SSBs and BMI on ischemic heart disease, ischemic stroke, T2D, and BMI. 28

Supplementary Table 9. Grading of Evidence of the Association of SSBs with ischemic heart disease, ischemic stroke, T2D , and weight gain.* 29

Supplementary Table 10. Age at event calculation for the etiologic effect of SSBs on ischemic heart disease and ischemic stroke. 30

Supplementary Table 11. Age at event calculation for the etiologic effect of SSBs on T2D. 31

Supplementary Table 12. Age at event calculation for the etiologic effect of BMI on ischemic stroke and T2D. 32

Supplementary Table 13. Effect estimates of the association between education level and area of residence with T2D and CVD used for the stratification of the GBD disease estimates*. 33

Supplementary Table 14. T2D burden disaggregation for a mock, a single national level age-sex T2D incidence estimate (low-income country) into six education level, urbanicity stratified estimates 34

Supplementary Figure 18. Pathway for estimating the direct and BMI-mediated CVD and T2D burden attributable to intake of SSBs and list of CRA analysis inputs. 35

Supplementary Table 1. Characteristics of global data sources of sugar-sweetened beverage intakes.

	World	Central or Eastern Europe and Central Asia[†]	High-Income Countries	Latin America and Caribbean	Middle East and North Africa	South Asia[†]	Southeast and East Asia	Sub-Saharan Africa
Number of surveys (% nationally or subnationally representative)	450 (94.1%)[*]	89 (100%)	210 (97.6%)	39 (92.3%)	39 (84.6%)	9 (77.8%)	42 (85.7%)	22 (81.8%)
Number of countries represented	118	18	24	22	16	5	18	15
Time period of surveys	1980-2018	1985-2015	1980-2018	1993-2015	1990-2015	1993-2012	1990-2017	1995-2016
Total sample size of surveyed subjects[§]	2,941,704	390,246	1,289,722	285,156	225,687	81,630	605,044	49,033
By sex								
Females	1,292,940 (44.2%)	154,851 (39.7%)	451,638 (35.0%)	170,074 (59.6%)	119,603 (53.0%)	41,930 (51.4%)	329,007 (54.4%)	25,837 (52.7%)
Males	1,633,578 (55.8%)	235,395 (60.3%)	838,084 (65.0%)	115,082 (40.4%)	106,084 (47.0%)	39,700 (48.6%)	276,037 (45.6%)	23,196(47.3%)
By area of residence								
Urban	2,046,673 (69.9%)	246,967 (63.3%)	974,386 (75.6%)	208,591 (73.1%)	152,482 (67.6%)	23,804 (29.2%)	426,267 (70.5%)	14,176 (28.9%)
Rural	879,845 (30.1%)	143,279 (36.7%)	315,336 (24.4%)	76,565 (26.9%)	73,205 (32.4%)	57,826 (70.8%)	178,777 (29.5%)	34,857 (71.1%)
By education level								
Low education	467,408 (16.0%)	20,509 (5.3%)	130,957 (10.2%)	109,074 (38.3%)	71,817 (31.8%)	35,838 (43.9%)	70,807 (11.7%)	28,405 (57.9%)
Medium education	1,099,716 (37.6%)	131,621 (33.7%)	530,353 (41.1%)	109,359 (38.4%)	68,382 (30.3%)	26,462 (32.4%)	219,211 (36.2%)	14,329 (29.2%)
High education	1,359,394 (46.5%)	238,116 (61.0%)	628,412 (48.7%)	66,723 (23.4%)	85,488 (37.9%)	19,330 (23.7%)	315,026 (52.1%)	6,299 (12.8%)
By adult/youth								
Adults (18+ years)	1,549,750 (53.0%)	119,355 (30.6%)	652,460 (50.6%)	91,677 (32.1%)	110,400 (48.9%)	40,819 (50.0%)	530,735 (87.7%)	4,304 (8.8%)
Children/adolescent (<18 years)	1,376,768 (46.8%)	270,891 (69.4%)	637,262 (49.4%)	193,479 (67.9%)	115,287 (51.1%)	40,811 (50.0%)	74,309 (12.3%)	44,729 (91.2%)
World population represented in 2020[f]	6,716,876 (86.8%)	309,339 (74.1%)	824,639 (100.0%)	562,591 (86.6%)	491,911 (89.9%)	1,787,540 (96.3%)	2,255,195 (97.2%)	485,660 (43.3%)
Number of surveys by characteristic[¥]								
By adult/youth								
Adults	191 (42.4%)	29 (32.6%)	93 (44.3%)	12 (30.1%)	12 (30.8%)	4 (44.4%)	31 (73.8%)	10 (45.5%)
Children/adolescents	323 (71.8%)	65 (73.0%)	143 (68.1%)	35 (89.7%)	30 (76.9%)	7 (77.8%)	27 (64.3%)	16 (72.7%)
By area of residence								
Both urban and rural	278 (61.8%)	62 (69.7%)	115 (54.8%)	28 (71.8%)	28 (71.8%)	4 (44.4%)	27 (64.3%)	14 (63.3%)
Only urban	13 (2.9%)	0 (0%)	1 (0.5%)	3 (7.7%)	6 (15.4%)	1 (11.1%)	0 (0%)	2 (9.0%)
Only rural	7 (1.6%)	0 (0%)	0 (0%)	0 (0%)	0 (0%)	3 (33.3%)	1 (2.4%)	3 (13.6%)
Not reported	152 (33.8%)	27 (30.3%)	94 (44.8%)	8 (20.5%)	5 (12.8%)	1 (11.1%)	14 (33.3%)	3 (13.6%)
By dietary assessment method								
FFQ	272 (60.4%)	58 (65.2%)	115 (54.8%)	26 (66.7%)	27 (69.2%)	5 (55.6%)	29 (69.0%)	12 (54.5%)
24hr recall	109 (24.2%)	14 (15.7%)	58 (27.7%)	8 (20.4%)	7 (17.9%)	2 (22.2%)	12 (28.5%)	8 (36.3%)
Household budget survey	54 (12.0%)	17 (19.1%)	37 (17.6%)	0 (0%)	0 (0%)	0 (0%)	0 (0%)	0 (0%)
DHS	15 (3.3%)	0 (0%)	0 (0%)	5 (12.8%)	5 (12.8%)	2 (22.2%)	1 (2.4%)	2 (9.1%)

[*]Of the 450 surveys reporting data on sugar-sweetened beverages, 46.7% of surveys were in High-Income Countries, followed by Central/Eastern Europe and Central Asia (19.8%), Southeast and East Asia (9.3%), Latin American and the Caribbean (8.7%), Middle East and Northern Africa (8.7%), Sub-Saharan Africa (4.9%), and South Asia (2.0%).

[§] The total of subjects sampled can correspond to multiple years, thus in some cases this number is larger than the world population represented in 2020 shown in a few rows below.

[f] Population expressed in millions. Total population of the countries with sugar-sweetened beverage intake data and the percentage in relation to the total population of all countries within that region.

[¥] The values represent the absolute number of surveys and the percentage in relation to the total number of surveys within that region.

[†] In prior GDD reports, the region Central or Eastern Europe and Central Asia was referred as Former Soviet Union, and Southeast and East Asia was referred as Asia.

Supplementary Table 2. Countries by world region (superregion) in GDD 2020.

Central or Eastern Europe and Central Asia[§]		High-Income Countries	Latin America and Caribbean		Middle East and North Africa	South Asia[§]	Southeast and East Asia	Sub-Saharan Africa	
n=29		*n=24*	*n=32*		*n=20*	*n=8*	*n=24*	*n=48*	
Albania	Slovenia	Australia	Antigua and Barbuda	Panama	Algeria	Afghanistan	Brunei	Angola	Liberia
Armenia	Tajikistan	Austria	Argentina	Paraguay	Bahrain	Bangladesh	Cambodia	Benin	Madagascar
Azerbaijan	Turkmenistan	Belgium	Bahamas, The	Peru	Egypt, Arab Rep.	Bhutan	China	Botswana	Malawi
Belarus	Ukraine	Canada	Barbados	St. Lucia	Iran, Islamic Rep.	India	Fiji	Burkina Faso	Mali
Bosnia and Herzegovina	Uzbekistan	Cyprus	Belize	St. Vincent and the Grenadines	Iraq	Maldives	Indonesia	Burundi	Mauritania
Bulgaria		Denmark	Bolivia	Suriname	Israel	Nepal	Japan	Cameroon	Mauritius
Croatia		Finland	Brazil	Trinidad and Tobago	Jordan	Pakistan	Kiribati	Cape Verde	Mozambique
Czech Republic		France	Chile	Uruguay	Kuwait	Sri Lanka	Korea, Rep.	Central African Republic	Namibia
Estonia		Germany	Colombia	Venezuela	Lebanon		Lao PDR	Chad	Niger
Georgia		Greece	Costa Rica		Libya		Malaysia	Comoros	Nigeria
Hungary		Iceland	Cuba		Morocco		Marshall Islands	Congo, Dem. Rep.	Rwanda
Kazakhstan		Ireland	Dominica		Oman		Micronesia, Fed. Sts.	Congo, Rep.	Sao Tome and Principe
Kyrgyz Republic		Italy	Dominican Republic		Palestine		Myanmar	Cote d'Ivoire	Senegal
Latvia		Luxembourg	Ecuador		Qatar		Papua New Guinea	Djibouti	Seychelles
Lithuania		Malta	El Salvador		Saudi Arabia		Philippines	Equatorial Guinea	Sierra Leone
Macedonia, FYR		Netherlands	Grenada		Syrian Arab Republic		Samoa	Eritrea	South Africa
Moldova		New Zealand	Guatemala		Tunisia		Singapore	Ethiopia (excludes Eritrea)	South Sudan*
Mongolia		Norway	Guyana		Turkey		Solomon Islands	Gabon	Sudan
Montenegro		Portugal	Haiti		United Arab Emirates		Taiwan	Gambia, The	Swaziland
Poland		Spain	Honduras		Yemen, Rep.		Thailand	Ghana	Tanzania
Romania		Sweden	Jamaica				Timor-Leste	Guinea	Togo
Russian Federation		Switzerland	Mexico				Tonga	Guinea-Bissau	Uganda
Serbia		United Kingdom	Nicaragua				Vanuatu	Kenya	Zambia
Slovak Republic		United States					Vietnam	Lesotho	Zimbabwe

[§] In prior GDD reports, the region Central/ Eastern Europe and Central Asia was referred as Former Soviet Union, and Southeast and East Asia was referred to as Asia.
* Excluded from this analysis because it lacked of disease estimates from the Global Burden of Disease

Supplementary Table 3. Global and regional mean (95% UI) sugar-sweetened beverage intakes (8 oz serving/week) in adults (20+ years) by age, sex, education, and area of residence across 185 countries in 2020.

	World	Centr/Eastern Europe and Centr Asia†	High-Income Countries	Latin Amer/Caribbean	Mid. East/North Africa	South Asia	Southeast and East Asia†	Sub-Saharan Africa
Overall	2.6 (2.4-2.8)	2.1 (1.8-2.4)	3.6 (3.4-3.7)	7.3 (6.7-8.1)	4.1 (3.5-4.9)	0.7 (0.5-1.1)	0.9 (0.8-1.0)	6.5 (5.2-8.1)
Female	2.5 (2.3-2.7)	1.9 (1.6-2.2)	3.2 (3.0-3.4)	7.0 (6.3-7.8)	4.1 (3.4-4.9)	0.7 (0.5-1.1)	0.9 (0.8-1.0)	6.3 (5.0-7.9)
Male	2.7 (2.5-2.9)	2.3 (2.0-2.7)	3.9 (3.8-4.2)	7.7 (7.0-8.6)	4.2 (3.5-5.0)	0.8 (0.5-1.1)	0.9 (0.8-1.1)	6.6 (5.3-8.3)
20-24 years	4.2 (3.9-4.6)	4.5 (3.9-5.3)	6.5 (6.2-6.8)	10.5 (9.6-11.5)	7.0 (5.8-8.3)	1.0 (0.7-1.5)	2.1 (1.9-2.5)	6.8 (5.5-8.6)
25-29 years	3.8 (3.5-4.2)	3.6 (3.0-4.2)	6.0 (5.7-6.3)	9.7 (8.8-10.8)	5.7 (4.7-6.9)	0.9 (0.6-1.4)	1.6 (1.4-1.9)	7.1 (5.7-9.1)
30-34 years	3.2 (2.9-3.5)	2.8 (2.4-3.4)	5.3 (5.1-5.6)	8.7 (7.8-9.8)	4.5 (3.8-5.5)	0.8 (0.6-1.3)	1.1 (1.0-1.3)	7.1 (5.6-9.2)
35-39 years	2.9 (2.7-3.2)	2.3 (1.9-2.7)	4.6 (4.4-4.9)	7.8 (7.0-8.8)	3.8 (3.2-4.7)	0.7 (0.5-1.2)	1.0 (0.9-1.2)	6.9 (5.3-9.0)
40-44 years	2.6 (2.3-2.8)	2.0 (1.6-2.3)	4.0 (3.8-4.2)	7.1 (6.3-8.0)	3.4 (2.8-4.2)	0.7 (0.4-1.1)	0.8 (0.7-1.0)	6.5 (5.0-8.5)
45-49 years	2.1 (2.0-2.3)	1.7 (1.4-2.0)	3.4 (3.2-3.6)	6.5 (5.8-7.3)	3.1 (2.5-3.8)	0.6 (0.4-1.0)	0.6 (0.5-0.7)	6.0 (4.6-7.9)
50-54 years	1.9 (1.7-2.0)	1.6 (1.3-1.8)	3.0 (2.8-3.1)	6.0 (5.3-6.7)	2.9 (2.4-3.5)	0.6 (0.4-0.9)	0.5 (0.4-0.6)	5.6 (4.3-7.3)
55-59 years	1.7 (1.6-1.9)	1.4 (1.2-1.7)	2.6 (2.5-2.7)	5.5 (4.9-6.2)	2.7 (2.3-3.3)	0.5 (0.4-0.8)	0.5 (0.4-0.6)	5.2 (3.9-6.9)
60-64 years	1.6 (1.5-1.7)	1.3 (1.1-1.6)	2.3 (2.2-2.4)	5.0 (4.4-5.8)	2.7 (2.2-3.2)	0.5 (0.3-0.8)	0.4 (0.4-0.5)	4.8 (3.5-6.6)
65-69 years	1.4 (1.3-1.5)	1.2 (1.0-1.4)	2.0 (1.9-2.1)	4.7 (4.1-5.4)	2.6 (2.1-3.2)	0.5 (0.3-0.7)	0.4 (0.3-0.4)	4.4 (3.1-6.3)
70-74 years	1.4 (1.2-1.5)	1.1 (0.9-1.4)	1.7 (1.6-1.9)	4.4 (3.8-5.2)	2.5 (2.0-3.1)	0.5 (0.3-0.8)	0.4 (0.3-0.5)	4.1 (2.7-6.0)
75-79 years	1.3 (1.2-1.4)	1.1 (0.8-1.4)	1.5 (1.4-1.7)	4.2 (3.5-5.0)	2.5 (2.0-3.2)	0.5 (0.3-0.8)	0.4 (0.3-0.5)	3.8 (2.5-5.9)
80-84 years	1.2 (1.1-1.3)	1.0 (0.7-1.3)	1.4 (1.3-1.5)	3.9 (3.2-4.8)	2.4 (1.9-3.2)	0.4 (0.2-0.8)	0.4 (0.3-0.4)	3.6 (2.2-5.8)
85-89 years	1.1 (1.0-1.2)	0.9 (0.7-1.2)	1.2 (1.1-1.3)	3.7 (3.0-4.6)	2.3 (1.8-3.0)	0.4 (0.2-0.7)	0.4 (0.3-0.5)	3.6 (2.1-6.0)
90-94 years	1.1 (1.0-1.2)	0.8 (0.6-1.1)	1.1 (1.0-1.2)	3.6 (2.8-4.5)	2.4 (1.7-3.1)	0.4 (0.2-0.7)	0.4 (0.4-0.5)	3.3 (1.9-5.8)
95+ years	1.1 (1.0-1.2)	0.7 (0.5-1.1)	1.0 (0.9-1.1)	3.4 (2.7-4.5)	2.5 (1.8-3.4)	0.2 (0.1-0.5)	0.4 (0.4-0.5)	3.3 (1.8-6.1)
0-6	2.4 (2.2-2.8)	2.1 (1.7-2.8)	3.5 (3.3-3.8)	6.7 (6.0-7.6)	4.6 (3.7-5.7)	0.6 (0.4-0.9)	0.9 (0.7-1.1)	5.2 (3.9-6.8)
6-<12	2.6 (2.4-2.8)	2.3 (1.9-2.7)	3.5 (3.3-3.8)	7.7 (6.9-8.7)	3.7 (3.1-4.5)	0.6 (0.4-1.0)	0.9 (0.8-1.0)	8.9 (7.0-11.2)
≥12	2.9 (2.8-3.2)	2.0 (1.7-2.3)	3.6 (3.4-3.8)	8.0 (6.9-9.2)	3.3 (2.9-4.0)	2.6 (1.8-4.2)	1.0 (0.9-1.1)	9.9 (7.5-13.0)
Rural	2.0 (1.8-2.2)	2.3 (2.0-2.8)	3.5 (3.4-3.7)	7.3 (6.4-8.2)	4.7 (3.7-6.1)	0.3 (0.3-0.5)	0.9 (0.8-1.1)	5.4 (4.3-6.7)
Urban	3.0 (2.8-3.3)	2.0 (1.7-2.3)	3.6 (3.4-3.8)	7.4 (6.7-8.1)	3.8 (3.3-4.5)	1.5 (0.9-2.4)	0.9 (0.8-1.0)	8.0 (6.2-10.3)

Data are the mean intakes (95% uncertainty interval) in 8 oz servings per week. All intakes are reported adjusted to 2,000 kcal/d for ages 20 to 74 years, and 1,700 kcal/d for ages 75+ years. Data are based on a Bayesian model that incorporated up to 451 individual-level dietary surveys, and additional survey-level and country-level covariates, to estimate dietary consumption levels. Total sugar-sweetened beverages (SSBs) intake was defined as any beverage with added sugars having ≥50 kcal per 8 oz serving, including commercial or homemade beverages, soft drinks, energy drinks, fruit drinks, punch, lemonade, and aguas frescas. This definition excludes 100% fruit and vegetable juices and non-caloric artificially sweetened drinks. Standardized serving size used for this analysis: 8 oz serving = 248 grams. Education level "Low" 0 to 6 years of education; "Medium" >6 years to 12 years of education; and "High" >12 years of education. Source data are provided as Source Data file 2.

† In prior GDD reports, the region Central or Eastern Europe and Central Asia was referred as Former Soviet Union, and Southeast and East Asia was referred as Asia.

Oz, ounces; SSBs, sugar-sweetened beverages; UIs, uncertainty intervals

Supplementary Table 4. National mean (95% UI) sugar-sweetened beverage intakes (8 oz servings/week) in adults (20+ years) by sex, education, and area of residence in the 30 most populous countries in 2020.*

		Sex		Education level			Area of residence	
Country	Overall	Females	Males	0-6 years	6-<12 years	≥12 years	Rural	Urban
China	0.2 (0.2-0.3)	0.2 (0.2-0.3)	0.2 (0.2-0.3)	0.2 (0.2-0.2)	0.2 (0.2-0.3)	0.2 (0.2-0.3)	0.2 (0.2-0.3)	0.2 (0.2-0.3)
India	0.2 (0.1-0.3)	0.1 (0.1-0.2)	0.2 (0.1-0.3)	0.1 (0.1-0.2)	0.2 (0.1-0.4)	0.3 (0.2-0.5)	0.1 (0.1-0.1)	0.4 (0.2-0.6)
United States	4.9 (4.7-5.2)	4.4 (4.1-4.7)	5.5 (5.1-5.8)	6.3 (5.9-6.9)	6.2 (5.8-6.7)	4.6 (4.3-4.9)	5.1 (4.9-5.4)	4.9 (4.6-5.2)
Indonesia	0.8 (0.6-1.1)	0.8 (0.6-1.1)	0.9 (0.7-1.2)	0.8 (0.6-1.0)	0.9 (0.7-1.2)	0.9 (0.7-1.2)	0.8 (0.6-1.1)	0.9 (0.6-1.1)
Brazil	4.1 (3.6-4.7)	3.9 (3.3-4.5)	4.4 (3.7-5.1)	4.0 (3.4-4.6)	4.3 (3.7-4.9)	4.3 (3.6-5.1)	3.7 (3.2-4.3)	4.2 (3.7-4.7)
Pakistan	4.2 (2.6-7.1)	4.2 (2.5-7.2)	4.2 (2.6-7.2)	3.1 (1.8-5.4)	5.7 (3.4-10.0)	7.6 (4.4-13.7)	1.8 (1.1-2.9)	8.3 (4.9-14.6)
Russia	2.1 (1.7-2.5)	1.9 (1.5-2.3)	2.3 (1.9-2.9)	2.0 (1.5-2.7)	2.2 (1.8-2.8)	2.0 (1.7-2.5)	2.2 (1.8-2.7)	2.0 (1.7-2.5)
Japan	1.9 (1.7-2.2)	1.7 (1.4-2.0)	2.1 (1.8-2.5)	1.6 (1.4-1.9)	1.9 (1.7-2.2)	1.9 (1.7-2.2)	1.8 (1.6-2.1)	1.9 (1.7-2.2)
Bangladesh	0.2 (0.2-0.4)	0.2 (0.2-0.4)	0.3 (0.2-0.4)	0.2 (0.1-0.3)	0.3 (0.2-0.5)	0.4 (0.3-0.7)	0.1 (0.1-0.1)	0.5 (0.3-0.8)
Nigeria	4.6 (3.0-6.9)	4.4 (2.8-7.0)	4.7 (3.0-7.3)	3.8 (2.4-6.1)	6.4 (4.1-10.1)	7.2 (4.4-11.7)	3.5 (2.3-5.5)	5.5 (3.5-8.7)
Mexico	8.5 (7.8-9.4)	7.9 (7.1-9.0)	9.2 (8.2-10.3)	8.2 (7.3-9.2)	8.7 (7.8-9.8)	8.8 (7.6-10.3)	7.7 (6.9-8.6)	8.7 (7.9-9.7)
Germany	2.7 (2.5-3.0)	2.3 (2.0-2.6)	3.2 (2.8-3.6)	3.1 (2.7-3.5)	3.0 (2.7-3.4)	2.2 (2.0-2.5)	2.8 (2.5-3.2)	2.7 (2.4-3.0)
Vietnam	1.7 (1.4-2.2)	1.7 (1.3-2.2)	1.8 (1.4-2.4)	1.6 (1.2-2.0)	1.9 (1.5-2.4)	1.9 (1.5-2.4)	1.7 (1.3-2.2)	1.8 (1.4-2.3)
Philippines	3.2 (2.8-3.7)	3.4 (2.9-3.9)	3.0 (2.4-3.7)	2.8 (2.4-3.3)	3.4 (2.9-3.9)	3.3 (2.9-3.9)	3.2 (2.7-3.7)	3.3 (2.9-3.8)
Egypt	2.7 (2.2-3.3)	2.9 (2.4-3.6)	2.4 (1.9-3.1)	2.7 (2.2-3.3)	2.9 (2.3-3.6)	2.4 (1.9-3.0)	2.8 (2.3-3.5)	2.5 (2.0-3.0)
Iran	2.7 (2.4-3.0)	2.6 (2.3-3.0)	2.8 (2.4-3.2)	2.7 (2.4-3.1)	2.9 (2.6-3.4)	2.4 (2.1-2.8)	3.0 (2.6-3.5)	2.6 (2.3-2.9)
Turkey	0.3 (0.1-0.7)	0.3 (0.1-0.7)	0.3 (0.1-0.8)	0.3 (0.1-0.7)	0.3 (0.2-0.8)	0.3 (0.1-0.6)	0.4 (0.2-0.8)	0.3 (0.1-0.7)
Thailand	4.1 (2.0-8.4)	3.9 (1.8-8.2)	4.3 (2.1-8.8)	3.8 (1.9-7.8)	4.6 (2.2-9.5)	4.6 (2.2-9.4)	4.0 (2.0-8.2)	4.2 (2.1-8.7)
Ethiopia	6.9 (5.5-8.7)	6.7 (5.2-8.7)	7.1 (5.5-9.3)	5.7 (4.4-7.3)	9.5 (7.3-12.4)	10.6 (7.7-14.9)	6.1 (4.8-7.9)	9.7 (7.5-12.7)
United Kingdom	4.1 (3.7-4.5)	4.0 (3.6-4.5)	4.2 (3.8-4.8)	4.4 (4.0-5.0)	4.4 (4.0-4.8)	3.2 (2.9-3.6)	4.3 (3.9-4.8)	4.1 (3.7-4.5)
Italy	1.5 (1.4-1.7)	1.4 (1.2-1.6)	1.7 (1.5-2.0)	1.7 (1.5-1.9)	1.6 (1.5-1.8)	1.2 (1.1-1.4)	1.6 (1.4-1.8)	1.5 (1.4-1.7)
France	2.4 (2.2-2.6)	2.0 (1.7-2.2)	2.8 (2.5-3.2)	2.6 (2.4-3.0)	2.6 (2.3-2.9)	1.9 (1.7-2.1)	2.5 (2.2-2.8)	2.4 (2.1-2.6)
Korea	0.9 (0.8-0.9)	0.7 (0.6-0.8)	1.1 (1.0-1.1)	0.7 (0.7-0.8)	0.9 (0.8-1.0)	0.9 (0.8-1.0)	0.8 (0.8-0.9)	0.9 (0.8-1.0)
Spain	3.0 (2.5-3.5)	2.8 (2.2-3.4)	3.1 (2.6-3.8)	3.1 (2.6-3.8)	3.1 (2.6-3.7)	2.3 (1.9-2.7)	3.1 (2.5-3.7)	2.9 (2.5-3.5)
DR Congo	1.8 (0.7-5.0)	1.7 (0.6-4.7)	1.9 (0.7-5.3)	1.5 (0.5-4.2)	2.6 (0.9-6.9)	2.9 (1.0-7.6)	1.4 (0.5-3.9)	2.3 (0.8-6.3)
South Africa	9.6 (7.5-12.5)	9.1 (6.9-12.1)	10.1 (7.6-13.5)	6.6 (4.8-9.1)	11.1 (8.5-14.6)	12.4 (9.3-16.6)	6.8 (5.3-9.0)	10.9 (8.4-14.3)
Ukraine	1.3 (1.0-1.6)	1.2 (0.9-1.5)	1.4 (1.0-1.8)	1.2 (0.9-1.7)	1.3 (1.0-1.8)	1.2 (1.0-1.6)	1.3 (1.0-1.8)	1.2 (0.9-1.6)
Myanmar	3.2 (2.3-4.7)	3.0 (2.1-4.8)	3.4 (2.3-5.2)	3.1 (2.1-4.7)	3.6 (2.5-5.6)	3.6 (2.5-5.6)	3.2 (2.2-4.9)	3.3 (2.3-5.0)
Colombia	17.4 (13.2-22.7)	16.6 (12.3-22.6)	18.2 (13.4-24.2)	16.6 (12.4-22.0)	17.8 (13.5-23.5)	18.0 (13.5-23.9)	15.7 (11.7-20.6)	17.8 (13.5-23.2)
Argentina	5.6 (4.7-6.7)	5.0 (4.1-6.0)	6.3 (5.1-7.9)	5.3 (4.4-6.5)	5.7 (4.7-6.9)	5.7 (4.8-7.0)	5.0 (4.1-6.1)	5.7 (4.8-6.8)

*Data are mean intakes (95% uncertainty interval) in 8 oz servings per week. Standardized serving size used for this analysis: 8 oz serving = 248 grams. Countries are ordered top to bottom from most to least populous based on 2020 adult (20+ years) population data.

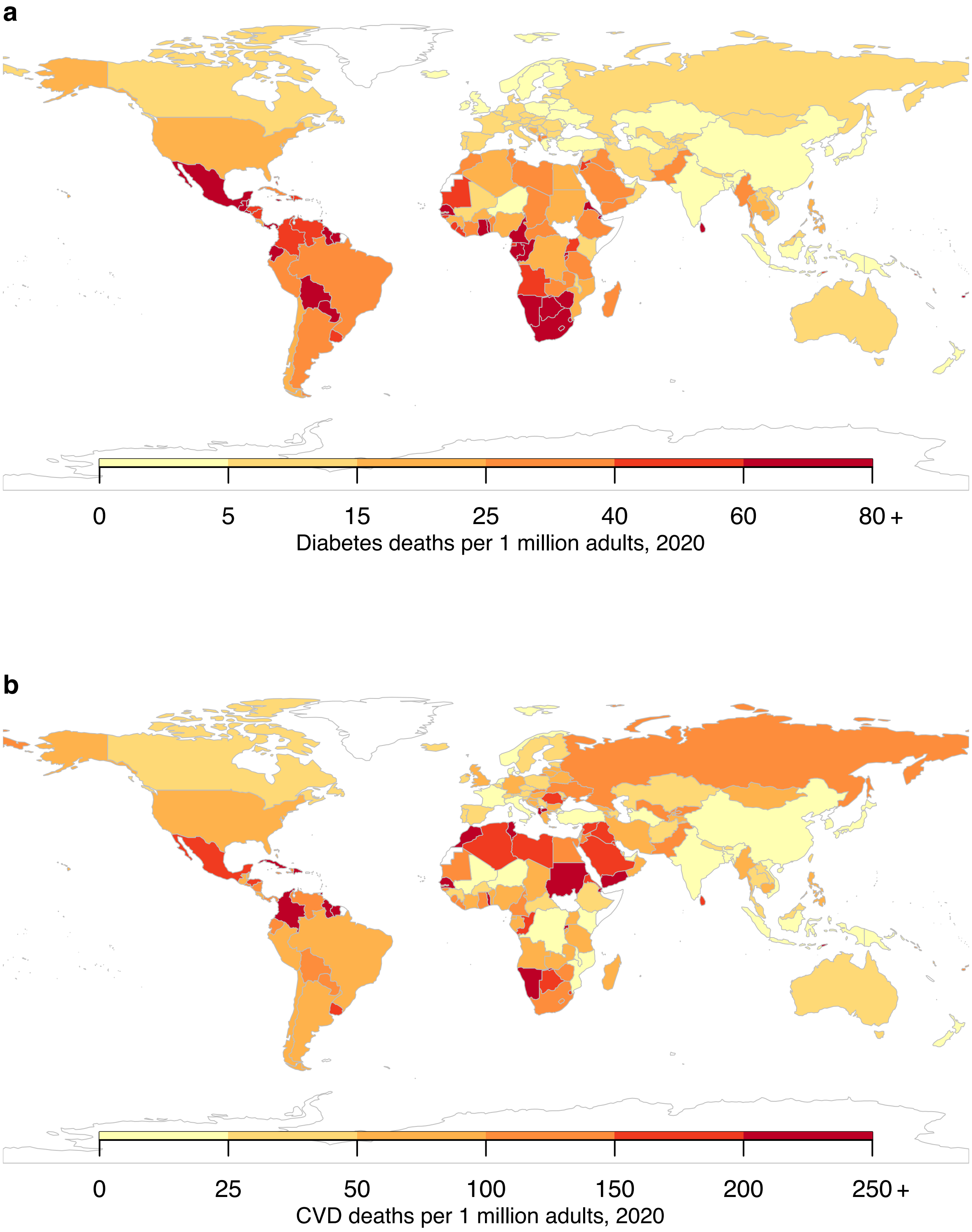

Supplementary Figure 1. **Deaths of T2D and CVD per 1 million adults attributable to intake of SSBs among adults (20+ years) in 184 countries in 2020. (a)** absolute SSB-attributable T2D deaths and **(b)** absolute SSB-attributable CVD deaths. The SSB-attributable absolute burden per 1 million adults was calculated by dividing the country absolute number of SSB-attributable cases by the country adult population (20+ years) in that same year and multiplying by 1 million. Values were truncated at 80 for the top panel (a) and at 250 for the bottom panel (b) to better reflect the absolute case distribution globally for T2D and CVD, respectively.

CVD, cardiovascular disease; SSBs, sugar sweetened beverages; T2D, type 2 diabetes.

a

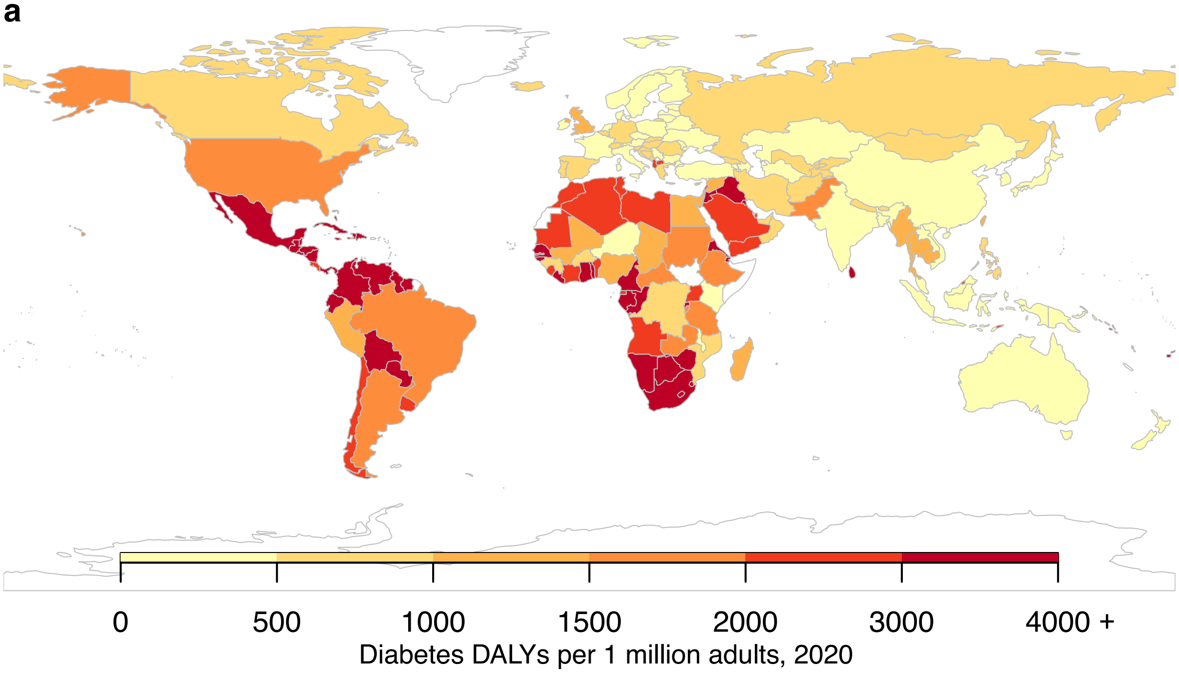

b

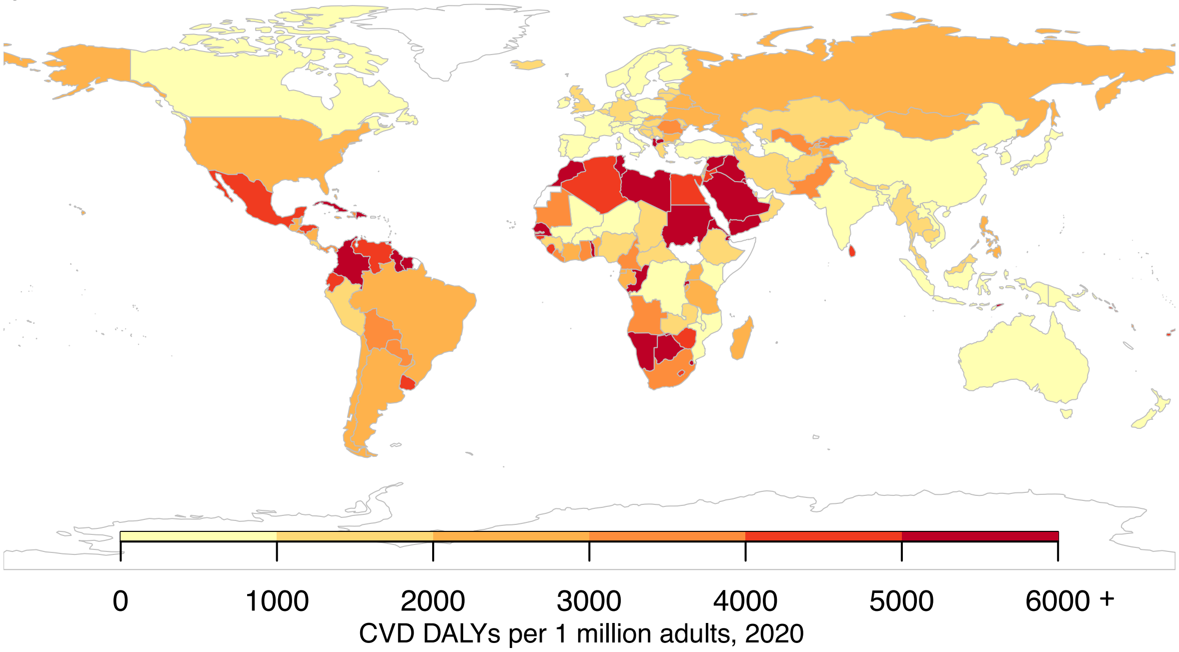

Supplementary Figure 2. **DALYs of T2D and CVD per 1 million adults attributable to intake of SSBs among adults (20+ years) in 184 countries in 2020. (a)** absolute SSB-attributable T2D DALYs and **(b)** absolute SSB-attributable CVD DALYs. The SSB-attributable absolute burden per 1 million adults was calculated by dividing the country absolute number of SSB-attributable cases by the country adult population (20+ years) in that same year. Values were truncated at 4,000 for the top panel (a) and 6,000 for the bottom panel (b) to better reflect the absolute case distribution globally for T2D and CVD, respectively.

CVD, cardiovascular disease; DALYs, disability-adjusted life years; SSBs, sugar sweetened beverages; T2D, type 2 diabetes.

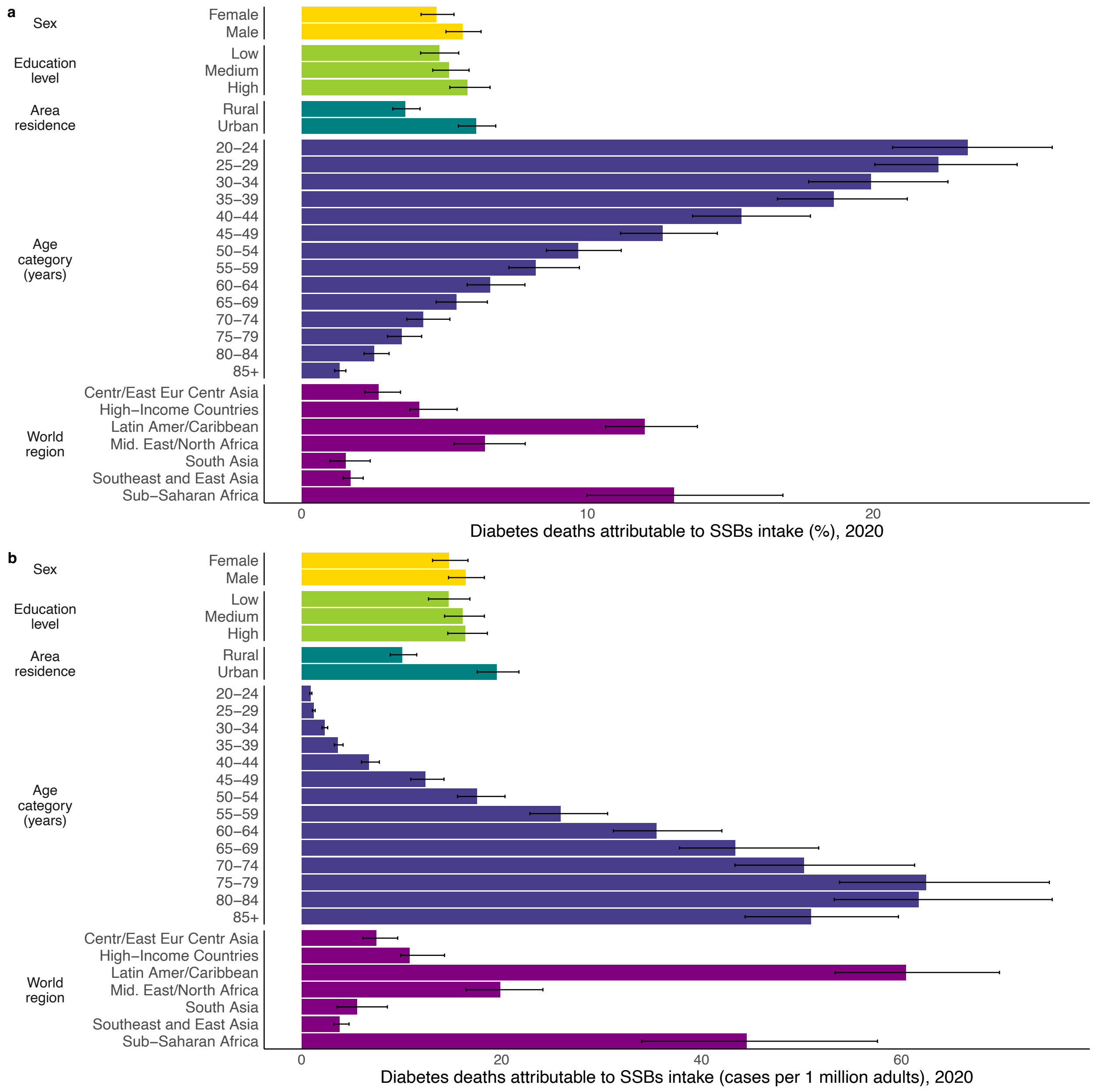

Supplementary Figure 3. **Deaths of T2D attributable to SSBs intake by key sociodemographic factors at the global level and by world region in 2020**. Bars represent the central estimate (median) of the proportional SSB-attributable T2D deaths in the top panel (a) and the absolute SSB-attributable T2D deaths per 1 million adults in the bottom panel (b). The error bars represent the 95% UI derived from the 2.5th and 97.5th percentiles of 1,000 multiway probabilistic Monte Carlo model simulations. The SSB-attributable absolute burden per 1 million adults was calculated by dividing the stratum absolute number of SSB-attributable cases by the stratum adult population (20+ years) in that same year and multiplying by 1 million. Colors differentiate sex (yellow), education (green light), area of residence (dark green), age category (dark purple), and world region (light purple). In prior GDD reports, the region Central/ Eastern Europe and Central Asia was referred as Former Soviet Union, and Southeast and East Asia was referred as Asia.

Centr/East Eur & Centr Asia, Central/Eastern Europe and Central Asia; GDD, Global Dietary Database; Latin Amer/Caribbean, Latin America/Caribbean; SSBs, sugar sweetened beverages; T2D, type 2 diabetes; UIs, uncertainty intervals

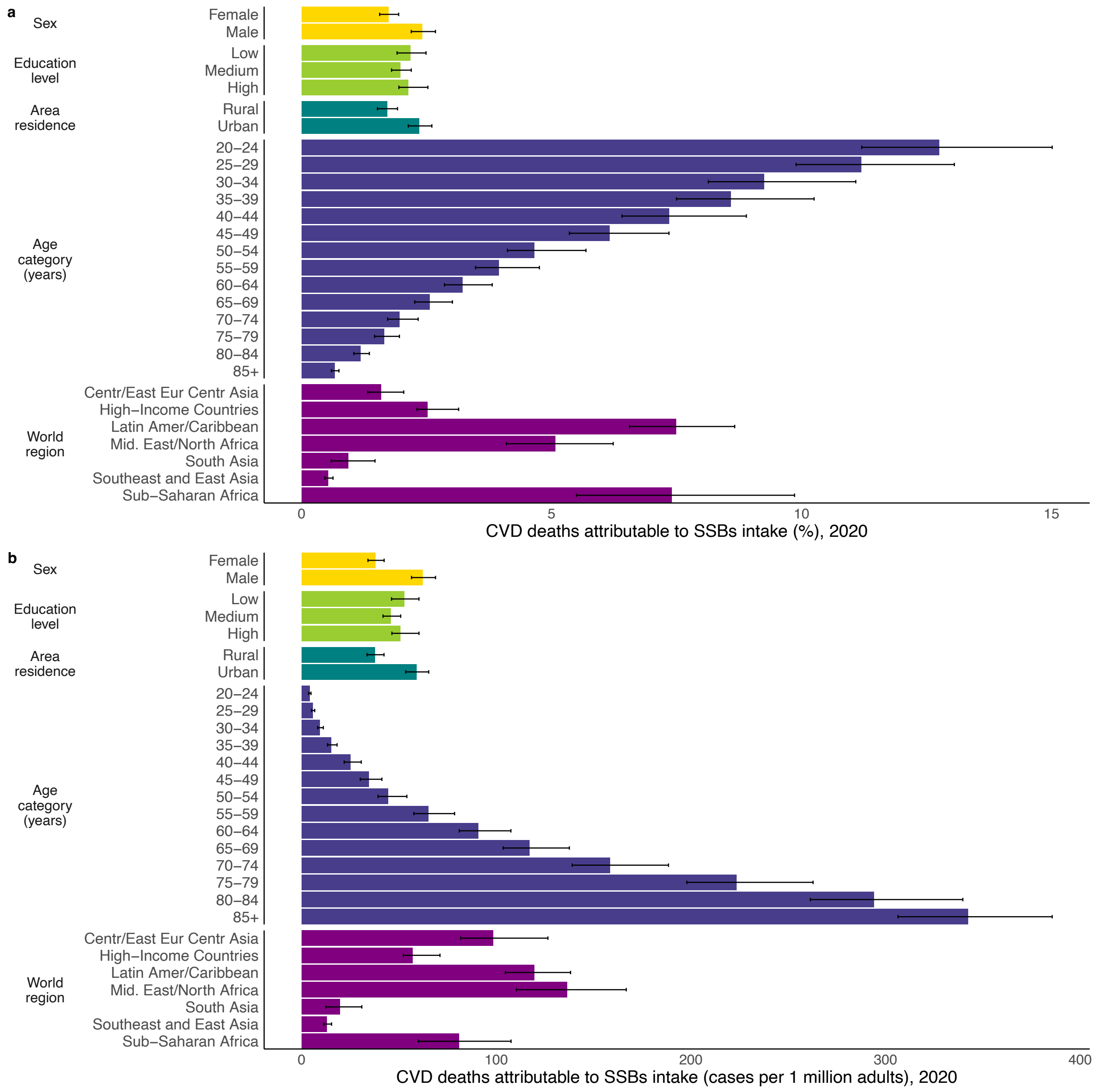

Supplementary Figure 4. **Deaths of CVD attributable to SSBs intake by key sociodemographic factors at the global level and by world region in 2020**. Bars represent the central estimate (median) of the proportional SSB-attributable CVD deaths in the top panel (a) and the absolute SSB-attributable CVD deaths per 1 million adults in the bottom panel (b). The error bars represent the 95% UI derived from the 2.5th and 97.5th percentiles of 1,000 multiway probabilistic Monte Carlo model simulations. The SSB-attributable absolute burden per 1 million adults was calculated by dividing the stratum absolute number of SSB-attributable cases by the stratum adult population (20+ years) in that same year and multiplying by 1 million. Colors differentiate sex (yellow), education (green light), area of residence (dark green), age category (dark purple), and world region (light purple). In prior GDD reports, the region Central/ Eastern Europe and Central Asia was referred as Former Soviet Union, and Southeast and East Asia was referred as Asia.

Centr/East Eur & Centr Asia, Central/Eastern Europe and Central Asia; CVD, cardiovascular disease; GDD, Global Dietary Database; Latin Amer/Caribbean, Latin America/Caribbean; SSBs, sugar sweetened beverages; UIs, uncertainty intervals.

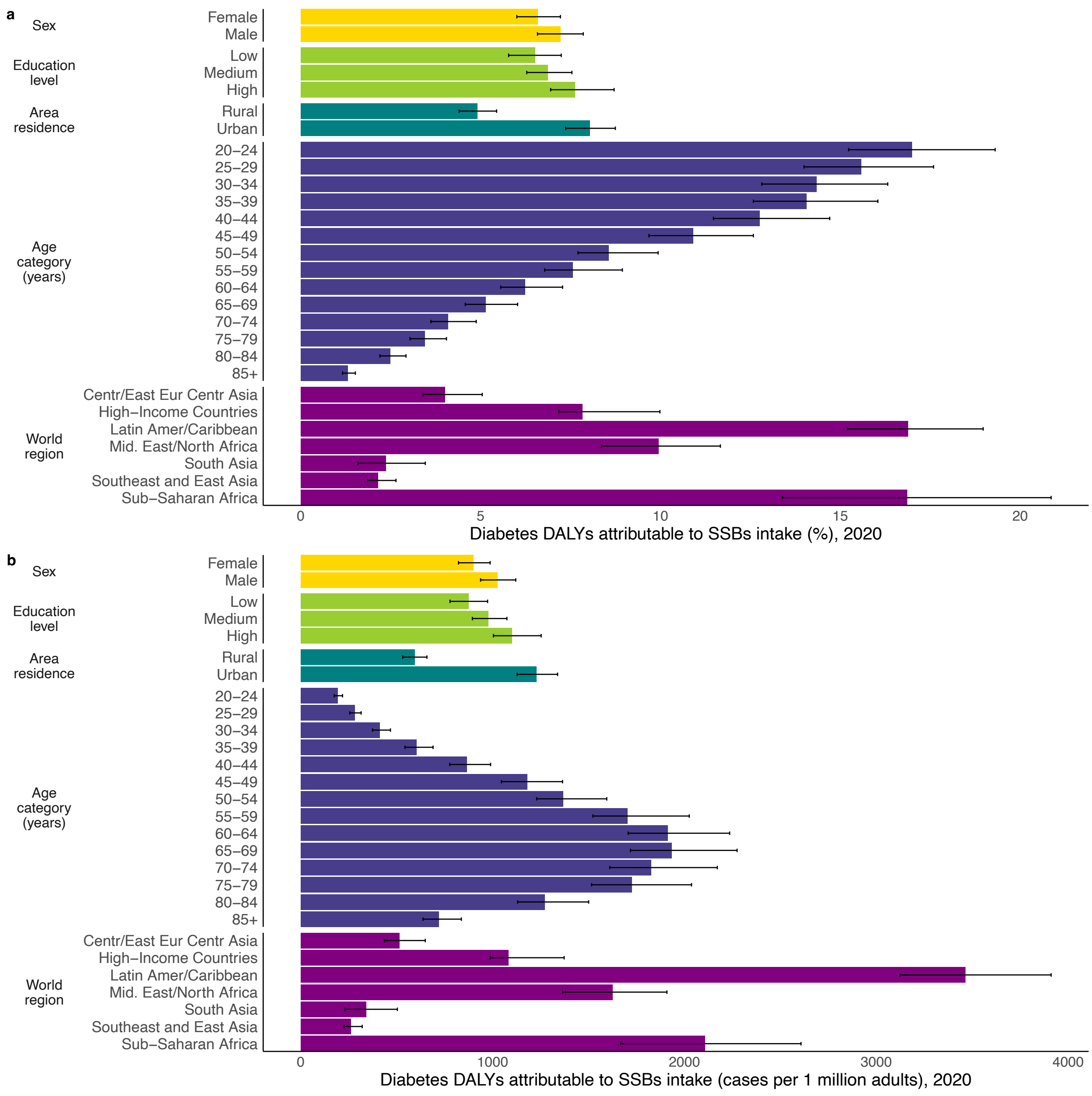

Supplementary Figure 5. **DALYs of T2D attributable to SSBs intake by key sociodemographic factors at the global level and by world region in 2020**. Bars represent the central estimate (median) of the proportional SSB-attributable T2D DALYs in the top panel (a) and the absolute SSB-attributable T2D DALYs per 1 million adults in the bottom panel (b). The error bars represent the 95% UI derived from the 2.5th and 97.5th percentiles of 1,000 multiway probabilistic Monte Carlo model simulations. The SSB-attributable absolute burden per 1 million adults was calculated by dividing the stratum absolute number of SSB-attributable cases by the stratum adult population (20+ years) in that same year and multiplying by 1 million. Colors differentiate sex (yellow), education (green light), area of residence (dark green), age category (dark purple), and world region (light purple). In prior GDD reports, the region Central/ Eastern Europe and Central Asia was referred as Former Soviet Union, and Southeast and East Asia was referred as Asia.

Centr/East Eur & Centr Asia, Central/Eastern Europe and Central Asia; DALYs, disability-adjusted life years; GDD, Global Dietary Database; Latin Amer/Caribbean, Latin America/Caribbean; SSBs, sugar sweetened beverages; T2D, type 2 diabetes; UIs, uncertainty intervals.

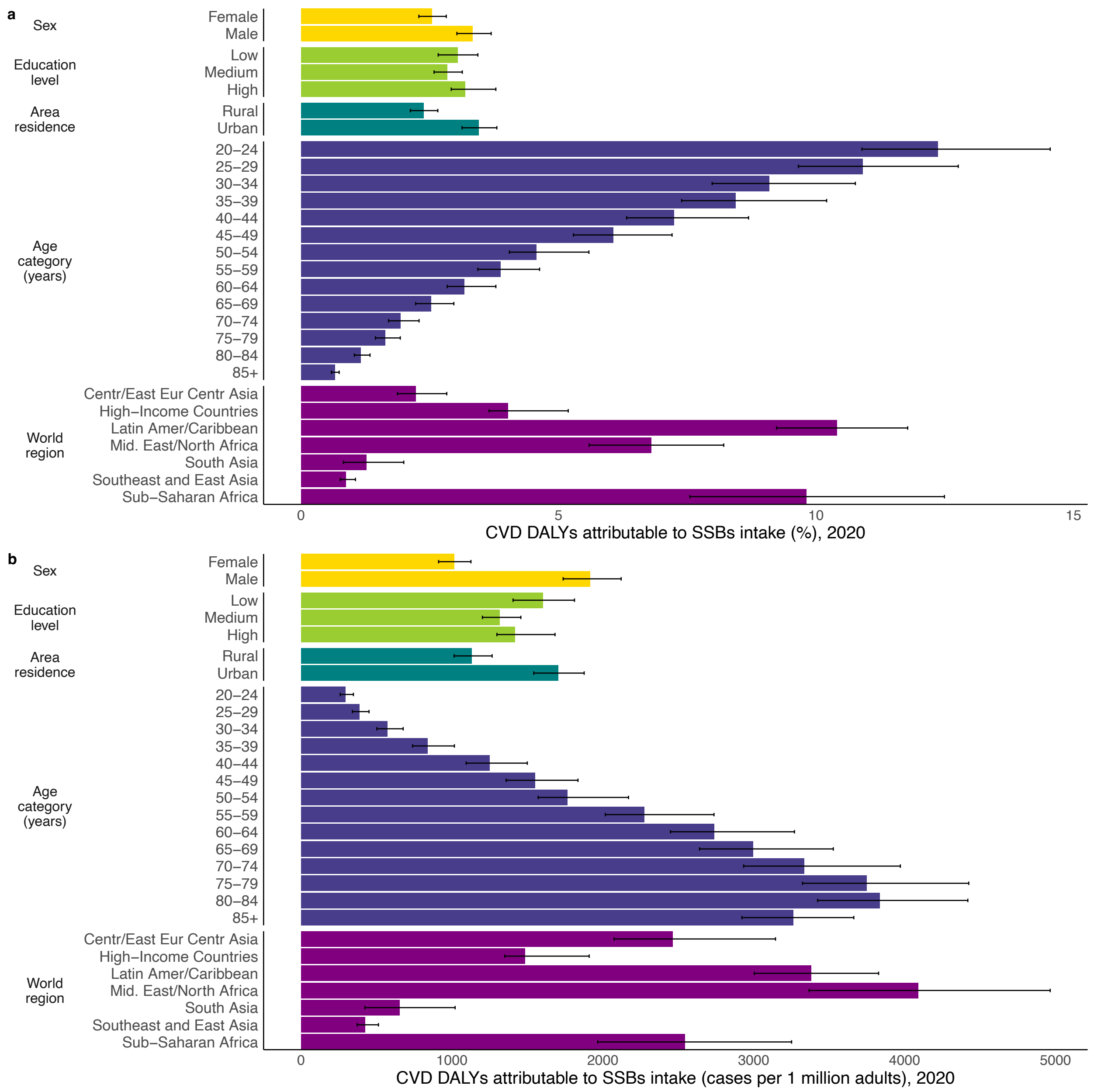

Supplementary Figure 6. **DALYs of CVD attributable to SSBs intake by key sociodemographic factors at the global level and by world region in 2020**. Bars represent the central estimate (median) of the proportional SSB-attributable CVD DALYs in the top panel (a) and the absolute SSB-attributable CVD DALYs per 1 million adults in the bottom panel (b). The error bars represent the 95% UI derived from the 2.5th and 97.5th percentiles of 1,000 multiway probabilistic Monte Carlo model simulations. The SSB-attributable absolute burden per 1 million adults was calculated by dividing the stratum absolute number of SSB-attributable cases by the stratum adult population (20+ years) in that same year and multiplying by 1 million. Colors differentiate sex (yellow), education (green light), area of residence (dark green), age category (dark purple), and world region (light purple). In prior GDD reports, the region Central/ Eastern Europe and Central Asia was referred as Former Soviet Union, and Southeast and East Asia was referred as Asia.

Centr/East Eur & Centr Asia, Central/Eastern Europe and Central Asia; CVD, cardiovascular disease; DALYs, disability-adjusted life years; GDD, Global Dietary Database; Latin Amer/Caribbean, Latin America/Caribbean; SSBs, sugar sweetened beverages; UIs, uncertainty intervals.

Supplementary Table 5. T2D burdens attributable to SSBs as per 1 million adults and proportional in 2020.[¥]

	T2D Incidence		T2D Deaths		T2D DALYs	
	Per 1M population	Proportional, %	Per 1M population	Proportional, %	Per 1M population	Proportional, %
Sex						
Female	387.9 (357.5-422.3)	9.5 (8.8-10.4)	14.7 (13.1-16.6)	4.7 (4.2-5.3)	900.9 (822.3-987.2)	6.6 (6.0-7.2)
Male	447.3 (412.8-490.6)	10.1 (9.3-11.1)	16.4 (14.7-18.3)	5.6 (5.0-6.3)	1026.2 (937.4-1120.8)	7.2 (6.6-7.9)
Education level						
Low	359.7 (325.5-398.4)	9.1 (8.2-10.1)	14.7 (12.7-16.8)	4.8 (4.2-5.5)	875.9 (778.0-974.0)	6.5 (5.8-7.2)
Medium	412.3 (382.4-447.4)	9.6 (8.9-10.4)	16.1 (14.3-18.3)	5.2 (4.6-5.9)	978.1 (894.7-1074.7)	6.9 (6.3-7.5)
High	531.3 (486.0-619.7)	11.1 (10.1-12.9)	16.4 (14.6-18.6)	5.8 (5.2-6.6)	1100.1 (1004.4-1253.2)	7.6 (6.9-8.7)
Area of residence						
Rural	244.4 (223.1-266.6)	7.0 (6.4-7.7)	10.1 (8.9-11.5)	3.6 (3.2-4.1)	593.4 (531.7-657.7)	4.9 (4.4-5.4)
Urban	543.2 (502.3-591.6)	11.3 (10.5-12.2)	19.5 (17.6-21.7)	6.1 (5.5-6.8)	1228.7 (1127.9-1338.8)	8.0 (7.4-8.7)
Age category						
20-24	304.7 (278.2-356.0)	14.5 (13.2-16.9)	0.9 (0.8-1.0)	23.3 (20.7-26.3)	193.2 (174.6-218.0)	17.0 (15.2-19.3)
25-29	301.4 (273.9-337.8)	15.6 (14.2-17.6)	1.2 (1.1-1.4)	22.3 (20.1-25.0)	281.1 (255.6-314.8)	15.6 (14.0-17.6)
30-34	346.9 (316.2-392.4)	14.8 (13.4-16.9)	2.3 (2.0-2.6)	19.9 (17.7-22.6)	411.0 (374.2-467.7)	14.3 (12.8-16.3)
35-39	438.2 (394.8-524.6)	13.9 (12.5-16.5)	3.6 (3.3-4.1)	18.6 (16.6-21.2)	602.7 (543.2-689.8)	14.1 (12.6-16.0)
40-44	548.0 (494.3-644.7)	12.9 (11.6-15.2)	6.7 (6.0-7.8)	15.4 (13.7-17.8)	865.0 (776.8-989.6)	12.8 (11.5-14.7)
45-49	601.0 (538.5-710.7)	10.9 (9.7-12.8)	12.4 (10.9-14.2)	12.6 (11.2-14.5)	1181.2 (1045.9-1364.5)	10.9 (9.7-12.6)
50-54	561.7 (507.3-691.6)	8.5 (7.6-10.5)	17.5 (15.6-20.3)	9.7 (8.6-11.2)	1366.6 (1229.6-1595.1)	8.6 (7.7-9.9)
55-59	592.5 (534.3-726.2)	7.6 (6.9-9.2)	25.9 (22.8-30.6)	8.2 (7.3-9.7)	1702.7 (1523.0-2025.1)	7.6 (6.8-8.9)
60-64	485.8 (438.7-576.2)	6.2 (5.6-7.4)	35.5 (31.2-42.0)	6.6 (5.8-7.8)	1913.6 (1706.7-2235.6)	6.2 (5.6-7.3)
65-69	341.9 (307.9-399.4)	5.1 (4.6-5.9)	43.4 (37.8-51.7)	5.4 (4.7-6.5)	1933.4 (1718.5-2274.3)	5.1 (4.6-6.0)
70-74	209.4 (185.8-251.8)	4.0 (3.5-4.8)	50.2 (43.3-61.3)	4.3 (3.7-5.2)	1825.9 (1610.6-2171.3)	4.1 (3.6-4.9)
75-79	96.8 (83.4-115.7)	3.2 (2.8-3.8)	62.5 (53.8-74.8)	3.5 (3.0-4.2)	1725.1 (1515.8-2037.0)	3.4 (3.0-4.1)
80-84	30.8 (26.0-38.5)	2.2 (1.8-2.7)	61.7 (53.3-75.1)	2.5 (2.2-3.1)	1272.7 (1130.6-1500.7)	2.5 (2.2-2.9)
85+	4.9 (4.2-6.0)	1.2 (1.0-1.4)	50.9 (44.4-59.7)	1.3 (1.2-1.5)	719.5 (637.5-837.3)	1.3 (1.2-1.5)
World region						
Centr/East Eur Centr Asia[§]	266.4 (224.7-323.3)	7.0 (5.9-8.5)	7.4 (6.1-9.6)	2.7 (2.2-3.5)	515.4 (436.3-649.4)	4.0 (3.4-5.0)
High-Income Countries	706.7 (638.3-873.6)	13.2 (12.0-16.2)	10.8 (9.9-14.3)	4.1 (3.8-5.4)	1082.7 (987.4-1373.0)	7.8 (7.2-10.0)
Latin Amer/Caribbean	1263.4 (1146.1-1399.7)	24.4 (22.2-26.9)	60.4 (53.3-69.8)	12.0 (10.6-13.8)	3463.4 (3123.8-3911.1)	16.9 (15.2-19.0)
Mid. East/North Africa	1000.5 (841.4-1161.3)	15.0 (12.6-17.3)	19.9 (16.4-24.1)	6.4 (5.3-7.8)	1626.2 (1365.2-1908.6)	10.0 (8.4-11.7)
South Asia	148.7 (103.8-213.4)	3.7 (2.6-5.3)	5.5 (3.5-8.6)	1.5 (1.0-2.4)	341.8 (230.2-503.9)	2.4 (1.6-3.5)
Southeast and East Asia[§]	118.6 (103.4-144.7)	3.1 (2.7-3.8)	3.8 (3.2-4.7)	1.7 (1.5-2.2)	260.4 (226.6-320.5)	2.1 (1.9-2.6)
Sub-Saharan Africa	589.3 (482.9-711.9)	21.5 (17.6-26.0)	44.5 (34.0-57.6)	13.0 (10.0-16.8)	2106.4 (1667.3-2607.5)	16.9 (13.4-20.9)

[¥] Data represent the central estimate (median) and 95% UI derived from the 2.5th and 97.5th percentiles of 1,000 multiway probabilistic Monte Carlo model simulations. The absolute burden per 1 million adults was calculated by dividing the absolute number of attributable cases by the country adult population (20+ years) in that year and multiplying by 1 million.

[§] In prior GDD reports, the region Central/ Eastern Europe and Central Asia was referred as Former Soviet Union, and Southeast and East Asia was referred as Asia.

DALYs, disability-adjusted life years; T2D, type 2 diabetes; UIs, uncertainty intervals.

Supplementary Table 6. CVD burdens attributable to SSBs as per 1 million adults and proportional in 2020.¥

	CVD Incidence		CVD Deaths		CVD DALYs	
	Per 1M population	Proportional, %	Per 1M population	Proportional, %	Per 1M population	Proportional, %
Sex						
Female	175.8 (158.7-193.9)	2.7 (2.4-3.0)	37.9 (34.1-42.4)	1.7 (1.6-1.9)	1014.6 (911.4-1125.9)	2.5 (2.3-2.8)
Male	285.1 (257.9-317.0)	3.4 (3.1-3.8)	62.1 (56.4-68.8)	2.4 (2.2-2.7)	1914.6 (1737.0-2121.3)	3.3 (3.0-3.7)
Education level						
Low	257.5 (226.2-294.1)	3.2 (2.8-3.6)	52.7 (46.2-60.3)	2.2 (1.9-2.5)	1601.6 (1405.0-1812.1)	3.0 (2.7-3.4)
Medium	209.8 (190.8-231.2)	2.9 (2.7-3.2)	45.9 (41.8-50.9)	2.0 (1.8-2.2)	1316.3 (1202.1-1456.6)	2.8 (2.6-3.1)
High	210.3 (192.1-240.4)	3.2 (2.9-3.6)	50.8 (46.4-60.3)	2.1 (1.9-2.5)	1418.1 (1298.1-1683.2)	3.2 (2.9-3.8)
Area of residence						
Rural	171.8 (152.6-192.3)	2.4 (2.1-2.7)	37.7 (33.6-42.3)	1.7 (1.5-1.9)	1131.5 (1014.4-1266.2)	2.4 (2.1-2.7)
Urban	272.8 (247.4-300.4)	3.6 (3.2-3.9)	58.9 (53.5-65.3)	2.4 (2.1-2.6)	1702.5 (1542.5-1876.2)	3.4 (3.1-3.8)
Age category						
20-24	26.2 (23.6-29.8)	12.0 (10.6-13.5)	4.1 (3.6-4.8)	12.8 (11.2-15.0)	295.3 (259.7-347.0)	12.4 (10.9-14.5)
25-29	39.5 (35.4-45.2)	11.2 (10.0-12.9)	5.7 (5.1-6.7)	11.2 (9.9-13.1)	385.1 (340.3-450.5)	10.9 (9.7-12.8)
30-34	69.1 (62.0-79.6)	9.8 (8.7-11.4)	9.3 (8.2-11.1)	9.3 (8.1-11.1)	570.7 (502.3-676.8)	9.1 (8.0-10.8)
35-39	113.4 (99.9-132.7)	9.1 (7.9-10.6)	15.1 (13.3-18.2)	8.6 (7.5-10.3)	838.5 (738.9-1016.3)	8.4 (7.4-10.2)
40-44	198.6 (175.3-231.3)	7.8 (6.8-9.2)	25.2 (21.8-30.6)	7.4 (6.4-8.9)	1248.6 (1094.1-1499.3)	7.2 (6.3-8.7)
45-49	273.5 (238.2-320.5)	6.2 (5.4-7.3)	34.6 (30.1-41.2)	6.2 (5.4-7.3)	1551.8 (1359.0-1835.3)	6.1 (5.3-7.2)
50-54	318.3 (280.8-375.0)	4.6 (4.1-5.4)	44.4 (39.2-54.1)	4.7 (4.1-5.7)	1764.1 (1571.4-2170.1)	4.6 (4.0-5.6)
55-59	407.0 (357.8-479.6)	4.0 (3.5-4.8)	65.0 (57.6-78.6)	3.9 (3.5-4.8)	2274.6 (2016.3-2736.6)	3.9 (3.4-4.6)
60-64	450.1 (399.2-533.1)	3.2 (2.9-3.8)	90.8 (81.0-107.5)	3.2 (2.9-3.8)	2737.0 (2448.2-3269.9)	3.2 (2.8-3.8)
65-69	474.4 (421.5-554.4)	2.6 (2.2-3.0)	117.2 (103.5-137.5)	2.6 (2.3-3.0)	2994.2 (2640.9-3527.2)	2.5 (2.2-3.0)
70-74	510.7 (448.2-600.4)	2.0 (1.7-2.3)	158.4 (139.0-188.5)	2.0 (1.7-2.3)	3335.0 (2933.2-3971.6)	1.9 (1.7-2.3)
75-79	562.8 (498.3-668.1)	1.6 (1.4-2.0)	223.5 (197.9-262.8)	1.7 (1.5-2.0)	3747.9 (3323.2-4424.9)	1.6 (1.4-1.9)
80-84	584.6 (519.8-677.2)	1.2 (1.1-1.4)	294.0 (261.4-339.7)	1.2 (1.0-1.4)	3836.1 (3423.2-4418.8)	1.2 (1.0-1.3)
85+	497.3 (444.4-561.6)	0.7 (0.6-0.8)	342.5 (306.4-385.6)	0.7 (0.6-0.7)	3261.1 (2921.6-3663.1)	0.7 (0.6-0.7)
World region						
Centr/East Eur Centr Asia§	348.9 (290.8-448.6)	2.4 (2.0-3.1)	98.5 (81.7-126.5)	1.6 (1.3-2.0)	2462.9 (2075.0-3144.2)	2.2 (1.9-2.8)
High-Income Countries	221.6 (203.2-263.7)	3.8 (3.5-4.5)	56.9 (52.2-71.1)	2.5 (2.3-3.1)	1483.2 (1350.2-1909.5)	4.0 (3.7-5.2)
Latin Amer/Caribbean	522.4 (465.4-593.4)	11.3 (10.1-12.8)	119.5 (104.7-138.2)	7.5 (6.6-8.7)	3380.8 (3003.2-3827.4)	10.4 (9.2-11.8)
Mid. East/North Africa	815.2 (673.6-979.8)	6.9 (5.7-8.3)	136.4 (110.3-166.8)	5.1 (4.1-6.2)	4090.5 (3367.2-4964.6)	6.8 (5.6-8.2)
South Asia	89.9 (57.0-145.4)	1.2 (0.7-1.9)	19.6 (12.5-30.9)	0.9 (0.6-1.5)	653.6 (423.2-1021.1)	1.3 (0.8-2.0)
Southeast and East Asia§	46.8 (41.0-57.1)	0.6 (0.6-0.8)	12.9 (11.4-15.4)	0.5 (0.5-0.6)	425.3 (371.8-513.1)	0.9 (0.8-1.1)
Sub-Saharan Africa	431.5 (333.3-545.0)	10.5 (8.1-13.3)	80.9 (59.9-107.6)	7.4 (5.5-9.9)	2542.4 (1966.2-3250.8)	9.8 (7.5-12.5)

¥ Data represent the central estimate (median) and 95% UI derived from the 2.5th and 97.5th percentiles of 1,000 multiway probabilistic Monte Carlo model simulations. The SSB-attributable absolute burden per 1 million adults was calculated by dividing the stratum absolute number of SSB-attributable cases by the stratum adult population (20+ years) in that same year and multiplying by 1 million.
§ In prior GDD reports, the region Central/ Eastern Europe and Central Asia was referred as Former Soviet Union, and Southeast and East Asia was referred as Asia.
CVD, cardiovascular disease; DALYs, disability-adjusted life years; UIs, uncertainty intervals.

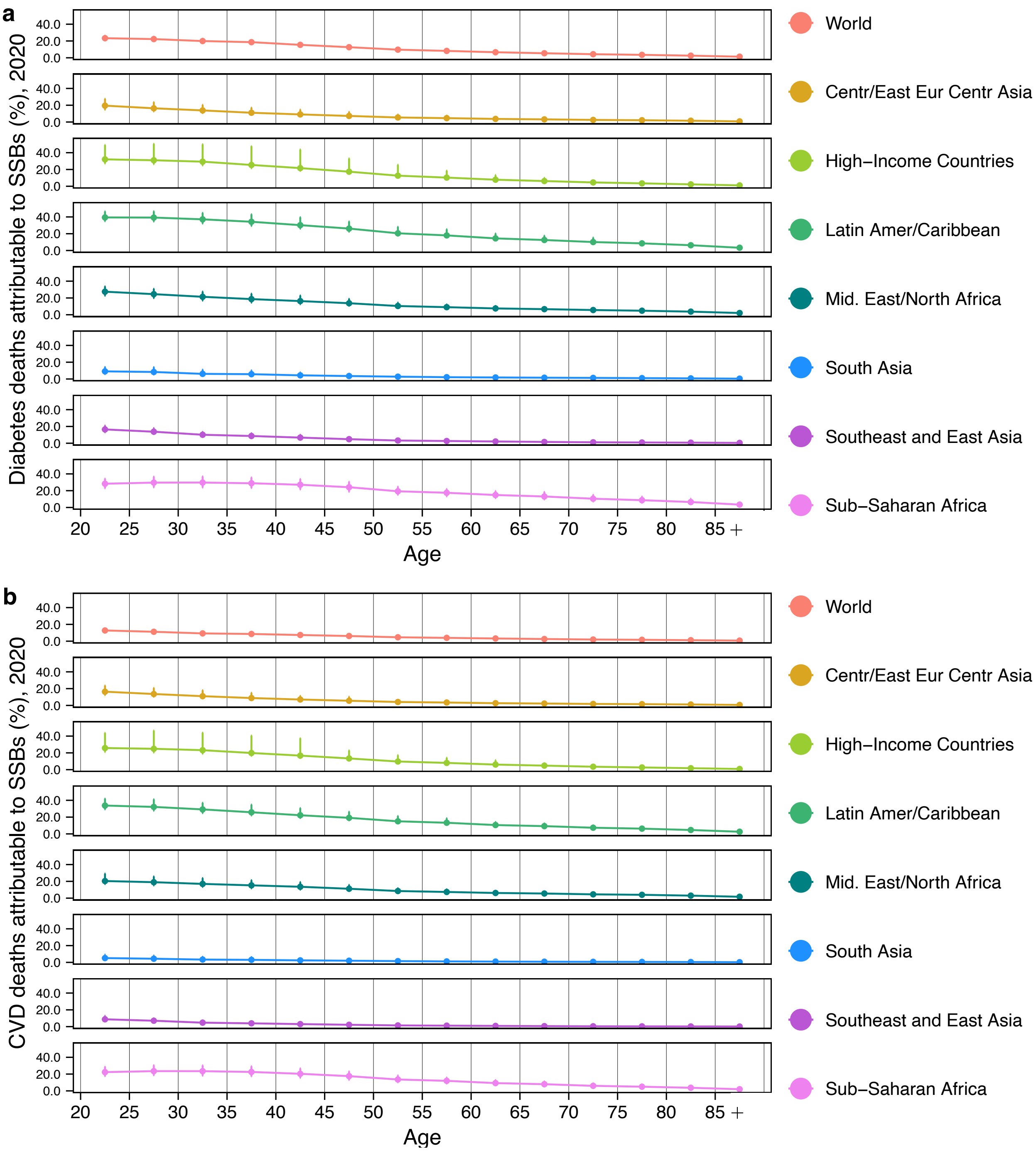

Supplementary Figure 7. **Proportional deaths of T2D and CVD attributable to SSBs intake among adults (20+ years) jointly stratified by world region and age in 2020.** The filled circles represent the central estimate (median) of the proportional SSB-attributable **(a)** T2D deaths and **(b)** CVD deaths. The error bars represent the 95% UI derived from the 2.5th and 97.5th percentiles of 1,000 multiway probabilistic Monte Carlo model simulations. The age groups are 20-24, 25-29, 30-34, 35-39, 40-44, 45-49, 50-54, 55-59, 60-64, 65-69, 70-74, 75-79, 80-84, 85+ years. In prior GDD reports, the region Central/ Eastern Europe and Central Asia was referred as Former Soviet Union, and Southeast and East Asia was referred as Asia.

Centr/East Eur Centr Asia, Central/Eastern Europe and Central Asia; CVD, cardiovascular disease; GDD, Global Dietary Database; Latin Amer/Caribbean, Latin America/Caribbean; SSBs, sugar sweetened beverages; T2D, type 2 diabetes; UIs, uncertainty intervals.

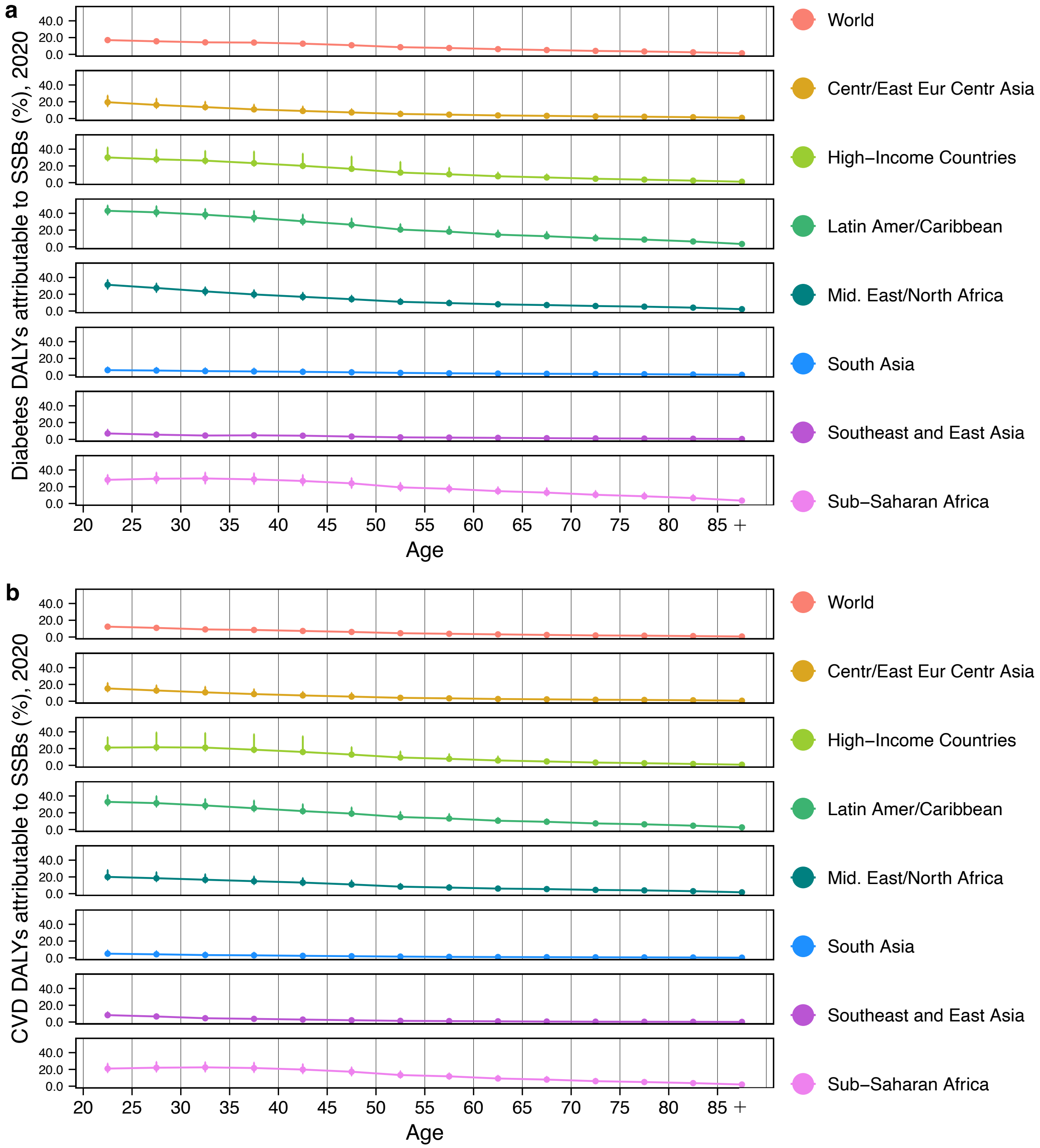

Supplementary Figure 8. **Proportional DALYs of T2D and CVD attributable to SSBs intake among adults (20+ years) jointly stratified by world region and age in 2020.** The filled circles represent the central estimate (median) of the proportional SSB-attributable **(a)** T2D DALYs and **(b)** CVD DALYs. The error bars represent the 95% UI derived from the 2.5th and 97.5th percentiles of 1,000 multiway probabilistic Monte Carlo model simulations. The age groups are 20-24, 25-29, 30-34, 35-39, 40-44, 45-49, 50-54, 55-59, 60-64, 65-69, 70-74, 75-79, 80-84, 85+ years. In prior GDD reports, the region Central/ Eastern Europe and Central Asia was referred as Former Soviet Union, and Southeast and East Asia was referred as Asia.

Centr/East Eur Centr Asia, Central/Eastern Europe and Central Asia; CVD, cardiovascular disease; DALYs, disability-adjusted life years; GDD, Global Dietary Database; Latin Amer/Caribbean, Latin America/Caribbean; SSBs, sugar sweetened beverages; T2D, type 2 diabetes; UIs, uncertainty intervals.

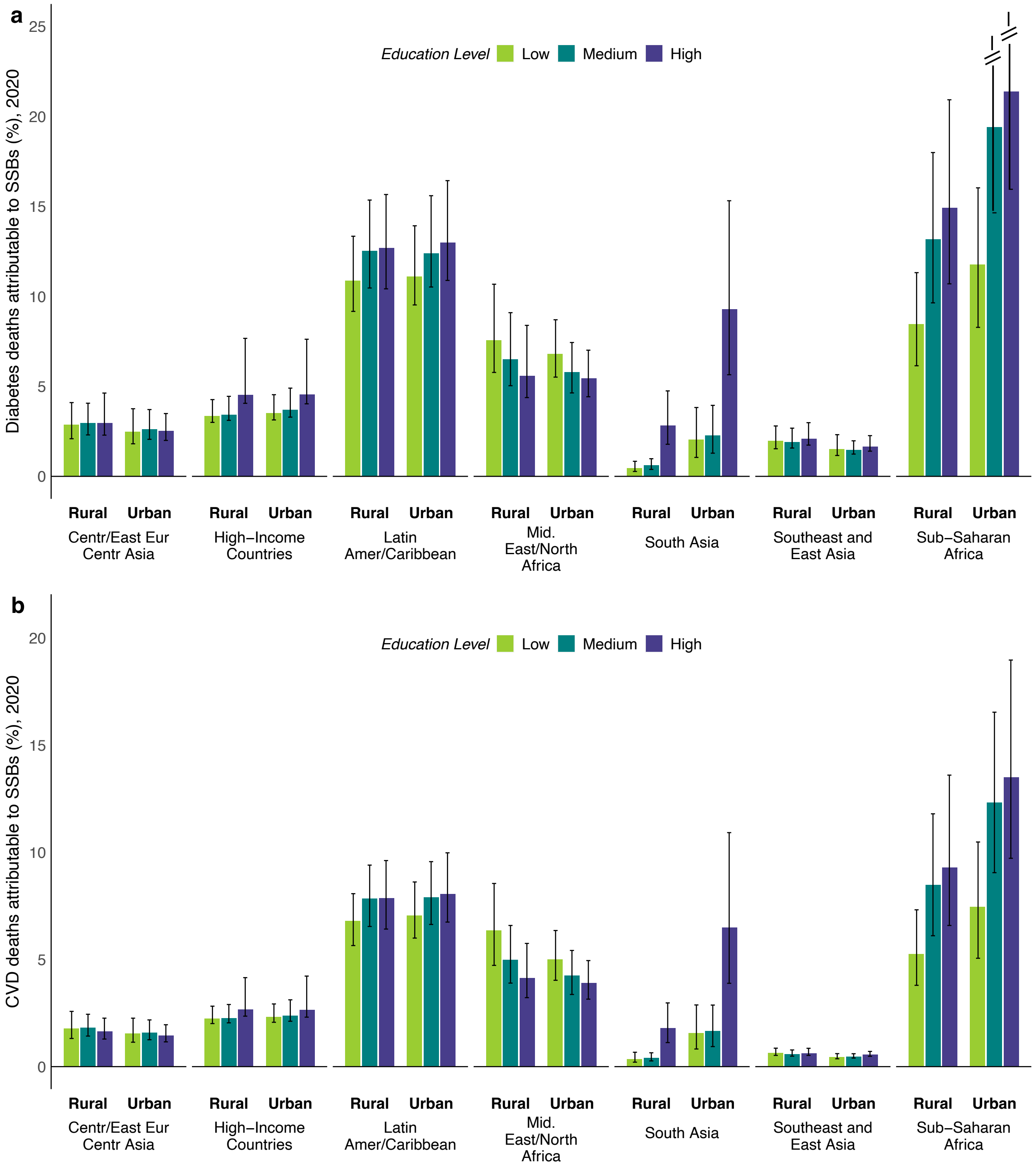

Supplementary Figure 9. **Proportional deaths of T2D and CVD attributable to intake of SSBs among adults (20+ years) jointly stratified by world region, area of residence, and education level in 2020.** Bars represent the central estimate (median) of the proportional SSB-attributable **(a)** T2D deaths and **(b)** CVD deaths. The error bars represent the 95% UI derived from the 2.5th and 97.5th percentiles of 1,000 multiway probabilistic Monte Carlo model simulations. Values were truncated at 25 for the top panel (a) and 95% UIs above are shown with a dashed line. Colors indicate high educated (purple), medium educated (dark light), low educated (light green) adults. In prior GDD reports, the region Central/ Eastern Europe and Central Asia was referred as Former Soviet Union, and Southeast and East Asia was referred to as Asia.

Centr/East Eur Centr Asia, Central/Eastern Europe and Central Asia; CVD, cardiovascular disease; GDD, Global Dietary Database; Latin Amer/Caribbean, Latin America/Caribbean; SSBs, sugar sweetened beverages; T2D, type 2 diabetes; UIs, uncertainty intervals.

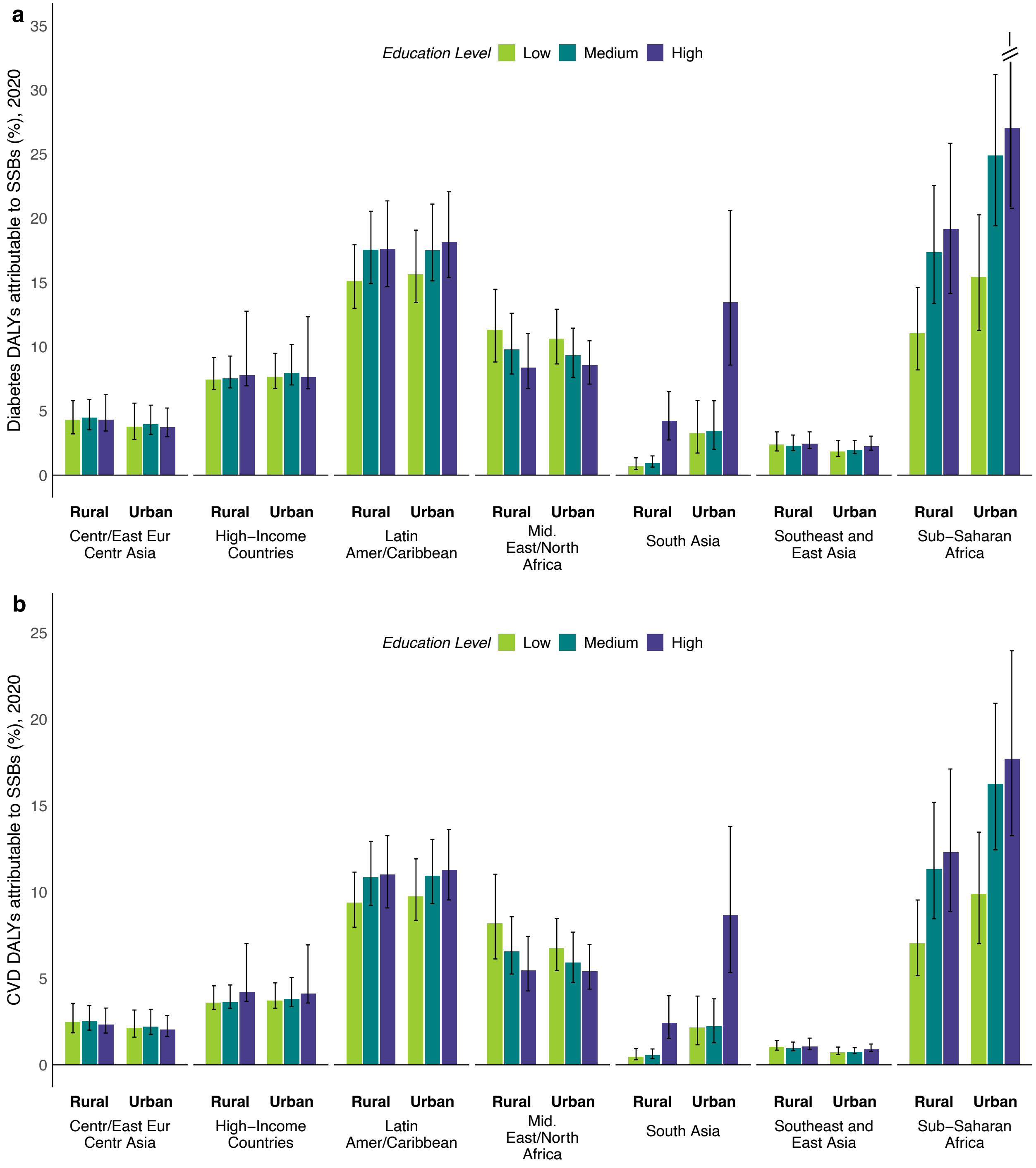

Supplementary Figure 10. **Proportional DALYs of T2D and CVD attributable to intake of SSBs among adults (20+ years) jointly stratified by world region, area of residence, and education level in 2020.** Bars represent the central estimate (median) of the proportional SSB-attributable **(a)** T2D DALYs and **(b)** CVD DALYs. The error bars represent the 95% UI derived from the 2.5th and 97.5th percentiles of 1,000 multiway probabilistic Monte Carlo model simulations. Values were truncated at 35 for the top panel (a) and 95%UIs above are shown with a dashed line. Colors indicate high educated (purple), medium educated (dark light), low educated (light green) adults. In prior GDD reports, the region Central/ Eastern Europe and Central Asia was referred as Former Soviet Union, and Southeast and East Asia was referred to as Asia.

Centr/East Eur Centr Asia, Central/Eastern Europe and Central Asia; CVD, cardiovascular disease; DALYs, disability-adjusted life years; GDD, Global Dietary Database; Latin Amer/Caribbean, Latin America/Caribbean; SSBs, sugar sweetened beverages; T2D, type 2 diabetes; UIs, uncertainty intervals.

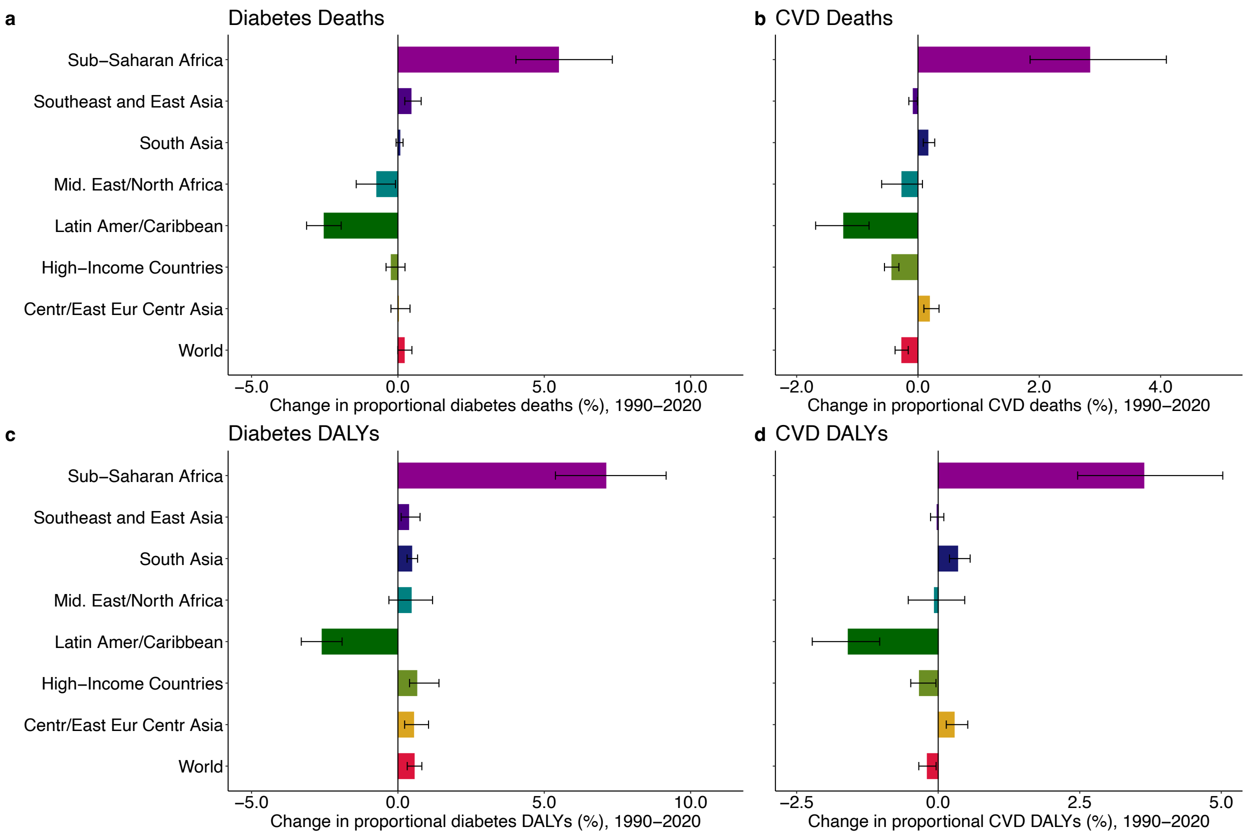

Supplementary Figure 11. **Change in proportional deaths and DALYs of T2D and CVD attributable to intake of SSBs among adults (20+ years) from 1990 to 2020 by world region.** Bars represent the central estimate (median) of the difference between 1990 and 2020 for the proportional SSB-attributable **(a)** T2D deaths, **(b)** CVD deaths, **(c)** T2D DALYs, and **(d)** CVD DALYs. The error bars represent the 95% UI derived from the 2.5th and 97.5th percentiles of 1,000 multiway probabilistic Monte Carlo model simulations. In prior GDD reports, the region Central/ Eastern Europe and Central Asia was referred as Former Soviet Union, and Southeast and East Asia was referred to as Asia.

Centr/East Eur Centr Asia, Central/Eastern Europe and Central Asia; CVD, cardiovascular disease; DALYs, disability-adjusted life years; GDD, Global Dietary Database; Latin Amer/Caribbean, Latin America/Caribbean; SSBs, sugar sweetened beverages; T2D, type 2 diabetes; UIs, uncertainty intervals.

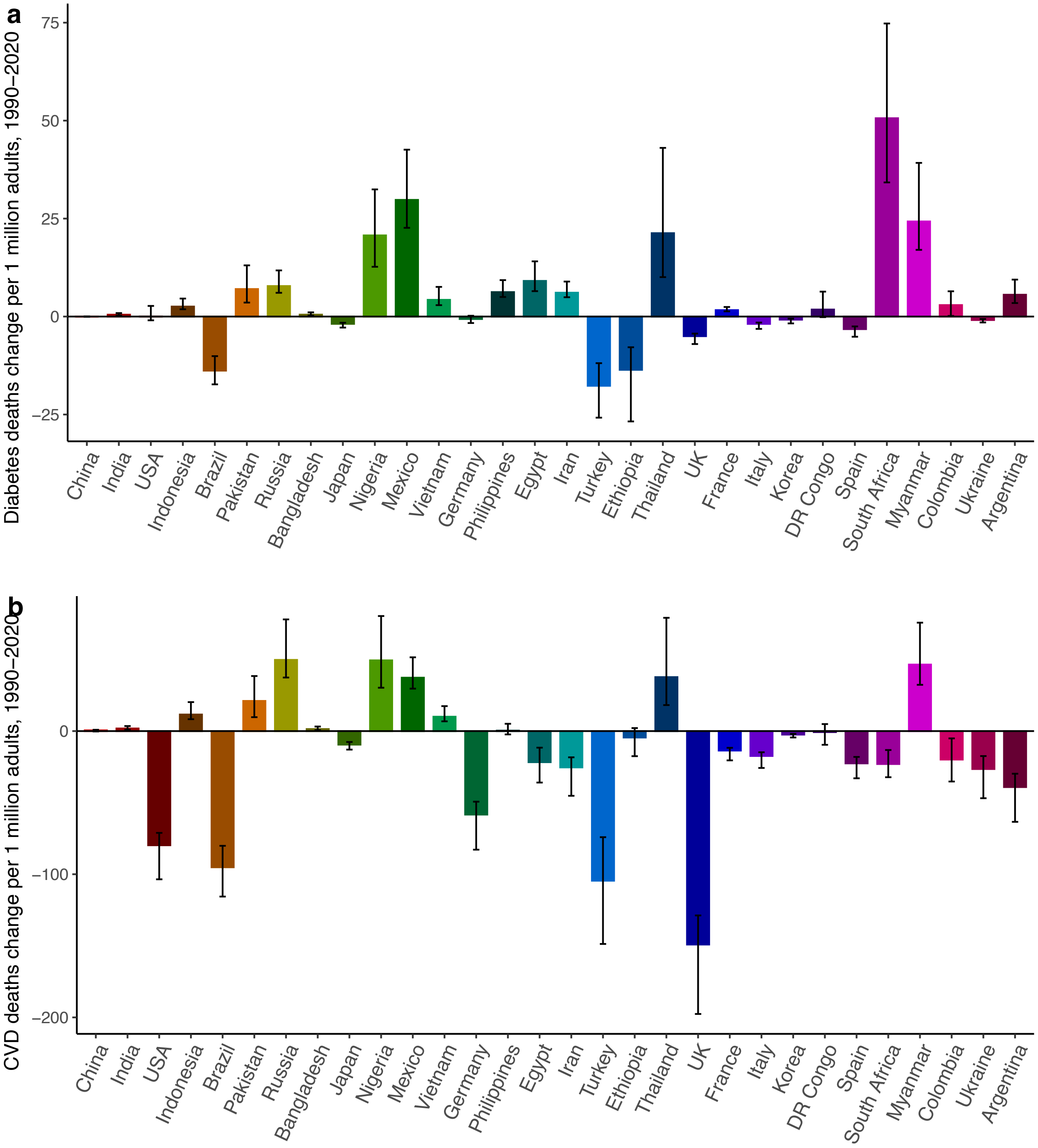

Supplementary Figure 12. **Change in deaths per 1 million adults of T2D and CVD attributable to intake of SSBs among adults (20+ years) from 1990 to 2020 among the 30 most populous countries.** Bars represent the central estimate (median) of the difference between 1990 and 2020 of the absolute SSB-attributable **(a)** T2D deaths and **(b)** CVD deaths. The error bars represent the 95% UI derived from the 2.5th and 97.5th percentiles of 1,000 multiway probabilistic Monte Carlo model simulations. The SSB-attributable absolute burden per 1 million adults was calculated by dividing the country absolute number of SSB-attributable cases by the country adult population (20+ years) in that same year. The difference in the absolute burden per 1 million adults was calculated by subtracting the per 1 million adult burden in 1990 from the per 1 million adult burden in 2020. Countries are ordered left to right from most to least populous based on 2020 adult (20+ years) population data.

CVD, cardiovascular disease; SSBs, sugar sweetened beverages; T2D, type 2 diabetes; UIs, uncertainty intervals.

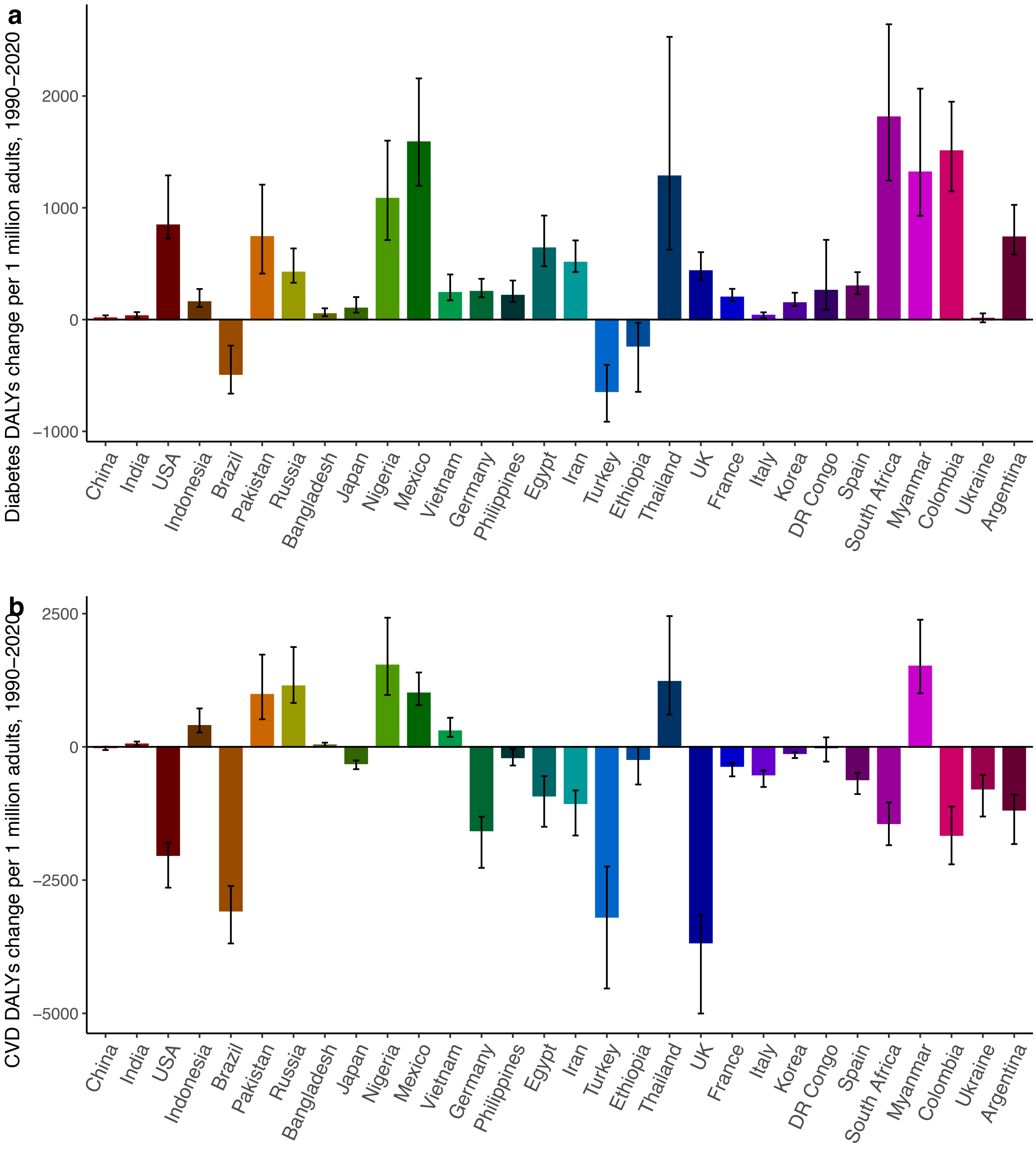

Supplementary Figure 13. **Change in DALYs per 1 million adults of T2D and CVD attributable to intake of SSBs among adults (20+ years) from 1990 to 2020 among the 30 most populous countries.** Bars represent the central estimate (median) of the difference between 1990 and 2020 of the absolute SSB-attributable **(a)** T2D DALYs and **(b)** CVD DALYs. The error bars represent the 95% UI derived from the 2.5th and 97.5th percentiles of 1,000 multiway probabilistic Monte Carlo model simulations. The SSB-attributable absolute burden per 1 million adults was calculated by dividing the country absolute number of SSB-attributable cases by the country adult population (20+ years) in that same year. The difference in the absolute burden per 1 million adults was calculated by subtracting the per 1 million adult burden in 1990 from the per 1 million adult burden in 2020. Countries are ordered left to right from most to least populous based on 2020 adult (20+ years) population data.

CVD, cardiovascular disease; DALYs, disability-adjusted life years; SSBs, sugar sweetened beverages; T2D, type 2 diabetes; UIs, uncertainty intervals.

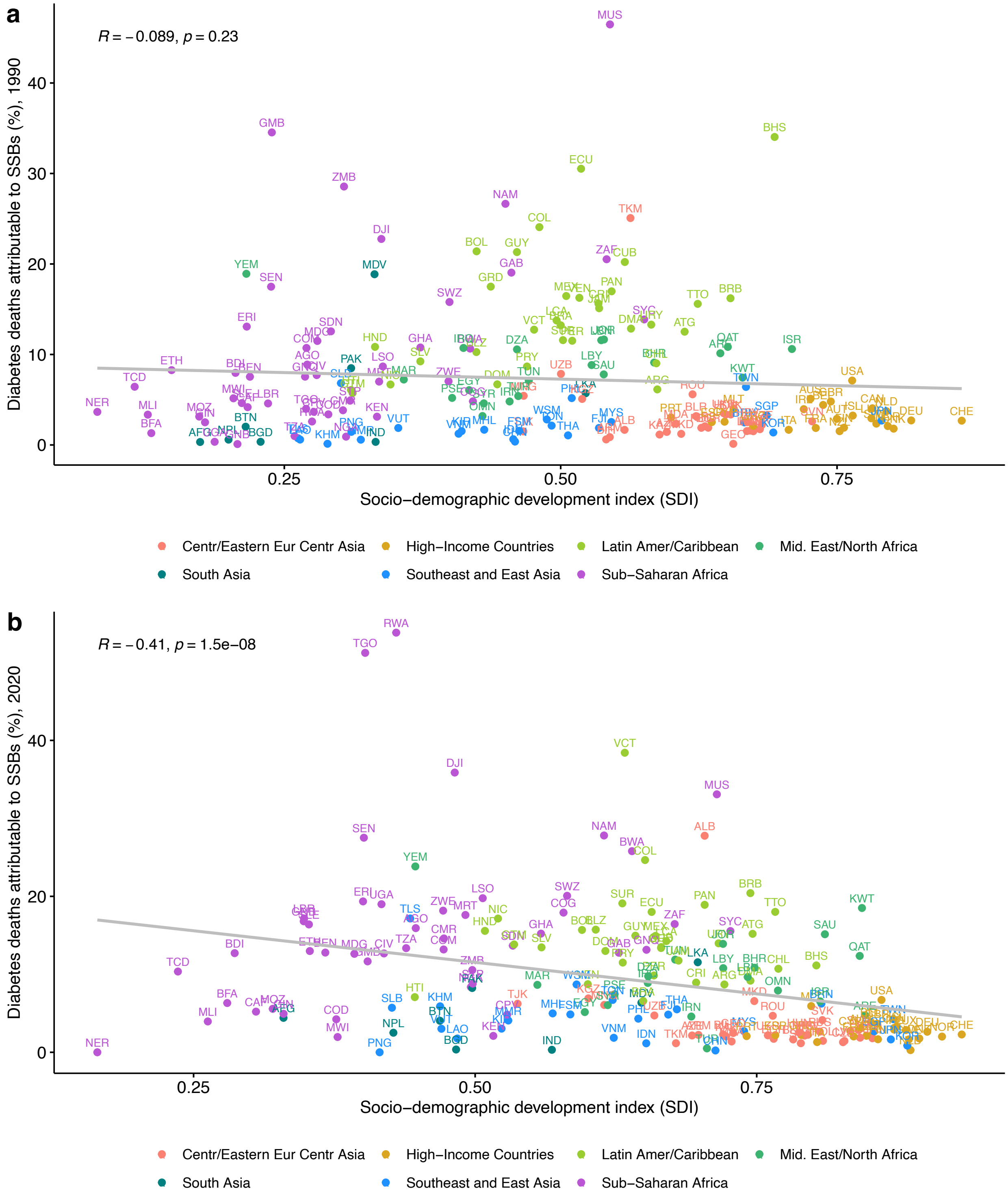

Supplementary Figure 14. **National correlation of proportional SSB-attributable T2D deaths and SDI at the national level in 1990 and 2020. (a)** 1990 and **(b)** 2020. Points represent the 184 countries included in this analysis (labeled with their ISO3 code and colored based on world region). The gray line represents the overall linear association, with Spearman correlation coefficient and associated P value (two-tailed) provided. No adjustments were made for multiple comparisons. SDI is a measure of a nation's development expressed on a scale of 0 to 1 sourced from the Global Burden of Disease study, based on a compositive average of the rankings of income per capita, average educational attainment and fertility rates.

Centr/Eastern Eur Centr Asia, Central/Eastern Europe and Central Asia; GDD, Global Dietary Database; Latin Amer/Caribbean, Latin America/Caribbean; SDI, sociodemographic development index; SSBs, sugar sweetened beverages; T2D, type 2 diabetes; UIs, uncertainty intervals.

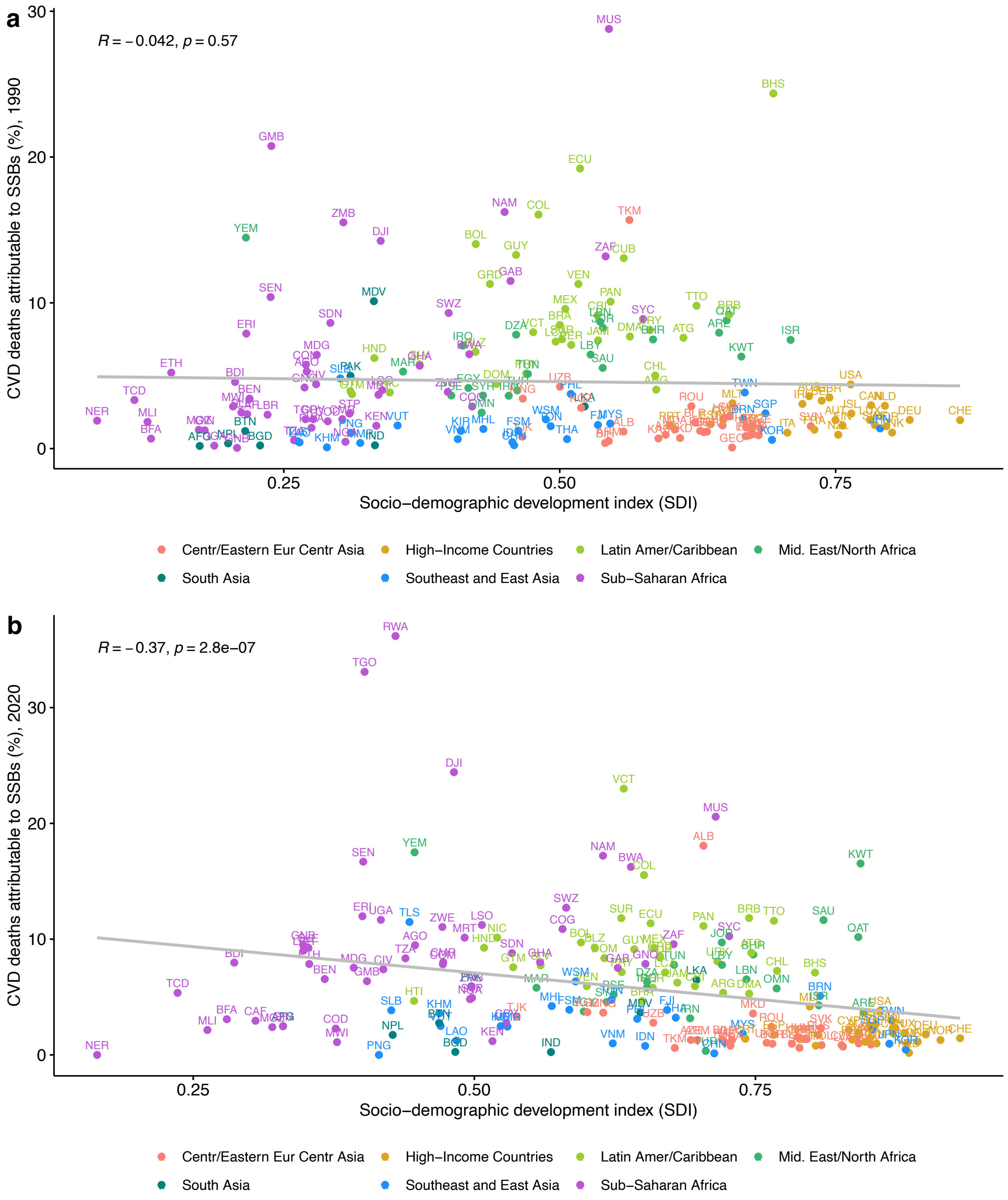

Supplementary Figure 15. **National correlation of proportional SSB-attributable CVD deaths and SDI at the national level in 1990 and 2020. (a)** 1990 and **(b)** 2020. Points represent the 184 countries included in this analysis (labeled with their ISO3 code and colored based on world region). The gray line represents the overall linear association, with Spearman correlation coefficient and associated P value (two-tailed) provided. No adjustments were made for multiple comparisons. SDI is a measure of a nation's development expressed on a scale of 0 to 1 sourced from the Global Burden of Disease study, based on a compositive average of the rankings of income per capita, average educational attainment and fertility rates.

Centr/Eastern Eur Centr Asia, Central/Eastern Europe and Central Asia; CVD, cardiovascular disease; GDD, Global Dietary Database; Latin Amer/Caribbean, Latin America/Caribbean; SDI, sociodemographic development index; SSBs, sugar sweetened beverages; UIs, uncertainty intervals.

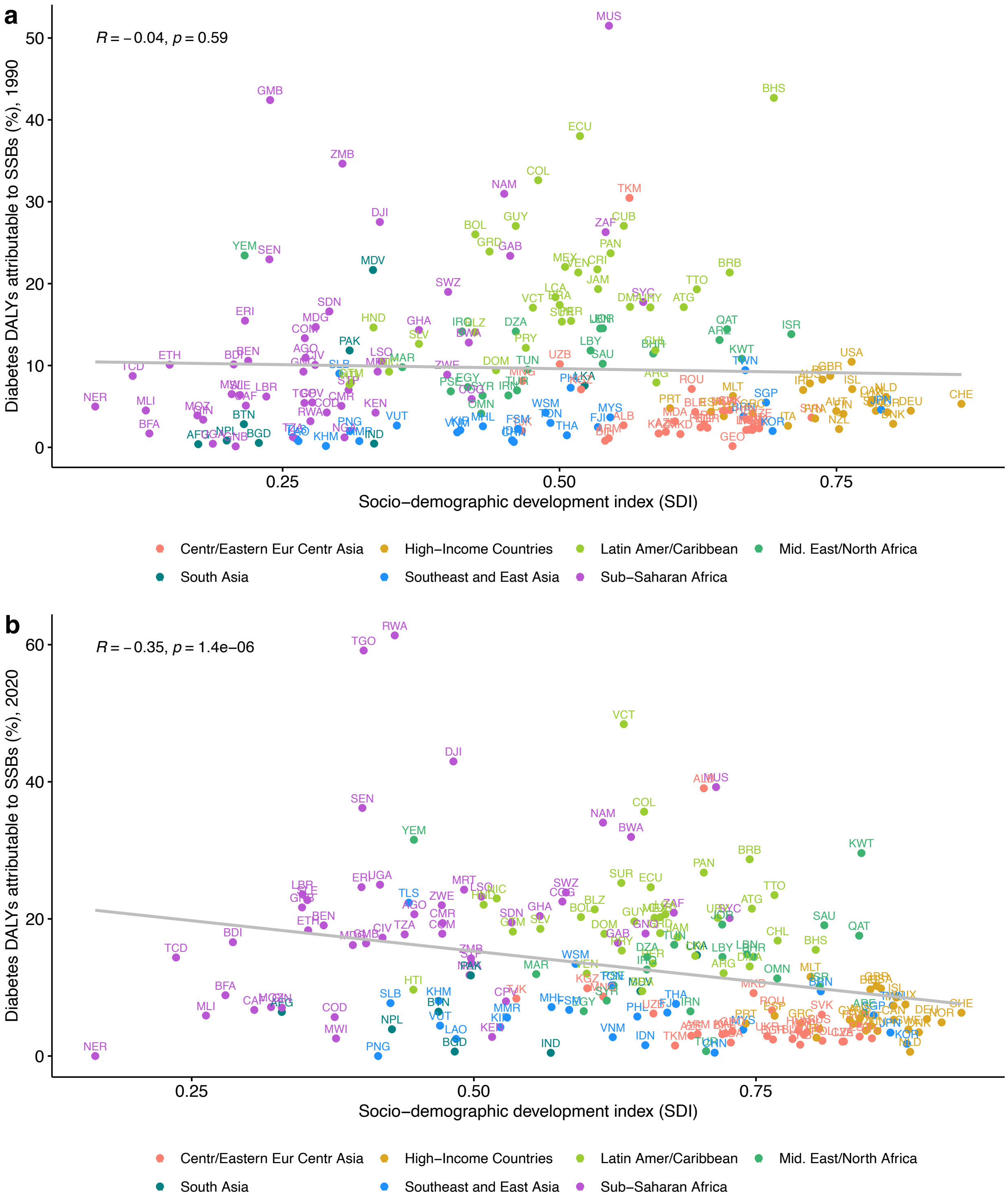

Supplementary Figure 16. **National correlation of proportional SSB-attributable T2D DALYs and SDI at the national level in 1990 and 2020. (a)** 1990 and **(b)** 2020. Points represent the 184 countries included in this analysis (labeled with their ISO3 code and colored based on world region). The gray line represents the overall linear association, with Spearman correlation coefficient and associated P value (two-tailed) provided. No adjustments were made for multiple comparisons. SDI is a measure of a nation's development expressed on a scale of 0 to 1 sourced from the Global Burden of Disease study, based on a compositive average of the rankings of income per capita, average educational attainment and fertility rates.

Centr/Eastern Eur Centr Asia, Central/Eastern Europe and Central Asia; DALYs, disability-adjusted life years;GDD, Global Dietary Database; Latin Amer/Caribbean, Latin America/Caribbean; SDI, sociodemographic development index; SSBs, sugar sweetened beverages; T2D, type 2 diabetes; UIs, uncertainty intervals.

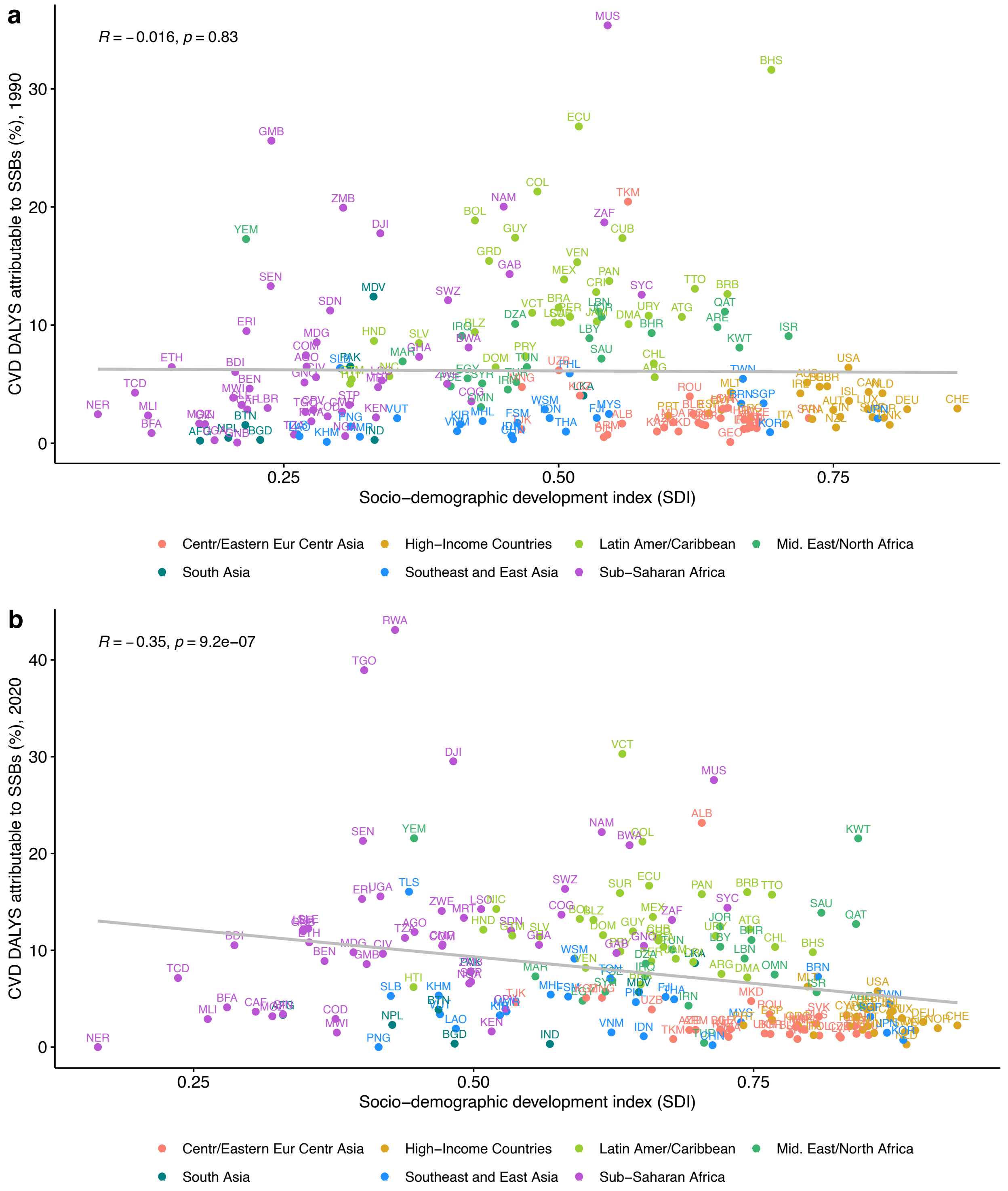

Supplementary Figure 17. **National correlation of proportional SSB-attributable CVD DALYs and SDI at the national level in 1990 and 2020. (a)** 1990 and **(b)** 2020. Points represent the 184 countries included in this analysis (labeled with their ISO3 code and colored based on world region). The gray line represents the overall linear association, with Spearman correlation coefficient and associated P value (two-tailed) provided. No adjustments were made for multiple comparisons. SDI is a measure of a nation's development expressed on a scale of 0 to 1 sourced from the Global Burden of Disease study, based on a compositive average of the rankings of income per capita, average educational attainment and fertility rates.

Centr/Eastern Eur Centr Asia, Central/Eastern Europe and Central Asia; CVD, cardiovascular disease; DALYs, disability-adjusted life years; GDD, Global Dietary Database; Latin Amer/Caribbean, Latin America/Caribbean; SDI, sociodemographic development index; SSBs, sugar sweetened beverages; UIs, uncertainty intervals.

Supplementary Table 7. Primary data inputs and associated data sources for comparative risk assessment analysis.

Model input	Source	Notes and assumptions
Population dietary SSB intake estimate distributions (g/d)	Global Dietary Database (GDD) 2020	The GDD systematically searched for and compiled representative data on individual-level dietary intakes from national and sub-national surveys. The final GDD model incorporated 1,225 dietary surveys representing 185 countries and 99.0% of the global population in 2020. Of these, 451 surveys reported data on SSBs, totaling 2.9 million individuals from 118 countries representing 87.1% of the global population. All intakes are reported adjusted to 2,000 kcal/day for ages 20–74 years, and 1,700 kcal/day for ages 75+ years through the residual method to reduce measurement error and account for regional differences in body size, metabolic efficiency, and physical activity. Units were characterized to be consistent with studies providing evidence on etiologic diet-disease relationships. A Bayesian hierarchical model incorporating 1,000 Monte Carlo simulations estimated the mean intake levels, with 95% uncertainty based on the 2.5th and 97.5th percentiles of the distribution, for each of the 264 strata within 185 countries from 1990 through 2020. Stratum-specific values were combined weighted to the stratum's proportion of the population for global, regional, national, or other subgroup analyses.
Overweight and underweight prevalence distributions	NCD-Risk Factor Collaboration	Stratum-level mean BMI estimated based on a Bayesian hierarchical model incorporating 1,820 national, subnational, or community population-based studies with measurements of height and weight in over 97 million adults. Self-report height and weight measurements were not included due to potential bias. A Monte Carlo simulation algorithm was used to fit the model and calculate posterior distributions of mean BMI and its 95% uncertainty, and then converted into overweight and underweight prevalence using established multivariable regression models. Overweight (BMI ≥ 25 kg/m^2) and underweight (BMI <18.5 kg/m^2).
Ischemic heart disease, Ischemic stroke, and T2D incidence, mortality, and DALYs estimate distributions	Global Burden of Disease (GBD) Study 2021	The GBD collected data from censuses, household surveys, civil registration, vital statistics, and other relevant records to estimate incidence, prevalence, mortality, years lived with disability (YLDs), years of life lost (YLLs), and disability-adjusted life-years (DALYs) for 371 diseases and injuries. These estimates were stratified by 204 countries and territories, 23 age groups, and sex, yearly from 1990 to 2021. For this analysis, we used GBD estimates of incidence, mortality, and DALYs for T2D, ischemic heart disease*, and ischemic stroke for years 1990 and 2020. The GBD defines T2D as fasting plasma glucose greater or equal to 126 mg/dL (7 mmol/L) or reporting the use of T2D medication. Because the type 2 T2D diagnostic criteria found in the studies included in GBD is not specific enough, T2D type 1 and 2 were differentiated by subtracting the estimates of T2D type 1 from the overall T2D at the most stratified level of age, sex, location, and year. Ischemic heart disease is estimated in GBD as the aggregate of myocardial infraction (heart attack), angina (chest pain), or ischemic cardiomyopathy (heart failure due to ischemic heart disease). Ischemic stroke is defined according to the WHO criteria as rapidly developing clinical signs of (usually focal) cerebral function disturbance lasting over 24 hours, or leading to death.
Linear, BMI-stratified effects of SSBs on weight gain or weight loss	Pooled analyses of cohort studies	Based on pooled results from three separate prospective cohort studies with follow-up periods from 1986 to 2006 including 120,877 individuals who were free of obesity (BMI ≥30) or chronic diseases, and with complete data on weight and lifestyle habits at baseline. Independent relations of changes in dietary habits with BMI change were assessed in 4-year periods using linear regression with robust variance and accounting for within-person repeated measures. Median intake frequencies of SSBs were summed to compute average servings/day. BMI changes shown are for 1 serving/day increased consumption, converted to standardized g/day servings.
Direct, proportional, age-adjusted effects of SSBs on CMDs	Meta-analyses of prospective cohort and RCTs	Direct relationships of SSB intake and ischemic heart disease, ischemic stroke, and T2D were obtained from published systematic reviews and evidence grading, based on meta-analyses of prospective cohort studies and randomized controlled trials including multivariable adjustment for age, sex, BMI, and other risk factors. Age-specific relative risks were calculated for each SSB-disease etiologic relationship based on the mean age-at-event and follow-up duration.
Direct, proportional, age-adjusted effects of BMI on CMDs	Pooled analyses of cohort studies	Age-specific relationships of BMI with incident ischemic heart disease, ischemic stroke, and T2D were obtained from pooled analysis of multiple cohort studies. Age-specific relative risks were calculated for each BMI-disease etiologic relationship based on the mean age-at-event and follow-up duration.
Optimal intake levels for dietary factors	Systemic review of etiologic effects and de-novo analyses	Systemic review of etiologic effects of diet on disease risk and de-novo analyses. Optimal intake levels for each dietary factor were derived from previously reported analyses or calculated de-novo, based on observed levels associated with mortality/morbidity in meta-analyses, feasibility based on observed national consumption levels globally, and consistency with major dietary guidelines.
Population demographic data	UN Population Division; Barro and Lee 2013	The GDD obtained annual, national level age, sex, and urbanicity population demographics from the UN population division; and annual national level educational attainment from Barro and Lee 2013 dataset. These values were used to determine the population proportion of each population stratum for informing population weights used in mean dietary intake estimates as well as for reporting summary T2D burden results in the present analysis.

BMI, body mass index; CVD, cardiovascular disease; CMD, cardiometabolic disease; DALYs, disability-adjusted life years; FPG, fasting plasma glucose; GBD, Global Burden of Disease Study; GDD, Global Dietary Database; NCD, non-communicable disease; RCT, randomized controlled trials; SSBs, sugar-sweetened beverages; T2D, type 2 diabetes; UN, United Nations; WHO, World Health Organization. *Ischemic heart disease is also referred as coronary heart disease

Supplementary Table 8. Etiologic effects of SSBs and BMI on ischemic heart disease, ischemic stroke, T2D, and BMI.

Risk factor	Outcome	Source of original RR	Original RR (95% CI)	Unit for original RR	Age at event¶	Effect size (RR [95% CI]†,# or change [95% CI]‡)	Unit for RR used in this analysis	Optimal mean intake§ (servings/day)
SSBs	Ischemic heart disease$	Yin *et al.*, 2021	1.15 (1.09-1.22)	250 mL per day	64.55 years	1.14 (1.09 - 1.21)†	1 8oz serving per day (248 g)	0
SSBs	Ischemic stroke	Bechthold *et al.*, 2019	1.07 (1.02-1.12)	250 mL per day	67.51 years	1.07 (1.02 - 1.11)†	1 8oz serving per day (248 g)	0
SSBs	T2D	Qin *et al.*, 2020	1.19 (1.13-1.25)	250 mL per day	55.23 years	1.18 (1.12 - 1.24)†	1 8oz serving per day (248 g)	0
BMI	Ischemic heart disease	Lu *et al.*, 2014	1.27 (1.23-1.31)	5 kg/m^2	66.28 years	1.05 (1.04 - 1.06)#	1 kg/m^2	NA
BMI	Ischemic stroke	Singh *et al.*, 2013	1.50 (1.40-1.60)	5 kg/m^2	57.40 years	1.08 (1.07 - 1.10)#	1 kg/m^2	NA
BMI	T2D	Singh *et al.*, 2013	2.32 (2.04-2.63)	5 kg/m^2	57.40 years	1.18 (1.15 - 1.21)#	1 kg/m^2	NA
SSBs	BMI <25	Mozaffarian *et al.* 2011	0.09 (0.05, 0.14)	1 serving/day	NA	0.09 (0.05, 0.14)‡	1 8oz serving per day (248 g)	NA
SSBs	BMI ≥25	Mozaffarian *et al.* 2011	0.23 (0.14, 0.32)	1 serving/day	NA	0.23 (0.14, 0.32)‡	1 8oz serving per day (248 g)	NA

¶ Age at event risk was calculated by first taking the mean of the range of age at baseline of each individual study in the metanalysis and then adding it up to the follow-up time of the corresponding study as half of the max follow-up time, or as 2/3 of the mean/median follow-up, depending on which was reported. The value obtained was next weighed by its corresponding weighting fraction reported in the metanalysis. Finally, the weighted values were added up to obtain the final age at event for the metanalysis. Age at event for SSBs intake on BMI is not reported as it is not necessary for the present analysis. The calculations for age-at-event are shown in **Supplementary Tables 10-12 and Supplementary Data 4.**
† The RRs for the association between SSBs intake and disease were standardized from the originally reported 250 mL serving size to our unit of interest, which is a daily intake of one 8oz serving/day (248g). To achieve this, the original estimate was first transformed to its natural logarithm. Then it was divided by 260, representing the grams equivalent to 250 mL of SSB (1 fl oz = 29.6 mL; 1 fl oz = 30.7 g). Next the result was multiplied by 248, and finally it was exponentiated to obtain the final relative risk. This methodology was also employed to calculate the corresponding 95% CI. These relative risks are based on models estimating log linear relationship between dietary intake and the disease estimate (direct effects) and linear relationship between dietary intake and BMI (indirect effects), as explained elsewhere.
# The RR for BMI and disease were transformed originally reported 5 kg/m^2 to 1 kg/m^2.This transformation was achieved by taking the natural logarithm of the effect estimate, dividing it by 5, and the exponentiating it to obtain the final RR. This methodology was employed to calculate the corresponding 95% CI.
‡ Change in BMI. Based on pooled results from 3 separate prospective cohort studies who were free of obesity (BMI ≥30) or chronic diseases and with complete data on weight and lifestyle habits at baseline. Independent relations of SSBs intake with BMI change were assessed in 4-year periods, using linear regression with robust variance and accounting for within-person repeated measures. In the original study, 1 serving/day of SSBs was calculated as the median intake frequencies of individual foods in each category of the food frequency questionnaire were summed to compute average servings/day. In previous studies of our group, this was assumed to be 8oz serving size, hence we assumed a 1 8oz serving size per day (248g).
§ Optimal intake of SSBs was previously calculated using reported methods based on risk (observed levels associated with lowest mortality/morbidity in meta-analyses), feasibility (observed national consumption levels globally) and consistency (with other assessments in major dietary guidelines).
$Ischemic heart disease is also referred as coronary heart disease

BMI, body mass index; CI, confidence interval; g, grams; mL, milliliters; RR, relative risk; SSBs, sugar-sweetened beverages; T2D, type 2 diabetes.

Supplementary Table 9. Grading of Evidence of the Association of SSBs with ischemic heart disease, ischemic stroke, T2D , and weight gain.*

Outcome	Strength	Consistency	Temporality	Coherence	Specificity	Analogy	Plausibility	Biological Gradient	Experiment
Ischemic heart disease$	++	+++	+++	+++	++	++	++	+++	++
Ischemic stroke	++	++	+++	+++	++	++	++	+++	++
T2D	++	+++	+++	+++	+++	+++§	+++	+++	+++
Weight gain	+++	+++	+++	+++	+++	+++§	+++	+++	+++

*Reproduced from evidence grading conducted in by Miller *et al.* in previous analyses. The 9 Bradford-Hill criteria for grading the evidence were strength, consistency, temporality, coherence, specificity, analogy, plausibility, biological gradient, and experiment. To score each Bradford-Hill criterion, the following general principles were utilized, focusing on evidence from meta-analyses of prospective cohort studies and/or randomized controlled trials: +++ Consistent evidence from several well-designed studies with relatively few limitations; ++ Consistent evidence from several studies but with some important limitations; + Emerging evidence from a few studies or conflicting results from several studies; – criterion not met. Definitions for each of the nine criteria and adaptations to the general scoring system were as follows: **Strength:** magnitude of association, including RRs for protective factors of >0.9 (+), 0.8±0.89 (++), or <0.8 (+++); and for harmful factors, of <1.11 (+), 1.25 (++), and >1.25 (+++). Since magnitude is directly dependent on both the selected serving size and frequency of consumption, we utilized serving sizes most similar to standard dietary guidelines and frequencies of consumption representing modest, standardized differences in intake (e.g., 1 serving/d of fruit) that are easily communicated and could be feasibly achieved by an intervention. **Consistency:** association is repeatedly observed in different populations and circumstances, including ≥80% of included study-specific estimates being in the expected direction (+++); ≥60 - <80% (++); ≥40 - <60% (+); and <40% (not meeting criteria). **Temporality:** exposure precedes outcome. Because all evidence was based on longitudinal studies, this was a necessary criterion (+++); when relatively few overall studies were available (<5), we graded this criterion conservatively as ++. **Coherence:** interpretation of association does not conflict with known natural history and biology of the disease, for example based on pathways of disease occurrence and laboratory findings on the dietary factor. **Specificity:** exposure linked to a specific outcome. Because many nutritional factors can plausibly have diverse effects and influence multiple outcomes, scoring was based on three principles: 1) dietary factor influences a mechanism/pathways known to cause the outcome; 2) dietary factor not associated with multiple other, unrelated non-communicable diseases (e.g., multiple cancers, chronic obstructive pulmonary disease); 3) dietary association has additional specificity within the set of cardiometabolic outcomes (coronary heart disease, stroke, T2D mellitus). **Analogy:** based on the effects of similar factors on the disease outcome; see detailed footnotes below. **Plausibility:** association supported by one or more credible biological mechanisms. **Biological gradient:** exposure and outcome are related by a monotonic dose-response curve. **Experiment:** association is also supported by evidence from randomized controlled trials on intermediate risk factors (or, less commonly, disease outcomes) plus supportive laboratory studies.

§ Based on analogies to other poor-quality carbohydrates in relation to both CVD, T2D, and weight gain.

$ Ischemic heart disease is also referred as coronary heart disease

CVD, cardiovascular disease; SSBs, sugar-sweetened beverages; T2D, type 2 diabetes.

Supplementary Table 10. Age at event calculation for the etiologic effect of SSBs on ischemic heart disease and ischemic stroke.

Study	Weighting	Weighting rescaled[†]	Weighting fraction	Age at baseline[¶] (y)	Type of follow-up time	Follow-up time (y)	Mean or 2/3 follow-up time[§]	Age at event[‡] (y)	Weighted age at event[#] (y)
SSBs intake on ischemic heart disease (Yin *et al.*, 2021)									
Eshak *et al.*, 2012	5.76	22.38	0.224	49.5	max follow-up	18	9.0	58.5	13.09
Gardener et al., 2012	2.54	18.38	0.184	68.6	mean follow-up	9.8	6.5	75.1	13.81
Koning *et al.*, 2012	15.11	29.93	0.299	57.5	max follow-up	22	11.0	68.5	20.50
Fung *et al.*, 2009	19.02	29.31	0.293	46.5	max follow-up	24	12.0	58.5	17.15
Total	*42.43*	*100*	*1*	*NA*	*NA*	*NA*	*NA*	*NA*	***64.55***
SSBs intake on ischemic stroke (Bechthold, 2019)									
Bernstein *et al.*, 2012 (HPFS)	5.1	NA	0.051	57.5	max follow-up	22	11.0	68.5	3.5
Bernstein *et al.*, 2012 (NHS)	15.5	NA	0.155	42.5	max follow-up	28	14.0	56.5	8.8
Eshak *et al.*, 2012	5.5	NA	0.055	49.5	max follow-up	18	9.0	58.5	3.2
Gardener *et al.*, 2012	2.3	NA	0.023	68.6	mean follow-up	9.8	6.5	75.1	1.7
Larsson *et al.*, 2014 (Men)	41.1	NA	0.411	62.0	mean follow-up	10.3	6.9	68.9	28.3
Larsson *et al.*, 2014 (Women)	30.2	NA	0.302	66.0	mean follow-up	10.3	6.9	72.9	22.0
Total	*99.7*		*1*						***67.51***

[†] Rescaling of weighting was done when the sum of the weighting was not equal or close to 100. This was the case for the metanalysis of Yin *et al.*, 2021 as the weighting in the metanalysis was for more than one disease.
[¶] Age at age at baseline was calculated as the mean of the range of age at baseline. For example, for Eshak *et al.*, 2012, the range age at baseline was 40 to 59, hence the age at baseline was calculated as (40+59)/2 = 49.5.
[§] The follow-up time was calculated as half of the max follow-up time, or as 2/3 of the mean or median follow-up, depending on the type of follow-up time reported.
[‡] Age at event corresponds to the sum of the age at baseline plus the mean or 2/3 follow-up time.
[#] Age at event was then weighted by its corresponding weighting fraction. The individual weighted age at event where were added up to obtain the final age at event for the metanalysis.
[$] Ischemic heart disease is also referred as coronary heart disease

HPFS, Health Professional Follow-up study; NHS, Nurses' Health Study; SSBs, sugar sweetened beverages; T2D, type 2 diabetes.

Supplementary Table 11. Age at event calculation for the etiologic effect of SSBs on T2D.

Study	Weighting	Weighting rescaled	Weighting fraction	Age at baseline¶ (y)	Type of follow-up time	Follow-up time (y)	Mean or 2/3 follow-up time§	Age at event‡ (y)	Weighted age at event# (y)
SSBs intake on T2D (Qin *et al*., 2020)									
Schulze *et al.*, 2004	4.2	NA	0.042	34.0	max follow-up	8	4	38.0	1.60
Paynter *et al*., 2006	7.24	NA	0.072	54.5	max follow-up	9	4.5	59.0	4.27
Montonen *et al*., 2007	0.46	NA	0.005	54.5	max follow-up	12	6	60.5	0.28
Palmer *et al*., 2008	8.98	NA	0.090	45.0	max follow-up	6	3	48.0	4.31
Odegaard *et al*., 2009	1.66	NA	0.017	59.5	mean follow-up	5.7	3.8	63.3	1.05
Duffey *et al*., 2010	7.43	NA	0.074	24.0	max follow-up	20	10	34.0	2.53
Koning *et al*., 2011	6.63	NA	0.066	57.5	max follow-up	20	10	67.5	4.48
Bhupathiraju *et al*., 2012 (NHS)	8.23	NA	0.082	42.5	max follow-up	24	12	54.5	4.49
Bhupathiraju *et al*., 2012 (HPFS)	7.01	NA	0.070	57.5	max follow-up	22	11	68.5	4.80
Eshak *et al*., 2012	2.75	NA	0.028	49.5	max follow-up	10	5	54.5	1.50
Fagherazzi *et al*., 2013	0.37	NA	0.004	52.6	max follow-up	14	7	59.6	0.22
O'Connor *et al*., 2015	9.13	NA	0.091	59.5	max follow-up	10.8	5.4	64.9	5.93
Ferreira-Pego *et al*., 2016	0.95	NA	0.010	67.5	median follow-up	3.24	2.2	69.7	0.66
Ma *et al*., 2016	2.98	NA	0.030	51.9	median follow-up	14	9.3	61.2	1.82
Huang *et al*., 2017	8.84	NA	0.088	64.5	mean follow-up	8.4	5.6	70.1	6.20
Papier *et al*., 2017	3.29	NA	0.033	28.7	max follow-up	8	4	32.7	1.08
Gardener *et al*., 2018	5.32	NA	0.053	69.0	mean follow-up	11	7.3	76.3	4.06
Hirahatake *et al*., 2019	7.8	NA	0.078	24.0	max follow-up	30	15	39.0	3.04
Stern et. a l, 2019	6.72	NA	0.067	42.1	median follow-up	2.16	1.4	43.5	2.93
	99.99		*1*	*49.4*					***55.23***

¶ Age at age at baseline was calculated as the mean of the range of age at baseline. For example, for Schulze *et al.*, 2004, the range age at baseline was 24 to 44, hence the age at baseline was calculated as (24+44)/2 = 34

§ The follow-up time was calculated as half of the max follow-up time, or as 2/3 of the mean or median follow-up, depending on the type of follow-up time reported.

‡ Age at event corresponds to the sum of the age at baseline plus the mean or 2/3 follow-up time.

# Age at event was then weighted by its corresponding weighting fraction. The individual weighted age at event where were added up to obtain the final age at event for the metanalysis.

HPFS, Health Professional Follow-up study; NHS, Nurses' Health Study; SSBs, sugar-sweetened beverages; T2D, type 2 diabetes.

Supplementary Table 12. Age at event calculation for the etiologic effect of BMI on ischemic stroke and T2D.

Study	Weighting	Weighting rescaled	Weighting fraction	Age at baseline¶ (y)	Type of follow-up time	Follow-up time (y)	Mean or 2/3 follow-up time§	Age at event‡ (y)	Weighted age at event# (y)
BMI on ischemic stroke and T2D (Singh *et al.*, 2013)									
APCSC (Asia Pacific Cohort Studies Collaboration)	NA	NA	0.333	47	mean follow-up	6.9	4.6	51.6	17.20
ERFC (Emerging Risk Factor Collaboration)	NA	NA	0.333	58	mean follow-up	7.9	5.3	63.3	21.09
PSC (Prospective Studies Collaboration)	NA	NA	0.333	52	mean follow-up 8(SD 6)	8	5.3	57.3	19.11
Total			*1*						***57.40***

¶ Age at age at baseline was calculated as the mean of the range of age at baseline. For example, for Eshak et al., 2012, the range age at baseline was 40 to 59, hence the age at baseline was calculated as (40+59)/2 = 49.5.

§ The follow-up time was calculated as half of the max follow-up time, or as 2/3 of the mean or median follow-up, depending on the type of follow-up time reported.

‡ Age at event corresponds to the sum of the age at baseline plus the mean or 2/3 follow-up time.

# Age at event was then weighted by its corresponding weighting fraction. The individual weighted age at event where were added up to obtain the final age at event for the metanalysis.

BMI, body-mass index; SSBs, sugar-sweetened beverages; T2D, type 2 diabetes.

Supplementary Table 13. Effect estimates of the association between education level and area of residence with T2D and CVD used for the stratification of the GBD disease estimates*.

Country income level†	Education Effects‡				Area of residence effects¥		
	RR high-low (95% CI)	*High education*	*Medium education*	*Low education*	RR urban-rural (95% CI)	*Urban*	*Rural*
**Type 2 T2D**							
Low income	1.31 (1.17, 1.47)	*1.146*	*1.000*	*0.872*	1.65 (1.28, 2.12)	*1.285*	*0.778*
Lower-middle income	1.33 (1.27, 1.40)	*1.154*	*1.000*	*0.866*	1.20 (1.10, 1.30)	*1.093*	*0.915*
Upper-middle income	0.89 (0.80, 0.99)	*0.944*	*1.000*	*1.059*	1.31 (1.12, 1.55)	*1.146*	*0.872*
High income	0.71 (0.64; 0.79)	*0.842*	*1.000*	*1.188*	1.07 (1.01, 1.12)	*1.032*	*0.969*
**Cardiovascular Disease**							
Low income	0.44 (0.31, 0.63)	*0.667*	*1.000*	*1.500*	-	-	-
Lower-middle income	0.67 (0.63, 0.72)	*0.819*	*1.000*	*1.222*	-	-	-
Upper-middle income	0.67 (0.63, 0.72)	*0.819*	*1.000*	*1.222*	1.19 (1.10, 1.29)	*1.092*	*0.916*
High income	0.70 (0.60, 0.81)	*0.836*	*1.000*	*1.197*	0.95 (0.93, 0.97)	*0.973*	*1.028*

*We identified meta-analyses, pooled analyses, and large surveys evaluating the association between sociodemographic factors such as educational attainment and urbanicity with CVD and T2D risk. We limited our analysis to high quality risk assessments adjusted only for age and sex, when possible, to avoid the attenuating effects of adjusting for additional covariates. We conducted independent fixed effects meta-analysis of collated effect sizes stratified by country income level to summarize the effect of education and area of residence in CVD and T2D separately. Given inconsistent definitions across studies and limited data availability, medium education attainment was assumed to be neutral (i.e., RR = 1). We distributed the central estimate of our meta-analyzed risk estimate for high versus. low education, and urban versus rural residence, by taking the square root and inverse square root of the central estimate of the relative risk. This, allowed to assume equidistance from the high education to the medium education level, and from medium education to low education level. We tested distributing the central estimate by incorporating information on the actual distance from high to medium education and medium to low education, when available. As the effects did not differ, we used the square root and inverse square root to keep consistency across studies. Further details of the studies used for the stratification of GBD estimates by education level and area of residence available in **Supplementary Data 5 and 6, respectively.**

¥ Estimates for T2D and area of residence slightly differ from previous analyses given the exclusion of the estimate for UMIC from Dagenais *et al.*, 2016 as we later found it was already included in the pooled analysis from den Braver *et al.*, 2018.

†Based on World Bank Country Income group classification

‡Education level was categorized following the GDD definition as "low" 0 to 6 years of education, "medium" >6 years to 12 years of education, and "high" >12 years of education.

CVD, cardiovascular disease; GDD, Global Dietary Database; RR, relative risk; T2D, type 2 diabetes.

Supplementary Table 14. T2D burden disaggregation for a mock, a single national level age-sex T2D incidence estimate (low-income country) into six education level, urbanicity stratified estimates

Educational level†	Urbanicity	Total stratum T2D burden estimate	Mock population proportion	*T2D burden weighted by population proportion*	Education effect	Urban effect	Raw, fully proportioned burden estimate*	Scaled, fully proportioned burden estimate¶
Low	Urban	1000	0.2	*200*	0.82	1.29	212	229.9
Medium	Urban	1000	0.1	*100*	1.00	1.29	129	140.2
High	Urban	1000	0.1	*100*	1.23	1.29	159	172.5
Low	Rural	1000	0.4	*400*	0.82	0.78	256	278.1
Medium	Rural	1000	0.15	*150*	1.00	0.78	117	127.2
High	Rural	1000	0.05	*50*	1.23	0.78	48	52.1
	Total:		*1*	*1000*			*920*	1000

† Education level was defined based on GDD as: low, 0 to 6 years of education; medium, >6 to 12 years of education; and high, >12 years of education.
* The total year-country-age-sex stratum-specific T2D incidence estimates were then multiplied by their respective population proportion, education level effect, and urbanicity effect for each of the six*, de-novo* strata to obtain the raw fully proportioned burden estimates.
¶ These values were then scaled to the total stratum T2D burden estimate to prevent under- or overestimation of the absolute number of T2D cases globally as: Total T2D burden weighted by population proportion* Stratum raw fully proportionated estimate burden / Total raw fully proportioned burden estimate (e.g., 1000*212/920 = 229.9).

T2D, type 2 diabetes.

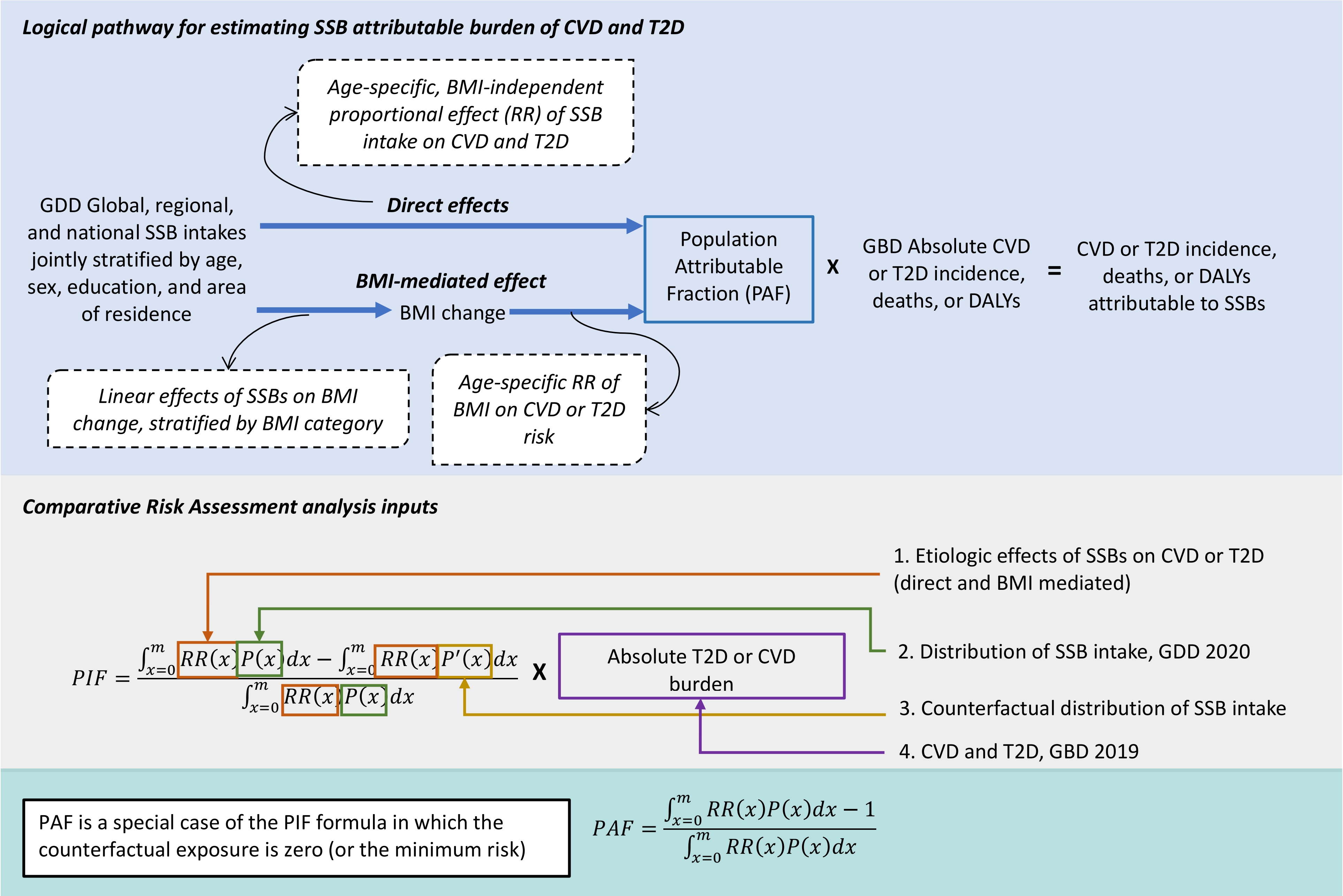

Supplementary Figure 18. **Pathway for estimating the direct and BMI-mediated CVD and T2D burden attributable to intake of SSBs and list of CRA analysis inputs.** Dotted boxes suggest the logical pathway through which SSBs impact CVD, T2D, or BMI.

CVD, cardiovascular disease; CRA, comparative risk assessment; BMI, body mass index; GBD, Global Burden of Disease; GDD, Global Dietary Database; NCD-RisC, NCD Risk Factor Collaboration; PAF, population attributable fraction; RR, relative risk. T2D, type 2 diabetes. Adapted from O'Hearn, 2023.
