# Supplementary material for: Burdens of type 2 diabetes and cardiovascular disease attributable to sugar-sweetened beverages in 184 countries
Source: Nat Med. 2025 Jan 6;31(2):552–64. doi: 10.1038/s41591-024-03345-4 (PMC11835746; doi:10.1038/s41591-024-03345-4)
Supplement: Supplementary file 2 — Reporting Summary [file 41591_2024_3345_MOESM2_ESM.pdf]

nature portfolio

Corresponding author(s): Laura Lara-Castor

Last updated by author(s): 10/03/2024

Reporting Summary

Nature Portfolio wishes to improve the reproducibility of the work that we publish. This form provides structure for consistency and transparency in reporting. For further information on Nature Portfolio policies, see our Editorial Policies and the Editorial Policy Checklist.

Statistics

For all statistical analyses, confirm that the following items are present in the figure legend, table legend, main text, or Methods section.

n/a	Confirmed	
☐	☒	The exact sample size (*n*) for each experimental group/condition, given as a discrete number and unit of measurement
☒	☐	A statement on whether measurements were taken from distinct samples or whether the same sample was measured repeatedly
☒	☐	The statistical test(s) used AND whether they are one- or two-sided<br>*Only common tests should be described solely by name; describe more complex techniques in the Methods section.*
☒	☐	A description of all covariates tested
☐	☒	A description of any assumptions or corrections, such as tests of normality and adjustment for multiple comparisons
☐	☒	A full description of the statistical parameters including central tendency (e.g. means) or other basic estimates (e.g. regression coefficient) AND variation (e.g. standard deviation) or associated estimates of uncertainty (e.g. confidence intervals)
☒	☐	For null hypothesis testing, the test statistic (e.g. *F*, *t*, *r*) with confidence intervals, effect sizes, degrees of freedom and *P* value noted<br>*Give P values as exact values whenever suitable.*
☐	☒	For Bayesian analysis, information on the choice of priors and Markov chain Monte Carlo settings
☒	☐	For hierarchical and complex designs, identification of the appropriate level for tests and full reporting of outcomes
☒	☐	Estimates of effect sizes (e.g. Cohen's *d*, Pearson's *r*), indicating how they were calculated

Our web collection on statistics for biologists contains articles on many of the points above.

Software and code

Policy information about availability of computer code

Data collection	No software was used for data collection for this analysis.
Data analysis	Custom code was developed using R (Version 4.4.0) for this research including, calculation of age-adjusted relative risks, SSB intake gamma parameters, comparative risk assessment analysis (including the PAF and attributable mortality for each stratum), and data visualizations. Packages used include rworldmap package (v1.3-6). Given the computational size, calculation of the SSB intake gamma parameters, comparative risk assessment modeling, absolute and relative differences, and summary statistics were run on the Tufts University High Performance Computing Cluster (https://it.tufts.edu/high-performance-computing), supported by the National Science Foundation (grant:2018149, https://www.nsf.gov/awardsearch/showAward?AWD_ID=2018149&HistoricalAwards=false) under active development by Research Technology, Tufts Technology Services (https://it.tufts.edu/researchtechnology.tufts.edu).<br><br>The statistical code can be made available to researchers upon request. Eligibility criteria for such requests include utilization for nonprofit purposes only, for appropriate scientific use based on a robust research plan, and by investigators from an academic institution. GDD will nominate co-authors to be included on any papers generated using GDD-generated statistical code. If you are interested in requesting access to the statistical code, please submit the following documents: (1) proposed research plan (please download and complete the proposed research plan form: https://www.globaldietarydatabase.org/sites/default/files/manual_upload/research-proposal-template.pdf), (2) data-sharing agreement (please download this form https://www.globaldietarydatabase.org/sites/default/files/manual_upload/tufts-gdd-data-sharing-agreement.docx and complete the highlighted fields, have someone who is authorized to enter your institution into a binding legal agreement with outside institutions sign the document. Note that this agreement does not apply when protected health information or personally identifiable information are shared), (3) email items (1) and (2) to info@globaldietarydatabase.org.

Please use the subject line "GDD Code Access Request". Once all documents have been received, the GDD team will be in contact with you within 2-4 weeks regarding subsequent steps. Data will be shared as .csv or .xlsx files, using a compressed format when appropriate.

For manuscripts utilizing custom algorithms or software that are central to the research but not yet described in published literature, software must be made available to editors and reviewers. We strongly encourage code deposition in a community repository (e.g. GitHub). See the Nature Portfolio guidelines for submitting code & software for further information.

Data

Policy information about availability of data

All manuscripts must include a data availability statement. This statement should provide the following information, where applicable:

- Accession codes, unique identifiers, or web links for publicly available datasets
- A description of any restrictions on data availability
- For clinical datasets or third party data, please ensure that the statement adheres to our policy

Data used in this analysis are publicly available from the following sources: (1) population SSB intake distributions based on individual-level survey data from the GDD (https://www.globaldietarydatabase.org/data-download); (2) optimal SSB intake levels from previous analyses;68 (3) direct age-adjusted etiologic effects of SSBs on diabetes, ischemic heart disease, and ischemic stroke adjusted for BMI, and of weight gain on diabetes, ischemic heart disease, and ischemic stroke from previous meta-analyses and pooled analyses of prospective cohorts; as well as linear, BMI-stratified effects of SSBs on weight gain or loss; (4) population overweight (BMI ≥25 kg/m2) and underweight (BMI <18.5 kg/m2) distributions from the NCD Risk Factor Collaboration (NCD-RisC: https://ncdrisc.org/data-downloads.html); (5) total diabetes, ischemic heart disease, and ischemic stroke incidence, DALYs, and mortality estimate distributions from the GBD study (https://vizhub.healthdata.org/gbd-results/); and (6) population demographic data from the United Nations Population Division (UN, https://population.un.org/wpp/), the Barro and Lee Educational Attainment Dataset 2013 (Barro and Lee, DOI: 10.3386/w15902), and Socio-demographic Index (SDI) data (Global Health Data Exchange: GBD, https://ghdx.healthdata.org/record/ihme-data/gbd-2019-socio-demographic-index-sdi-1950-2019).

The GDD SSB intake data was collapsed for 85+ years using the 4,000 simulations corresponding to the stratum level intake data derived from the Bayesian model. These data were used to obtain the gamma parameters of the SSB intake distribution used in the model. The 4,000 simulations files can be made available to researchers upon request. Eligibility criteria for such requests include utilization for nonprofit purposes only, for appropriate scientific use based on a robust research plan, and by investigators from an academic institution. If you are interested in requesting access to the data, please submit the following documents: (1) proposed research plan (please download and complete the proposed research plan form: https://www.globaldietarydatabase.org/sites/default/files/manual_upload/research-proposal-template.pdf), (2) data-sharing agreement (please download this form https://www.globaldietarydatabase.org/sites/default/files/manual_upload/tufts-gdd-data-sharing-agreement.docx and complete the highlighted fields, have someone who is authorized to enter your institution into a binding legal agreement with outside institutions sign the document. Note that this agreement does not apply when protected health information or personally identifiable information are shared), (3) email items (1) and (2) to info@globaldietarydatabase.org.

Please use the subject line "GDD Data Access Request". Once all documents have been received, the GDD team will be in contact with you within 2-4 weeks regarding subsequent steps. Data will be shared as .csv or .xlsx files, using a compressed format when appropriate.

Research involving human participants, their data, or biological material

Policy information about studies with human participants or human data. See also policy information about sex, gender (identity/presentation), and sexual orientation and race, ethnicity and racism.

Reporting on sex and gender	This study involved secondary data analysis from publicly available databases. No human research participants were involved in this original analysis. Sex was defined as female or male and this was extracted from the individual surveys as provided. No distinction between sex vs. gender was possible for this study.
Reporting on race, ethnicity, or other socially relevant groupings	This analysis is based in multiple inputs from published de-identified studies. The population characteristics for each study are available within each study publication/report of each input. In our findings we report estimates stratified by socially relevant groups including education level and area of residence.
Population characteristics	This analysis is based in multiple inputs from published de-identified studies. The population characteristics for each study are available within each study publication/report of each input.
Recruitment	The recruitment methodology for each study is available within each study publication/report of each input.
Ethics oversight	This investigation was exempt from ethical review board approval because it was based on published de-identified nationally representative data, without personally identifiable information.

Note that full information on the approval of the study protocol must also be provided in the manuscript.

Field-specific reporting

Please select the one below that is the best fit for your research. If you are not sure, read the appropriate sections before making your selection.

☐ Life sciences ☒ Behavioural & social sciences ☐ Ecological, evolutionary & environmental sciences

For a reference copy of the document with all sections, see nature.com/documents/nr-reporting-summary-flat.pdf

Behavioural & social sciences study design

All studies must disclose on these points even when the disclosure is negative.

Study description	Quantitative, comparative risk assessment (CRA) modeling analysis incorporating independently derived inputs and parameters on demographics, risk factors, their etiologic effects, and disease burdens to model attributable SSB cardiometabolic burdens at the global, regional, and national levels, and by key sociodemographic factors.
Research sample	This study involved a secondary data analysis from publicly available databases. The model estimated SSB-attributable cardiometabolic disease burdens for the global adult population (20+ years) stratified by sex (men and women), age (14 age groups), educational attainment (low, medium, high), and urbanicity (rural or urban), yielding 30,912 population strata across 184 countries in each year (1990 and 2020). No distinction between sex vs. gender was possible for this study. Sex was defined as female or male and this was extracted from the datasets as provided.
Sampling strategy	Given the nature of the study, using independently derived datasets, the sampling procedures varied across datasets including: dietary intake surveys, T2D incidence data, BMI surveys, estimated etiologic effects.
Data collection	This study consisted on a secondary data analysis, thus no individual-level data was collected. Data used in this analysis are publicly available from the following sources: (1) population SSB intake distributions based on individual-level survey data from the GDD (https://www.globaldietarydatabase.org/data-download); (2) optimal SSB intake levels from previous analyses;68 (3) direct age-adjusted etiologic effects of SSBs on diabetes, ischemic heart disease, and ischemic stroke adjusted for BMI, and of weight gain on diabetes, ischemic heart disease, and ischemic stroke from previous meta-analyses and pooled analyses of prospective cohorts; as well as linear, BMI-stratified effects of SSBs on weight gain or loss; (4) population overweight (BMI ≥25 kg/m2) and underweight (BMI <18.5 kg/m2) distributions from the NCD Risk Factor Collaboration (NCD-RisC: https://ncdrisc.org/data-downloads.html); (5) total diabetes, ischemic heart disease, and ischemic stroke incidence, DALYs, and mortality estimate distributions from the GBD study (https://vizhub.healthdata.org/gbd-results/); and (6) population demographic data from the United Nations Population Division (UN, https://population.un.org/wpp/), the Barro and Lee Educational Attainment Dataset 2013 (Barro and Lee, DOI: 10.3386/w15902), and Socio-demographic Index (SDI) data (Global Health Data Exchange: GBD, https://ghdx.healthdata.org/record/ihme-data/gbd-2019-socio-demographic-index-sdi-1950-2019).<br><br>Our primary dietary input, SSB intakes from the GDD, is unique to this study and was modeled by our team. The GDD systematically searched for and compiled representative data on individual-level dietary intakes from national surveys and sub-national surveys. The final GDD model incorporated 1,224 dietary surveys representing 185 countries from seven world regions and 99.0% of the global population in 2020. Of these, 450 surveys reported data on SSBs, totaling 2.9 million individuals from 118 countries representing 86.8% of the global population. Most surveys were nationally or sub-nationally representative (94.2%), collected at the individual level (84.7%), and included estimates in, both urban and rural area of residence (61.6%). The global sample size consisted of 2,941,704 participants: 44.3% female and 55.7% male; 70% urban areas and 30% from rural areas; 16% low, 37.6% medium, and 46.4% high education; and 53% adults and 47% children/adolescent. Further details on characteristics of surveys with data on SSBs, including availability of surveys per world region, is available in Supplementary Table 1.
Timing	The estimates reported in this study correspond to years 1990 and 2020.
Data exclusions	Of 188 countries with dietary survey data, three were dropped from the GDD estimation model due to unavailability of FAO food availability data (Andorra, Democratic People's Republic of Korea, and Somalia); and 1 additional country (South Sudan) was excluded as it lacked overweight/obesity estimates from the NCD Risk Factor Collaboration study.
Non-participation	No human research participants were involved in this original analysis.
Randomization	This study is a comparative risk assessment analysis and thus not randomization is not applicable

Reporting for specific materials, systems and methods

We require information from authors about some types of materials, experimental systems and methods used in many studies. Here, indicate whether each material, system or method listed is relevant to your study. If you are not sure if a list item applies to your research, read the appropriate section before selecting a response.

Materials & experimental systems

n/a	Involved in the study	
☒	☐	Antibodies
☒	☐	Eukaryotic cell lines
☒	☐	Palaeontology and archaeology
☒	☐	Animals and other organisms
☒	☐	Clinical data
☒	☐	Dual use research of concern
☒	☐	Plants

Methods

n/a	Involved in the study	
☒	☐	ChIP-seq
☒	☐	Flow cytometry
☒	☐	MRI-based neuroimaging
