# Supplementary material for: Burdens of type 2 diabetes and cardiovascular disease attributable to sugar-sweetened beverages in 184 countries
Source: Nat Med. 2025 Jan 6;31(2):552–64. doi: 10.1038/s41591-024-03345-4 (PMC11835746; doi:10.1038/s41591-024-03345-4)
Supplement: Supplementary file 3 — Proportional and absolute T2D and CVD burdens attributable to SSBs in 1990 and 2020 globally, regionally and nationally. [file 41591_2024_3345_MOESM3_ESM.pdf]

Burdens of type 2 diabetes and cardiovascular disease burdens to sugar-sweetened beverages in 184 countries

Supplementary Data 1 | Proportional and absolute T2D and CVD burdens attributable to SSBs in 1990 and 2020 globally, regionally, and nationally

Supplementary Data 4. Proportional and absolute T2D and CVD burdens attributable to SSBs in 1990 and 2020 globally, regionally, and nationally.

		Proportional burden % §		Absolute burden §		Absolute burden per 1M population §, ƒ	
Location	Outcome	1990	2020	1990	2020	1990	2020
World	T2D Incidence	8.5 (8.0-9.1)	9.8 (9.1-10.5)	599917 (564278-643483)	2158238 (2008314-2318849)	198 (186-212)	418 (389-449)
	CVD Incidence	3.2 (3.0-3.5)	3.1 (2.8-3.4)	633677 (587074-692613)	1189005 (1080582-1305312)	209 (193-228)	230 (209-253)
	T2D DALYs	6.3 (5.9-6.8)	6.9 (6.4-7.4)	1554698 (1453959-1663292)	4979168 (4572262-5360995)	512 (479-548)	965 (886-1039)
	CVD DALYs	3.2 (3.0-3.5)	3.0 (2.7-3.3)	5206832 (4857773-5622155)	7556681 (6872717-8264576)	1715 (1600-1852)	1464 (1332-1601)
	T2D Deaths	4.9 (4.6-5.4)	5.1 (4.6-5.7)	30757 (28375-33416)	80278 (72298-88825)	10.1 (9.3-11.0)	15.6 (14.0-17.2)
	CVD Deaths	2.4 (2.2-2.6)	2.1 (1.9-2.3)	180525 (167598-196616)	257961 (235059-283708)	59.5 (55.2-64.8)	50.0 (45.5-55.0)
Word region							
Centr/Eastern Europe and Centr Asia†	T2D Incidence	5.5 (4.7-6.5)	7.0 (5.9-8.5)	31094 (26620-36900)	83449 (70404-101281)	112 (95.8-133)	266 (225-323)
	CVD Incidence	1.9 (1.6-2.3)	2.4 (2.0-3.1)	67985 (57795-83879)	109322 (91102-140562)	245 (208-302)	349 (291-449)
	T2D DALYs	3.5 (3.0-4.2)	4.0 (3.4-5.0)	58583 (50663-70571)	161486 (136692-203447)	211 (182-254)	515 (436-649)
	CVD DALYs	1.9 (1.7-2.4)	2.2 (1.9-2.8)	720560 (615284-889132)	771656 (650105-985109)	2594 (2215-3201)	2463 (2075-3144)
	T2D Deaths	2.7 (2.3-3.3)	2.7 (2.2-3.5)	856 (723-1046)	2332 (1917-3010)	3.1 (2.6-3.8)	7.4 (6.1-9.6)
	CVD Deaths	1.4 (1.2-1.7)	1.6 (1.3-2.0)	26514 (22358-32929)	30858 (25597-39644)	95.5 (80.5-119)	98.5 (81.7-127)
High-Income Countries	T2D Incidence	13.4 (12.2-15.9)	13.2 (12.0-16.2)	159062 (144574-190429)	451454 (407755-558029)	321 (292-385)	707 (638-874)
	CVD Incidence	4.7 (4.3-5.6)	3.8 (3.5-4.5)	202021 (184028-243570)	141530 (129782-168424)	408 (372-492)	222 (203-264)
	T2D DALYs	7.2 (6.6-8.6)	7.8 (7.2-10.0)	301134 (277331-362342)	691616 (630770-877072)	608 (560-732)	1083 (987-1373)
	CVD DALYs	4.4 (4.0-5.2)	4.0 (3.7-5.2)	1568574 (1440152-1879375)	947477 (862515-1219794)	3168 (2908-3795)	1483 (1350-1909)
	T2D Deaths	4.4 (4.0-5.2)	4.1 (3.8-5.4)	5852 (5401-6981)	6891 (6329-9130)	11.8 (10.9-14.1)	10.8 (9.9-14.3)
	CVD Deaths	3.0 (2.7-3.6)	2.5 (2.3-3.1)	60682 (55965-72883)	36354 (33363-45398)	123 (113-147)	56.9 (52.2-71.1)
Latin Amer/Caribbean	T2D Incidence	27.6 (25.3-30.0)	24.4 (22.2-26.9)	201223 (184517-220198)	556705 (505038-616752)	862 (790-943)	1263 (1146-1400)
	CVD Incidence	12.5 (11.2-13.8)	11.3 (10.1-12.8)	121084 (108761-134341)	230175 (205066-261490)	519 (466-575)	522 (465-593)
	T2D DALYs	19.5 (17.8-21.5)	16.9 (15.2-19.0)	613276 (561148-677908)	1526144 (1376506-1723419)	2627 (2404-2904)	3463 (3124-3911)
	CVD DALYs	12.0 (10.7-13.4)	10.4 (9.2-11.8)	1128340 (1006767-1258213)	1489719 (1323356-1686540)	4833 (4312-5389)	3381 (3003-3827)

Supplementary Data 4. Proportional and absolute T2D and CVD burdens attributable to SSBs in 1990 and 2020 globally, regionally, and nationally (continued).

		Proportional burden % §		Absolute burden §		Absolute burden per 1M population §, ƒ	
Location	Outcome	1990	2020	1990	2020	1990	2020
Mid. East/North Africa	T2D Deaths	14.6 (13.1-16.4)	12.0 (10.6-13.8)	12286 (11025-13805)	26627 (23506-30759)	52.6 (47.2-59.1)	60.4 (53.3-69.8)
	CVD Deaths	8.8 (7.7-9.9)	7.5 (6.6-8.7)	37774 (33157-42583)	52641 (46121-60879)	162 (142-182)	119 (105-138)
	T2D Incidence	14.1 (12.0-16.1)	15.0 (12.6-17.3)	51594 (44053-59282)	345790 (290801-401372)	350 (299-402)	1001 (841-1161)
	CVD Incidence	6.7 (5.5-8.0)	6.9 (5.7-8.3)	107896 (88516-129118)	281762 (232813-338630)	732 (601-876)	815 (674-980)
	T2D DALYs	9.5 (8.0-11.1)	10.0 (8.4-11.7)	116407 (97557-136529)	562030 (471824-659650)	790 (662-927)	1626 (1365-1909)
	CVD DALYs	6.9 (5.7-8.2)	6.8 (5.6-8.2)	760078 (625969-905438)	1413739 (1163755-1715848)	5159 (4248-6145)	4090 (3367-4965)
South Asia†	T2D Deaths	7.2 (5.9-8.6)	6.4 (5.3-7.8)	2268 (1870-2740)	6862 (5685-8340)	15.4 (12.7-18.6)	19.9 (16.4-24.1)
	CVD Deaths	5.4 (4.4-6.4)	5.1 (4.1-6.2)	24501 (20077-29571)	47126 (38125-57643)	166 (136-201)	136 (110-167)
	T2D Incidence	2.6 (1.7-3.8)	3.7 (2.6-5.3)	31458 (21613-46151)	174141 (121624-249954)	54.5 (37.4-80.0)	149 (104-213)
	CVD Incidence	0.9 (0.6-1.5)	1.2 (0.7-1.9)	35110 (21228-57660)	105345 (66757-170331)	60.8 (36.8-99.9)	89.9 (57.0-145)
	T2D DALYs	1.9 (1.2-2.9)	2.4 (1.6-3.5)	86672 (56371-131786)	400335 (269653-590095)	150 (97.7-228)	342 (230-504)
	CVD DALYs	0.9 (0.6-1.5)	1.3 (0.8-2.0)	234933 (150385-376504)	765481 (495643-1195890)	407 (261-652)	654 (423-1021)
Southeast and East Asia	T2D Deaths	1.5 (0.9-2.3)	1.5 (1.0-2.4)	1711 (1074-2673)	6462 (4147-10043)	3.0 (1.9-4.6)	5.5 (3.5-8.6)
	CVD Deaths	0.8 (0.5-1.2)	0.9 (0.6-1.5)	7155 (4532-11467)	22988 (14609-36191)	12.4 (7.9-19.9)	19.6 (12.5-30.9)
	T2D Incidence	2.7 (2.3-3.6)	3.1 (2.7-3.8)	68772 (60514-91805)	204091 (177885-248905)	63.5 (55.8-84.7)	119 (103-145)
	CVD Incidence	0.7 (0.7-0.9)	0.6 (0.6-0.8)	31379 (27877-39587)	80602 (70517-98202)	29.0 (25.7-36.5)	46.8 (41.0-57.1)
	T2D DALYs	1.8 (1.6-2.1)	2.1 (1.9-2.6)	128922 (115733-154190)	448068 (389905-551487)	119 (107-142)	260 (227-321)
	CVD DALYs	0.9 (0.8-1.1)	0.9 (0.8-1.1)	328631 (293254-397865)	731868 (639732-882808)	303 (271-367)	425 (372-513)
Sub-Saharan Africa	T2D Deaths	1.3 (1.1-1.5)	1.7 (1.5-2.2)	1905 (1712-2244)	6502 (5535-8157)	1.8 (1.6-2.1)	3.8 (3.2-4.7)
	CVD Deaths	0.6 (0.5-0.7)	0.5 (0.5-0.6)	9520 (8567-11246)	22265 (19581-26455)	8.8 (7.9-10.4)	12.9 (11.4-15.4)
	T2D Incidence	12.7 (10.5-15.1)	21.5 (17.6-26.0)	47831 (39705-56884)	312721 (256225-377773)	216 (180-257)	589 (483-712)
	CVD Incidence	6.1 (4.8-7.7)	10.5 (8.1-13.3)	62602 (49181-78498)	228979 (176841-289189)	283 (222-355)	432 (333-545)
	T2D DALYs	9.7 (7.9-12.0)	16.9 (13.4-20.9)	230675 (187465-283545)	1117722 (884731-1383622)	1043 (848-1282)	2106 (1667-2608)
	CVD DALYs	6.1 (4.9-7.7)	9.8 (7.5-12.5)	422713 (332816-532091)	1349061 (1043299-1724982)	1912 (1505-2407)	2542 (1966-3251)
	T2D Deaths	7.5 (5.9-9.6)	13.0 (10.0-16.8)	5524 (4334-7073)	23623 (18046-30566)	25.0 (19.6-32.0)	44.5 (34.0-57.6)
	CVD Deaths	4.6 (3.5-5.9)	7.4 (5.5-9.9)	12959 (9807-16838)	42946 (31784-57085)	58.6 (44.4-76.2)	80.9 (59.9-108)
Country							
Afghanistan	T2D Incidence	0.5 (0.2-1.7)	8.8 (4.4-17.0)	107 (48.3-326)	9942 (4995-19045)	21.0 (9.4-63.9)	552 (277-1057)

Supplementary Data 4. Proportional and absolute T2D and CVD burdens attributable to SSBs in 1990 and 2020 globally, regionally, and nationally (continued).

		Proportional burden % §		Absolute burden §		Absolute burden per 1M population §, ƒ	
Location	Outcome	1990	2020	1990	2020	1990	2020
	CVD Incidence	0.2 (0.1-0.5)	3.3 (1.5-7.1)	180 (83.7-431)	3474 (1588-7555)	35.2 (16.4-84.5)	193 (88.1-419)
	T2D DALYs	0.4 (0.2-1.0)	6.4 (3.1-12.8)	280 (131-712)	14434 (7015-28826)	54.8 (25.7-139)	801 (389-1600)
	CVD DALYs	0.2 (0.1-0.5)	3.3 (1.6-7.3)	1578 (736-3910)	27581 (12833-61132)	309 (144-766)	1531 (712-3393)
	T2D Deaths	0.3 (0.1-0.8)	4.4 (2.1-9.6)	5.2 (2.4-12.6)	145 (67.3-315)	1.0 (0.5-2.5)	8.1 (3.7-17.5)
	CVD Deaths	0.2 (0.1-0.4)	2.5 (1.1-5.4)	51.9 (23.4-126)	769 (361-1684)	10.2 (4.6-24.6)	42.7 (20.0-93.5)
Albania	T2D Incidence	4.5 (3.0-7.3)	51.9 (42.6-60.6)	146 (97.6-235)	3405 (2790-3987)	77.2 (51.6-124)	1560 (1279-1827)
	CVD Incidence	2.0 (1.3-3.3)	26.5 (19.7-33.9)	162 (108-268)	4268 (3186-5569)	86.0 (57.2-142)	1956 (1460-2552)
	T2D DALYs	2.7 (1.8-4.6)	39.1 (30.2-48.1)	168 (112-290)	6335 (4911-7850)	89.1 (59.4-154)	2903 (2250-3597)
	CVD DALYs	1.7 (1.1-2.8)	23.2 (17.1-29.9)	1286 (863-2160)	31210 (22910-40626)	681 (457-1144)	14302 (10499-18617)
	T2D Deaths	1.7 (1.1-3.0)	27.8 (20.3-36.7)	1.4 (1.0-2.6)	55.3 (39.8-73.7)	0.8 (0.5-1.4)	25.4 (18.3-33.8)
	CVD Deaths	1.2 (0.8-2.0)	18.1 (13.1-24.4)	44.5 (30.3-74.8)	1473 (1054-1998)	23.5 (16.0-39.6)	675 (483-916)
Algeria	T2D Incidence	19.2 (13.1-27.7)	19.6 (13.3-28.5)	5982 (4050-8700)	37770 (25507-54441)	511 (346-742)	1376 (929-1984)
	CVD Incidence	9.6 (6.2-14.6)	9.6 (6.2-14.6)	13344 (8641-20288)	31863 (20324-48095)	1139 (737-1731)	1161 (741-1753)
	T2D DALYs	14.2 (9.5-20.9)	14.5 (9.5-21.3)	9385 (6300-13756)	57488 (37520-84027)	801 (538-1174)	2095 (1367-3062)
	CVD DALYs	10.1 (6.8-15.1)	8.6 (5.6-12.8)	75177 (50145-112244)	121917 (79221-181418)	6416 (4280-9579)	4443 (2887-6611)
	T2D Deaths	10.6 (6.9-16.2)	9.8 (6.3-14.7)	116 (76.1-177)	502 (326-763)	9.9 (6.5-15.1)	18.3 (11.9-27.8)
	CVD Deaths	7.8 (5.1-11.9)	6.3 (4.1-9.5)	2516 (1642-3840)	4468 (2902-6754)	215 (140-328)	163 (106-246)
Angola	T2D Incidence	13.8 (9.6-20.2)	26.6 (18.6-36.1)	1362 (936-1971)	13389 (9413-18239)	275 (189-398)	951 (668-1295)
	CVD Incidence	6.7 (4.4-10.4)	12.8 (8.3-18.7)	1240 (808-1931)	6283 (4108-9233)	251 (163-390)	446 (292-656)
	T2D DALYs	11.0 (7.2-16.4)	20.7 (14.2-28.9)	6436 (4254-9653)	42172 (28640-58589)	1301 (860-1951)	2994 (2033-4160)
	CVD DALYs	6.5 (4.2-10.3)	11.9 (7.8-17.5)	9060 (5799-14414)	44560 (29004-66361)	1831 (1172-2914)	3164 (2059-4711)
	T2D Deaths	8.9 (5.7-13.9)	15.9 (10.4-23.2)	141 (89.9-217)	720 (479-1036)	28.5 (18.2-43.8)	51.2 (34.0-73.6)
	CVD Deaths	5.3 (3.4-8.8)	9.5 (6.0-14.5)	278 (177-464)	1399 (868-2155)	56.3 (35.7-93.8)	99.3 (61.6-153)
Antigua and Barbuda	T2D Incidence	27.3 (19.0-37.7)	30.9 (21.6-42.5)	44.4 (31.3-61.9)	173 (123-240)	1179 (829-1642)	2497 (1775-3456)
	CVD Incidence	11.9 (7.9-17.3)	15.6 (10.6-23.0)	29.3 (19.6-43.2)	71.1 (47.2-103)	779 (520-1146)	1023 (679-1487)
	T2D DALYs	17.1 (11.5-24.6)	21.5 (14.8-30.6)	174 (117-250)	462 (315-661)	4617 (3107-6626)	6653 (4539-9517)
	CVD DALYs	10.7 (7.2-15.8)	12.2 (8.2-17.8)	203 (136-299)	218 (147-316)	5384 (3617-7942)	3131 (2119-4553)

Supplementary Data 4. Proportional and absolute T2D and CVD burdens attributable to SSBs in 1990 and 2020 globally, regionally, and nationally (continued).

		Proportional burden % §		Absolute burden §		Absolute burden per 1M population §, ƒ	
Location	Outcome	1990	2020	1990	2020	1990	2020
Argentina	T2D Deaths	12.5 (8.3-18.5)	15.2 (10.2-22.1)	4.5 (3.0-6.7)	8.0 (5.3-11.6)	121 (79.1-179)	115 (75.9-167)
	CVD Deaths	7.6 (5.1-11.5)	8.8 (5.8-13.2)	8.1 (5.4-12.1)	8.5 (5.6-12.6)	215 (142-322)	122 (79.9-181)
	T2D Incidence	12.2 (9.6-16.2)	18.8 (15.0-24.2)	6473 (5046-8566)	26742 (21381-34101)	328 (255-433)	874 (698-1114)
	CVD Incidence	5.5 (4.3-7.9)	8.3 (6.6-11.5)	7660 (5984-10941)	13453 (10527-18604)	388 (303-554)	439 (344-608)
	T2D DALYs	7.9 (6.3-11.1)	12.1 (9.7-16.2)	17285 (13775-24099)	49554 (39986-66302)	875 (697-1219)	1619 (1306-2166)
	CVD DALYs	5.6 (4.4-7.9)	7.6 (6.0-10.5)	68763 (54011-97417)	70138 (55717-97054)	3479 (2733-4929)	2291 (1820-3170)
Armenia	T2D Deaths	6.1 (4.9-8.9)	8.7 (7.0-12.3)	433 (349-634)	850 (679-1191)	21.9 (17.7-32.1)	27.8 (22.2-38.9)
	CVD Deaths	4.0 (3.2-5.8)	5.4 (4.3-7.5)	2518 (1992-3614)	2676 (2133-3750)	127 (101-183)	87.4 (69.7-123)
	T2D Incidence	1.8 (1.2-3.7)	5.5 (3.4-8.9)	89.2 (55.5-180)	474 (306-776)	40.6 (25.3-82.1)	218 (141-357)
	CVD Incidence	0.7 (0.4-1.2)	1.9 (1.2-3.4)	134 (86.4-234)	529 (344-955)	60.9 (39.3-107)	243 (158-439)
	T2D DALYs	1.1 (0.7-2.0)	3.2 (2.1-5.6)	209 (132-371)	950 (613-1703)	95.2 (60.3-169)	437 (282-783)
	CVD DALYs	0.7 (0.5-1.4)	1.8 (1.2-3.2)	1295 (825-2449)	3641 (2382-6560)	590 (376-1115)	1674 (1095-3015)
Australia	T2D Deaths	0.9 (0.6-1.5)	2.2 (1.4-4.1)	3.9 (2.5-6.8)	13.8 (9.0-25.0)	1.8 (1.2-3.1)	6.3 (4.1-11.5)
	CVD Deaths	0.5 (0.3-0.9)	1.3 (0.8-2.3)	44.9 (28.9-78.6)	145 (94.1-262)	20.4 (13.2-35.8)	66.8 (43.3-121)
	T2D Incidence	13.9 (10.9-18.5)	9.9 (7.8-14.6)	2729 (2126-3611)	6013 (4675-8865)	231 (180-305)	315 (245-465)
	CVD Incidence	5.5 (4.3-8.0)	3.7 (2.8-5.8)	4592 (3580-6588)	4080 (3129-6360)	388 (303-557)	214 (164-334)
	T2D DALYs	7.8 (6.2-11.6)	5.5 (4.3-8.6)	5316 (4171-7842)	9245 (7172-14353)	449 (353-663)	485 (376-753)
	CVD DALYs	5.1 (4.0-7.7)	3.1 (2.4-4.8)	38336 (29894-57332)	14096 (10980-21496)	3241 (2528-4848)	740 (576-1128)
Austria	T2D Deaths	5.1 (4.0-8.2)	3.0 (2.4-4.6)	109 (84.9-172)	122 (95.6-185)	9.2 (7.2-14.5)	6.4 (5.0-9.7)
	CVD Deaths	3.6 (2.8-5.4)	1.9 (1.5-2.7)	1482 (1145-2216)	543 (424-768)	125 (96.8-187)	28.5 (22.2-40.3)
	T2D Incidence	8.2 (6.7-10.5)	8.7 (7.1-11.3)	612 (500-791)	1478 (1206-1952)	104 (84.7-134)	204 (166-269)
	CVD Incidence	2.8 (2.3-3.8)	2.6 (2.2-3.7)	1086 (900-1485)	1307 (1075-1863)	184 (152-252)	180 (148-257)
	T2D DALYs	4.4 (3.7-6.0)	4.4 (3.7-5.9)	1831 (1531-2492)	2754 (2299-3694)	310 (259-422)	380 (317-509)
	CVD DALYs	2.8 (2.3-3.9)	2.2 (1.8-3.2)	13596 (11330-18980)	6472 (5393-9454)	2303 (1920-3216)	892 (743-1303)
Azerbaijan	T2D Deaths	2.9 (2.4-4.1)	2.2 (1.8-3.1)	51.1 (42.4-71.2)	45.6 (38.2-65.0)	8.7 (7.2-12.1)	6.3 (5.3-9.0)
	CVD Deaths	2.0 (1.6-2.7)	1.5 (1.2-2.0)	558 (468-764)	291 (243-397)	94.5 (79.3-129)	40.1 (33.5-54.8)
	T2D Incidence	3.3 (2.2-5.6)	4.9 (3.3-7.9)	193 (127-335)	1325 (884-2145)	46.3 (30.4-80.5)	186 (124-301)
	CVD Incidence	1.1 (0.8-2.0)	1.7 (1.2-2.8)	398 (270-687)	1341 (925-2304)	95.7 (65.0-165)	188 (130-324)
	T2D DALYs	1.9 (1.3-3.3)	3.0 (2.0-5.1)	435 (290-787)	2560 (1761-4309)	105 (69.8-189)	360 (247-605)
	CVD DALYs	1.3 (0.9-2.4)	1.8 (1.2-3.0)	5149 (3510-9308)	10083 (6995-17052)	1237 (843-2236)	1417 (983-2396)
Bahamas, The	T2D Deaths	1.4 (1.0-2.7)	2.2 (1.5-3.9)	7.5 (5.1-13.9)	35.7 (24.6-65.8)	1.8 (1.2-3.3)	5.0 (3.5-9.2)
	CVD Deaths	0.9 (0.6-1.7)	1.3 (0.9-2.2)	161 (110-283)	354 (244-596)	38.7 (26.4-68.0)	49.7 (34.3-83.7)
	T2D Incidence	56.7 (42.8-69.8)	21.7 (14.1-31.4)	311 (235-384)	428 (281-623)	2129 (1605-2627)	1551 (1019-2258)

Supplementary Data 4. Proportional and absolute T2D and CVD burdens attributable to SSBs in 1990 and 2020 globally, regionally, and nationally (continued).

		Proportional burden % §		Absolute burden §		Absolute burden per 1M population §, ƒ	
Location	Outcome	1990	2020	1990	2020	1990	2020
	CVD Incidence	30.8 (21.6-41.8)	10.2 (6.4-15.7)	218 (152-298)	185 (115-285)	1495 (1043-2040)	671 (418-1032)
	T2D DALYs	42.7 (30.4-55.7)	15.5 (9.8-23.2)	1104 (780-1436)	1079 (684-1587)	7551 (5337-9822)	3911 (2482-5754)
	CVD DALYs	31.6 (22.0-42.6)	9.8 (6.0-14.9)	1792 (1252-2406)	811 (503-1242)	12260 (8563-16461)	2939 (1822-4502)
	T2D Deaths	34.0 (23.3-46.5)	11.1 (7.0-17.0)	23.9 (16.2-32.8)	14.8 (9.3-22.7)	164 (111-224)	53.7 (33.7-82.4)
	CVD Deaths	24.4 (16.6-33.8)	7.1 (4.4-10.9)	58.3 (39.7-81.2)	26.4 (16.6-40.2)	399 (272-555)	95.7 (60.2-146)
Bahrain	T2D Incidence	16.0 (10.9-23.0)	18.6 (12.5-27.3)	194 (130-280)	2333 (1577-3450)	655 (440-945)	1793 (1212-2650)
	CVD Incidence	9.4 (6.3-14.4)	11.4 (7.5-17.5)	191 (126-293)	1119 (732-1731)	644 (425-987)	860 (563-1330)
	T2D DALYs	11.5 (7.8-16.9)	14.5 (9.8-20.9)	501 (341-745)	4193 (2796-6048)	1692 (1152-2512)	3221 (2148-4647)
	CVD DALYs	9.3 (6.3-14.1)	11.1 (7.3-16.2)	1390 (928-2089)	2910 (1923-4232)	4690 (3132-7049)	2236 (1478-3251)
	T2D Deaths	9.1 (6.2-13.7)	10.8 (7.6-16.2)	11.5 (7.7-17.5)	66.9 (46.1-101)	38.7 (26.1-59.2)	51.4 (35.4-77.3)
	CVD Deaths	7.5 (5.0-11.3)	8.7 (5.8-12.7)	41.4 (27.8-62.5)	83.9 (55.9-123)	140 (93.7-211)	64.4 (43.0-94.9)
Bangladesh	T2D Incidence	0.8 (0.5-1.6)	1.0 (0.6-2.2)	792 (507-1582)	4524 (2782-9627)	16.3 (10.4-32.6)	43.1 (26.5-91.7)
	CVD Incidence	0.3 (0.2-0.5)	0.4 (0.2-0.7)	751 (495-1318)	2720 (1710-5135)	15.5 (10.2-27.2)	25.9 (16.3-48.9)
	T2D DALYs	0.5 (0.3-1.0)	0.6 (0.4-1.3)	1986 (1248-3710)	9437 (5771-18249)	41.0 (25.7-76.5)	89.8 (54.9-174)
	CVD DALYs	0.3 (0.2-0.5)	0.4 (0.2-0.7)	6062 (3938-10964)	16168 (10195-30245)	125 (81.2-226)	154 (97.1-288)
	T2D Deaths	0.3 (0.2-0.6)	0.4 (0.2-0.7)	38.1 (24.6-69.4)	134 (82.9-251)	0.8 (0.5-1.4)	1.3 (0.8-2.4)
	CVD Deaths	0.2 (0.1-0.4)	0.3 (0.2-0.5)	179 (118-304)	532 (332-936)	3.7 (2.4-6.3)	5.1 (3.2-8.9)
Barbados	T2D Incidence	32.5 (26.2-39.8)	41.3 (33.7-49.8)	271 (217-334)	814 (664-987)	1568 (1253-1931)	3695 (3014-4482)
	CVD Incidence	15.2 (11.8-19.2)	20.7 (16.0-25.8)	200 (154-254)	461 (357-581)	1157 (893-1468)	2094 (1621-2639)
	T2D DALYs	21.4 (16.7-26.9)	28.7 (22.8-35.2)	1250 (976-1571)	2877 (2283-3524)	7225 (5641-9086)	13064 (10366-16000)
	CVD DALYs	12.6 (9.7-16.0)	16.0 (12.2-20.0)	1243 (949-1580)	1506 (1156-1892)	7186 (5487-9136)	6837 (5249-8592)
	T2D Deaths	16.2 (12.4-21.0)	20.4 (15.8-26.0)	36.6 (27.9-47.8)	62.4 (48.5-79.3)	212 (161-276)	284 (220-360)
	CVD Deaths	9.2 (6.9-12.0)	11.8 (9.0-15.2)	54.4 (41.0-71.3)	64.8 (48.9-83.0)	314 (237-412)	294 (222-377)
Belarus	T2D Incidence	7.3 (4.9-11.0)	4.1 (2.8-6.7)	707 (468-1058)	668 (444-1089)	99.5 (66.0-149)	90.6 (60.2-148)
	CVD Incidence	2.3 (1.6-3.7)	1.3 (0.9-2.1)	2267 (1566-3572)	1570 (1078-2613)	319 (220-503)	213 (146-354)
	T2D DALYs	4.3 (2.9-6.6)	2.5 (1.7-4.3)	1218 (814-1912)	1136 (763-1971)	172 (115-269)	154 (103-267)
	CVD DALYs	2.5 (1.7-4.0)	1.3 (0.9-2.2)	25123 (17106-40025)	16481 (11271-27611)	3538 (2409-5637)	2234 (1528-3743)
	T2D Deaths	3.2 (2.1-5.4)	1.6 (1.1-2.9)	15.5 (10.4-25.8)	10.8 (7.4-18.6)	2.2 (1.5-3.6)	1.5 (1.0-2.5)

Supplementary Data 4. Proportional and absolute T2D and CVD burdens attributable to SSBs in 1990 and 2020 globally, regionally, and nationally (continued).

		Proportional burden % §		Absolute burden §		Absolute burden per 1M population §, ƒ	
Location	Outcome	1990	2020	1990	2020	1990	2020
	CVD Deaths	1.8 (1.2-2.8)	1.0 (0.7-1.6)	946 (649-1471)	672 (454-1103)	133 (91.4-207)	91.1 (61.6-150)
Belgium	T2D Incidence	15.1 (12.7-19.1)	16.6 (14.0-20.8)	2205 (1839-2792)	5070 (4239-6390)	292 (244-370)	565 (472-712)
	CVD Incidence	5.4 (4.6-8.0)	5.0 (4.2-7.1)	3406 (2859-5006)	2554 (2136-3722)	451 (379-663)	285 (238-415)
	T2D DALYs	8.3 (7.1-11.1)	9.7 (8.2-12.9)	4579 (3892-6145)	8518 (7220-11313)	607 (516-814)	949 (804-1261)
	CVD DALYs	4.8 (4.1-7.1)	3.9 (3.3-5.6)	24777 (21053-36707)	9699 (8301-14168)	3282 (2789-4863)	1081 (925-1579)
	T2D Deaths	4.4 (3.8-6.3)	3.9 (3.4-5.5)	78.6 (68.1-113)	57.7 (49.7-80.9)	10.4 (9.0-14.9)	6.4 (5.5-9.0)
	CVD Deaths	3.3 (2.8-4.9)	2.6 (2.2-3.6)	989 (852-1479)	425 (365-589)	131 (113-196)	47.3 (40.7-65.7)
Belize	T2D Incidence	21.1 (14.5-29.5)	30.4 (21.7-41.4)	53.7 (36.9-75.9)	454 (323-621)	632 (434-892)	1878 (1338-2569)
	CVD Incidence	9.8 (6.6-14.3)	14.9 (10.1-21.4)	38.1 (25.7-55.7)	185 (126-266)	447 (302-655)	764 (521-1100)
	T2D DALYs	14.1 (9.5-20.1)	21.4 (14.7-29.7)	194 (132-278)	1294 (886-1809)	2277 (1553-3272)	5356 (3666-7486)
	CVD DALYs	9.4 (6.3-13.7)	13.1 (9.0-18.8)	263 (177-384)	648 (446-929)	3089 (2079-4518)	2681 (1845-3844)
	T2D Deaths	10.3 (6.8-15.0)	15.7 (10.7-22.4)	4.4 (3.0-6.5)	22.8 (15.6-32.6)	52.3 (34.8-76.3)	94.4 (64.5-135)
	CVD Deaths	6.6 (4.5-10.1)	9.3 (6.3-13.6)	9.0 (6.0-13.7)	21.4 (14.4-31.4)	106 (71.0-161)	88.4 (59.8-130)
Benin	T2D Incidence	14.1 (9.0-21.6)	25.1 (17.0-36.1)	536 (343-826)	5139 (3487-7365)	245 (157-377)	894 (607-1282)
	CVD Incidence	5.8 (3.6-9.9)	11.2 (6.9-17.6)	533 (322-920)	2442 (1515-3804)	244 (147-421)	425 (264-662)
	T2D DALYs	10.6 (6.6-17.2)	19.1 (12.3-28.3)	1800 (1123-2900)	13483 (8737-20209)	823 (513-1326)	2346 (1520-3517)
	CVD DALYs	4.6 (2.8-7.9)	8.9 (5.6-14.3)	2637 (1610-4468)	11714 (7214-18705)	1206 (736-2043)	2038 (1255-3255)
	T2D Deaths	7.5 (4.6-12.7)	12.8 (8.0-20.3)	37.1 (22.5-61.4)	197 (121-313)	17.0 (10.3-28.1)	34.3 (21.1-54.5)
	CVD Deaths	3.4 (2.1-6.1)	6.6 (3.9-10.8)	92.5 (54.3-165)	388 (233-644)	42.3 (24.8-75.2)	67.5 (40.5-112)
Bhutan	T2D Incidence	4.4 (2.2-8.4)	10.3 (5.3-18.1)	21.4 (10.9-41.7)	172 (89.5-305)	87.3 (44.4-171)	337 (176-598)
	CVD Incidence	1.4 (0.7-3.3)	3.9 (1.9-7.9)	21.4 (10.8-48.9)	144 (71.1-300)	87.6 (44.0-200)	283 (140-589)
	T2D DALYs	2.8 (1.4-5.8)	6.5 (3.3-12.1)	50.9 (26.3-107)	386 (196-712)	208 (108-436)	758 (385-1397)
	CVD DALYs	1.5 (0.8-3.5)	3.9 (1.9-7.7)	126 (63.3-298)	671 (330-1354)	515 (259-1218)	1316 (646-2656)
	T2D Deaths	2.0 (1.0-4.4)	4.0 (1.9-8.5)	0.9 (0.4-2.0)	6.9 (3.1-14.6)	3.8 (1.8-8.0)	13.5 (6.1-28.6)
	CVD Deaths	1.2 (0.6-2.7)	2.8 (1.3-5.7)	3.5 (1.8-7.9)	22.1 (10.7-46.3)	14.5 (7.3-32.3)	43.3 (21.1-90.7)
Bolivia	T2D Incidence	35.2 (27.1-43.3)	29.1 (22.0-36.9)	2401 (1847-2974)	9844 (7409-12527)	700 (538-867)	1404 (1057-1787)
	CVD Incidence	19.1 (14.3-24.6)	15.2 (11.1-20.1)	1893 (1410-2451)	3977 (2922-5268)	552 (411-714)	567 (417-751)
	T2D DALYs	26.0 (19.6-33.2)	20.3 (14.9-26.4)	9777 (7332-12494)	26513 (19363-34303)	2850 (2137-3642)	3782 (2762-4893)

Supplementary Data 4. Proportional and absolute T2D and CVD burdens attributable to SSBs in 1990 and 2020 globally, regionally, and nationally (continued).

		Proportional burden % §		Absolute burden §		Absolute burden per 1M population §, ƒ	
Location	Outcome	1990	2020	1990	2020	1990	2020
	CVD DALYs	18.9 (14.1-24.4)	13.2 (9.8-17.6)	21361 (16136-27657)	23888 (17503-31806)	6226 (4703-8061)	3407 (2497-4537)
	T2D Deaths	21.4 (15.9-27.9)	15.7 (11.4-21.4)	248 (184-326)	562 (404-770)	72.2 (53.8-95.1)	80.2 (57.6-110)
	CVD Deaths	14.0 (10.4-18.4)	9.7 (7.1-13.4)	694 (511-919)	852 (621-1182)	202 (149-268)	122 (88.6-169)
Bosnia and Herzegovina	T2D Incidence	1.2 (0.8-2.4)	5.5 (3.8-8.9)	109 (71.7-216)	1045 (722-1696)	36.4 (23.9-72.0)	396 (273-643)
	CVD Incidence	0.6 (0.4-0.9)	2.1 (1.5-3.4)	114 (77.0-194)	610 (423-975)	38.0 (25.6-64.7)	231 (160-369)
	T2D DALYs	0.8 (0.5-1.6)	3.2 (2.2-5.2)	197 (132-379)	2384 (1633-3892)	65.5 (43.9-126)	903 (619-1475)
	CVD DALYs	0.5 (0.3-0.9)	1.8 (1.2-2.8)	1209 (811-2122)	4789 (3331-7530)	402 (270-706)	1815 (1262-2853)
	T2D Deaths	0.6 (0.4-1.1)	2.3 (1.6-3.8)	3.2 (2.2-5.8)	47.6 (32.7-78.4)	1.1 (0.7-1.9)	18.0 (12.4-29.7)
	CVD Deaths	0.4 (0.3-0.7)	1.3 (0.9-2.1)	40.7 (27.4-68.5)	208 (143-330)	13.6 (9.1-22.8)	78.9 (54.1-125)
Botswana	T2D Incidence	16.6 (11.6-23.0)	40.9 (29.9-52.4)	183 (127-255)	2142 (1551-2732)	326 (226-455)	1598 (1157-2038)
	CVD Incidence	8.8 (5.7-12.8)	23.0 (15.9-30.9)	230 (148-337)	1665 (1151-2261)	410 (264-602)	1242 (859-1687)
	T2D DALYs	12.8 (8.3-19.1)	32.0 (22.1-43.0)	1011 (655-1496)	7524 (5221-10148)	1802 (1167-2667)	5612 (3895-7570)
	CVD DALYs	8.1 (5.3-12.3)	20.9 (14.1-28.7)	1438 (941-2132)	7103 (4823-9887)	2563 (1678-3802)	5299 (3598-7375)
	T2D Deaths	10.6 (6.6-16.7)	25.8 (16.6-36.6)	29.1 (18.0-45.6)	184 (120-263)	51.8 (32.1-81.4)	137 (89.4-196)
	CVD Deaths	6.5 (4.1-10.1)	16.2 (10.4-23.3)	46.6 (29.8-71.8)	237 (152-338)	83.1 (53.1-128)	177 (113-252)
Brazil	T2D Incidence	24.2 (20.5-28.8)	14.2 (11.8-18.6)	57228 (48445-68377)	101691 (83258-133513)	700 (592-836)	668 (547-877)
	CVD Incidence	11.5 (9.7-14.0)	6.3 (5.3-8.8)	31596 (26647-38875)	37013 (30652-51108)	386 (326-475)	243 (201-336)
	T2D DALYs	17.4 (14.9-21.1)	9.5 (8.0-12.6)	171336 (145915-208658)	243007 (204341-322564)	2095 (1784-2551)	1595 (1342-2118)
	CVD DALYs	11.5 (9.7-14.2)	6.5 (5.4-8.8)	423803 (357420-523779)	318204 (265676-433569)	5182 (4370-6404)	2089 (1744-2846)
	T2D Deaths	13.2 (11.3-16.5)	6.6 (5.6-9.1)	3370 (2879-4195)	4160 (3515-5780)	41.2 (35.2-51.3)	27.3 (23.1-37.9)
	CVD Deaths	8.5 (7.2-10.5)	4.6 (3.9-6.3)	13510 (11427-16694)	10580 (8875-14334)	165 (140-204)	69.5 (58.3-94.1)
Brunei	T2D Incidence	6.5 (4.2-9.5)	14.8 (10.0-20.9)	33.9 (21.8-50.2)	466 (316-665)	233 (150-345)	1524 (1032-2174)
	CVD Incidence	2.1 (1.4-3.2)	4.9 (3.3-7.3)	6.5 (4.3-9.8)	32.7 (21.9-47.7)	44.7 (29.5-67.6)	107 (71.6-156)
	T2D DALYs	3.8 (2.5-5.6)	9.4 (6.4-14.1)	86.7 (57.6-129)	716 (483-1067)	595 (396-889)	2340 (1578-3485)
	CVD DALYs	3.4 (2.2-5.2)	7.3 (4.9-10.9)	162 (105-248)	575 (383-858)	1111 (719-1701)	1880 (1253-2802)
	T2D Deaths	2.5 (1.7-3.8)	6.2 (4.1-9.6)	1.8 (1.2-2.7)	8.4 (5.6-12.8)	12.3 (8.2-18.8)	27.6 (18.2-41.9)

Supplementary Data 4. Proportional and absolute T2D and CVD burdens attributable to SSBs in 1990 and 2020 globally, regionally, and nationally (continued).

		Proportional burden % §		Absolute burden §		Absolute burden per 1M population §, ƒ	
Location	Outcome	1990	2020	1990	2020	1990	2020
	CVD Deaths	2.1 (1.4-3.0)	5.1 (3.4-7.6)	3.8 (2.5-5.7)	14.5 (9.7-21.5)	26.4 (17.4-39.4)	47.3 (31.7-70.2)
Bulgaria	T2D Incidence	3.8 (2.7-6.1)	4.2 (3.0-7.0)	767 (552-1240)	1200 (870-2028)	120 (86.3-194)	214 (155-361)
	CVD Incidence	1.4 (1.1-2.2)	1.3 (1.0-2.1)	1137 (835-1763)	1095 (800-1763)	178 (130-276)	195 (143-314)
	T2D DALYs	2.4 (1.8-4.1)	2.4 (1.8-4.2)	1849 (1361-3127)	2598 (1880-4569)	289 (213-489)	463 (335-814)
	CVD DALYs	1.5 (1.1-2.4)	1.3 (1.0-2.2)	15136 (11133-24221)	11782 (8644-18617)	2365 (1740-3784)	2099 (1540-3317)
	T2D Deaths	1.9 (1.4-3.2)	1.7 (1.3-2.7)	36.4 (26.9-60.9)	40.4 (29.4-63.4)	5.7 (4.2-9.5)	7.2 (5.2-11.3)
	CVD Deaths	1.2 (0.9-1.8)	1.0 (0.7-1.4)	579 (424-875)	490 (354-735)	90.4 (66.2-137)	87.3 (63.0-131)
Burkina Faso	T2D Incidence	2.0 (0.6-5.9)	11.4 (3.9-27.9)	125 (40.0-365)	2927 (1010-7196)	33.6 (10.8-98.4)	314 (108-772)
	CVD Incidence	1.0 (0.3-3.0)	4.9 (1.6-13.1)	164 (52.4-492)	1714 (561-4605)	44.2 (14.1-132)	184 (60.1-494)
	T2D DALYs	1.7 (0.5-5.1)	8.9 (3.0-22.3)	663 (214-2039)	9275 (3090-23522)	179 (57.6-549)	995 (331-2523)
	CVD DALYs	0.9 (0.3-2.6)	4.1 (1.3-11.0)	873 (278-2624)	9320 (3112-26068)	235 (75.0-707)	1000 (334-2796)
	T2D Deaths	1.3 (0.4-3.9)	6.3 (2.1-16.7)	16.3 (5.2-48.8)	165 (55.4-434)	4.4 (1.4-13.1)	17.7 (5.9-46.6)
	CVD Deaths	0.7 (0.2-2.0)	3.1 (1.0-8.9)	29.7 (9.5-89.4)	317 (106-909)	8.0 (2.6-24.1)	34.0 (11.3-97.6)
Burundi	T2D Incidence	11.9 (7.8-17.8)	21.0 (14.5-30.1)	412 (269-624)	2157 (1474-3120)	178 (116-269)	408 (279-590)
	CVD Incidence	5.9 (3.8-10.2)	10.4 (6.7-16.8)	612 (383-1047)	1939 (1222-3186)	264 (165-451)	366 (231-602)
	T2D DALYs	10.1 (6.5-15.6)	16.6 (11.1-24.8)	3226 (2074-4978)	9748 (6425-14659)	1390 (893-2145)	1842 (1214-2770)
	CVD DALYs	6.0 (3.8-10.2)	10.5 (6.9-16.7)	5394 (3362-9224)	13088 (8564-21111)	2324 (1448-3974)	2473 (1618-3988)
	T2D Deaths	8.0 (5.1-12.9)	12.7 (8.2-19.8)	86.5 (53.9-145)	215 (139-336)	37.3 (23.2-62.5)	40.7 (26.2-63.5)
	CVD Deaths	4.6 (2.9-8.2)	8.0 (5.0-13.5)	165 (101-303)	381 (244-649)	71.1 (43.7-130)	71.9 (46.0-123)
Cambodia	T2D Incidence	0.3 (0.2-0.5)	11.8 (8.7-16.2)	17.8 (11.7-33.3)	4823 (3532-6627)	4.3 (2.9-8.1)	478 (350-657)
	CVD Incidence	0.1 (0.1-0.2)	4.6 (3.4-7.2)	12.5 (8.1-20.3)	1714 (1252-2640)	3.0 (2.0-4.9)	170 (124-262)
	T2D DALYs	0.2 (0.1-0.3)	8.0 (5.9-11.8)	69.0 (46.3-115)	11462 (8430-16858)	16.8 (11.3-28.0)	1136 (836-1671)
	CVD DALYs	0.1 (0.1-0.2)	5.3 (3.9-8.0)	211 (140-330)	20130 (14654-30498)	51.4 (34.2-80.5)	1995 (1453-3023)
	T2D Deaths	0.1 (0.1-0.2)	5.9 (4.4-9.0)	1.5 (1.0-2.5)	205 (152-319)	0.4 (0.3-0.6)	20.3 (15.1-31.7)
	CVD Deaths	0.1 (0.1-0.1)	3.6 (2.7-5.6)	5.7 (3.7-9.0)	590 (435-914)	1.4 (0.9-2.2)	58.4 (43.1-90.6)
Cameroon	T2D Incidence	6.5 (4.1-10.0)	24.9 (17.3-33.1)	501 (319-765)	10648 (7378-14166)	98.3 (62.5-150)	850 (589-1131)
	CVD Incidence	3.1 (2.0-5.1)	12.0 (8.0-17.5)	515 (327-848)	6086 (4006-8862)	101 (64.2-166)	486 (320-708)
	T2D DALYs	5.1 (3.2-8.1)	19.4 (13.4-27.1)	2322 (1476-3692)	38481 (26764-53857)	455 (289-724)	3073 (2137-4300)
	CVD DALYs	2.7 (1.7-4.4)	10.6 (7.0-15.6)	2912 (1838-4815)	40161 (26337-59609)	571 (360-944)	3207 (2103-4760)
	T2D Deaths	3.8 (2.4-6.3)	14.6 (9.5-21.3)	54.4 (34.2-89.2)	752 (495-1112)	10.7 (6.7-17.5)	60.1 (39.5-88.8)
	CVD Deaths	2.0 (1.3-3.4)	8.0 (5.2-12.7)	95.0 (59.6-161)	1284 (830-1983)	18.6 (11.7-31.6)	103 (66.3-158)
Canada	T2D Incidence	10.3 (8.7-13.3)	8.9 (7.3-12.4)	3922 (3326-5116)	15083 (12398-21173)	197 (167-257)	506 (416-711)
	CVD Incidence	4.3 (3.7-5.8)	3.4 (2.8-4.8)	6855 (5885-9416)	6266 (5238-8909)	344 (295-473)	210 (176-299)

Supplementary Data 4. Proportional and absolute T2D and CVD burdens attributable to SSBs in 1990 and 2020 globally, regionally, and nationally (continued).

		Proportional burden % §		Absolute burden §		Absolute burden per 1M population §, ƒ	
Location	Outcome	1990	2020	1990	2020	1990	2020
	T2D DALYs	5.8 (5.0-8.1)	5.3 (4.4-7.6)	6707 (5797-9410)	18934 (15815-27345)	337 (291-472)	635 (531-918)
	CVD DALYs	4.3 (3.7-5.9)	3.0 (2.5-4.3)	48328 (41486-66006)	26463 (22264-37948)	2427 (2083-3314)	888 (747-1273)
	T2D Deaths	4.1 (3.6-5.9)	3.1 (2.7-4.6)	165 (145-238)	186 (159-277)	8.3 (7.3-11.9)	6.2 (5.3-9.3)
	CVD Deaths	3.0 (2.6-4.1)	1.9 (1.6-2.7)	1804 (1577-2503)	1018 (860-1413)	90.6 (79.2-126)	34.2 (28.9-47.4)
Cape Verde	T2D Incidence	7.1 (4.7-11.1)	11.1 (7.2-17.4)	24.0 (16.1-37.2)	176 (115-279)	164 (110-255)	502 (328-795)
	CVD Incidence	3.0 (1.9-5.1)	4.7 (2.9-7.9)	31.1 (19.5-52.8)	96.0 (59.9-160)	212 (133-361)	273 (171-457)
	T2D DALYs	5.5 (3.6-8.4)	8.0 (5.2-12.5)	59.4 (38.9-93.0)	458 (297-732)	406 (266-637)	1304 (847-2085)
	CVD DALYs	2.8 (1.8-4.6)	4.0 (2.5-6.7)	159 (102-258)	580 (359-992)	1090 (701-1765)	1652 (1023-2826)
	T2D Deaths	3.7 (2.3-6.0)	4.8 (3.0-8.2)	0.9 (0.6-1.5)	7.1 (4.5-12.0)	6.3 (4.0-10.4)	20.3 (12.8-34.3)
	CVD Deaths	2.0 (1.2-3.3)	2.7 (1.7-4.6)	5.9 (3.6-10.0)	21.5 (13.1-36.2)	40.1 (24.6-68.2)	61.1 (37.2-103)
Central African Republic	T2D Incidence	6.7 (4.3-10.4)	8.9 (5.9-14.1)	206 (132-316)	979 (653-1563)	160 (102-245)	460 (307-734)
	CVD Incidence	2.9 (1.8-4.8)	3.7 (2.4-6.6)	149 (95.8-245)	365 (233-636)	115 (74.1-190)	171 (109-299)
	T2D DALYs	5.1 (3.4-8.2)	6.7 (4.4-11.0)	1118 (735-1804)	3466 (2269-5764)	865 (569-1396)	1627 (1066-2707)
	CVD DALYs	2.9 (1.8-4.7)	3.7 (2.4-6.5)	1530 (954-2523)	3393 (2150-6190)	1184 (738-1953)	1593 (1010-2907)
	T2D Deaths	4.2 (2.7-6.9)	5.2 (3.4-8.9)	25.3 (16.6-42.4)	59.9 (38.8-104)	19.6 (12.8-32.8)	28.1 (18.2-48.9)
	CVD Deaths	2.4 (1.5-4.1)	2.9 (1.9-5.2)	46.9 (28.9-81.9)	99.1 (62.8-173)	36.3 (22.4-63.4)	46.6 (29.5-81.1)
Chad	T2D Incidence	11.3 (7.6-16.5)	18.9 (13.0-26.2)	480 (327-703)	3445 (2376-4788)	190 (129-278)	497 (343-690)
	CVD Incidence	5.0 (3.1-8.4)	8.3 (5.3-13.0)	643 (399-1046)	2106 (1329-3333)	254 (158-414)	304 (192-481)
	T2D DALYs	8.7 (5.6-13.5)	14.4 (9.5-20.1)	1696 (1102-2619)	10248 (6734-14512)	671 (436-1036)	1478 (971-2093)
	CVD DALYs	4.3 (2.7-7.3)	7.1 (4.6-11.1)	3513 (2171-5971)	13390 (8496-20818)	1390 (859-2362)	1931 (1225-3002)
	T2D Deaths	6.4 (4.0-10.4)	10.4 (6.7-15.8)	36.8 (23.0-60.1)	176 (115-267)	14.6 (9.1-23.8)	25.4 (16.5-38.4)
	CVD Deaths	3.3 (2.0-5.6)	5.4 (3.4-8.9)	124 (74.6-210)	426 (269-709)	48.9 (29.5-83.2)	61.4 (38.9-102)
Chile	T2D Incidence	18.3 (13.9-24.5)	25.0 (19.1-32.7)	3288 (2490-4429)	19953 (15153-26523)	409 (310-551)	1406 (1068-1869)
	CVD Incidence	6.9 (5.1-9.5)	10.8 (8.1-14.7)	1934 (1444-2677)	4947 (3673-6743)	241 (180-333)	349 (259-475)
	T2D DALYs	11.9 (8.9-16.3)	16.8 (12.7-22.0)	6598 (4989-9048)	28692 (21787-37753)	821 (621-1126)	2021 (1535-2660)
	CVD DALYs	6.8 (5.0-9.1)	10.3 (7.8-13.9)	19612 (14539-26381)	30688 (23173-41215)	2441 (1810-3284)	2162 (1633-2904)
	T2D Deaths	9.0 (6.7-12.6)	10.7 (8.1-14.4)	135 (99.3-190)	299 (226-401)	16.8 (12.4-23.6)	21.1 (15.9-28.2)
	CVD Deaths	5.0 (3.8-6.9)	7.3 (5.5-9.8)	757 (570-1049)	1126 (846-1525)	94.3 (71.0-131)	79.4 (59.6-107)
China	T2D Incidence	1.2 (0.9-2.4)	0.8 (0.6-1.7)	19667 (14872-39870)	29598 (22699-62590)	27.5 (20.8-55.7)	26.9 (20.6-56.8)
	CVD Incidence	0.3 (0.3-0.6)	0.2 (0.2-0.3)	10218 (7869-17234)	20783 (16261-31937)	14.3 (11.0-24.1)	18.9 (14.8-29.0)
	T2D DALYs	0.7 (0.5-1.2)	0.5 (0.4-0.9)	26216 (20220-47926)	54204 (42073-101661)	36.7 (28.3-67.0)	49.2 (38.2-92.2)
	CVD DALYs	0.3 (0.3-0.6)	0.2 (0.2-0.3)	79123 (61496-134879)	112173 (87995-172944)	111 (86.0-189)	102 (79.8-157)

Supplementary Data 4. Proportional and absolute T2D and CVD burdens attributable to SSBs in 1990 and 2020 globally, regionally, and nationally (continued).

		Proportional burden % §		Absolute burden §		Absolute burden per 1M population §, ƒ	
Location	Outcome	1990	2020	1990	2020	1990	2020
	T2D Deaths	0.4 (0.3-0.6)	0.2 (0.2-0.3)	247 (198-393)	399 (318-578)	0.3 (0.3-0.5)	0.4 (0.3-0.5)
	CVD Deaths	0.2 (0.2-0.4)	0.1 (0.1-0.2)	2236 (1759-3404)	4011 (3163-5520)	3.1 (2.5-4.8)	3.6 (2.9-5.0)
Colombia	T2D Incidence	43.7 (35.6-52.4)	48.1 (39.3-57.3)	20944 (17084-25184)	69665 (56971-83199)	1177 (960-1415)	1971 (1612-2354)
	CVD Incidence	21.3 (16.6-27.0)	23.0 (18.0-29.2)	15402 (11842-19590)	38314 (29417-48799)	866 (666-1101)	1084 (832-1381)
	T2D DALYs	32.6 (25.9-40.0)	35.6 (28.3-44.0)	51834 (41165-64110)	156544 (124144-193880)	2913 (2314-3603)	4429 (3512-5485)
	CVD DALYs	21.3 (16.5-26.9)	21.2 (16.4-27.1)	132021 (102007-167258)	203818 (157607-260830)	7420 (5733-9400)	5766 (4459-7379)
	T2D Deaths	24.1 (18.5-30.6)	24.7 (19.0-31.6)	805 (615-1020)	1695 (1310-2179)	45.2 (34.6-57.3)	48.0 (37.1-61.6)
	CVD Deaths	16.1 (12.1-20.8)	15.5 (11.6-20.4)	4410 (3333-5729)	8009 (6030-10562)	248 (187-322)	227 (171-299)
Comoros	T2D Incidence	15.2 (10.6-21.0)	22.4 (16.2-30.0)	53.1 (36.9-72.8)	283 (205-381)	295 (205-404)	642 (464-864)
	CVD Incidence	7.7 (5.0-11.8)	10.9 (7.4-16.3)	64.6 (42.2-101)	221 (149-331)	359 (234-561)	501 (338-752)
	T2D DALYs	13.3 (9.1-19.4)	17.8 (12.6-25.1)	313 (211-455)	1120 (785-1570)	1737 (1171-2524)	2543 (1782-3563)
	CVD DALYs	7.4 (5.0-11.6)	10.5 (7.2-15.4)	398 (263-629)	1127 (767-1682)	2210 (1459-3488)	2557 (1740-3817)
	T2D Deaths	10.7 (7.0-16.0)	13.2 (8.9-19.5)	7.7 (5.1-11.8)	23.3 (15.6-34.2)	42.9 (28.2-65.6)	52.9 (35.5-77.6)
	CVD Deaths	5.8 (3.7-9.1)	7.8 (5.2-12.0)	12.1 (7.7-19.6)	36.0 (24.3-55.4)	67.0 (42.6-109)	81.6 (55.2-126)
Congo, Dem. Rep.	T2D Incidence	5.5 (1.9-15.3)	7.8 (2.7-21.1)	1618 (573-4489)	9339 (3281-25640)	105 (37.1-291)	239 (84.0-656)
	CVD Incidence	2.4 (0.8-7.5)	3.2 (1.1-9.4)	1665 (576-5241)	4606 (1588-13433)	108 (37.3-339)	118 (40.6-344)
	T2D DALYs	4.3 (1.5-11.9)	5.7 (2.0-15.9)	8995 (3177-25048)	32775 (11426-90970)	582 (206-1622)	839 (292-2328)
	CVD DALYs	2.3 (0.8-7.0)	2.9 (1.0-8.5)	12229 (4129-38236)	30367 (10438-88508)	792 (267-2476)	777 (267-2265)
	T2D Deaths	3.4 (1.2-10.1)	4.2 (1.5-11.8)	216 (72.9-653)	618 (208-1742)	14.0 (4.7-42.3)	15.8 (5.3-44.6)
	CVD Deaths	1.9 (0.6-5.8)	2.3 (0.8-6.7)	393 (131-1211)	956 (325-2829)	25.4 (8.5-78.4)	24.5 (8.3-72.4)
Congo, Rep.	T2D Incidence	7.3 (4.8-11.2)	28.1 (19.9-37.4)	168 (110-258)	2947 (2073-3902)	161 (106-247)	1105 (777-1463)
	CVD Incidence	3.4 (2.2-5.6)	14.0 (9.5-20.4)	179 (117-295)	1782 (1208-2626)	172 (112-283)	668 (453-984)
	T2D DALYs	5.9 (3.9-9.5)	22.5 (15.5-31.4)	1143 (759-1833)	12165 (8385-16785)	1097 (728-1760)	4560 (3143-6292)
	CVD DALYs	3.5 (2.3-5.8)	13.7 (9.2-19.9)	1931 (1243-3188)	14654 (9787-21567)	1854 (1193-3060)	5493 (3668-8084)
	T2D Deaths	4.8 (3.1-8.0)	17.9 (12.0-26.2)	29.0 (18.4-48.2)	251 (165-366)	27.9 (17.7-46.3)	94.0 (61.8-137)
	CVD Deaths	2.9 (1.8-4.8)	10.9 (7.0-16.5)	61.4 (39.6-103)	462 (303-707)	58.9 (38.0-98.4)	173 (114-265)
Costa Rica	T2D Incidence	30.7 (21.7-40.3)	21.7 (14.6-29.7)	1324 (941-1753)	3830 (2607-5291)	764 (543-1012)	1044 (710-1442)

Supplementary Data 4. Proportional and absolute T2D and CVD burdens attributable to SSBs in 1990 and 2020 globally, regionally, and nationally (continued).

		Proportional burden % §		Absolute burden §		Absolute burden per 1M population §, ƒ	
Location	Outcome	1990	2020	1990	2020	1990	2020
	CVD Incidence	14.0 (9.5-19.8)	9.4 (6.2-13.7)	1040 (706-1467)	1867 (1224-2740)	601 (408-847)	509 (334-747)
	T2D DALYs	21.7 (15.2-29.4)	14.6 (9.7-20.8)	2861 (1970-3849)	8144 (5414-11807)	1652 (1138-2223)	2219 (1475-3217)
	CVD DALYs	12.8 (8.6-18.1)	8.8 (5.8-12.9)	6197 (4191-8796)	6528 (4308-9616)	3579 (2421-5080)	1779 (1174-2620)
	T2D Deaths	15.6 (10.5-22.1)	8.9 (5.7-13.3)	38.3 (25.7-53.9)	85.6 (55.0-128)	22.1 (14.8-31.2)	23.3 (15.0-34.9)
	CVD Deaths	9.1 (6.1-13.2)	5.9 (3.9-9.0)	223 (149-322)	227 (149-345)	129 (86.1-186)	61.8 (40.6-94.1)
Cote d'Ivoire	T2D Incidence	12.8 (8.7-18.7)	21.9 (15.2-30.2)	1108 (751-1582)	9169 (6352-12657)	206 (140-295)	732 (507-1011)
	CVD Incidence	6.2 (4.1-9.8)	10.8 (7.2-15.7)	1185 (782-1890)	5544 (3629-8048)	221 (146-352)	443 (290-643)
	T2D DALYs	10.1 (6.8-15.1)	17.3 (11.7-24.4)	3870 (2620-5781)	27420 (18628-38807)	721 (488-1077)	2190 (1487-3099)
	CVD DALYs	5.6 (3.7-8.9)	9.6 (6.5-14.3)	8046 (5199-12524)	37567 (24936-56139)	1498 (968-2333)	3000 (1991-4483)
	T2D Deaths	7.7 (5.0-11.9)	12.7 (8.4-18.4)	77.1 (49.1-118)	460 (302-672)	14.4 (9.1-22.0)	36.7 (24.1-53.7)
	CVD Deaths	4.4 (2.8-7.1)	7.4 (4.8-11.2)	241 (153-385)	1154 (730-1780)	44.8 (28.5-71.7)	92.2 (58.3-142)
Croatia	T2D Incidence	5.4 (4.0-7.9)	6.3 (4.7-9.3)	624 (462-927)	1056 (779-1544)	178 (132-265)	319 (235-466)
	CVD Incidence	2.0 (1.5-3.2)	2.1 (1.6-3.0)	671 (504-1059)	668 (501-975)	192 (144-303)	202 (151-294)
	T2D DALYs	3.5 (2.6-5.4)	3.5 (2.7-5.2)	1113 (837-1756)	2074 (1558-3047)	318 (239-502)	626 (471-920)
	CVD DALYs	2.0 (1.5-3.1)	1.9 (1.4-2.7)	7747 (5843-12151)	5002 (3737-7350)	2215 (1671-3475)	1510 (1128-2220)
	T2D Deaths	2.5 (1.9-4.0)	2.2 (1.7-3.2)	17.8 (13.5-28.4)	34.0 (25.3-48.7)	5.1 (3.9-8.1)	10.3 (7.7-14.7)
	CVD Deaths	1.4 (1.1-2.2)	1.4 (1.0-2.0)	312 (233-475)	241 (179-344)	89.2 (66.5-136)	72.6 (54.1-104)
Cuba	T2D Incidence	37.8 (30.2-46.1)	27.6 (20.9-35.5)	7182 (5691-8773)	12063 (9118-15618)	1022 (810-1248)	1358 (1027-1759)
	CVD Incidence	17.8 (13.6-23.1)	12.8 (9.5-17.3)	9104 (6900-11820)	9567 (7101-12894)	1295 (982-1682)	1077 (800-1452)
	T2D DALYs	27.1 (20.5-34.6)	20.2 (15.2-26.8)	22350 (16907-28501)	28448 (21350-37699)	3180 (2406-4055)	3203 (2404-4245)
	CVD DALYs	17.4 (13.2-22.5)	11.3 (8.4-15.4)	81568 (61905-106119)	58512 (43469-79854)	11606 (8808-15100)	6589 (4895-8992)
	T2D Deaths	20.2 (14.8-27.2)	13.5 (9.8-18.5)	433 (318-579)	267 (193-365)	61.6 (45.2-82.4)	30.1 (21.8-41.1)
	CVD Deaths	13.1 (9.8-17.2)	8.4 (6.1-11.6)	3200 (2380-4218)	2463 (1805-3408)	455 (339-600)	277 (203-384)
Cyprus	T2D Incidence	6.7 (3.4-13.3)	9.7 (5.0-18.4)	161 (81.9-321)	499 (260-958)	314 (159-625)	534 (279-1027)
	CVD Incidence	3.0 (1.5-6.3)	3.3 (1.7-6.8)	54.8 (27.9-116)	107 (53.8-214)	107 (54.3-227)	114 (57.7-229)
	T2D DALYs	3.8 (1.9-7.8)	5.3 (2.7-10.5)	392 (197-793)	832 (420-1633)	763 (383-1544)	892 (450-1749)
	CVD DALYs	2.8 (1.4-5.8)	3.3 (1.7-6.8)	979 (498-2016)	1082 (542-2240)	1905 (969-3924)	1159 (581-2400)
	T2D Deaths	2.6 (1.3-5.4)	2.7 (1.4-5.7)	11.4 (5.6-23.3)	13.6 (6.8-28.2)	22.1 (11.0-45.3)	14.6 (7.3-30.2)
	CVD Deaths	2.0 (1.0-4.1)	2.2 (1.1-4.5)	38.9 (19.9-82.2)	42.1 (21.2-88.5)	75.7 (38.6-160)	45.1 (22.7-94.9)

Supplementary Data 4. Proportional and absolute T2D and CVD burdens attributable to SSBs in 1990 and 2020 globally, regionally, and nationally (continued).

		Proportional burden % §		Absolute burden §		Absolute burden per 1M population §, ƒ	
Location	Outcome	1990	2020	1990	2020	1990	2020
Czech Republic	T2D Incidence	5.0 (3.9-7.4)	3.8 (3.0-5.9)	1105 (871-1647)	1704 (1331-2612)	152 (120-227)	200 (156-306)
	CVD Incidence	1.9 (1.5-2.7)	1.4 (1.1-2.0)	1888 (1500-2643)	1321 (1059-1889)	260 (206-364)	155 (124-221)
	T2D DALYs	3.0 (2.4-4.4)	2.1 (1.7-3.1)	2253 (1778-3332)	3388 (2690-5016)	310 (245-458)	397 (315-588)
	CVD DALYs	1.9 (1.5-2.6)	1.2 (0.9-1.7)	21202 (16783-30200)	6968 (5500-10036)	2917 (2309-4155)	816 (644-1176)
	T2D Deaths	2.2 (1.7-3.3)	1.3 (1.0-1.9)	41.5 (32.5-62.6)	57.0 (43.7-83.2)	5.7 (4.5-8.6)	6.7 (5.1-9.8)
	CVD Deaths	1.4 (1.1-2.0)	0.8 (0.7-1.2)	845 (662-1212)	326 (251-460)	116 (91.0-167)	38.2 (29.4-53.9)
Denmark	T2D Incidence	5.3 (4.2-8.1)	6.7 (5.3-9.8)	280 (220-423)	914 (720-1330)	71.8 (56.5-109)	203 (160-295)
	CVD Incidence	1.3 (1.1-2.0)	2.0 (1.6-3.1)	274 (223-406)	383 (306-602)	70.2 (57.1-104)	84.8 (67.8-133)
	T2D DALYs	2.9 (2.3-4.7)	3.5 (2.8-5.1)	632 (514-1036)	1432 (1154-2096)	162 (132-266)	318 (256-465)
	CVD DALYs	1.6 (1.3-2.4)	1.7 (1.4-2.6)	5997 (4901-9430)	2187 (1766-3222)	1538 (1257-2419)	485 (392-714)
	T2D Deaths	1.8 (1.5-2.8)	1.8 (1.5-2.5)	14.4 (11.9-22.0)	23.2 (19.1-33.0)	3.7 (3.0-5.6)	5.2 (4.2-7.3)
	CVD Deaths	1.1 (0.9-1.6)	1.1 (0.9-1.5)	249 (205-356)	89.8 (73.8-121)	64.0 (52.5-91.4)	19.9 (16.4-26.7)
Djibouti	T2D Incidence	31.2 (22.3-41.4)	48.5 (36.1-59.0)	58.9 (41.9-78.2)	692 (518-847)	224 (160-298)	1132 (846-1386)
	CVD Incidence	18.4 (12.1-26.7)	30.0 (20.5-40.3)	108 (71.6-158)	832 (573-1129)	413 (273-600)	1360 (937-1846)
	T2D DALYs	27.5 (19.2-37.9)	43.0 (31.1-54.0)	355 (244-490)	3348 (2425-4282)	1350 (928-1864)	5474 (3965-7001)
	CVD DALYs	17.8 (11.8-26.5)	29.5 (20.4-39.6)	562 (372-832)	4924 (3450-6698)	2139 (1417-3166)	8052 (5642-10953)
	T2D Deaths	22.8 (15.1-32.9)	35.9 (24.6-46.8)	8.6 (5.8-12.5)	77.9 (53.9-104)	32.8 (21.9-47.7)	127 (88.2-169)
	CVD Deaths	14.2 (9.1-21.6)	24.4 (16.2-33.7)	15.9 (10.3-23.7)	149 (99.2-207)	60.7 (39.1-90.4)	243 (162-338)
Dominica	T2D Incidence	26.8 (18.8-36.9)	19.9 (13.4-27.9)	50.6 (35.4-69.9)	81.9 (54.8-116)	1254 (877-1734)	1670 (1117-2358)
	CVD Incidence	12.5 (8.4-18.0)	9.0 (5.9-13.2)	32.2 (21.4-46.1)	32.5 (20.7-48.2)	798 (532-1143)	663 (422-984)
	T2D DALYs	17.2 (11.6-24.4)	13.1 (8.6-19.0)	205 (138-292)	265 (176-386)	5091 (3419-7230)	5408 (3596-7872)
	CVD DALYs	10.1 (6.6-14.7)	7.2 (4.6-10.6)	223 (147-324)	147 (94.2-217)	5539 (3653-8025)	2994 (1921-4422)
	T2D Deaths	12.9 (8.5-18.5)	9.2 (6.0-13.6)	5.4 (3.6-7.8)	5.3 (3.4-7.8)	134 (88.2-193)	107 (69.2-160)
	CVD Deaths	7.7 (5.1-11.2)	5.3 (3.4-7.8)	9.5 (6.3-13.9)	5.8 (3.7-8.6)	235 (155-344)	119 (76.3-174)
Dominican Republic	T2D Incidence	13.3 (9.9-18.3)	25.0 (19.1-33.0)	1272 (944-1748)	10872 (8297-14364)	353 (262-485)	1573 (1200-2078)
	CVD Incidence	6.1 (4.5-8.8)	10.9 (8.1-15.0)	928 (688-1331)	5013 (3684-6974)	257 (191-369)	725 (533-1009)
	T2D DALYs	9.4 (7.1-12.9)	17.8 (13.5-23.9)	3240 (2427-4468)	26849 (20410-36269)	898 (673-1239)	3884 (2953-5247)
	CVD DALYs	6.4 (4.8-9.2)	11.6 (8.4-15.9)	8498 (6402-12109)	44234 (32477-60475)	2356 (1775-3358)	6399 (4698-8748)

Supplementary Data 4. Proportional and absolute T2D and CVD burdens attributable to SSBs in 1990 and 2020 globally, regionally, and nationally (continued).

		Proportional burden % §		Absolute burden §		Absolute burden per 1M population §, ƒ	
Location	Outcome	1990	2020	1990	2020	1990	2020
	T2D Deaths	6.7 (5.0-9.5)	13.0 (9.6-17.7)	50.8 (37.5-72.2)	367 (269-501)	14.1 (10.4-20.0)	53.0 (38.9-72.5)
	CVD Deaths	4.4 (3.3-6.3)	8.4 (6.1-11.5)	254 (191-362)	1480 (1076-2047)	70.4 (52.9-100)	214 (156-296)
Ecuador	T2D Incidence	48.6 (40.5-56.4)	34.7 (27.1-43.3)	5412 (4529-6325)	21862 (17037-27189)	1039 (870-1214)	1943 (1514-2417)
	CVD Incidence	27.2 (21.9-33.3)	17.8 (13.3-23.3)	4559 (3630-5563)	8369 (6246-10906)	875 (697-1068)	744 (555-969)
	T2D DALYs	38.0 (30.7-45.7)	24.6 (18.6-32.0)	16204 (13074-19521)	48533 (36819-62434)	3111 (2510-3748)	4314 (3273-5550)
	CVD DALYs	26.8 (21.8-32.5)	16.7 (12.7-22.0)	35317 (28554-42795)	45002 (34297-59432)	6781 (5483-8217)	4000 (3049-5283)
	T2D Deaths	30.5 (24.3-38.0)	18.0 (13.6-24.1)	341 (272-424)	784 (585-1057)	65.5 (52.3-81.3)	69.7 (52.0-94.0)
	CVD Deaths	19.2 (15.2-24.1)	11.4 (8.5-15.4)	1177 (932-1473)	1586 (1188-2156)	226 (179-283)	141 (106-192)
Egypt, Arab Rep.	T2D Incidence	10.0 (7.6-13.0)	9.5 (7.2-12.9)	4318 (3304-5682)	32494 (24893-44515)	155 (119-204)	551 (422-756)
	CVD Incidence	5.0 (3.8-7.7)	4.7 (3.5-7.3)	15112 (11254-23023)	34437 (25928-53687)	544 (405-828)	584 (440-911)
	T2D DALYs	7.4 (5.6-9.8)	6.5 (5.0-9.1)	16418 (12594-22089)	72481 (55565-100036)	591 (453-795)	1230 (943-1698)
	CVD DALYs	5.5 (4.2-7.8)	4.8 (3.6-6.8)	145257 (109678-207517)	254408 (192488-362992)	5227 (3947-7467)	4318 (3267-6161)
	T2D Deaths	6.1 (4.6-8.3)	5.2 (3.9-7.4)	389 (296-539)	1370 (1028-1929)	14.0 (10.6-19.4)	23.2 (17.5-32.7)
	CVD Deaths	4.2 (3.2-6.0)	3.8 (2.8-5.4)	4356 (3314-6270)	7976 (6010-11608)	157 (119-226)	135 (102-197)
El Salvador	T2D Incidence	18.5 (13.0-26.2)	28.3 (20.3-38.6)	1025 (720-1461)	5335 (3853-7303)	395 (277-563)	1278 (923-1750)
	CVD Incidence	7.5 (5.2-11.3)	11.1 (7.6-16.4)	778 (534-1162)	2373 (1611-3562)	300 (206-448)	569 (386-853)
	T2D DALYs	12.6 (8.7-18.4)	18.6 (12.9-26.3)	3144 (2168-4624)	17887 (12552-25520)	1212 (835-1782)	4286 (3008-6115)
	CVD DALYs	8.5 (5.9-12.5)	11.4 (8.0-16.7)	7541 (5220-11177)	16122 (11305-23827)	2906 (2012-4308)	3863 (2709-5709)
	T2D Deaths	9.2 (6.3-13.7)	13.5 (9.2-19.6)	58.0 (40.0-86.0)	356 (248-521)	22.4 (15.4-33.1)	85.4 (59.5-125)
	CVD Deaths	5.8 (4.0-8.6)	7.7 (5.3-11.6)	235 (162-351)	585 (407-867)	90.5 (62.4-135)	140 (97.5-208)
Equatorial Guinea	T2D Incidence	11.6 (7.7-16.5)	23.2 (15.8-32.6)	46.0 (30.9-66.4)	589 (402-835)	227 (152-327)	769 (524-1090)
	CVD Incidence	5.1 (3.2-8.2)	11.1 (7.1-16.7)	46.3 (29.3-74.4)	259 (163-388)	228 (144-367)	338 (213-507)
	T2D DALYs	9.2 (6.1-13.9)	17.9 (12.0-25.6)	289 (189-434)	1925 (1278-2782)	1426 (933-2141)	2514 (1668-3632)
	CVD DALYs	5.1 (3.3-8.4)	10.5 (6.6-15.7)	444 (282-725)	1778 (1130-2717)	2187 (1392-3572)	2320 (1475-3547)
	T2D Deaths	7.5 (4.9-11.8)	13.1 (8.5-19.8)	7.1 (4.6-11.2)	35.6 (22.6-53.8)	35.0 (22.7-55.0)	46.5 (29.4-70.2)
	CVD Deaths	4.2 (2.6-7.1)	7.9 (5.0-12.4)	14.3 (9.0-24.4)	56.2 (35.3-89.4)	70.3 (44.1-120)	73.4 (46.0-117)
Eritrea	T2D Incidence	18.0 (12.5-24.9)	30.2 (21.8-39.2)	362 (251-498)	2479 (1777-3223)	360 (249-494)	1446 (1037-1880)

Supplementary Data 4. Proportional and absolute T2D and CVD burdens attributable to SSBs in 1990 and 2020 globally, regionally, and nationally (continued).

		Proportional burden % §		Absolute burden §		Absolute burden per 1M population §, ƒ	
Location	Outcome	1990	2020	1990	2020	1990	2020
	CVD Incidence	9.0 (5.9-14.1)	14.5 (9.6-20.9)	406 (261-651)	1523 (1008-2193)	403 (259-646)	888 (588-1279)
	T2D DALYs	15.5 (10.2-22.4)	24.6 (16.9-33.3)	2579 (1706-3753)	10853 (7508-14835)	2559 (1693-3724)	6332 (4381-8655)
	CVD DALYs	9.5 (6.1-15.2)	15.3 (10.1-21.7)	3110 (1970-5138)	11639 (7542-16560)	3085 (1955-5098)	6791 (4400-9661)
	T2D Deaths	13.1 (8.6-19.9)	19.4 (12.6-27.3)	60.3 (39.6-90.9)	222 (145-315)	59.8 (39.3-90.2)	129 (84.7-184)
	CVD Deaths	7.9 (5.1-12.9)	12.0 (7.7-17.5)	85.4 (54.3-140)	326 (211-482)	84.7 (53.8-139)	190 (123-281)
Estonia	T2D Incidence	4.2 (3.2-6.6)	5.4 (4.2-7.9)	80.5 (61.9-128)	186 (145-274)	72.6 (55.8-116)	178 (138-262)
	CVD Incidence	1.3 (1.1-2.2)	1.7 (1.3-2.6)	210 (167-336)	264 (212-414)	190 (150-303)	252 (203-396)
	T2D DALYs	2.5 (2.0-4.4)	2.8 (2.3-4.5)	128 (101-223)	372 (299-581)	115 (90.8-201)	356 (286-556)
	CVD DALYs	1.4 (1.1-2.3)	1.4 (1.1-2.1)	2413 (1906-4031)	820 (648-1237)	2176 (1719-3635)	784 (620-1183)
	T2D Deaths	1.8 (1.4-3.0)	1.9 (1.5-3.0)	1.4 (1.1-2.4)	5.4 (4.3-8.7)	1.3 (1.0-2.2)	5.2 (4.1-8.4)
	CVD Deaths	1.0 (0.8-1.4)	1.0 (0.8-1.4)	96.4 (75.5-142)	37.5 (29.0-52.8)	86.9 (68.1-128)	35.9 (27.8-50.5)
Ethiopia (excludes Eritrea)	T2D Incidence	11.9 (8.8-16.0)	23.8 (18.7-29.8)	4679 (3492-6323)	24230 (18836-30109)	225 (168-304)	432 (336-537)
	CVD Incidence	6.0 (4.3-9.2)	11.0 (8.2-15.2)	4389 (3144-6804)	15738 (11697-21878)	211 (151-327)	281 (209-390)
	T2D DALYs	10.1 (7.4-14.3)	18.3 (13.9-23.4)	37054 (27062-52858)	86447 (65140-110320)	1779 (1299-2538)	1541 (1161-1967)
	CVD DALYs	6.4 (4.6-9.7)	10.8 (8.0-14.7)	32549 (23316-50290)	74621 (54780-102088)	1563 (1119-2415)	1330 (977-1820)
	T2D Deaths	8.3 (5.8-12.0)	13.0 (9.3-17.6)	928 (648-1358)	1727 (1225-2348)	44.6 (31.1-65.2)	30.8 (21.8-41.9)
	CVD Deaths	5.2 (3.6-7.9)	7.9 (5.6-11.0)	931 (655-1439)	2246 (1602-3131)	44.7 (31.4-69.1)	40.0 (28.6-55.8)
Fiji	T2D Incidence	4.2 (2.9-6.6)	10.9 (7.7-15.2)	102 (68.5-159)	796 (560-1108)	270 (182-424)	1419 (998-1974)
	CVD Incidence	1.5 (1.1-2.5)	3.9 (2.8-5.9)	26.7 (19.1-43.9)	147 (105-223)	71.0 (50.9-117)	262 (188-398)
	T2D DALYs	2.5 (1.7-4.3)	6.3 (4.5-9.5)	513 (361-872)	3687 (2615-5547)	1366 (961-2321)	6571 (4660-9885)
	CVD DALYs	2.1 (1.5-3.8)	5.2 (3.7-7.9)	678 (471-1213)	2690 (1906-4074)	1804 (1255-3230)	4793 (3396-7259)
	T2D Deaths	1.9 (1.3-3.2)	4.8 (3.4-7.4)	11.7 (8.4-20.0)	86.6 (61.6-132)	31.2 (22.3-53.3)	154 (110-234)
	CVD Deaths	1.6 (1.1-2.9)	3.9 (2.8-6.0)	16.9 (11.8-30.4)	74.0 (53.1-113)	44.9 (31.3-80.9)	132 (94.5-201)
Finland	T2D Incidence	7.1 (5.8-9.9)	6.5 (5.4-9.3)	648 (525-898)	1213 (986-1722)	174 (141-241)	278 (226-395)
	CVD Incidence	2.5 (2.1-3.9)	1.6 (1.3-2.3)	1068 (892-1682)	611 (509-884)	286 (239-451)	140 (117-203)
	T2D DALYs	4.1 (3.4-6.0)	3.7 (3.0-5.4)	878 (729-1274)	1664 (1379-2470)	235 (196-342)	381 (316-566)
	CVD DALYs	2.2 (1.9-3.5)	1.5 (1.2-2.1)	8362 (7011-12905)	3307 (2795-4707)	2243 (1880-3461)	758 (640-1078)
	T2D Deaths	1.9 (1.7-2.7)	1.7 (1.4-2.4)	8.7 (7.4-12.2)	9.1 (7.7-13.1)	2.3 (2.0-3.3)	2.1 (1.8-3.0)
	CVD Deaths	1.6 (1.3-2.4)	1.0 (0.8-1.4)	313 (265-464)	149 (127-202)	83.9 (71.1-125)	34.2 (29.0-46.2)
France	T2D Incidence	6.8 (5.7-9.1)	9.1 (7.7-12.1)	4462 (3710-5896)	12148 (10283-16041)	109 (90.4-144)	244 (206-322)
	CVD Incidence	2.5 (2.1-3.5)	2.6 (2.2-3.7)	7034 (6076-10081)	9721 (8322-14230)	171 (148-246)	195 (167-285)

Supplementary Data 4. Proportional and absolute T2D and CVD burdens attributable to SSBs in 1990 and 2020 globally, regionally, and nationally (continued).

		Proportional burden % §		Absolute burden §		Absolute burden per 1M population §, ƒ	
Location	Outcome	1990	2020	1990	2020	1990	2020
	T2D DALYs	3.5 (3.0-4.9)	4.7 (4.0-6.3)	7275 (6245-10181)	18734 (16061-25247)	177 (152-248)	376 (322-506)
	CVD DALYs	2.0 (1.8-2.8)	2.2 (1.9-3.0)	37689 (32546-52539)	27832 (24074-39090)	918 (793-1280)	558 (483-784)
	T2D Deaths	1.9 (1.6-2.7)	2.0 (1.8-2.8)	148 (130-210)	262 (230-365)	3.6 (3.2-5.1)	5.2 (4.6-7.3)
	CVD Deaths	1.3 (1.1-1.8)	1.3 (1.1-1.8)	1485 (1298-2020)	1127 (984-1504)	36.2 (31.6-49.2)	22.6 (19.7-30.2)
Gabon	T2D Incidence	28.3 (20.3-37.3)	21.2 (14.3-29.6)	362 (259-479)	964 (644-1367)	786 (561-1038)	801 (536-1136)
	CVD Incidence	14.7 (9.8-21.4)	10.3 (6.6-15.8)	383 (251-567)	481 (306-735)	831 (544-1228)	399 (254-611)
	T2D DALYs	23.4 (15.9-32.7)	16.5 (10.8-24.2)	2305 (1559-3250)	3978 (2576-5864)	4996 (3379-7047)	3307 (2141-4875)
	CVD DALYs	14.3 (9.3-21.0)	9.7 (6.1-15.1)	3030 (2007-4446)	3195 (2017-4924)	6569 (4350-9638)	2656 (1677-4094)
	T2D Deaths	19.0 (12.2-27.7)	12.8 (7.9-19.7)	63.1 (40.4-91.9)	84.8 (52.4-129)	137 (87.6-199)	70.5 (43.6-107)
	CVD Deaths	11.5 (7.2-17.7)	7.5 (4.6-12.0)	109 (68.6-166)	107 (65.7-174)	236 (149-359)	89.3 (54.6-144)
Gambia, The	T2D Incidence	49.8 (38.9-59.8)	21.8 (14.8-31.1)	307 (239-373)	710 (476-1005)	691 (538-839)	647 (434-916)
	CVD Incidence	28.7 (20.0-38.6)	9.7 (6.1-14.8)	486 (344-660)	467 (295-726)	1095 (775-1486)	426 (269-661)
	T2D DALYs	42.4 (31.4-53.1)	16.4 (10.8-24.0)	1200 (890-1506)	2255 (1482-3289)	2704 (2003-3392)	2055 (1350-2997)
	CVD DALYs	25.6 (17.5-34.8)	8.6 (5.5-13.2)	3172 (2174-4357)	3468 (2231-5400)	7144 (4897-9814)	3160 (2033-4920)
	T2D Deaths	34.5 (24.6-45.6)	11.7 (7.4-18.1)	26.9 (18.9-35.2)	42.2 (26.5-66.2)	60.6 (42.5-79.2)	38.5 (24.2-60.3)
	CVD Deaths	20.8 (13.9-30.3)	6.4 (3.9-10.3)	107 (70.4-156)	115 (70.4-187)	241 (159-351)	105 (64.1-171)
Georgia	T2D Incidence	0.3 (0.2-0.6)	6.7 (4.5-10.4)	17.7 (12.0-43.7)	746 (503-1162)	4.8 (3.3-12.0)	252 (170-392)
	CVD Incidence	0.1 (0.1-0.1)	2.0 (1.4-3.2)	48.1 (32.8-76.6)	811 (553-1304)	13.2 (9.0-21.0)	274 (187-440)
	T2D DALYs	0.2 (0.1-0.3)	3.6 (2.5-5.9)	43.3 (29.7-72.2)	1697 (1152-2749)	11.9 (8.1-19.8)	572 (389-927)
	CVD DALYs	0.1 (0.1-0.2)	1.8 (1.3-3.0)	512 (349-819)	4971 (3385-8003)	140 (95.6-224)	1677 (1142-2699)
	T2D Deaths	0.1 (0.1-0.2)	2.5 (1.7-4.0)	0.8 (0.5-1.2)	26.3 (17.5-42.4)	0.2 (0.1-0.3)	8.9 (5.9-14.3)
	CVD Deaths	0.1 (0.1-0.1)	1.3 (0.9-2.0)	18.6 (12.5-28.7)	200 (134-307)	5.1 (3.4-7.8)	67.6 (45.1-104)
Germany	T2D Incidence	8.4 (7.0-10.6)	9.9 (8.3-12.7)	8997 (7476-11383)	24950 (20755-31890)	145 (121-184)	367 (305-469)
	CVD Incidence	3.0 (2.5-4.3)	2.9 (2.5-4.1)	21511 (18138-30486)	17453 (14699-24555)	347 (293-492)	257 (216-361)
	T2D DALYs	4.5 (3.8-6.0)	5.4 (4.6-7.1)	21145 (17967-28270)	40194 (33791-53599)	341 (290-456)	591 (497-789)
	CVD DALYs	2.9 (2.5-4.1)	2.6 (2.2-3.7)	169987 (144429-242400)	79855 (67903-115967)	2743 (2331-3912)	1175 (999-1706)
	T2D Deaths	2.7 (2.3-3.6)	2.6 (2.3-3.7)	548 (467-730)	556 (480-775)	8.8 (7.5-11.8)	8.2 (7.1-11.4)
	CVD Deaths	2.0 (1.7-2.7)	1.8 (1.5-2.4)	6801 (5846-9513)	3496 (2995-4780)	110 (94.3-154)	51.4 (44.1-70.3)
Ghana	T2D Incidence	17.7 (12.4-24.5)	26.5 (19.3-35.4)	1954 (1361-2713)	14917 (10847-19853)	296 (206-411)	910 (661-1211)

Supplementary Data 4. Proportional and absolute T2D and CVD burdens attributable to SSBs in 1990 and 2020 globally, regionally, and nationally (continued).

		Proportional burden % [§]		Absolute burden [§]		Absolute burden per 1M population [§, ƒ]	
Location	Outcome	1990	2020	1990	2020	1990	2020
	CVD Incidence	7.6 (5.0-11.4)	12.4 (8.5-17.9)	2482 (1650-3712)	10430 (7092-15015)	376 (250-563)	636 (432-916)
	T2D DALYs	14.3 (9.8-20.4)	20.4 (14.5-28.1)	7458 (5071-10551)	52196 (36547-71869)	1131 (769-1600)	3183 (2229-4383)
	CVD DALYs	7.3 (4.8-10.9)	10.6 (7.2-15.3)	20632 (13444-30530)	62108 (41932-89506)	3129 (2039-4630)	3787 (2557-5458)
	T2D Deaths	10.8 (7.1-16.1)	15.2 (10.3-22.4)	149 (96.9-220)	1001 (679-1485)	22.6 (14.7-33.4)	61.0 (41.4-90.6)
	CVD Deaths	5.7 (3.6-8.7)	8.0 (5.2-12.1)	637 (405-970)	1939 (1259-2972)	96.6 (61.5-147)	118 (76.8-181)
Greece	T2D Incidence	7.1 (5.6-9.7)	8.7 (6.9-11.6)	1262 (993-1739)	2352 (1846-3182)	170 (134-234)	278 (218-375)
	CVD Incidence	2.0 (1.6-2.9)	1.9 (1.6-2.9)	1010 (824-1481)	1010 (822-1527)	136 (111-199)	119 (97.0-180)
	T2D DALYs	4.2 (3.3-6.0)	4.8 (3.8-6.6)	2168 (1729-3084)	4593 (3664-6408)	292 (233-415)	542 (432-756)
	CVD DALYs	2.2 (1.8-3.3)	2.3 (1.8-3.4)	13099 (10702-19492)	10667 (8687-16163)	1762 (1440-2622)	1259 (1025-1908)
	T2D Deaths	2.1 (1.7-3.1)	2.2 (1.8-3.2)	24.5 (20.3-36.3)	38.3 (31.6-55.7)	3.3 (2.7-4.9)	4.5 (3.7-6.6)
	CVD Deaths	1.4 (1.2-2.0)	1.4 (1.1-1.9)	504 (416-709)	432 (353-612)	67.9 (56.0-95.4)	51.0 (41.6-72.2)
Grenada	T2D Incidence	35.7 (28.7-43.4)	25.8 (19.9-32.8)	76.4 (61.8-93.0)	170 (131-217)	1565 (1267-1905)	2170 (1668-2775)
	CVD Incidence	15.7 (12.1-20.3)	12.7 (9.4-16.9)	56.6 (43.5-73.8)	65.5 (48.8-88.5)	1161 (892-1512)	838 (624-1131)
	T2D DALYs	23.9 (18.8-30.5)	17.9 (13.5-23.5)	400 (315-508)	560 (421-735)	8195 (6446-10421)	7160 (5381-9395)
	CVD DALYs	15.4 (12.0-20.0)	11.0 (8.2-15.1)	541 (420-700)	330 (244-456)	11097 (8617-14336)	4223 (3121-5833)
	T2D Deaths	17.5 (13.4-23.0)	13.4 (9.9-18.2)	11.2 (8.6-14.8)	11.3 (8.3-15.4)	230 (176-303)	144 (106-197)
	CVD Deaths	11.3 (8.6-15.2)	8.5 (6.2-11.9)	21.0 (15.9-28.2)	12.3 (8.9-17.2)	431 (327-578)	157 (113-220)
Guatemala	T2D Incidence	10.9 (8.2-14.5)	26.8 (21.4-33.2)	920 (691-1243)	15138 (12030-18862)	230 (173-311)	1514 (1203-1887)
	CVD Incidence	4.7 (3.5-6.9)	11.3 (8.7-14.6)	517 (381-763)	3829 (2902-5006)	129 (95.4-191)	383 (290-501)
	T2D DALYs	7.9 (6.0-10.6)	18.2 (14.3-23.0)	2198 (1674-2947)	47945 (37582-60377)	550 (419-737)	4796 (3759-6040)
	CVD DALYs	5.4 (4.0-7.5)	11.5 (8.9-14.8)	5869 (4401-8057)	23823 (18616-30519)	1469 (1102-2016)	2383 (1862-3053)
	T2D Deaths	5.7 (4.4-7.8)	13.8 (10.7-17.8)	31.7 (24.2-42.6)	897 (693-1152)	7.9 (6.0-10.7)	89.8 (69.4-115)
	CVD Deaths	3.7 (2.8-5.3)	7.6 (5.8-9.8)	165 (126-235)	774 (597-1008)	41.3 (31.5-58.8)	77.4 (59.7-101)
Guinea	T2D Incidence	4.4 (2.8-6.8)	9.6 (6.2-13.9)	204 (129-319)	1436 (946-2100)	71.6 (45.2-112)	241 (158-352)
	CVD Incidence	1.9 (1.2-3.3)	3.9 (2.4-6.5)	265 (161-471)	976 (607-1655)	93.0 (56.3-165)	164 (102-277)
	T2D DALYs	3.4 (2.1-5.5)	7.1 (4.5-10.6)	889 (541-1424)	5004 (3205-7472)	312 (190-499)	839 (537-1252)
	CVD DALYs	1.6 (1.0-2.8)	3.4 (2.1-5.6)	1594 (966-2802)	6690 (4042-10854)	559 (339-982)	1121 (677-1819)
	T2D Deaths	2.5 (1.5-4.2)	4.9 (3.1-7.7)	21.1 (12.5-35.1)	98.5 (60.9-154)	7.4 (4.4-12.3)	16.5 (10.2-25.7)
	CVD Deaths	1.3 (0.7-2.2)	2.5 (1.5-4.2)	56.2 (33.8-102)	221 (130-373)	19.7 (11.9-35.9)	37.0 (21.8-62.5)

Supplementary Data 4. Proportional and absolute T2D and CVD burdens attributable to SSBs in 1990 and 2020 globally, regionally, and nationally (continued).

		Proportional burden % §		Absolute burden §		Absolute burden per 1M population §, ƒ	
Location	Outcome	1990	2020	1990	2020	1990	2020
Guinea-Bissau	T2D Incidence	0.2 (0.1-0.3)	27.1 (19.2-35.7)	1.3 (0.8-2.8)	834 (592-1110)	3.1 (1.9-6.6)	891 (633-1187)
	CVD Incidence	0.1 (0.0-0.1)	12.7 (8.4-18.3)	1.3 (0.8-2.4)	437 (289-637)	3.1 (1.8-5.7)	467 (309-681)
	T2D DALYs	0.1 (0.1-0.2)	21.7 (14.9-29.3)	6.2 (3.8-12.4)	2901 (1987-3931)	14.6 (8.9-29.2)	3101 (2123-4201)
	CVD DALYs	0.1 (0.0-0.1)	12.0 (7.9-17.6)	13.2 (7.9-23.8)	4237 (2779-6279)	31.1 (18.6-55.9)	4529 (2970-6711)
	T2D Deaths	0.1 (0.1-0.2)	16.8 (11.2-23.9)	0.1 (0.1-0.3)	52.7 (34.9-75.5)	0.3 (0.2-0.6)	56.3 (37.3-80.7)
	CVD Deaths	0.1 (0.0-0.1)	9.5 (6.0-14.2)	0.4 (0.3-0.8)	127 (81.0-193)	1.0 (0.6-1.8)	136 (86.6-206)
Guyana	T2D Incidence	37.6 (27.6-50.2)	28.7 (20.4-40.1)	873 (635-1171)	1500 (1060-2086)	2242 (1632-3009)	3039 (2147-4227)
	CVD Incidence	17.6 (12.2-24.7)	12.7 (8.6-18.6)	359 (244-510)	386 (262-567)	922 (627-1310)	782 (531-1150)
	T2D DALYs	27.0 (19.2-37.1)	19.6 (13.5-28.1)	2914 (2060-4002)	4489 (3077-6406)	7484 (5290-10279)	9094 (6234-12978)
	CVD DALYs	17.4 (12.0-24.6)	12.0 (8.0-17.6)	4668 (3233-6635)	3266 (2177-4839)	11990 (8305-17041)	6618 (4411-9804)
	T2D Deaths	21.3 (14.7-29.8)	15.0 (10.1-21.7)	58.6 (40.7-82.3)	75.5 (50.7-110)	150 (105-211)	153 (103-222)
	CVD Deaths	13.3 (9.1-19.2)	9.1 (6.1-13.8)	150 (103-216)	109 (72.5-165)	386 (265-555)	221 (147-334)
Haiti	T2D Incidence	11.4 (7.6-17.4)	14.6 (9.9-21.2)	1614 (1069-2415)	7669 (5254-11187)	487 (322-728)	1170 (802-1707)
	CVD Incidence	5.3 (3.5-8.7)	6.8 (4.5-10.5)	840 (562-1381)	2242 (1522-3491)	253 (170-416)	342 (232-533)
	T2D DALYs	7.7 (5.2-11.9)	9.7 (6.6-14.6)	6604 (4495-10317)	20312 (13816-30677)	1991 (1355-3111)	3099 (2108-4680)
	CVD DALYs	5.0 (3.4-8.3)	6.2 (4.2-9.6)	11425 (7517-18884)	24026 (16081-37625)	3445 (2267-5694)	3665 (2453-5740)
	T2D Deaths	6.1 (4.1-9.6)	7.1 (4.9-11.4)	147 (99.4-233)	339 (227-542)	44.3 (30.0-70.3)	51.7 (34.7-82.6)
	CVD Deaths	3.9 (2.6-6.5)	4.7 (3.2-7.6)	361 (240-610)	750 (500-1216)	109 (72.2-184)	114 (76.2-186)
Honduras	T2D Incidence	20.2 (16.1-25.6)	32.5 (26.3-39.7)	1122 (885-1425)	9973 (8075-12259)	525 (414-667)	1709 (1384-2101)
	CVD Incidence	9.0 (6.8-12.1)	14.1 (10.9-18.4)	686 (513-932)	3683 (2836-4772)	321 (240-436)	631 (486-818)
	T2D DALYs	14.6 (11.3-18.9)	22.1 (17.4-27.8)	2466 (1911-3207)	20778 (16382-26202)	1153 (894-1500)	3562 (2808-4491)
	CVD DALYs	8.7 (6.5-11.6)	12.1 (9.3-15.8)	5064 (3852-6773)	27641 (20893-36416)	2369 (1801-3168)	4738 (3581-6242)
	T2D Deaths	10.8 (8.3-14.4)	15.6 (12.0-20.3)	31.5 (24.3-42.2)	264 (202-343)	14.7 (11.4-19.8)	45.2 (34.7-58.7)
	CVD Deaths	6.2 (4.7-8.7)	9.3 (6.9-12.3)	158 (118-219)	1024 (766-1372)	74.1 (55.3-103)	176 (131-235)
Hungary	T2D Incidence	7.0 (5.5-10.0)	6.5 (5.1-9.4)	1768 (1376-2496)	2438 (1878-3551)	236 (184-334)	313 (241-456)
	CVD Incidence	2.8 (2.2-3.9)	2.2 (1.7-3.1)	2962 (2293-4167)	2168 (1666-2995)	396 (306-557)	279 (214-385)
	T2D DALYs	4.5 (3.5-6.5)	3.7 (2.9-5.3)	3612 (2829-5286)	4860 (3750-6895)	483 (378-706)	624 (482-886)

Supplementary Data 4. Proportional and absolute T2D and CVD burdens attributable to SSBs in 1990 and 2020 globally, regionally, and nationally (continued).

		Proportional burden % §		Absolute burden §		Absolute burden per 1M population §, ƒ	
Location	Outcome	1990	2020	1990	2020	1990	2020
	CVD DALYs	2.9 (2.3-4.0)	2.1 (1.6-2.9)	31451 (24301-42796)	15540 (11839-21438)	4202 (3247-5718)	1997 (1521-2755)
	T2D Deaths	3.4 (2.6-4.9)	2.5 (1.9-3.6)	60.3 (46.4-86.2)	72.0 (54.7-103)	8.1 (6.2-11.5)	9.2 (7.0-13.2)
	CVD Deaths	2.1 (1.6-2.9)	1.5 (1.2-2.1)	1140 (875-1528)	690 (530-938)	152 (117-204)	88.7 (68.1-120)
Iceland	T2D Incidence	12.9 (10.5-16.5)	14.2 (11.3-18.2)	30.3 (24.5-39.0)	116 (92.1-148)	178 (144-229)	456 (363-583)
	CVD Incidence	4.0 (3.2-5.9)	4.2 (3.5-6.5)	41.4 (33.6-61.8)	57.4 (46.7-85.9)	243 (198-363)	226 (184-339)
	T2D DALYs	7.1 (5.9-9.4)	8.5 (7.0-11.6)	45.5 (37.6-60.3)	158 (128-214)	268 (221-354)	623 (506-844)
	CVD DALYs	3.6 (3.0-5.2)	3.7 (3.0-5.4)	388 (323-566)	277 (226-403)	2279 (1898-3322)	1093 (893-1589)
	T2D Deaths	3.2 (2.7-4.8)	3.3 (2.7-4.7)	0.5 (0.4-0.8)	0.8 (0.7-1.2)	3.1 (2.6-4.5)	3.3 (2.7-4.8)
	CVD Deaths	2.4 (2.0-3.6)	2.3 (1.9-3.4)	15.1 (12.6-22.4)	11.0 (9.0-16.0)	88.5 (74.2-132)	43.6 (35.7-63.3)
India	T2D Incidence	0.7 (0.4-1.6)	0.7 (0.4-1.8)	6201 (3685-14835)	24693 (14541-61068)	13.7 (8.1-32.7)	27.7 (16.3-68.4)
	CVD Incidence	0.2 (0.2-0.4)	0.3 (0.2-0.6)	7373 (4478-13592)	21356 (12776-39254)	16.3 (9.9-30.0)	23.9 (14.3-44.0)
	T2D DALYs	0.5 (0.3-0.9)	0.5 (0.3-1.0)	15780 (9416-31694)	58562 (34524-122617)	34.8 (20.8-69.9)	65.6 (38.7-137)
	CVD DALYs	0.3 (0.2-0.6)	0.3 (0.2-0.6)	54554 (33481-110857)	150172 (90153-283237)	120 (73.8-244)	168 (101-317)
	T2D Deaths	0.3 (0.2-0.6)	0.3 (0.2-0.6)	291 (173-531)	1006 (590-1820)	0.6 (0.4-1.2)	1.1 (0.7-2.0)
	CVD Deaths	0.2 (0.1-0.4)	0.2 (0.1-0.4)	1556 (962-2805)	4707 (2820-8537)	3.4 (2.1-6.2)	5.3 (3.2-9.6)
Indonesia	T2D Incidence	1.3 (1.0-2.7)	2.4 (1.7-4.0)	2387 (1711-4696)	18232 (12773-30637)	25.0 (17.9-49.2)	102 (71.3-171)
	CVD Incidence	0.5 (0.4-0.9)	0.9 (0.7-1.5)	1541 (1125-2973)	8061 (5882-13484)	16.2 (11.8-31.2)	45.0 (32.8-75.2)
	T2D DALYs	0.9 (0.6-1.6)	1.6 (1.2-2.6)	6167 (4519-11595)	39799 (29206-66036)	64.7 (47.4-122)	222 (163-368)
	CVD DALYs	0.6 (0.5-1.4)	1.1 (0.8-2.0)	23772 (17348-50819)	117078 (83597-207261)	249 (182-533)	653 (466-1156)
	T2D Deaths	0.6 (0.5-1.1)	1.2 (0.8-1.9)	113 (83.3-199)	673 (500-1104)	1.2 (0.9-2.1)	3.8 (2.8-6.2)
	CVD Deaths	0.4 (0.3-0.9)	0.8 (0.6-1.3)	589 (432-1165)	3204 (2330-5340)	6.2 (4.5-12.2)	17.9 (13.0-29.8)
Iran, Islamic Rep.	T2D Incidence	8.9 (7.4-11.4)	9.2 (7.6-12.0)	4458 (3691-5690)	28198 (23346-36591)	178 (148-228)	489 (405-634)
	CVD Incidence	4.5 (3.8-6.7)	4.7 (3.9-6.6)	12308 (10271-17857)	32869 (27373-47321)	493 (411-715)	570 (475-820)
	T2D DALYs	6.3 (5.3-8.4)	6.5 (5.5-8.7)	8251 (6931-10938)	48404 (40847-64347)	330 (277-438)	839 (708-1116)
	CVD DALYs	4.6 (3.8-6.4)	4.3 (3.6-5.8)	76183 (63429-107707)	114674 (96594-154829)	3049 (2538-4310)	1988 (1675-2684)
	T2D Deaths	4.8 (4.0-6.7)	4.6 (3.8-6.4)	129 (106-180)	651 (541-903)	5.2 (4.3-7.2)	11.3 (9.4-15.7)
	CVD Deaths	3.6 (3.0-5.2)	3.2 (2.6-4.4)	2438 (2023-3482)	4150 (3453-5755)	97.6 (81.0-139)	71.9 (59.9-99.8)
Iraq	T2D Incidence	20.9 (14.1-30.3)	18.0 (12.0-26.8)	7319 (4812-10638)	41132 (27300-61992)	988 (649-1436)	1968 (1306-2966)
	CVD Incidence	9.4 (6.3-14.8)	8.4 (5.5-13.4)	8554 (5719-13465)	22033 (14604-35356)	1155 (772-1817)	1054 (699-1692)
	T2D DALYs	14.2 (9.5-21.4)	12.6 (8.4-19.2)	16627 (11162-25117)	67754 (44848-103386)	2244 (1507-3390)	3242 (2146-4946)
	CVD DALYs	9.1 (6.1-14.0)	7.3 (4.9-11.4)	57497 (38310-88960)	111612 (74105-175144)	7760 (5171-12007)	5340 (3545-8380)
	T2D Deaths	10.7 (7.1-16.7)	8.9 (6.0-14.1)	315 (208-499)	821 (546-1316)	42.5 (28.1-67.3)	39.3 (26.1-63.0)
	CVD Deaths	7.1 (4.8-11.2)	5.8 (3.9-9.4)	1947 (1310-3053)	3894 (2588-6299)	263 (177-412)	186 (124-301)

Supplementary Data 4. Proportional and absolute T2D and CVD burdens attributable to SSBs in 1990 and 2020 globally, regionally, and nationally (continued).

		Proportional burden % §		Absolute burden §		Absolute burden per 1M population §, ƒ	
Location	Outcome	1990	2020	1990	2020	1990	2020
Ireland	T2D Incidence	13.3 (10.5-16.6)	12.3 (9.6-15.7)	533 (417-667)	1316 (1028-1663)	242 (190-303)	366 (286-462)
	CVD Incidence	4.0 (3.1-5.7)	3.9 (3.1-5.6)	701 (549-1017)	645 (502-927)	319 (249-462)	179 (139-258)
	T2D DALYs	7.0 (5.6-9.2)	7.3 (5.7-9.5)	968 (769-1278)	1735 (1348-2282)	440 (349-581)	482 (375-634)
	CVD DALYs	4.2 (3.3-5.9)	3.5 (2.8-4.9)	8914 (7048-12511)	3416 (2692-4781)	4052 (3204-5687)	950 (748-1329)
	T2D Deaths	4.0 (3.2-5.8)	2.9 (2.3-4.1)	17.2 (13.7-25.0)	12.4 (9.9-17.7)	7.8 (6.2-11.4)	3.4 (2.7-4.9)
	CVD Deaths	3.1 (2.4-4.4)	2.3 (1.8-3.1)	349 (277-502)	136 (108-188)	159 (126-228)	37.8 (30.0-52.4)
Israel	T2D Incidence	20.0 (17.0-24.5)	16.1 (13.5-20.8)	1592 (1346-1963)	4227 (3527-5504)	605 (511-745)	758 (632-986)
	CVD Incidence	8.5 (7.0-11.0)	6.6 (5.5-8.9)	1503 (1237-1948)	1591 (1320-2178)	571 (470-740)	285 (237-390)
	T2D DALYs	13.8 (11.7-17.6)	10.2 (8.5-13.3)	4094 (3448-5242)	7856 (6599-10127)	1555 (1310-1990)	1408 (1183-1815)
	CVD DALYs	9.1 (7.5-11.7)	5.7 (4.7-7.5)	16317 (13537-21064)	6018 (4988-7961)	6196 (5141-7999)	1079 (894-1427)
	T2D Deaths	10.6 (8.8-14.2)	6.5 (5.4-8.7)	100 (83.2-136)	156 (128-208)	38.1 (31.6-51.5)	28.0 (22.9-37.3)
	CVD Deaths	7.5 (6.1-9.9)	4.3 (3.5-5.6)	715 (586-952)	275 (224-359)	272 (223-361)	49.3 (40.2-64.4)
Italy	T2D Incidence	5.1 (4.3-7.2)	5.3 (4.4-7.7)	6058 (5068-8450)	9517 (8029-14005)	140 (117-195)	191 (161-282)
	CVD Incidence	1.8 (1.5-2.5)	1.9 (1.6-2.7)	5557 (4785-8046)	5485 (4665-8150)	128 (110-186)	110 (93.8-164)
	T2D DALYs	2.6 (2.3-3.8)	2.7 (2.3-3.8)	12670 (11042-18206)	16331 (14135-23196)	292 (255-420)	328 (284-466)
	CVD DALYs	1.6 (1.4-2.3)	1.2 (1.1-1.7)	41425 (35847-59475)	21775 (18770-30065)	956 (827-1373)	438 (377-605)
	T2D Deaths	1.7 (1.5-2.6)	1.3 (1.1-1.7)	301 (264-454)	252 (220-329)	6.9 (6.1-10.5)	5.1 (4.4-6.6)
	CVD Deaths	1.1 (0.9-1.5)	0.8 (0.7-1.0)	1661 (1442-2246)	1040 (902-1319)	38.3 (33.3-51.8)	20.9 (18.1-26.5)
Jamaica	T2D Incidence	28.4 (22.7-35.1)	26.4 (20.7-33.0)	1347 (1078-1663)	3354 (2645-4200)	1023 (819-1264)	1652 (1303-2069)
	CVD Incidence	13.8 (10.8-17.5)	12.3 (9.5-15.8)	1070 (831-1353)	1660 (1266-2138)	813 (631-1028)	818 (624-1053)
	T2D DALYs	19.3 (15.0-24.5)	17.3 (13.4-22.2)	6352 (4930-8066)	10760 (8245-13854)	4826 (3746-6128)	5301 (4062-6826)
	CVD DALYs	10.3 (8.0-13.3)	9.1 (7.0-11.8)	4578 (3544-5878)	5095 (3908-6646)	3478 (2693-4466)	2510 (1925-3274)
	T2D Deaths	15.1 (11.5-19.6)	11.8 (8.9-15.5)	183 (139-238)	243 (186-320)	139 (106-181)	120 (91.6-158)
	CVD Deaths	7.4 (5.7-9.7)	6.2 (4.7-8.2)	192 (145-252)	206 (155-272)	146 (111-191)	102 (76.3-134)
Japan	T2D Incidence	7.4 (6.1-10.5)	5.9 (4.9-8.8)	21970 (18216-31601)	28981 (24012-41922)	240 (199-346)	276 (229-399)
	CVD Incidence	2.2 (1.9-3.0)	1.5 (1.2-2.0)	7062 (5959-9580)	8396 (7121-11259)	77.3 (65.2-105)	79.9 (67.8-107)
	T2D DALYs	4.6 (3.9-6.7)	3.4 (2.9-5.1)	30656 (25858-44039)	45508 (38544-68406)	335 (283-482)	433 (367-651)
	CVD DALYs	2.1 (1.8-2.8)	1.5 (1.3-2.1)	68693 (58094-93164)	46030 (38954-65154)	751 (636-1019)	438 (371-620)
	T2D Deaths	2.7 (2.3-3.9)	1.7 (1.4-2.3)	309 (266-446)	158 (136-217)	3.4 (2.9-4.9)	1.5 (1.3-2.1)
	CVD Deaths	1.4 (1.2-1.8)	1.0 (0.8-1.2)	2561 (2179-3287)	1947 (1630-2439)	28.0 (23.8-36.0)	18.5 (15.5-23.2)

Supplementary Data 4. Proportional and absolute T2D and CVD burdens attributable to SSBs in 1990 and 2020 globally, regionally, and nationally (continued).

		Proportional burden % §		Absolute burden §		Absolute burden per 1M population §, ƒ	
Location	Outcome	1990	2020	1990	2020	1990	2020
Jordan	T2D Incidence	20.0 (15.5-25.5)	25.7 (19.8-33.5)	1449 (1118-1866)	17947 (13573-23328)	962 (742-1239)	3089 (2336-4015)
	CVD Incidence	10.6 (7.9-14.3)	13.6 (10.1-18.7)	1556 (1140-2080)	9676 (7170-13238)	1033 (757-1381)	1665 (1234-2278)
	T2D DALYs	14.5 (11.1-19.0)	19.2 (14.6-25.2)	3209 (2440-4200)	25278 (19159-33715)	2131 (1620-2789)	4350 (3297-5802)
	CVD DALYs	10.7 (8.1-14.1)	12.5 (9.4-16.7)	8736 (6562-11513)	24094 (18146-32300)	5800 (4357-7645)	4146 (3123-5559)
	T2D Deaths	11.7 (8.7-16.1)	13.9 (10.5-18.9)	67.8 (50.4-93.5)	283 (213-391)	45.0 (33.5-62.1)	48.7 (36.7-67.4)
	CVD Deaths	8.3 (6.2-11.0)	9.7 (7.3-13.4)	265 (199-352)	750 (558-1024)	176 (132-234)	129 (96.0-176)
Kazakhstan	T2D Incidence	2.9 (1.9-5.1)	5.4 (3.5-8.6)	542 (348-948)	2904 (1900-4632)	55.3 (35.5-96.7)	238 (156-380)
	CVD Incidence	0.9 (0.6-1.6)	1.8 (1.2-2.9)	903 (605-1576)	2079 (1397-3356)	92.1 (61.7-161)	171 (115-275)
	T2D DALYs	1.7 (1.1-2.9)	3.3 (2.2-5.2)	875 (586-1507)	4263 (2840-6780)	89.3 (59.7-154)	350 (233-556)
	CVD DALYs	1.0 (0.7-1.8)	1.5 (1.0-2.4)	9718 (6546-17370)	13227 (8973-21755)	991 (667-1771)	1085 (736-1785)
	T2D Deaths	1.1 (0.8-2.0)	2.0 (1.4-3.3)	9.1 (6.2-16.0)	28.5 (19.3-46.4)	0.9 (0.6-1.6)	2.3 (1.6-3.8)
	CVD Deaths	0.7 (0.5-1.2)	1.1 (0.7-1.7)	315 (212-529)	505 (339-811)	32.1 (21.6-53.9)	41.4 (27.8-66.5)
Kenya	T2D Incidence	5.0 (2.8-8.7)	3.5 (1.9-6.2)	472 (263-820)	1221 (680-2177)	50.1 (27.9-86.9)	45.2 (25.2-80.6)
	CVD Incidence	2.5 (1.4-4.9)	1.7 (0.9-3.5)	890 (473-1747)	1636 (867-3256)	94.4 (50.2-185)	60.6 (32.1-121)
	T2D DALYs	4.2 (2.3-7.6)	2.8 (1.5-5.2)	2402 (1310-4286)	6043 (3309-11153)	255 (139-454)	224 (123-413)
	CVD DALYs	2.2 (1.2-4.1)	1.6 (0.9-3.1)	2516 (1349-4781)	6543 (3567-12427)	267 (143-507)	242 (132-460)
	T2D Deaths	3.1 (1.7-5.9)	2.1 (1.1-4.2)	58.9 (31.2-111)	143 (76.5-289)	6.2 (3.3-11.7)	5.3 (2.8-10.7)
	CVD Deaths	1.6 (0.8-3.1)	1.2 (0.6-2.3)	74.9 (39.6-148)	195 (103-372)	7.9 (4.2-15.7)	7.2 (3.8-13.8)
Kiribati	T2D Incidence	3.9 (2.5-6.9)	7.9 (5.0-12.5)	8.2 (5.1-14.5)	52.4 (33.1-83.4)	224 (140-395)	789 (498-1257)
	CVD Incidence	1.2 (0.7-2.2)	2.3 (1.5-3.9)	2.1 (1.3-3.9)	7.7 (4.9-13.0)	56.4 (36.1-106)	116 (74.2-196)
	T2D DALYs	2.1 (1.3-3.9)	4.2 (2.7-7.2)	34.0 (21.9-62.7)	180 (115-308)	925 (595-1704)	2717 (1732-4636)
	CVD DALYs	1.6 (1.0-3.3)	3.3 (2.1-5.9)	36.7 (23.3-74.3)	151 (96.1-270)	999 (634-2021)	2274 (1448-4076)
	T2D Deaths	1.5 (1.0-2.9)	3.0 (1.9-5.2)	0.7 (0.5-1.4)	3.5 (2.2-6.0)	20.0 (12.8-37.5)	52.7 (33.5-89.7)
	CVD Deaths	1.2 (0.8-2.4)	2.5 (1.6-4.4)	0.9 (0.6-1.8)	3.7 (2.3-6.7)	24.8 (15.7-48.8)	55.9 (35.0-100)
Korea, Rep.	T2D Incidence	3.5 (3.0-6.2)	3.0 (2.6-5.0)	2951 (2503-5294)	9606 (8352-16314)	107 (90.9-192)	227 (197-385)
	CVD Incidence	0.9 (0.8-1.3)	0.8 (0.7-1.1)	991 (876-1404)	1253 (1114-1743)	36.0 (31.8-51.0)	29.6 (26.3-41.1)
	T2D DALYs	2.0 (1.8-3.5)	1.8 (1.6-2.9)	5018 (4396-8747)	14000 (12229-22596)	182 (160-317)	330 (289-533)
	CVD DALYs	0.9 (0.8-1.5)	0.7 (0.6-1.0)	7101 (6265-11337)	5923 (5275-8460)	258 (227-411)	140 (125-200)
	T2D Deaths	1.4 (1.2-2.2)	0.9 (0.8-1.2)	76.6 (67.5-120)	84.3 (74.2-118)	2.8 (2.5-4.4)	2.0 (1.8-2.8)
	CVD Deaths	0.6 (0.5-0.9)	0.5 (0.4-0.6)	202 (179-291)	207 (181-267)	7.3 (6.5-10.6)	4.9 (4.3-6.3)
Kuwait	T2D Incidence	14.3 (9.4-21.5)	36.7 (26.1-49.2)	616 (402-917)	13937 (9855-18844)	521 (340-775)	4454 (3150-6022)
	CVD Incidence	7.3 (4.8-12.5)	20.7 (14.3-30.1)	538 (353-950)	6916 (4741-9945)	455 (298-803)	2210 (1515-3178)

Supplementary Data 4. Proportional and absolute T2D and CVD burdens attributable to SSBs in 1990 and 2020 globally, regionally, and nationally (continued).

		Proportional burden % §		Absolute burden §		Absolute burden per 1M population §, ƒ	
Location	Outcome	1990	2020	1990	2020	1990	2020
	T2D DALYs	10.8 (7.2-16.8)	29.6 (21.0-40.7)	769 (510-1196)	16834 (11942-23032)	650 (431-1011)	5380 (3816-7361)
	CVD DALYs	8.1 (5.3-13.3)	21.6 (14.8-31.7)	2779 (1795-4560)	20045 (13809-29646)	2349 (1518-3855)	6406 (4413-9474)
	T2D Deaths	7.4 (4.9-12.0)	18.5 (12.7-27.0)	7.4 (4.9-12.2)	101 (68.6-148)	6.3 (4.1-10.3)	32.3 (21.9-47.3)
	CVD Deaths	6.3 (4.1-10.7)	16.5 (11.2-24.8)	74.0 (48.5-125)	555 (377-828)	62.5 (41.0-105)	177 (120-265)
Kyrgyz Republic	T2D Incidence	11.8 (8.2-16.8)	16.1 (11.5-22.0)	353 (246-501)	1748 (1247-2411)	152 (106-216)	449 (320-619)
	CVD Incidence	3.7 (2.6-5.7)	5.2 (3.7-7.8)	779 (537-1186)	1576 (1128-2389)	336 (232-511)	405 (290-613)
	T2D DALYs	7.1 (4.9-10.4)	9.9 (7.0-14.4)	614 (431-908)	2661 (1891-3880)	265 (186-392)	683 (486-996)
	CVD DALYs	4.0 (2.8-6.0)	5.1 (3.6-7.6)	8323 (5879-12551)	13416 (9520-20175)	3589 (2535-5412)	3445 (2445-5181)
	T2D Deaths	5.1 (3.4-8.2)	6.9 (4.9-10.9)	7.4 (5.1-12.0)	24.1 (17.2-38.2)	3.2 (2.2-5.2)	6.2 (4.4-9.8)
	CVD Deaths	2.8 (1.9-4.3)	3.7 (2.6-5.7)	273 (189-417)	466 (329-730)	118 (81.7-180)	120 (84.4-187)
Lao PDR	T2D Incidence	1.2 (0.8-2.6)	3.8 (2.8-5.9)	47.1 (31.9-107)	715 (524-1111)	24.7 (16.7-56.1)	169 (124-262)
	CVD Incidence	0.4 (0.3-0.8)	1.4 (1.0-2.1)	29.4 (20.1-52.2)	201 (151-312)	15.4 (10.5-27.3)	47.3 (35.7-73.5)
	T2D DALYs	0.8 (0.5-1.6)	2.5 (1.9-4.2)	189 (130-378)	1562 (1163-2583)	99.0 (67.9-198)	368 (274-609)
	CVD DALYs	0.6 (0.4-1.3)	1.9 (1.4-3.1)	913 (627-1993)	4191 (3129-6881)	478 (328-1043)	988 (737-1622)
	T2D Deaths	0.6 (0.4-1.1)	1.8 (1.3-3.0)	4.2 (2.9-7.6)	25.5 (19.2-44.1)	2.2 (1.5-4.0)	6.0 (4.5-10.4)
	CVD Deaths	0.4 (0.3-0.8)	1.2 (0.9-1.9)	24.3 (16.7-45.5)	111 (82.0-173)	12.7 (8.7-23.8)	26.2 (19.3-40.9)
Latvia	T2D Incidence	4.0 (3.1-6.0)	4.0 (3.1-5.9)	121 (93.8-185)	202 (154-299)	63.6 (49.1-97.1)	135 (104-201)
	CVD Incidence	1.1 (0.9-1.7)	1.0 (0.8-1.5)	283 (223-426)	226 (180-334)	148 (117-223)	152 (121-224)
	T2D DALYs	2.3 (1.8-3.9)	2.1 (1.7-3.3)	232 (185-397)	475 (374-746)	122 (96.8-208)	319 (251-501)
	CVD DALYs	1.3 (1.0-2.2)	1.0 (0.8-1.5)	4031 (3166-6808)	1842 (1447-2740)	2113 (1659-3568)	1237 (971-1839)
	T2D Deaths	1.7 (1.4-2.8)	1.5 (1.1-2.2)	3.3 (2.7-5.4)	8.0 (6.3-12.1)	1.7 (1.4-2.8)	5.4 (4.3-8.1)
	CVD Deaths	0.9 (0.7-1.4)	0.7 (0.6-1.0)	156 (123-230)	85.5 (66.2-122)	81.7 (64.7-120)	57.4 (44.5-82.0)
Lebanon	T2D Incidence	20.1 (16.8-24.7)	22.7 (18.8-28.4)	1316 (1101-1614)	6751 (5556-8363)	915 (766-1123)	1488 (1224-1843)
	CVD Incidence	10.6 (8.6-14.3)	10.1 (8.2-13.4)	2446 (1975-3388)	6160 (5006-8300)	1701 (1373-2356)	1357 (1103-1829)
	T2D DALYs	14.5 (12.0-18.3)	14.8 (12.3-18.5)	3828 (3167-4861)	13224 (10938-16829)	2662 (2203-3381)	2914 (2410-3708)
	CVD DALYs	11.1 (9.1-14.7)	9.1 (7.5-11.8)	14525 (11790-19203)	12825 (10411-16400)	10102 (8200-13355)	2826 (2294-3614)
	T2D Deaths	11.6 (9.4-15.0)	9.7 (7.7-12.9)	81.2 (65.6-106)	179 (139-237)	56.5 (45.6-73.9)	39.5 (30.7-52.3)
	CVD Deaths	8.7 (7.1-11.6)	6.5 (5.2-8.5)	491 (397-664)	514 (412-667)	341 (276-462)	113 (90.8-147)
Lesotho	T2D Incidence	13.5 (9.2-19.8)	29.8 (21.5-39.7)	171 (115-249)	1183 (853-1583)	231 (156-337)	958 (691-1282)

Supplementary Data 4. Proportional and absolute T2D and CVD burdens attributable to SSBs in 1990 and 2020 globally, regionally, and nationally (continued).

		Proportional burden % §		Absolute burden §		Absolute burden per 1M population §, ƒ	
Location	Outcome	1990	2020	1990	2020	1990	2020
	CVD Incidence	6.8 (4.4-11.3)	15.9 (10.9-23.0)	222 (141-364)	746 (507-1089)	300 (190-493)	604 (410-882)
	T2D DALYs	10.5 (6.7-16.2)	23.3 (16.0-32.8)	976 (625-1511)	6785 (4649-9507)	1322 (847-2047)	5495 (3766-7700)
	CVD DALYs	5.3 (3.3-8.7)	14.3 (9.5-21.1)	814 (506-1356)	5140 (3421-7624)	1103 (686-1837)	4163 (2771-6175)
	T2D Deaths	8.7 (5.4-13.7)	19.8 (12.8-29.3)	30.2 (18.6-47.5)	196 (126-284)	41.0 (25.2-64.3)	158 (102-230)
	CVD Deaths	4.0 (2.4-6.7)	11.2 (7.3-17.4)	29.6 (17.9-50.3)	175 (114-275)	40.1 (24.2-68.2)	142 (92.0-223)
Liberia	T2D Incidence	8.2 (5.1-12.8)	29.5 (20.9-39.3)	165 (103-257)	2460 (1731-3305)	178 (111-277)	999 (703-1341)
	CVD Incidence	3.4 (2.1-5.9)	13.6 (9.1-20.3)	186 (111-320)	1322 (867-1975)	200 (120-345)	536 (352-802)
	T2D DALYs	6.2 (3.8-10.2)	23.6 (16.2-32.7)	634 (384-1047)	7582 (5236-10531)	683 (414-1129)	3077 (2125-4274)
	CVD DALYs	3.0 (1.8-5.1)	12.2 (8.1-18.3)	1141 (674-1978)	8301 (5491-12564)	1230 (727-2133)	3369 (2229-5099)
	T2D Deaths	4.6 (2.7-7.9)	17.2 (11.4-25.3)	13.9 (8.3-24.4)	125 (82.6-187)	15.0 (9.0-26.4)	50.9 (33.5-75.8)
	CVD Deaths	2.3 (1.3-4.3)	9.0 (5.8-14.1)	39.8 (23.2-72.8)	259 (165-407)	42.9 (25.0-78.5)	105 (67.0-165)
Libya	T2D Incidence	16.8 (11.8-23.9)	19.4 (13.4-28.1)	809 (570-1155)	7320 (5081-10591)	392 (276-560)	1664 (1155-2407)
	CVD Incidence	8.2 (5.5-12.3)	10.0 (6.8-14.8)	1464 (976-2205)	5022 (3419-7495)	710 (473-1069)	1141 (777-1703)
	T2D DALYs	11.8 (8.3-16.9)	14.4 (10.2-21.0)	1388 (971-1986)	11237 (7931-16325)	673 (471-963)	2554 (1802-3710)
	CVD DALYs	8.9 (6.2-12.9)	10.4 (7.2-15.1)	7525 (5264-10988)	26405 (18536-39075)	3649 (2552-5328)	6001 (4213-8880)
	T2D Deaths	8.8 (6.1-13.3)	10.8 (7.5-16.2)	20.0 (13.7-30.4)	120 (83.3-181)	9.7 (6.6-14.8)	27.3 (18.9-41.2)
	CVD Deaths	6.4 (4.5-9.5)	7.8 (5.4-11.5)	233 (160-343)	811 (562-1204)	113 (77.6-166)	184 (128-274)
Lithuania	T2D Incidence	3.6 (2.8-5.8)	4.8 (3.7-6.9)	132 (101-210)	291 (223-424)	51.1 (39.2-81.3)	133 (102-195)
	CVD Incidence	1.1 (0.8-1.6)	1.2 (0.9-1.8)	363 (282-548)	423 (329-640)	140 (109-212)	194 (151-294)
	T2D DALYs	2.1 (1.6-3.6)	2.6 (2.0-4.2)	206 (158-354)	640 (498-1045)	79.7 (61.0-137)	294 (229-480)
	CVD DALYs	1.2 (0.9-2.0)	1.2 (1.0-2.0)	4287 (3335-7057)	3370 (2584-5358)	1658 (1289-2729)	1547 (1186-2459)
	T2D Deaths	1.5 (1.2-2.7)	1.8 (1.4-2.9)	2.3 (1.8-4.0)	9.8 (7.6-15.6)	0.9 (0.7-1.6)	4.5 (3.5-7.2)
	CVD Deaths	0.9 (0.7-1.3)	0.9 (0.7-1.4)	165 (128-250)	154 (117-231)	63.9 (49.6-96.6)	70.6 (53.7-106)
Luxembourg	T2D Incidence	9.8 (7.3-13.7)	12.5 (9.4-17.4)	53.9 (40.1-76.4)	203 (151-285)	184 (137-260)	411 (306-577)
	CVD Incidence	3.0 (2.2-4.5)	3.9 (2.9-6.0)	39.3 (28.6-59.6)	64.9 (47.7-102)	134 (97.5-203)	131 (96.5-207)
	T2D DALYs	5.4 (4.1-7.8)	7.5 (5.6-10.5)	100 (75.2-144)	290 (216-407)	342 (256-491)	586 (436-824)
	CVD DALYs	2.9 (2.1-4.2)	3.0 (2.2-4.4)	649 (480-952)	349 (257-523)	2213 (1637-3247)	706 (520-1058)
	T2D Deaths	2.9 (2.2-4.3)	2.9 (2.1-4.1)	1.7 (1.3-2.5)	2.0 (1.5-2.9)	5.9 (4.3-8.6)	4.1 (3.1-5.9)
	CVD Deaths	2.0 (1.4-2.8)	1.9 (1.4-2.6)	25.6 (18.9-36.2)	14.6 (10.9-20.5)	87.4 (64.6-123)	29.6 (22.0-41.5)

Supplementary Data 4. Proportional and absolute T2D and CVD burdens attributable to SSBs in 1990 and 2020 globally, regionally, and nationally (continued).

		Proportional burden % §		Absolute burden §		Absolute burden per 1M population §, ƒ	
Location	Outcome	1990	2020	1990	2020	1990	2020
Macedonia, FYR	T2D Incidence	2.6 (1.9-4.7)	15.1 (11.5-20.0)	118 (86.2-214)	1779 (1356-2373)	90.3 (66.0-164)	1097 (836-1463)
	CVD Incidence	1.1 (0.8-1.7)	6.0 (4.4-8.2)	111 (82.3-176)	1032 (766-1425)	84.9 (63.1-135)	636 (472-879)
	T2D DALYs	1.6 (1.2-2.9)	9.2 (6.8-12.6)	232 (173-417)	3565 (2649-4853)	178 (132-319)	2198 (1633-2991)
	CVD DALYs	1.0 (0.7-1.6)	4.8 (3.5-6.5)	1298 (969-2095)	8247 (6125-11390)	994 (742-1605)	5083 (3775-7021)
	T2D Deaths	1.2 (0.9-2.0)	6.6 (4.8-9.5)	4.3 (3.2-7.2)	61.4 (45.1-87.8)	3.3 (2.5-5.5)	37.8 (27.8-54.1)
	CVD Deaths	0.7 (0.5-1.1)	3.6 (2.6-5.0)	46.7 (34.5-73.8)	349 (256-492)	35.8 (26.4-56.5)	215 (158-303)
Madagascar	T2D Incidence	16.7 (11.3-23.8)	19.6 (13.8-27.7)	1027 (688-1454)	4374 (3060-6181)	203 (136-287)	322 (225-455)
	CVD Incidence	8.2 (5.2-13.1)	9.2 (5.8-14.2)	1874 (1192-3014)	4563 (2930-6946)	370 (235-594)	336 (216-511)
	T2D DALYs	14.7 (9.7-21.3)	16.2 (11.1-23.3)	6869 (4514-10059)	18847 (12805-27396)	1355 (890-1984)	1387 (943-2017)
	CVD DALYs	8.5 (5.4-13.4)	9.8 (6.4-15.1)	13762 (8730-21977)	36009 (23012-56029)	2714 (1722-4334)	2651 (1694-4124)
	T2D Deaths	11.5 (7.4-17.6)	12.6 (8.4-18.8)	174 (112-268)	380 (253-576)	34.3 (22.1-52.9)	28.0 (18.6-42.4)
	CVD Deaths	6.4 (4.1-10.5)	7.5 (4.9-11.8)	408 (256-664)	1000 (634-1547)	80.4 (50.5-131)	73.6 (46.7-114)
Malawi	T2D Incidence	7.3 (4.8-11.4)	3.1 (2.0-5.4)	346 (226-541)	369 (235-658)	81.8 (53.4-128)	42.3 (26.9-75.4)
	CVD Incidence	3.9 (2.5-6.9)	1.5 (1.0-2.8)	637 (405-1099)	458 (294-835)	151 (95.8-260)	52.4 (33.7-95.6)
	T2D DALYs	6.5 (4.2-10.3)	2.6 (1.6-4.5)	2722 (1752-4306)	2374 (1522-4192)	644 (414-1018)	272 (174-480)
	CVD DALYs	3.9 (2.5-6.5)	1.5 (1.0-2.8)	3550 (2336-5995)	3215 (2034-5883)	839 (552-1417)	368 (233-674)
	T2D Deaths	5.2 (3.3-8.7)	2.0 (1.2-3.4)	73.0 (47.1-122)	57.8 (36.0-102)	17.3 (11.1-28.8)	6.6 (4.1-11.7)
	CVD Deaths	2.9 (1.8-5.0)	1.1 (0.7-2.0)	105 (68.8-182)	93.3 (58.9-167)	24.8 (16.3-43.0)	10.7 (6.8-19.1)
Malaysia	T2D Incidence	6.1 (4.5-8.5)	5.7 (4.2-8.2)	1672 (1225-2357)	6991 (5136-10029)	176 (129-248)	316 (232-454)
	CVD Incidence	2.0 (1.5-2.9)	2.1 (1.6-3.5)	755 (577-1119)	2337 (1794-3851)	79.3 (60.6-118)	106 (81.2-174)
	T2D DALYs	3.7 (2.8-5.3)	3.8 (2.9-5.9)	3379 (2533-4841)	10862 (8142-16602)	355 (266-509)	491 (368-751)
	CVD DALYs	2.5 (1.9-3.8)	2.6 (2.0-4.3)	11304 (8505-17338)	25858 (19537-43103)	1187 (893-1821)	1170 (884-1950)
	T2D Deaths	2.5 (1.9-3.8)	2.7 (2.1-4.3)	57.7 (43.7-87.7)	119 (91.1-188)	6.1 (4.6-9.2)	5.4 (4.1-8.5)
	CVD Deaths	1.7 (1.3-2.6)	1.8 (1.4-3.0)	327 (250-499)	753 (578-1239)	34.4 (26.3-52.4)	34.0 (26.1-56.0)
Maldives	T2D Incidence	25.7 (16.8-35.1)	12.2 (6.9-19.3)	50.6 (33.1-69.8)	157 (88.3-247)	537 (351-740)	385 (216-605)
	CVD Incidence	10.1 (6.0-15.7)	5.1 (2.8-8.7)	29.0 (17.3-45.8)	47.8 (26.1-80.5)	308 (183-485)	117 (63.9-197)
	T2D DALYs	21.7 (13.5-30.7)	9.5 (5.3-15.3)	215 (134-305)	263 (148-423)	2285 (1426-3234)	645 (362-1037)
	CVD DALYs	12.4 (7.6-18.8)	5.7 (3.2-9.4)	586 (357-895)	382 (211-636)	6220 (3788-9490)	935 (516-1557)
	T2D Deaths	18.9 (11.2-27.7)	6.4 (3.5-11.0)	5.1 (3.1-7.4)	3.1 (1.7-5.3)	54.1 (32.6-78.8)	7.5 (4.1-12.9)
	CVD Deaths	10.1 (6.0-15.5)	3.7 (2.0-6.4)	16.7 (9.9-25.9)	11.1 (6.0-19.3)	178 (105-275)	27.2 (14.7-47.3)
Mali	T2D Incidence	5.8 (3.6-9.9)	7.8 (4.7-13.5)	615 (373-1049)	3598 (2127-6157)	171 (104-292)	424 (251-726)

Supplementary Data 4. Proportional and absolute T2D and CVD burdens attributable to SSBs in 1990 and 2020 globally, regionally, and nationally (continued).

		Proportional burden % §		Absolute burden §		Absolute burden per 1M population §, ƒ	
Location	Outcome	1990	2020	1990	2020	1990	2020
	CVD Incidence	2.7 (1.6-4.9)	3.4 (2.0-6.1)	434 (260-802)	1149 (688-2041)	121 (72.4-224)	135 (81.1-241)
	T2D DALYs	4.5 (2.8-7.9)	5.9 (3.6-10.2)	1976 (1220-3446)	8957 (5388-15477)	551 (340-960)	1056 (635-1825)
	CVD DALYs	2.4 (1.4-4.6)	2.9 (1.8-5.3)	2180 (1325-4311)	5614 (3370-10406)	607 (369-1201)	662 (397-1227)
	T2D Deaths	3.3 (2.1-6.0)	4.0 (2.4-7.2)	35.5 (21.5-63.6)	118 (70.7-214)	9.9 (6.0-17.7)	13.9 (8.3-25.2)
	CVD Deaths	1.8 (1.1-3.4)	2.1 (1.3-3.9)	69.9 (41.8-128)	178 (107-320)	19.5 (11.6-35.7)	21.0 (12.6-37.7)
Malta	T2D Incidence	11.5 (8.6-16.1)	20.7 (15.8-27.1)	65.3 (48.1-90.9)	368 (284-485)	261 (192-363)	1029 (794-1357)
	CVD Incidence	4.4 (3.3-6.6)	6.5 (4.8-9.4)	46.8 (34.3-71.2)	101 (74.6-148)	187 (137-284)	282 (209-414)
	T2D DALYs	6.3 (4.7-9.2)	11.6 (8.9-15.9)	156 (117-228)	627 (479-864)	625 (466-912)	1753 (1340-2417)
	CVD DALYs	4.3 (3.2-6.5)	6.3 (4.7-8.9)	854 (638-1297)	869 (652-1243)	3411 (2547-5178)	2431 (1824-3478)
	T2D Deaths	4.0 (3.0-6.1)	5.9 (4.5-8.6)	4.0 (3.0-6.1)	7.8 (5.9-11.4)	15.9 (11.9-24.3)	21.9 (16.5-31.8)
	CVD Deaths	3.1 (2.3-4.9)	4.2 (3.1-5.9)	33.1 (24.6-51.5)	36.7 (27.6-52.6)	132 (98.3-206)	103 (77.2-147)
Marshall Islands	T2D Incidence	5.1 (3.3-8.3)	13.7 (9.5-19.7)	6.0 (3.9-9.9)	58.3 (40.2-84.9)	237 (157-393)	1598 (1101-2325)
	CVD Incidence	1.3 (0.9-2.2)	3.8 (2.7-5.8)	0.9 (0.6-1.5)	5.3 (3.7-8.1)	36.7 (25.5-61.5)	146 (102-221)
	T2D DALYs	2.6 (1.8-4.3)	7.1 (5.1-11.3)	15.5 (10.7-25.3)	164 (115-255)	614 (427-1007)	4485 (3154-6976)
	CVD DALYs	1.9 (1.3-3.3)	5.4 (3.7-8.6)	23.3 (15.9-41.8)	150 (103-237)	927 (632-1663)	4106 (2834-6490)
	T2D Deaths	1.7 (1.2-2.9)	5.0 (3.5-8.5)	0.3 (0.2-0.5)	2.6 (1.8-4.3)	10.5 (7.4-18.5)	70.5 (48.2-117)
	CVD Deaths	1.3 (0.9-2.5)	4.2 (2.9-6.8)	0.6 (0.4-1.1)	3.7 (2.6-6.0)	23.3 (16.2-43.4)	102 (71.5-165)
Mauritania	T2D Incidence	12.0 (7.8-18.1)	32.9 (23.0-44.9)	171 (113-259)	1499 (1042-2063)	191 (125-288)	642 (446-884)
	CVD Incidence	5.3 (3.3-8.7)	16.1 (10.5-24.6)	271 (171-445)	1598 (1024-2417)	301 (191-495)	685 (439-1036)
	T2D DALYs	9.3 (5.8-14.3)	24.3 (16.2-34.7)	744 (471-1159)	5190 (3484-7492)	827 (523-1288)	2223 (1493-3210)
	CVD DALYs	4.7 (2.9-8.0)	13.4 (8.6-20.3)	1992 (1222-3335)	8724 (5569-13415)	2214 (1358-3706)	3738 (2386-5747)
	T2D Deaths	7.0 (4.3-11.4)	17.6 (11.1-26.8)	18.1 (11.1-29.6)	115 (72.6-175)	20.1 (12.3-32.9)	49.4 (31.1-74.9)
	CVD Deaths	3.7 (2.3-6.4)	10.1 (6.2-16.2)	68.7 (40.8-120)	314 (194-500)	76.3 (45.4-133)	135 (83.0-214)
Mauritius	T2D Incidence	60.6 (52.9-68.1)	47.7 (38.8-57.2)	1623 (1403-1835)	5320 (4287-6410)	2543 (2199-2875)	5520 (4448-6651)
	CVD Incidence	29.9 (22.4-38.1)	22.9 (16.6-31.3)	854 (649-1088)	1197 (859-1627)	1337 (1017-1705)	1242 (892-1688)
	T2D DALYs	51.5 (41.8-60.6)	39.2 (30.1-49.8)	6904 (5610-8116)	23524 (18040-29788)	10816 (8790-12716)	24409 (18719-30909)
	CVD DALYs	35.4 (27.5-43.7)	27.6 (21.0-35.5)	17514 (13618-21612)	12815 (9721-16569)	27440 (21336-33860)	13297 (10087-17192)
	T2D Deaths	46.5 (36.3-56.3)	33.1 (24.7-43.7)	166 (131-202)	579 (429-763)	260 (204-316)	601 (445-792)

Supplementary Data 4. Proportional and absolute T2D and CVD burdens attributable to SSBs in 1990 and 2020 globally, regionally, and nationally (continued).

		Proportional burden % §		Absolute burden §		Absolute burden per 1M population §, ƒ	
Location	Outcome	1990	2020	1990	2020	1990	2020
	CVD Deaths	28.8 (21.2-37.1)	20.6 (14.8-28.2)	577 (426-744)	427 (307-585)	904 (667-1166)	443 (319-607)
Mexico	T2D Incidence	32.3 (28.7-36.6)	30.0 (26.4-35.0)	70019 (61867-79437)	169425 (148014-197396)	1694 (1497-1922)	2007 (1753-2338)
	CVD Incidence	15.0 (13.0-17.8)	13.5 (11.5-16.7)	26959 (23307-32603)	60850 (51694-75000)	652 (564-789)	721 (612-888)
	T2D DALYs	22.0 (19.5-25.8)	20.2 (17.6-24.0)	226579 (200584-264040)	597753 (520777-713712)	5483 (4854-6389)	7081 (6169-8455)
	CVD DALYs	13.9 (12.2-16.5)	13.4 (11.7-16.5)	148815 (130823-176870)	389257 (338284-477184)	3601 (3166-4280)	4611 (4007-5653)
	T2D Deaths	16.5 (14.4-19.6)	14.9 (12.9-18.3)	4591 (4025-5472)	11904 (10276-14588)	111 (97.4-132)	141 (122-173)
	CVD Deaths	9.6 (8.3-11.5)	9.2 (8.0-11.4)	4894 (4261-5890)	13128 (11329-16271)	118 (103-143)	156 (134-193)
Micronesia, Fed. Sts.	T2D Incidence	4.3 (2.9-7.0)	12.1 (8.4-17.1)	9.1 (6.1-14.9)	72.6 (50.0-103)	213 (142-349)	1072 (739-1519)
	CVD Incidence	1.2 (0.8-1.9)	3.6 (2.6-5.7)	2.7 (1.9-4.3)	11.5 (8.0-18.1)	62.3 (44.5-101)	170 (119-267)
	T2D DALYs	2.3 (1.6-3.8)	6.7 (4.8-9.9)	30.2 (21.6-51.3)	210 (149-309)	706 (505-1201)	3103 (2204-4572)
	CVD DALYs	1.7 (1.2-3.1)	5.2 (3.6-8.1)	65.4 (45.5-120)	287 (199-448)	1529 (1065-2803)	4240 (2936-6617)
	T2D Deaths	1.6 (1.1-2.6)	4.9 (3.5-7.5)	0.6 (0.4-1.0)	3.7 (2.6-5.9)	14.4 (10.3-24.4)	54.9 (38.9-86.5)
	CVD Deaths	1.2 (0.9-2.1)	3.9 (2.7-6.2)	1.7 (1.2-2.9)	7.3 (5.2-11.8)	40.1 (28.3-68.8)	109 (76.3-174)
Moldova	T2D Incidence	5.2 (3.6-8.4)	3.4 (2.3-5.6)	304 (207-484)	372 (256-623)	108 (73.8-173)	117 (80.2-195)
	CVD Incidence	1.7 (1.2-2.7)	1.0 (0.7-1.7)	504 (351-801)	378 (270-657)	180 (125-286)	118 (84.6-206)
	T2D DALYs	3.2 (2.2-5.2)	2.0 (1.4-3.4)	525 (357-863)	667 (469-1145)	187 (127-308)	209 (147-359)
	CVD DALYs	1.8 (1.2-2.8)	1.1 (0.7-1.8)	5572 (3882-9006)	3364 (2389-5825)	1986 (1383-3209)	1054 (749-1826)
	T2D Deaths	2.4 (1.6-4.3)	1.4 (1.0-2.5)	6.6 (4.5-12.1)	7.2 (5.1-13.0)	2.3 (1.6-4.3)	2.2 (1.6-4.1)
	CVD Deaths	1.3 (0.9-2.1)	0.8 (0.6-1.4)	211 (146-341)	134 (93.2-229)	75.3 (52.1-121)	42.1 (29.2-71.7)
Mongolia	T2D Incidence	14.1 (9.6-20.0)	13.9 (9.5-19.8)	146 (98.5-206)	720 (489-1023)	138 (93.2-195)	353 (240-502)
	CVD Incidence	4.5 (3.0-6.6)	4.9 (3.4-7.2)	352 (243-517)	694 (473-1036)	334 (230-490)	340 (232-508)
	T2D DALYs	8.1 (5.5-11.7)	8.7 (5.9-12.7)	243 (166-349)	1216 (835-1784)	230 (157-330)	596 (409-874)
	CVD DALYs	4.8 (3.2-6.9)	5.1 (3.5-7.7)	3083 (2092-4482)	4595 (3109-6943)	2919 (1980-4243)	2252 (1524-3403)
	T2D Deaths	5.4 (3.7-8.2)	6.1 (4.1-9.4)	3.0 (2.0-4.5)	11.4 (7.8-17.8)	2.8 (1.9-4.3)	5.6 (3.8-8.7)
	CVD Deaths	3.4 (2.3-5.1)	3.6 (2.5-5.5)	102 (69.5-151)	138 (93.5-206)	96.5 (65.8-143)	67.5 (45.8-101)
Montenegro	T2D Incidence	3.5 (1.4-8.2)	5.4 (2.2-12.5)	47.3 (19.5-111)	137 (57.4-322)	116 (47.9-272)	288 (121-677)
	CVD Incidence	1.6 (0.7-3.8)	2.2 (0.9-5.2)	41.1 (17.0-100)	93.9 (39.4-217)	101 (41.8-247)	197 (82.9-457)
	T2D DALYs	2.1 (0.9-5.1)	3.1 (1.3-7.5)	75.3 (31.6-178)	255 (106-601)	185 (77.8-438)	535 (222-1264)
	CVD DALYs	1.6 (0.7-3.8)	1.9 (0.8-4.5)	382 (158-920)	814 (349-1968)	941 (388-2263)	1711 (735-4138)
	T2D Deaths	1.5 (0.7-3.7)	2.1 (0.9-5.2)	1.1 (0.4-2.6)	3.8 (1.6-9.0)	2.6 (1.1-6.5)	7.9 (3.3-19.0)
	CVD Deaths	1.1 (0.5-2.6)	1.4 (0.6-3.3)	12.8 (5.4-29.8)	34.3 (14.6-81.2)	31.6 (13.2-73.4)	72.1 (30.6-171)

Supplementary Data 4. Proportional and absolute T2D and CVD burdens attributable to SSBs in 1990 and 2020 globally, regionally, and nationally (continued).

		Proportional burden % §		Absolute burden §		Absolute burden per 1M population §, ƒ	
Location	Outcome	1990	2020	1990	2020	1990	2020
Morocco	T2D Incidence	14.0 (9.4-20.1)	16.9 (11.6-24.0)	5789 (3893-8290)	39094 (26903-55752)	471 (317-675)	1625 (1118-2317)
	CVD Incidence	6.5 (4.4-10.1)	7.8 (5.2-11.9)	10141 (6829-15965)	28828 (19448-44006)	826 (556-1300)	1198 (808-1829)
	T2D DALYs	9.8 (6.6-14.3)	11.9 (8.2-17.4)	9051 (6124-13154)	64107 (43982-92635)	737 (499-1071)	2664 (1828-3850)
	CVD DALYs	6.9 (4.7-10.2)	7.3 (4.9-11.0)	78912 (52664-116774)	162989 (109588-245834)	6425 (4288-9508)	6774 (4555-10217)
	T2D Deaths	7.2 (4.9-11.1)	8.6 (5.9-13.7)	110 (73.3-169)	625 (428-990)	9.0 (6.0-13.8)	26.0 (17.8-41.1)
	CVD Deaths	5.3 (3.6-7.9)	5.8 (3.9-8.9)	2601 (1760-3927)	6040 (4031-9330)	212 (143-320)	251 (168-388)
Mozambique	T2D Incidence	4.3 (2.4-7.7)	8.8 (4.9-14.2)	322 (179-578)	2379 (1337-3840)	58.5 (32.6-105)	171 (95.9-275)
	CVD Incidence	2.3 (1.3-4.8)	4.0 (2.2-7.2)	605 (325-1267)	1895 (1046-3536)	110 (59.0-230)	136 (75.0-254)
	T2D DALYs	3.9 (2.1-7.6)	7.1 (4.0-12.3)	2331 (1278-4457)	12364 (7017-21113)	424 (232-810)	887 (503-1514)
	CVD DALYs	1.7 (0.9-3.2)	3.2 (1.8-5.7)	1828 (987-3448)	8818 (4897-15709)	332 (179-627)	633 (351-1127)
	T2D Deaths	3.1 (1.7-6.1)	5.6 (3.1-9.9)	60.6 (33.4-119)	267 (150-469)	11.0 (6.1-21.6)	19.2 (10.8-33.6)
	CVD Deaths	1.3 (0.7-2.4)	2.4 (1.3-4.3)	58.8 (32.2-113)	267 (150-487)	10.7 (5.8-20.6)	19.2 (10.7-34.9)
Myanmar	T2D Incidence	1.1 (0.7-2.3)	8.3 (5.9-12.4)	717 (475-1450)	19758 (13970-29429)	33.8 (22.4-68.3)	557 (394-830)
	CVD Incidence	0.4 (0.3-0.7)	3.1 (2.2-4.8)	330 (221-572)	4799 (3350-7404)	15.5 (10.4-26.9)	135 (94.4-209)
	T2D DALYs	0.8 (0.5-1.4)	5.6 (4.0-8.6)	3264 (2181-6055)	52623 (36965-80848)	154 (103-285)	1484 (1042-2279)
	CVD DALYs	0.6 (0.4-1.1)	3.7 (2.6-5.6)	7801 (5283-15636)	67265 (47102-101143)	367 (249-736)	1896 (1328-2851)
	T2D Deaths	0.6 (0.4-1.0)	4.1 (2.9-6.5)	72.1 (49.1-122)	986 (697-1568)	3.4 (2.3-5.8)	27.8 (19.6-44.2)
	CVD Deaths	0.4 (0.3-0.7)	2.4 (1.7-3.8)	208 (138-365)	2013 (1427-3120)	9.8 (6.5-17.2)	56.8 (40.2-87.9)
Namibia	T2D Incidence	38.5 (28.4-50.8)	44.7 (33.2-58.0)	480 (354-631)	1767 (1306-2282)	758 (560-997)	1300 (960-1678)
	CVD Incidence	22.0 (15.2-31.8)	24.3 (16.6-34.6)	672 (457-972)	1471 (1008-2120)	1062 (722-1536)	1082 (741-1559)
	T2D DALYs	31.0 (21.2-43.7)	34.1 (23.7-47.1)	2935 (2006-4149)	8553 (5923-11766)	4641 (3172-6560)	6290 (4355-8651)
	CVD DALYs	20.0 (13.3-29.1)	22.2 (15.0-32.1)	4236 (2773-6181)	9510 (6437-13779)	6698 (4384-9772)	6993 (4733-10132)
	T2D Deaths	26.6 (17.4-39.7)	27.8 (18.2-40.7)	88.1 (57.9-130)	236 (152-342)	139 (91.6-205)	173 (112-252)
	CVD Deaths	16.2 (10.5-24.8)	17.2 (11.1-25.8)	143 (92.4-220)	328 (212-494)	227 (146-348)	241 (156-363)
Nepal	T2D Incidence	1.2 (0.6-2.8)	6.1 (3.1-11.4)	243 (114-539)	4624 (2341-8728)	27.2 (12.7-60.3)	263 (133-497)
	CVD Incidence	0.4 (0.2-1.0)	2.2 (1.1-4.8)	237 (115-522)	2777 (1375-5954)	26.5 (12.9-58.4)	158 (78.3-339)
	T2D DALYs	0.8 (0.4-1.8)	3.9 (2.0-7.9)	587 (281-1298)	10568 (5315-21003)	65.6 (31.4-145)	602 (303-1197)
	CVD DALYs	0.5 (0.2-1.0)	2.3 (1.1-4.8)	1745 (842-3755)	18346 (8989-38823)	195 (94.1-420)	1045 (512-2212)
	T2D Deaths	0.6 (0.3-1.3)	2.5 (1.2-5.4)	9.5 (4.6-20.9)	156 (75.3-335)	1.1 (0.5-2.3)	8.9 (4.3-19.1)
	CVD Deaths	0.4 (0.2-0.8)	1.7 (0.9-3.8)	50.9 (24.2-111)	600 (295-1302)	5.7 (2.7-12.4)	34.2 (16.8-74.2)

Supplementary Data 4. Proportional and absolute T2D and CVD burdens attributable to SSBs in 1990 and 2020 globally, regionally, and nationally (continued).

		Proportional burden % §		Absolute burden §		Absolute burden per 1M population §, ƒ	
Location	Outcome	1990	2020	1990	2020	1990	2020
Netherlands	T2D Incidence	11.8 (10.2-14.7)	1.1 (0.5-3.3)	2602 (2252-3231)	469 (198-1376)	233 (202-289)	34.9 (14.7-102)
	CVD Incidence	4.3 (3.7-5.8)	0.4 (0.2-1.0)	3583 (3102-4937)	283 (120-776)	321 (278-442)	21.1 (9.0-57.8)
	T2D DALYs	6.2 (5.5-8.4)	0.6 (0.3-1.8)	5774 (5124-7875)	757 (328-2185)	517 (459-705)	56.4 (24.4-163)
	CVD DALYs	4.2 (3.7-5.7)	0.3 (0.1-0.7)	29826 (26173-40533)	952 (413-2505)	2671 (2344-3630)	70.9 (30.7-187)
	T2D Deaths	3.7 (3.3-5.2)	0.3 (0.1-0.7)	134 (121-188)	8.5 (3.7-22.2)	12.0 (10.8-16.9)	0.6 (0.3-1.7)
	CVD Deaths	2.9 (2.6-4.0)	0.2 (0.1-0.4)	1132 (1001-1535)	40.8 (17.8-104)	101 (89.7-137)	3.0 (1.3-7.8)
New Zealand	T2D Incidence	4.1 (3.1-6.8)	6.1 (4.7-9.1)	214 (163-361)	848 (650-1259)	92.3 (70.6-156)	237 (181-352)
	CVD Incidence	1.4 (1.1-2.2)	2.1 (1.7-3.3)	303 (237-471)	530 (419-825)	131 (102-203)	148 (117-230)
	T2D DALYs	2.2 (1.8-3.7)	3.7 (2.9-5.7)	368 (291-614)	1308 (1022-2007)	159 (126-265)	365 (285-560)
	CVD DALYs	1.3 (1.0-2.1)	1.8 (1.4-2.7)	2240 (1763-3479)	1999 (1564-2992)	968 (761-1503)	558 (437-835)
	T2D Deaths	1.5 (1.2-2.5)	2.1 (1.7-3.3)	6.1 (4.9-9.8)	12.9 (10.2-20.2)	2.7 (2.1-4.2)	3.6 (2.9-5.6)
	CVD Deaths	1.0 (0.8-1.4)	1.1 (0.9-1.6)	84.5 (67.4-121)	79.5 (63.2-113)	36.5 (29.1-52.1)	22.2 (17.6-31.6)
Nicaragua	T2D Incidence	13.1 (9.4-18.0)	31.8 (25.0-40.1)	649 (463-896)	7410 (5810-9391)	360 (257-497)	1822 (1428-2309)
	CVD Incidence	5.7 (4.1-8.4)	14.8 (11.0-19.5)	346 (244-509)	2575 (1917-3418)	192 (135-283)	633 (471-840)
	T2D DALYs	9.2 (6.7-13.0)	23.0 (17.5-29.4)	1511 (1091-2118)	16880 (12840-21524)	839 (606-1176)	4150 (3157-5292)
	CVD DALYs	5.7 (4.1-8.0)	14.3 (10.7-18.9)	1921 (1383-2696)	12138 (9033-15999)	1067 (768-1497)	2984 (2221-3934)
	T2D Deaths	6.7 (4.8-9.8)	17.2 (12.8-22.8)	23.2 (16.6-33.1)	236 (175-311)	12.9 (9.2-18.4)	57.9 (43.0-76.6)
	CVD Deaths	3.8 (2.7-5.7)	10.1 (7.5-13.6)	59.0 (42.0-87.3)	416 (307-564)	32.8 (23.4-48.5)	102 (75.5-139)
Niger	T2D Incidence	6.4 (3.9-10.7)	0.0 (0.0-0.0)	321 (191-538)	0.6 (0.4-0.9)	95.5 (56.9-160)	0.1 (0.0-0.1)
	CVD Incidence	2.9 (1.7-5.5)	0.0 (0.0-0.0)	348 (207-674)	0.3 (0.2-0.6)	104 (61.5-201)	0.0 (0.0-0.1)
	T2D DALYs	5.0 (3.0-8.7)	0.0 (0.0-0.0)	978 (580-1694)	1.4 (0.9-2.2)	291 (173-504)	0.2 (0.1-0.2)
	CVD DALYs	2.5 (1.4-4.5)	0.0 (0.0-0.0)	1451 (845-2636)	1.5 (0.9-2.5)	432 (251-784)	0.2 (0.1-0.3)
	T2D Deaths	3.7 (2.2-6.6)	0.0 (0.0-0.0)	17.6 (10.4-31.7)	0.0 (0.0-0.0)	5.2 (3.1-9.4)	0.0 (0.0-0.0)
	CVD Deaths	1.9 (1.1-3.6)	0.0 (0.0-0.0)	45.7 (26.0-88.0)	0.0 (0.0-0.1)	13.6 (7.7-26.2)	0.0 (0.0-0.0)
Nigeria	T2D Incidence	1.6 (1.0-3.1)	15.5 (10.7-22.1)	1092 (687-2059)	36645 (25263-52304)	25.7 (16.1-48.4)	387 (267-553)
	CVD Incidence	0.7 (0.4-1.4)	7.7 (5.0-11.8)	1431 (868-2754)	30861 (19883-47343)	33.7 (20.4-64.8)	326 (210-501)
	T2D DALYs	1.2 (0.8-2.4)	11.8 (7.8-17.3)	4920 (3061-9315)	113577 (74716-166643)	116 (72.0-219)	1201 (790-1762)
	CVD DALYs	0.6 (0.4-1.1)	6.6 (4.2-10.1)	8557 (5225-16046)	164695 (104288-252385)	201 (123-377)	1741 (1103-2668)
	T2D Deaths	0.9 (0.6-1.7)	8.4 (5.2-13.0)	120 (73.5-220)	2229 (1392-3462)	2.8 (1.7-5.2)	23.6 (14.7-36.6)
	CVD Deaths	0.5 (0.3-0.9)	4.8 (3.0-7.7)	300 (179-557)	5362 (3320-8581)	7.1 (4.2-13.1)	56.7 (35.1-90.7)
Norway	T2D Incidence	7.8 (6.2-10.6)	8.4 (6.8-11.7)	591 (475-817)	1075 (861-1499)	189 (152-261)	258 (207-360)

Supplementary Data 4. Proportional and absolute T2D and CVD burdens attributable to SSBs in 1990 and 2020 globally, regionally, and nationally (continued).

		Proportional burden % §		Absolute burden §		Absolute burden per 1M population §, ƒ	
Location	Outcome	1990	2020	1990	2020	1990	2020
	CVD Incidence	2.1 (1.8-3.1)	2.4 (2.0-4.0)	593 (499-874)	574 (472-960)	190 (160-280)	138 (113-231)
	T2D DALYs	4.3 (3.6-6.2)	4.9 (4.0-7.4)	879 (725-1265)	1547 (1251-2302)	281 (232-404)	372 (301-553)
	CVD DALYs	2.2 (1.8-3.4)	2.0 (1.6-3.1)	6469 (5412-10102)	2014 (1699-3135)	2068 (1730-3230)	484 (408-753)
	T2D Deaths	2.1 (1.8-3.2)	2.0 (1.7-3.0)	10.2 (8.7-15.6)	11.7 (9.9-17.1)	3.2 (2.8-5.0)	2.8 (2.4-4.1)
	CVD Deaths	1.5 (1.3-2.3)	1.3 (1.1-1.8)	266 (226-397)	83.3 (70.6-120)	85.1 (72.2-127)	20.0 (17.0-28.7)
Oman	T2D Incidence	6.3 (4.0-10.5)	15.4 (10.2-23.9)	166 (103-276)	2388 (1570-3741)	197 (122-328)	638 (419-999)
	CVD Incidence	3.0 (2.0-5.2)	8.2 (5.3-13.5)	227 (145-394)	1841 (1203-3032)	269 (172-468)	492 (321-810)
	T2D DALYs	4.1 (2.7-7.1)	11.3 (7.4-17.9)	340 (216-588)	3649 (2413-5807)	403 (256-698)	975 (645-1551)
	CVD DALYs	3.1 (1.9-5.2)	7.5 (4.9-12.2)	1666 (1049-2867)	6203 (4055-10082)	1979 (1247-3405)	1657 (1083-2693)
	T2D Deaths	3.2 (2.0-5.7)	7.9 (5.1-12.9)	7.2 (4.7-12.9)	54.5 (34.6-88.6)	8.6 (5.5-15.3)	14.6 (9.2-23.7)
	CVD Deaths	2.5 (1.6-4.2)	5.7 (3.7-9.4)	52.5 (34.0-90.0)	191 (125-311)	62.3 (40.4-107)	51.1 (33.4-83.1)
Pakistan	T2D Incidence	17.3 (10.7-26.1)	16.8 (10.4-26.1)	19579 (12169-29871)	95324 (58982-148930)	387 (240-590)	781 (483-1221)
	CVD Incidence	6.1 (3.4-10.9)	7.3 (4.1-12.5)	24578 (13263-43059)	65873 (37396-113009)	486 (262-851)	540 (307-926)
	T2D DALYs	11.8 (6.9-19.0)	11.8 (6.8-18.7)	56659 (32916-89931)	226722 (129196-361304)	1119 (650-1777)	1858 (1059-2961)
	CVD DALYs	6.5 (3.7-11.1)	7.8 (4.5-13.3)	149142 (83887-252813)	478994 (280279-811209)	2947 (1657-4995)	3926 (2297-6649)
	T2D Deaths	8.5 (4.7-14.7)	8.2 (4.6-14.0)	1142 (623-1961)	3626 (1998-6183)	22.6 (12.3-38.7)	29.7 (16.4-50.7)
	CVD Deaths	5.0 (2.8-8.7)	5.9 (3.3-10.0)	4612 (2571-8018)	13687 (7611-23194)	91.1 (50.8-158)	112 (62.4-190)
Palestine	T2D Incidence	10.2 (6.9-14.5)	14.5 (10.0-20.6)	269 (180-380)	2530 (1737-3596)	315 (211-445)	963 (661-1369)
	CVD Incidence	5.1 (3.4-7.9)	7.4 (4.9-11.2)	455 (302-708)	1833 (1211-2779)	533 (354-830)	698 (461-1058)
	T2D DALYs	6.8 (4.6-9.9)	10.3 (6.9-14.7)	791 (523-1154)	4421 (2949-6306)	928 (613-1353)	1683 (1123-2400)
	CVD DALYs	4.8 (3.2-7.2)	6.9 (4.6-10.1)	3066 (2052-4542)	7227 (4819-10623)	3595 (2406-5326)	2751 (1834-4043)
	T2D Deaths	5.2 (3.5-7.9)	7.4 (5.0-11.0)	20.8 (13.8-31.4)	74.0 (50.2-109)	24.4 (16.1-36.8)	28.2 (19.1-41.4)
	CVD Deaths	3.6 (2.4-5.5)	5.2 (3.4-7.8)	111 (72.9-169)	246 (163-365)	130 (85.5-198)	93.7 (62.0-139)
Panama	T2D Incidence	32.5 (25.6-40.4)	37.7 (30.4-46.4)	1090 (872-1369)	5446 (4388-6742)	828 (663-1041)	1935 (1559-2395)
	CVD Incidence	15.4 (11.8-20.1)	17.8 (13.6-23.2)	874 (672-1142)	2698 (2064-3503)	664 (511-868)	959 (734-1245)
	T2D DALYs	23.7 (18.6-30.3)	26.8 (20.8-34.3)	2887 (2268-3700)	14413 (11225-18547)	2194 (1724-2812)	5121 (3988-6590)
	CVD DALYs	13.7 (10.7-18.1)	15.8 (12.2-20.7)	5156 (4008-6857)	10458 (8099-13629)	3919 (3046-5212)	3716 (2878-4843)
	T2D Deaths	17.0 (13.1-22.5)	18.9 (14.5-25.0)	49.6 (38.3-65.4)	232 (178-307)	37.7 (29.1-49.7)	82.6 (63.3-109)
	CVD Deaths	10.1 (7.7-13.8)	11.1 (8.4-14.8)	194 (148-262)	398 (300-528)	148 (112-200)	142 (106-188)

Supplementary Data 4. Proportional and absolute T2D and CVD burdens attributable to SSBs in 1990 and 2020 globally, regionally, and nationally (continued).

		Proportional burden % §		Absolute burden §		Absolute burden per 1M population §, ƒ	
Location	Outcome	1990	2020	1990	2020	1990	2020
Papua New Guinea	T2D Incidence	3.7 (2.4-7.1)	0.0 (0.0-0.0)	273 (173-515)	0.0 (0.0-0.0)	125 (79.6-237)	0.0 (0.0-0.0)
	CVD Incidence	1.1 (0.8-2.2)	0.0 (0.0-0.0)	72.4 (48.7-137)	0.0 (0.0-0.0)	33.3 (22.4-62.9)	0.0 (0.0-0.0)
	T2D DALYs	2.0 (1.4-4.0)	0.0 (0.0-0.0)	1042 (703-2016)	0.0 (0.0-0.0)	479 (323-927)	0.0 (0.0-0.0)
	CVD DALYs	1.4 (0.9-3.0)	0.0 (0.0-0.0)	1101 (725-2345)	0.0 (0.0-0.0)	506 (333-1078)	0.0 (0.0-0.0)
	T2D Deaths	1.4 (1.0-2.9)	0.0 (0.0-0.0)	21.0 (14.3-40.8)	0.0 (0.0-0.0)	9.6 (6.6-18.7)	0.0 (0.0-0.0)
	CVD Deaths	1.1 (0.7-2.3)	0.0 (0.0-0.0)	27.8 (18.7-56.9)	0.0 (0.0-0.0)	12.8 (8.6-26.2)	0.0 (0.0-0.0)
Paraguay	T2D Incidence	17.5 (13.0-23.2)	23.0 (16.9-31.1)	847 (627-1127)	5199 (3839-7066)	413 (305-549)	1178 (870-1601)
	CVD Incidence	7.8 (5.5-10.8)	10.7 (7.5-15.0)	467 (329-647)	1506 (1053-2140)	228 (160-315)	341 (239-485)
	T2D DALYs	12.2 (9.0-16.7)	15.4 (11.1-21.5)	2369 (1742-3235)	16078 (11497-22567)	1154 (848-1576)	3644 (2606-5115)
	CVD DALYs	7.4 (5.3-10.2)	9.9 (7.0-14.2)	4989 (3579-6976)	13375 (9431-19123)	2430 (1743-3397)	3031 (2138-4334)
	T2D Deaths	8.7 (6.2-12.1)	11.5 (8.0-16.2)	46.1 (32.9-63.9)	353 (248-502)	22.5 (16.0-31.1)	80.0 (56.3-114)
	CVD Deaths	5.2 (3.6-7.3)	7.1 (5.0-10.3)	172 (121-243)	480 (335-690)	83.8 (59.2-118)	109 (75.9-156)
Peru	T2D Incidence	21.3 (15.1-30.4)	18.7 (13.0-27.2)	3270 (2319-4706)	12305 (8621-17792)	296 (210-425)	550 (385-795)
	CVD Incidence	10.1 (7.1-15.1)	9.9 (6.9-15.0)	3699 (2548-5459)	8561 (5906-13013)	334 (230-493)	382 (264-581)
	T2D DALYs	15.4 (10.8-22.6)	13.5 (9.4-20.1)	9288 (6479-13579)	31444 (21786-46841)	840 (586-1227)	1404 (973-2092)
	CVD DALYs	10.7 (7.5-15.6)	8.9 (6.2-13.2)	26095 (18176-38145)	36202 (24730-53992)	2359 (1643-3448)	1617 (1104-2411)
	T2D Deaths	11.5 (8.0-17.3)	9.8 (6.7-15.1)	184 (126-277)	588 (393-899)	16.6 (11.4-25.0)	26.3 (17.6-40.2)
	CVD Deaths	7.1 (4.9-10.6)	5.8 (4.0-8.9)	807 (556-1211)	1202 (831-1854)	73.0 (50.3-109)	53.7 (37.1-82.8)
Philippines	T2D Incidence	9.1 (7.4-11.1)	8.0 (6.5-10.1)	6558 (5289-8056)	18928 (15363-24011)	219 (177-269)	286 (232-363)
	CVD Incidence	3.9 (3.1-5.0)	3.4 (2.7-4.4)	2943 (2390-3795)	7066 (5776-9257)	98.3 (79.8-127)	107 (87.3-140)
	T2D DALYs	7.3 (5.9-9.1)	5.8 (4.6-7.6)	21178 (17173-26836)	61328 (49374-81019)	707 (573-896)	926 (746-1224)
	CVD DALYs	5.9 (4.7-7.6)	4.6 (3.7-6.1)	76935 (61256-98753)	156076 (125424-206861)	2569 (2045-3297)	2358 (1895-3125)
	T2D Deaths	5.2 (4.2-6.8)	4.3 (3.5-5.8)	379 (306-494)	1251 (1025-1685)	12.6 (10.2-16.5)	18.9 (15.5-25.5)
	CVD Deaths	3.7 (3.0-4.8)	3.1 (2.5-4.1)	1847 (1482-2369)	4095 (3313-5424)	61.7 (49.5-79.1)	61.9 (50.0-81.9)
Poland	T2D Incidence	4.1 (3.4-6.2)	3.9 (3.3-5.9)	3435 (2882-5225)	5799 (4927-8814)	134 (113-204)	191 (162-290)
	CVD Incidence	1.6 (1.4-2.3)	1.5 (1.2-2.1)	3963 (3334-5790)	3137 (2640-4623)	155 (130-226)	103 (87.0-152)
	T2D DALYs	2.5 (2.1-3.7)	2.2 (1.9-3.4)	6456 (5494-9633)	10723 (9116-16291)	252 (215-377)	353 (300-537)
	CVD DALYs	1.7 (1.5-2.7)	1.2 (1.0-1.7)	55298 (46969-85171)	24055 (20074-34587)	2161 (1836-3329)	793 (661-1140)
	T2D Deaths	1.9 (1.6-2.9)	1.5 (1.2-2.2)	103 (87.5-159)	142 (117-211)	4.0 (3.4-6.2)	4.7 (3.8-6.9)
	CVD Deaths	1.2 (1.0-1.7)	0.8 (0.7-1.2)	1931 (1615-2780)	1038 (846-1440)	75.5 (63.1-109)	34.2 (27.9-47.5)
Portugal	T2D Incidence	8.7 (7.4-11.3)	8.9 (7.6-11.8)	1795 (1535-2366)	4224 (3595-5630)	255 (218-337)	506 (431-675)
	CVD Incidence	2.3 (2.0-3.2)	2.3 (2.0-3.2)	1057 (916-1457)	739 (637-1027)	150 (130-207)	88.6 (76.4-123)

Supplementary Data 4. Proportional and absolute T2D and CVD burdens attributable to SSBs in 1990 and 2020 globally, regionally, and nationally (continued).

		Proportional burden % §		Absolute burden §		Absolute burden per 1M population §, ƒ	
Location	Outcome	1990	2020	1990	2020	1990	2020
	T2D DALYs	4.8 (4.2-6.6)	4.8 (4.1-6.4)	4252 (3694-5912)	7065 (6130-9556)	605 (526-841)	847 (735-1146)
	CVD DALYs	2.3 (2.0-3.1)	2.3 (2.0-3.1)	13569 (11827-18113)	6689 (5810-9360)	1931 (1683-2578)	802 (697-1122)
	T2D Deaths	3.0 (2.7-4.6)	2.1 (1.8-2.9)	90.8 (78.9-137)	80.8 (71.0-113)	12.9 (11.2-19.5)	9.7 (8.5-13.5)
	CVD Deaths	1.6 (1.4-2.1)	1.4 (1.2-1.8)	544 (473-733)	281 (244-377)	77.5 (67.3-104)	33.7 (29.2-45.2)
Qatar	T2D Incidence	18.7 (12.2-28.6)	21.2 (13.9-32.4)	193 (125-300)	4316 (2831-6712)	620 (400-960)	1812 (1189-2818)
	CVD Incidence	11.5 (7.4-18.4)	13.2 (8.4-21.2)	180 (115-293)	1693 (1076-2786)	576 (369-938)	711 (452-1170)
	T2D DALYs	14.4 (9.5-22.4)	17.6 (11.7-27.0)	337 (219-511)	4864 (3266-7518)	1079 (703-1637)	2042 (1371-3156)
	CVD DALYs	11.1 (7.1-17.3)	12.7 (8.1-20.2)	947 (611-1482)	3037 (1927-4889)	3035 (1958-4751)	1275 (809-2052)
	T2D Deaths	10.8 (7.3-16.7)	12.4 (8.1-19.5)	6.0 (3.9-9.2)	44.2 (29.1-68.5)	19.1 (12.6-29.6)	18.5 (12.2-28.7)
	CVD Deaths	8.8 (5.7-13.8)	10.2 (6.5-16.2)	25.8 (16.6-40.2)	74.5 (47.5-119)	82.8 (53.1-129)	31.3 (19.9-50.1)
Romania	T2D Incidence	11.0 (8.7-14.3)	11.3 (8.7-14.8)	3741 (2971-4909)	5564 (4340-7339)	235 (186-308)	365 (284-481)
	CVD Incidence	3.9 (3.1-5.3)	3.6 (2.8-4.8)	6939 (5520-9371)	6720 (5278-8881)	435 (346-588)	440 (346-582)
	T2D DALYs	7.1 (5.6-9.8)	6.7 (5.2-8.9)	7336 (5798-9973)	10580 (8295-14074)	460 (364-626)	693 (544-922)
	CVD DALYs	4.0 (3.1-5.3)	3.4 (2.7-4.6)	73666 (58209-99067)	55170 (43348-74358)	4622 (3652-6216)	3616 (2841-4874)
	T2D Deaths	5.6 (4.4-7.8)	4.7 (3.6-6.6)	107 (84.1-149)	122 (94.6-172)	6.7 (5.3-9.3)	8.0 (6.2-11.3)
	CVD Deaths	2.9 (2.3-4.0)	2.4 (1.9-3.3)	2821 (2193-3887)	2400 (1850-3250)	177 (138-244)	157 (121-213)
Russian Federation	T2D Incidence	3.5 (2.7-5.8)	7.1 (5.5-10.2)	6066 (4638-9880)	27857 (21461-40041)	58.5 (44.8-95.4)	249 (192-357)
	CVD Incidence	1.3 (1.0-1.9)	2.6 (2.0-3.7)	19558 (15352-29424)	52156 (41080-74529)	189 (148-284)	465 (367-665)
	T2D DALYs	2.2 (1.7-3.5)	4.0 (3.1-5.7)	9720 (7541-15789)	57683 (45263-84812)	93.8 (72.8-152)	515 (404-757)
	CVD DALYs	1.2 (1.0-1.9)	2.4 (1.9-3.5)	187746 (148118-290754)	332257 (258264-482915)	1812 (1430-2806)	2965 (2305-4310)
	T2D Deaths	1.6 (1.2-2.5)	2.7 (2.1-3.9)	107 (83.3-165)	989 (769-1460)	1.0 (0.8-1.6)	8.8 (6.9-13.0)
	CVD Deaths	0.9 (0.7-1.3)	1.7 (1.3-2.5)	6831 (5349-9968)	13017 (10156-18767)	65.9 (51.6-96.2)	116 (90.6-167)
Rwanda	T2D Incidence	3.7 (2.2-7.0)	66.5 (59.9-71.8)	146 (86.4-276)	6881 (6183-7503)	50.0 (29.5-94.3)	1061 (953-1157)
	CVD Incidence	1.8 (1.1-3.6)	46.5 (38.8-53.3)	214 (123-431)	10314 (8438-12237)	73.1 (42.1-147)	1590 (1301-1887)
	T2D DALYs	3.2 (1.9-6.1)	61.4 (54.0-67.4)	1372 (809-2599)	39811 (34356-44721)	469 (276-888)	6139 (5297-6896)
	CVD DALYs	1.9 (1.1-3.8)	43.1 (35.7-50.0)	1843 (1042-3708)	50497 (40191-60563)	630 (356-1267)	7786 (6197-9338)
	T2D Deaths	2.6 (1.5-4.9)	53.8 (45.5-60.6)	36.2 (21.1-69.8)	1092 (917-1275)	12.4 (7.2-23.9)	168 (141-197)
	CVD Deaths	1.4 (0.8-2.8)	36.2 (28.4-43.6)	54.1 (30.5-107)	1749 (1361-2189)	18.5 (10.4-36.5)	270 (210-337)
Samoa	T2D Incidence	8.5 (5.6-13.3)	25.5 (17.8-36.1)	33.6 (21.9-52.3)	270 (189-383)	442 (288-687)	2567 (1791-3633)

Supplementary Data 4. Proportional and absolute T2D and CVD burdens attributable to SSBs in 1990 and 2020 globally, regionally, and nationally (continued).

		Proportional burden % [§]		Absolute burden [§]		Absolute burden per 1M population [§, ƒ]	
Location	Outcome	1990	2020	1990	2020	1990	2020
	CVD Incidence	2.3 (1.5-3.7)	6.8 (4.5-10.4)	8.9 (5.8-14.6)	45.5 (29.2-69.2)	117 (76.0-192)	432 (277-657)
	T2D DALYs	4.2 (2.7-6.9)	13.4 (9.0-20.0)	88.4 (57.1-145)	678 (455-1013)	1163 (751-1900)	6438 (4320-9617)
	CVD DALYs	2.9 (1.8-5.0)	9.1 (5.9-13.9)	130 (81.6-224)	755 (492-1149)	1704 (1073-2940)	7170 (4674-10912)
	T2D Deaths	2.8 (1.9-4.7)	8.7 (5.7-13.3)	1.7 (1.1-2.7)	10.6 (7.0-16.1)	21.8 (14.1-36.1)	101 (66.1-153)
	CVD Deaths	2.0 (1.3-3.4)	6.3 (4.1-9.8)	3.6 (2.3-6.1)	21.1 (13.7-32.6)	47.7 (30.1-80.4)	200 (130-309)
Sao Tome and Principe	T2D Incidence	9.3 (6.0-13.6)	17.2 (11.5-24.6)	8.9 (5.7-13.1)	61.4 (40.9-87.9)	181 (117-267)	598 (399-857)
	CVD Incidence	3.8 (2.3-6.1)	7.7 (4.8-12.2)	11.6 (7.0-18.9)	41.5 (25.7-65.7)	236 (143-386)	404 (250-640)
	T2D DALYs	7.0 (4.4-10.6)	13.6 (8.9-20.0)	22.6 (14.3-34.3)	139 (91.8-203)	461 (291-699)	1352 (894-1982)
	CVD DALYs	3.2 (2.0-5.3)	6.7 (4.2-10.7)	56.1 (34.2-91.7)	237 (147-373)	1144 (698-1870)	2306 (1427-3639)
	T2D Deaths	4.9 (3.0-8.0)	8.8 (5.5-14.2)	0.4 (0.2-0.6)	1.5 (0.9-2.4)	7.4 (4.5-12.3)	14.3 (9.0-23.0)
	CVD Deaths	2.4 (1.5-4.0)	4.9 (3.0-8.1)	2.0 (1.2-3.3)	7.5 (4.7-12.2)	40.2 (24.1-67.7)	73.3 (45.5-119)
Saudi Arabia	T2D Incidence	14.2 (10.1-20.5)	23.4 (16.3-33.6)	3587 (2534-5158)	45473 (31318-64526)	450 (318-647)	1895 (1305-2689)
	CVD Incidence	7.0 (4.9-10.8)	13.3 (9.1-20.0)	3975 (2750-6032)	28323 (19281-42183)	499 (345-757)	1180 (803-1758)
	T2D DALYs	10.2 (7.2-15.3)	19.1 (13.2-27.5)	5690 (3986-8403)	66027 (45675-95345)	714 (500-1055)	2751 (1903-3973)
	CVD DALYs	7.2 (5.0-10.7)	13.9 (9.5-20.6)	28459 (19912-42399)	170722 (116987-254749)	3572 (2500-5322)	7114 (4875-10616)
	T2D Deaths	7.8 (5.3-12.1)	15.1 (10.6-22.2)	92.1 (62.7-140)	712 (491-1048)	11.6 (7.9-17.6)	29.7 (20.5-43.7)
	CVD Deaths	5.5 (3.8-8.6)	11.6 (8.1-17.5)	866 (597-1351)	4445 (3088-6594)	109 (74.9-170)	185 (129-275)
Senegal	T2D Incidence	28.2 (20.3-37.5)	45.3 (34.8-55.6)	2115 (1507-2830)	15498 (11772-19061)	662 (472-886)	1975 (1500-2429)
	CVD Incidence	14.9 (9.8-21.8)	24.9 (17.7-33.9)	2402 (1567-3539)	8722 (6181-11917)	752 (491-1108)	1111 (788-1519)
	T2D DALYs	23.0 (16.1-31.7)	36.2 (26.8-46.5)	7382 (5154-10217)	45350 (33280-58097)	2310 (1613-3198)	5779 (4241-7403)
	CVD DALYs	13.3 (8.9-19.8)	21.3 (15.0-29.3)	16881 (11193-25158)	56411 (39668-78977)	5283 (3503-7874)	7188 (5055-10064)
	T2D Deaths	17.5 (11.7-26.2)	27.5 (19.2-37.9)	150 (99.9-228)	810 (562-1119)	47.0 (31.3-71.2)	103 (71.6-143)
	CVD Deaths	10.4 (6.6-16.2)	16.7 (10.9-24.3)	577 (368-893)	2017 (1339-2957)	181 (115-279)	257 (171-377)
Serbia	T2D Incidence	4.4 (2.1-8.9)	2.9 (1.4-6.3)	1171 (552-2328)	1231 (573-2639)	179 (84.5-357)	179 (83.3-384)
	CVD Incidence	1.9 (0.9-3.9)	1.0 (0.5-2.0)	1338 (629-2708)	996 (475-2042)	205 (96.4-415)	145 (69.0-297)
	T2D DALYs	2.8 (1.4-6.1)	1.7 (0.8-3.6)	2332 (1106-5119)	2550 (1196-5498)	357 (170-785)	371 (174-799)
	CVD DALYs	1.6 (0.8-3.2)	0.8 (0.4-1.7)	12975 (6212-26138)	7059 (3397-14587)	1989 (952-4006)	1026 (494-2120)
	T2D Deaths	2.1 (1.0-4.4)	1.2 (0.6-2.6)	40.2 (19.4-86.5)	43.8 (21.1-96.9)	6.2 (3.0-13.3)	6.4 (3.1-14.1)
	CVD Deaths	1.1 (0.5-2.3)	0.6 (0.3-1.3)	491 (234-996)	328 (158-678)	75.3 (35.9-153)	47.7 (23.0-98.5)

Supplementary Data 4. Proportional and absolute T2D and CVD burdens attributable to SSBs in 1990 and 2020 globally, regionally, and nationally (continued).

		Proportional burden % §		Absolute burden §		Absolute burden per 1M population §, ƒ	
Location	Outcome	1990	2020	1990	2020	1990	2020
Seychelles	T2D Incidence	22.9 (18.5-28.4)	25.4 (20.1-31.7)	27.0 (21.8-33.5)	178 (140-224)	715 (577-886)	2593 (2048-3275)
	CVD Incidence	9.8 (7.5-13.3)	11.8 (9.0-15.8)	17.8 (13.3-24.5)	38.5 (29.0-52.1)	471 (353-649)	562 (424-760)
	T2D DALYs	17.8 (14.0-22.8)	20.1 (15.9-25.8)	67.6 (53.4-87.4)	342 (272-441)	1790 (1414-2312)	4989 (3974-6427)
	CVD DALYs	12.6 (9.8-16.4)	14.4 (11.2-18.7)	301 (235-396)	417 (326-541)	7971 (6207-10472)	6089 (4754-7895)
	T2D Deaths	13.9 (10.4-19.2)	15.5 (11.8-20.9)	1.3 (0.9-1.7)	3.8 (2.9-5.1)	33.5 (24.8-46.1)	55.1 (41.9-74.4)
	CVD Deaths	8.9 (6.6-12.4)	10.3 (7.8-14.0)	9.8 (7.3-13.5)	12.6 (9.6-17.0)	260 (192-358)	184 (140-248)
Sierra Leone	T2D Incidence	8.5 (5.6-13.2)	29.4 (20.9-39.1)	277 (179-432)	3541 (2504-4711)	140 (90.3-218)	912 (645-1213)
	CVD Incidence	3.7 (2.3-6.3)	13.5 (8.9-19.8)	388 (243-669)	2397 (1569-3482)	196 (122-338)	617 (404-897)
	T2D DALYs	6.4 (4.1-10.2)	22.7 (16.0-31.1)	937 (602-1493)	10077 (7014-13890)	473 (304-753)	2595 (1806-3576)
	CVD DALYs	3.2 (2.0-5.7)	12.2 (8.1-18.0)	2689 (1661-4817)	17625 (11680-26398)	1357 (838-2430)	4538 (3007-6797)
	T2D Deaths	4.6 (2.8-8.1)	16.4 (10.8-24.2)	20.0 (12.0-34.5)	169 (110-247)	10.1 (6.1-17.4)	43.5 (28.3-63.5)
	CVD Deaths	2.5 (1.5-4.5)	9.3 (5.9-14.6)	92.8 (55.6-170)	578 (372-907)	46.8 (28.0-85.9)	149 (95.8-233)
Singapore	T2D Incidence	9.8 (7.5-15.2)	8.8 (6.8-13.5)	828 (630-1293)	2426 (1865-3707)	391 (298-611)	499 (383-762)
	CVD Incidence	2.9 (2.3-4.6)	2.8 (2.2-4.9)	225 (175-365)	560 (441-963)	106 (82.6-172)	115 (90.7-198)
	T2D DALYs	5.5 (4.3-8.5)	5.9 (4.6-9.4)	1058 (829-1657)	3096 (2377-4871)	501 (392-784)	636 (488-1001)
	CVD DALYs	3.4 (2.7-5.5)	3.1 (2.5-5.4)	3153 (2469-5129)	3066 (2407-5280)	1491 (1168-2426)	630 (495-1085)
	T2D Deaths	3.3 (2.6-5.4)	2.7 (2.2-4.4)	11.4 (9.1-18.7)	4.3 (3.4-7.0)	5.4 (4.3-8.9)	0.9 (0.7-1.4)
	CVD Deaths	2.4 (1.9-3.8)	2.2 (1.7-3.5)	93.4 (74.5-148)	97.6 (76.7-159)	44.2 (35.3-69.8)	20.1 (15.8-32.7)
Slovak Republic	T2D Incidence	7.7 (6.0-10.6)	10.1 (7.9-13.3)	706 (550-976)	1681 (1306-2212)	201 (157-278)	387 (300-509)
	CVD Incidence	2.7 (2.2-3.9)	3.4 (2.7-4.6)	1099 (870-1574)	1482 (1170-2050)	313 (248-448)	341 (269-471)
	T2D DALYs	4.6 (3.6-6.7)	6.0 (4.8-8.3)	1338 (1057-1940)	2894 (2269-3981)	381 (301-552)	666 (522-916)
	CVD DALYs	3.0 (2.4-4.4)	3.2 (2.5-4.4)	14344 (11362-21024)	11936 (9311-16683)	4084 (3235-5986)	2746 (2142-3837)
	T2D Deaths	3.4 (2.7-4.9)	4.1 (3.2-5.9)	22.8 (17.8-34.0)	33.5 (25.8-48.0)	6.5 (5.1-9.7)	7.7 (5.9-11.0)
	CVD Deaths	2.2 (1.8-3.2)	2.3 (1.8-3.4)	536 (423-782)	521 (402-757)	153 (121-223)	120 (92.4-174)
Slovenia	T2D Incidence	5.8 (4.3-8.6)	8.1 (6.0-11.3)	251 (184-376)	536 (396-747)	174 (127-260)	321 (237-447)
	CVD Incidence	2.4 (1.8-3.5)	3.1 (2.3-4.6)	277 (208-410)	455 (334-674)	192 (144-283)	272 (200-403)
	T2D DALYs	3.6 (2.7-5.7)	4.7 (3.5-7.0)	434 (322-678)	970 (717-1432)	300 (223-469)	580 (429-856)
	CVD DALYs	2.1 (1.6-3.2)	2.2 (1.6-3.2)	2018 (1507-3092)	1353 (984-1998)	1395 (1042-2139)	809 (588-1195)
	T2D Deaths	2.6 (1.9-4.1)	2.9 (2.1-4.4)	6.8 (5.0-10.5)	12.2 (8.8-18.6)	4.7 (3.5-7.3)	7.3 (5.2-11.1)
	CVD Deaths	1.5 (1.1-2.3)	1.5 (1.1-2.2)	78.9 (57.4-117)	63.3 (45.7-93.9)	54.6 (39.7-80.7)	37.8 (27.3-56.1)

Supplementary Data 4. Proportional and absolute T2D and CVD burdens attributable to SSBs in 1990 and 2020 globally, regionally, and nationally (continued).

		Proportional burden % §		Absolute burden §		Absolute burden per 1M population §, ƒ	
Location	Outcome	1990	2020	1990	2020	1990	2020
Solomon Islands	T2D Incidence	15.6 (10.0-22.7)	12.6 (7.9-19.2)	71.6 (46.5-104)	277 (173-421)	536 (348-781)	810 (507-1230)
	CVD Incidence	4.8 (3.1-8.0)	3.8 (2.4-6.4)	30.1 (19.1-50.4)	65.7 (41.4-110)	225 (143-377)	192 (121-323)
	T2D DALYs	9.0 (5.8-14.2)	7.7 (4.8-12.4)	329 (206-511)	1055 (662-1679)	2462 (1542-3818)	3086 (1937-4912)
	CVD DALYs	6.4 (4.0-10.7)	5.3 (3.3-8.9)	671 (422-1132)	1370 (855-2313)	5017 (3159-8464)	4007 (2501-6765)
	T2D Deaths	6.8 (4.3-11.0)	5.7 (3.6-9.3)	7.1 (4.5-11.3)	20.2 (12.6-33.5)	53.4 (33.6-84.8)	59.2 (36.7-98.1)
	CVD Deaths	4.8 (3.0-8.1)	3.9 (2.4-6.6)	17.6 (11.0-29.7)	34.9 (22.0-59.9)	132 (82.2-222)	102 (64.3-175)
South Africa	T2D Incidence	31.8 (26.2-38.7)	27.6 (22.1-34.6)	14249 (11697-17237)	46938 (37498-58774)	781 (641-945)	1258 (1005-1575)
	CVD Incidence	17.2 (13.5-21.8)	14.6 (11.4-19.2)	18811 (14601-23960)	30897 (24083-40407)	1031 (801-1314)	828 (645-1083)
	T2D DALYs	26.3 (21.2-33.2)	20.9 (16.3-27.4)	68340 (54831-85671)	207391 (160766-271569)	3747 (3006-4697)	5558 (4308-7278)
	CVD DALYs	18.7 (15.0-23.5)	13.1 (10.2-17.3)	97386 (77793-122204)	145120 (112629-190477)	5340 (4265-6701)	3889 (3018-5105)
	T2D Deaths	20.5 (15.9-26.4)	16.4 (12.3-22.3)	1671 (1302-2159)	5327 (3972-7176)	91.6 (71.4-118)	143 (106-192)
	CVD Deaths	13.2 (10.2-17.0)	9.6 (7.1-13.0)	2783 (2171-3601)	4829 (3602-6585)	153 (119-197)	129 (96.5-176)
Spain	T2D Incidence	7.9 (6.4-11.2)	10.1 (8.2-13.9)	7800 (6272-11095)	19978 (16096-27308)	278 (224-396)	529 (426-723)
	CVD Incidence	2.6 (2.2-3.9)	3.5 (2.8-5.2)	4812 (3970-7141)	6649 (5382-9961)	172 (142-255)	176 (142-264)
	T2D DALYs	4.4 (3.6-6.1)	5.9 (4.8-8.0)	14158 (11664-19989)	30503 (24540-41517)	505 (416-713)	807 (649-1099)
	CVD DALYs	2.5 (2.1-3.7)	2.8 (2.3-4.1)	38124 (31033-55537)	28282 (23254-40923)	1361 (1108-1982)	749 (615-1083)
	T2D Deaths	2.5 (2.1-3.5)	2.2 (1.8-2.9)	246 (200-340)	207 (172-275)	8.8 (7.1-12.1)	5.5 (4.5-7.3)
	CVD Deaths	1.7 (1.4-2.3)	1.7 (1.4-2.4)	1478 (1222-2019)	1137 (943-1557)	52.8 (43.6-72.1)	30.1 (25.0-41.2)
Sri Lanka	T2D Incidence	9.6 (6.9-13.5)	18.8 (14.1-24.5)	3367 (2408-4725)	28553 (21323-37249)	335 (239-470)	1945 (1453-2538)
	CVD Incidence	3.5 (2.4-5.4)	8.3 (5.6-12.0)	1292 (865-1998)	7213 (4929-10496)	128 (86.0-199)	491 (336-715)
	T2D DALYs	7.5 (5.3-10.9)	14.7 (10.4-20.4)	9266 (6510-13475)	71599 (51029-99650)	921 (647-1339)	4878 (3477-6790)
	CVD DALYs	4.0 (2.8-5.9)	8.7 (6.1-12.4)	17970 (12537-26429)	62784 (44384-89269)	1786 (1246-2627)	4278 (3024-6082)
	T2D Deaths	5.7 (3.8-8.9)	11.5 (7.7-17.0)	193 (127-300)	1260 (829-1853)	19.1 (12.6-29.8)	85.9 (56.5-126)
	CVD Deaths	2.9 (1.9-4.5)	6.5 (4.3-9.8)	567 (373-887)	2274 (1487-3425)	56.4 (37.1-88.2)	155 (101-233)
St. Lucia	T2D Incidence	27.2 (21.4-34.6)	29.6 (23.1-38.1)	110 (86.4-141)	380 (295-484)	1511 (1189-1937)	2764 (2148-3521)
	CVD Incidence	12.8 (9.9-17.4)	13.8 (10.2-18.6)	56.3 (42.6-76.3)	148 (111-202)	776 (587-1051)	1079 (811-1472)
	T2D DALYs	18.3 (14.1-24.0)	20.7 (15.7-26.6)	440 (340-580)	1086 (824-1401)	6059 (4684-7984)	7896 (5991-10190)

Supplementary Data 4. Proportional and absolute T2D and CVD burdens attributable to SSBs in 1990 and 2020 globally, regionally, and nationally (continued).

		Proportional burden % [§]		Absolute burden [§]		Absolute burden per 1M population [§, ƒ]	
Location	Outcome	1990	2020	1990	2020	1990	2020
	CVD DALYs	10.2 (7.8-13.8)	10.4 (7.7-13.9)	356 (270-481)	395 (292-528)	4901 (3713-6625)	2871 (2124-3844)
	T2D Deaths	13.8 (10.3-18.6)	14.3 (10.7-18.9)	10.7 (8.0-14.4)	17.9 (13.4-23.9)	147 (110-199)	131 (97.7-174)
	CVD Deaths	7.3 (5.5-10.1)	7.1 (5.2-9.9)	13.9 (10.4-19.1)	15.6 (11.5-21.6)	191 (143-263)	113 (83.6-157)
St. Vincent and the Grenadines	T2D Incidence	25.2 (19.8-31.8)	61.5 (53.4-68.9)	75.1 (58.9-95.5)	475 (414-535)	1378 (1081-1751)	6129 (5338-6901)
	CVD Incidence	12.0 (9.1-16.0)	35.0 (29.1-41.4)	39.3 (29.5-52.9)	213 (177-253)	720 (542-969)	2750 (2281-3265)
	T2D DALYs	17.1 (13.0-22.6)	48.4 (41.1-56.1)	329 (252-438)	1761 (1482-2040)	6039 (4616-8027)	22695 (19108-26303)
	CVD DALYs	11.0 (8.4-14.7)	30.3 (25.0-36.2)	349 (263-466)	1041 (860-1252)	6406 (4823-8536)	13419 (11093-16144)
	T2D Deaths	12.7 (9.6-17.7)	38.4 (31.9-45.8)	8.5 (6.3-11.7)	35.9 (29.8-43.0)	155 (116-215)	462 (384-554)
	CVD Deaths	8.0 (6.0-11.3)	23.0 (18.6-28.4)	13.0 (9.7-18.5)	43.3 (34.8-53.6)	239 (178-339)	558 (449-692)
Sudan	T2D Incidence	23.2 (15.0-33.7)	26.5 (17.1-38.3)	4701 (3008-6850)	27280 (17663-39653)	536 (343-781)	1263 (818-1836)
	CVD Incidence	10.4 (6.3-16.3)	11.7 (7.2-18.2)	10851 (6569-17085)	24292 (14752-38298)	1237 (749-1948)	1125 (683-1773)
	T2D DALYs	16.6 (10.5-24.7)	19.5 (12.4-28.7)	8118 (5135-12065)	38251 (24282-56476)	926 (586-1376)	1771 (1124-2615)
	CVD DALYs	11.2 (7.0-17.1)	12.0 (7.4-18.3)	99674 (60657-151809)	159568 (98264-244446)	11365 (6916-17309)	7388 (4550-11319)
	T2D Deaths	12.6 (7.6-19.7)	13.7 (8.4-21.2)	127 (76.9-198)	408 (248-634)	14.4 (8.8-22.5)	18.9 (11.5-29.3)
	CVD Deaths	8.6 (5.2-13.6)	8.8 (5.4-13.9)	2996 (1823-4835)	4774 (2891-7511)	342 (208-551)	221 (134-348)
Suriname	T2D Incidence	22.0 (14.9-31.6)	35.5 (25.0-47.9)	191 (129-276)	1232 (866-1665)	852 (577-1234)	3245 (2281-4387)
	CVD Incidence	10.1 (6.7-15.0)	16.4 (11.1-23.7)	124 (80.0-186)	479 (326-697)	554 (357-829)	1262 (859-1836)
	T2D DALYs	15.4 (10.3-22.7)	25.3 (17.5-35.7)	584 (391-865)	3460 (2416-4864)	2608 (1747-3862)	9115 (6364-12814)
	CVD DALYs	10.2 (6.7-15.1)	15.9 (10.8-22.8)	1220 (802-1808)	2948 (2009-4230)	5450 (3579-8072)	7767 (5294-11144)
	T2D Deaths	11.6 (7.7-17.5)	19.1 (13.0-28.0)	11.0 (7.2-16.6)	52.7 (35.8-76.2)	49.2 (32.2-74.2)	139 (94.2-201)
	CVD Deaths	7.5 (4.9-11.2)	11.8 (7.9-17.0)	40.1 (26.3-59.7)	101 (67.3-145)	179 (117-267)	266 (177-381)
Swaziland	T2D Incidence	24.3 (16.9-33.4)	30.3 (21.4-41.2)	181 (125-249)	850 (602-1157)	552 (382-760)	1425 (1010-1940)
	CVD Incidence	13.5 (8.9-20.1)	16.6 (11.1-24.0)	169 (111-251)	424 (281-600)	514 (340-765)	710 (472-1006)

Supplementary Data 4. Proportional and absolute T2D and CVD burdens attributable to SSBs in 1990 and 2020 globally, regionally, and nationally (continued).

		Proportional burden % §		Absolute burden §		Absolute burden per 1M population §, ƒ	
Location	Outcome	1990	2020	1990	2020	1990	2020
	T2D DALYs	19.0 (12.5-27.8)	23.9 (16.1-33.8)	1072 (708-1585)	4471 (3008-6450)	3267 (2156-4831)	7495 (5043-10813)
	CVD DALYs	12.1 (7.9-18.3)	16.3 (10.8-23.7)	971 (631-1467)	3181 (2109-4634)	2958 (1922-4471)	5333 (3535-7769)
	T2D Deaths	15.8 (10.0-23.7)	20.1 (13.0-29.5)	31.0 (20.0-46.7)	120 (77.3-177)	94.5 (60.8-142)	200 (130-296)
	CVD Deaths	9.3 (5.9-14.4)	12.7 (8.3-18.9)	31.3 (19.8-48.5)	98.8 (63.5-149)	95.5 (60.4-148)	166 (106-250)
Sweden	T2D Incidence	8.2 (6.9-11.0)	7.3 (6.1-10.3)	1235 (1037-1664)	2090 (1741-2938)	191 (160-257)	269 (224-378)
	CVD Incidence	2.3 (1.9-3.1)	1.8 (1.5-2.6)	1309 (1132-1786)	1018 (864-1456)	202 (175-276)	131 (111-187)
	T2D DALYs	4.5 (3.8-6.2)	3.8 (3.3-5.4)	2075 (1766-2879)	2965 (2503-4186)	321 (273-445)	381 (322-538)
	CVD DALYs	2.2 (1.9-3.0)	1.6 (1.3-2.2)	13906 (11984-19113)	4426 (3795-6192)	2151 (1853-2956)	569 (488-796)
	T2D Deaths	2.4 (2.0-3.2)	1.7 (1.4-2.2)	33.7 (29.3-46.1)	32.3 (28.0-43.6)	5.2 (4.5-7.1)	4.2 (3.6-5.6)
	CVD Deaths	1.6 (1.4-2.2)	1.1 (0.9-1.4)	620 (536-841)	205 (176-264)	95.9 (82.9-130)	26.3 (22.6-34.0)
Switzerland	T2D Incidence	9.9 (7.9-13.0)	10.5 (8.5-13.7)	1454 (1156-1910)	3751 (3051-4947)	286 (227-375)	541 (440-713)
	CVD Incidence	3.2 (2.5-4.5)	3.1 (2.5-4.4)	1085 (861-1523)	1101 (873-1582)	213 (169-299)	159 (126-228)
	T2D DALYs	5.3 (4.3-7.1)	6.3 (5.1-8.3)	2540 (2039-3395)	5231 (4184-6922)	499 (401-667)	754 (603-998)
	CVD DALYs	2.9 (2.3-4.1)	2.2 (1.8-3.1)	9980 (7935-13977)	4009 (3229-5525)	1962 (1560-2748)	578 (466-797)
	T2D Deaths	2.7 (2.2-3.6)	2.3 (1.9-3.1)	41.7 (34.1-56.3)	28.1 (22.8-37.2)	8.2 (6.7-11.1)	4.1 (3.3-5.4)
	CVD Deaths	2.0 (1.6-2.7)	1.4 (1.2-1.9)	409 (331-566)	190 (154-253)	80.5 (65.1-111)	27.4 (22.2-36.5)
Syrian Arab Republic	T2D Incidence	9.2 (6.4-12.7)	10.9 (7.7-15.3)	1286 (902-1788)	6561 (4652-9290)	249 (175-346)	623 (441-881)
	CVD Incidence	4.1 (2.9-6.7)	5.6 (3.9-8.9)	2430 (1711-3922)	8331 (5804-13187)	471 (331-760)	790 (551-1251)
	T2D DALYs	6.3 (4.5-9.1)	8.1 (5.8-11.7)	2401 (1709-3412)	11124 (7940-16163)	465 (331-661)	1055 (753-1534)
	CVD DALYs	5.1 (3.6-7.3)	5.7 (4.1-9.0)	25674 (17948-37408)	53799 (38216-83166)	4972 (3476-7245)	5104 (3626-7891)
	T2D Deaths	4.6 (3.3-6.9)	6.1 (4.3-9.3)	38.1 (27.4-58.1)	127 (89.2-195)	7.4 (5.3-11.3)	12.0 (8.5-18.5)
	CVD Deaths	3.6 (2.6-5.6)	4.6 (3.2-7.1)	741 (527-1120)	1864 (1305-2860)	144 (102-217)	177 (124-271)
Taiwan	T2D Incidence	17.5 (14.1-21.7)	12.7 (10.1-16.4)	5756 (4589-7141)	12445 (9868-15993)	439 (350-544)	636 (504-817)
	CVD Incidence	6.3 (5.0-8.1)	4.7 (3.8-6.2)	4389 (3494-5693)	7074 (5669-9355)	334 (266-434)	361 (290-478)
	T2D DALYs	9.4 (7.6-12.1)	7.4 (5.9-9.7)	15520 (12464-19776)	27290 (21756-35906)	1182 (950-1507)	1394 (1111-1834)
	CVD DALYs	5.5 (4.4-7.0)	4.5 (3.5-5.9)	18906 (15229-24383)	19262 (15244-25707)	1440 (1160-1858)	984 (779-1313)
	T2D Deaths	6.4 (5.1-8.7)	4.2 (3.4-5.9)	316 (253-431)	408 (325-566)	24.0 (19.3-32.8)	20.9 (16.6-28.9)
	CVD Deaths	3.8 (3.1-5.1)	3.0 (2.4-4.0)	585 (471-773)	605 (482-803)	44.6 (35.9-58.9)	30.9 (24.6-41.0)
Tajikistan	T2D Incidence	3.6 (2.2-6.5)	13.4 (9.0-19.4)	103 (62.7-186)	2123 (1423-3064)	42.1 (25.7-76.2)	411 (275-593)
	CVD Incidence	1.1 (0.7-1.9)	4.4 (3.1-6.9)	206 (137-353)	1930 (1338-3009)	84.6 (56.4-145)	373 (259-582)
	T2D DALYs	2.0 (1.3-3.7)	8.4 (5.6-12.8)	261 (170-473)	3673 (2521-5712)	107 (69.9-194)	710 (488-1105)
	CVD DALYs	1.2 (0.8-2.0)	4.6 (3.2-7.1)	2376 (1571-4022)	13038 (8942-19788)	974 (644-1649)	2521 (1729-3826)

Supplementary Data 4. Proportional and absolute T2D and CVD burdens attributable to SSBs in 1990 and 2020 globally, regionally, and nationally (continued).

		Proportional burden % §		Absolute burden §		Absolute burden per 1M population §, ƒ	
Location	Outcome	1990	2020	1990	2020	1990	2020
Tanzania	T2D Deaths	1.5 (1.0-2.8)	6.2 (4.1-10.1)	4.8 (3.1-9.0)	47.0 (30.8-77.4)	2.0 (1.3-3.7)	9.1 (5.9-15.0)
	CVD Deaths	0.8 (0.5-1.4)	3.3 (2.3-5.1)	77.3 (51.1-129)	421 (289-656)	31.7 (21.0-53.0)	81.5 (55.9-127)
	T2D Incidence	1.4 (0.9-2.8)	21.4 (14.9-29.0)	171 (103-343)	9962 (6911-13378)	15.5 (9.4-31.3)	365 (253-490)
	CVD Incidence	0.7 (0.4-1.4)	11.3 (7.4-16.3)	300 (181-573)	11746 (7607-17232)	27.3 (16.5-52.2)	431 (279-632)
	T2D DALYs	1.3 (0.8-2.6)	17.7 (11.7-24.8)	1250 (763-2586)	46653 (31082-65392)	114 (69.5-236)	1710 (1139-2397)
	CVD DALYs	0.7 (0.5-1.4)	11.3 (7.4-16.4)	1690 (1047-3362)	81568 (53142-120143)	154 (95.4-306)	2990 (1948-4404)
Thailand	T2D Deaths	1.0 (0.6-2.1)	13.4 (8.6-19.6)	33.3 (20.3-71.5)	1071 (697-1571)	3.0 (1.8-6.5)	39.3 (25.5-57.6)
	CVD Deaths	0.6 (0.3-1.1)	8.3 (5.3-12.8)	51.8 (31.8-107)	2514 (1582-3890)	4.7 (2.9-9.7)	92.2 (58.0-143)
	T2D Incidence	2.3 (1.0-5.1)	10.5 (5.1-20.0)	1597 (714-3499)	30254 (14617-57474)	47.9 (21.4-105)	562 (271-1067)
	CVD Incidence	0.8 (0.4-1.8)	4.3 (2.1-8.6)	824 (398-1823)	10265 (4957-20718)	24.8 (11.9-54.7)	191 (92.0-385)
	T2D DALYs	1.5 (0.7-3.4)	7.6 (3.7-14.8)	4221 (1902-9663)	76501 (36902-150146)	127 (57.1-290)	1420 (685-2787)
	CVD DALYs	1.0 (0.5-2.3)	4.9 (2.4-9.8)	8546 (4072-20015)	80720 (39437-159824)	257 (122-601)	1498 (732-2967)
Timor-Leste	T2D Deaths	1.1 (0.5-2.5)	5.5 (2.7-11.2)	81.7 (37.9-189)	1277 (611-2598)	2.5 (1.1-5.7)	23.7 (11.3-48.2)
	CVD Deaths	0.6 (0.3-1.5)	3.2 (1.5-6.6)	232 (113-540)	2422 (1160-5011)	7.0 (3.4-16.2)	45.0 (21.5-93.0)
	T2D Incidence	1.6 (1.0-3.3)	31.0 (24.6-38.9)	7.1 (4.3-14.8)	961 (768-1211)	18.8 (11.4-39.1)	1413 (1130-1781)
	CVD Incidence	0.6 (0.4-1.3)	13.5 (10.2-18.6)	4.4 (2.8-9.5)	340 (256-465)	11.8 (7.3-25.2)	501 (377-684)
	T2D DALYs	1.0 (0.6-2.3)	22.4 (17.2-29.5)	18.6 (11.5-40.4)	1924 (1477-2528)	49.1 (30.4-107)	2830 (2172-3719)
	CVD DALYs	0.8 (0.5-1.7)	16.1 (12.2-21.7)	78.0 (48.3-179)	5600 (4258-7570)	206 (128-474)	8238 (6264-11134)
Togo	T2D Deaths	0.7 (0.5-1.6)	17.2 (12.6-23.4)	0.3 (0.2-0.7)	29.4 (21.7-40.3)	0.9 (0.5-1.8)	43.3 (31.9-59.3)
	CVD Deaths	0.5 (0.3-1.1)	11.5 (8.6-15.8)	1.9 (1.2-4.2)	176 (131-245)	5.1 (3.2-11.3)	259 (193-360)
	T2D Incidence	7.0 (4.4-11.0)	66.4 (58.9-71.9)	126 (79.1-198)	6059 (5367-6579)	77.3 (48.5-122)	1506 (1334-1635)
	CVD Incidence	3.0 (1.8-5.2)	42.8 (34.3-50.1)	176 (107-306)	7288 (5827-8638)	108 (65.3-188)	1811 (1448-2147)
	T2D DALYs	5.4 (3.4-8.8)	59.2 (50.9-65.5)	474 (302-778)	24146 (20701-26968)	291 (185-477)	6001 (5145-6703)
	CVD DALYs	2.7 (1.6-4.6)	38.9 (31.5-46.3)	1158 (724-1986)	50639 (40212-60564)	710 (444-1217)	12586 (9994-15052)
	T2D Deaths	4.0 (2.5-6.9)	51.2 (42.0-58.3)	9.8 (6.1-17.0)	533 (438-616)	6.0 (3.7-10.4)	133 (109-153)
	CVD Deaths	2.0 (1.2-3.5)	33.1 (25.5-40.1)	36.3 (22.0-63.7)	1721 (1317-2100)	22.2 (13.5-39.0)	428 (327-522)

Supplementary Data 4. Proportional and absolute T2D and CVD burdens attributable to SSBs in 1990 and 2020 globally, regionally, and nationally (continued).

		Proportional burden % §		Absolute burden §		Absolute burden per 1M population §, ƒ	
Location	Outcome	1990	2020	1990	2020	1990	2020
Tonga	T2D Incidence	5.7 (3.5-9.9)	20.3 (13.6-30.0)	13.5 (8.2-23.2)	105 (70.4-156)	288 (174-494)	1828 (1224-2716)
	CVD Incidence	1.7 (1.1-3.1)	5.4 (3.4-8.9)	4.0 (2.5-7.1)	19.9 (12.6-32.6)	85.2 (53.5-152)	345 (219-566)
	T2D DALYs	3.0 (1.9-5.5)	10.3 (6.6-16.0)	46.0 (28.9-84.0)	300 (192-465)	982 (617-1791)	5217 (3329-8079)
	CVD DALYs	2.1 (1.3-4.1)	7.1 (4.5-11.7)	43.5 (27.2-82.4)	207 (132-338)	928 (581-1758)	3605 (2289-5871)
	T2D Deaths	2.1 (1.4-3.9)	6.7 (4.3-10.6)	1.0 (0.6-1.8)	5.7 (3.7-9.2)	21.3 (13.2-37.8)	99.7 (64.0-160)
	CVD Deaths	1.5 (1.0-3.0)	4.8 (3.0-7.7)	1.2 (0.8-2.5)	6.1 (3.9-10.0)	26.5 (16.7-52.6)	107 (68.4-174)
Trinidad and Tobago	T2D Incidence	27.9 (19.6-38.3)	34.4 (24.8-46.0)	1267 (886-1737)	3834 (2760-5092)	1823 (1274-2499)	3725 (2681-4947)
	CVD Incidence	13.6 (9.2-19.6)	16.7 (11.5-23.6)	621 (420-904)	1509 (1031-2146)	893 (604-1300)	1467 (1002-2085)
	T2D DALYs	19.3 (13.4-27.9)	23.5 (16.3-32.8)	6167 (4253-8940)	14956 (10410-20850)	8870 (6118-12859)	14531 (10114-20258)
	CVD DALYs	13.1 (9.0-19.1)	15.7 (10.9-22.4)	6354 (4357-9231)	8854 (6083-12476)	9139 (6267-13277)	8602 (5910-12122)
	T2D Deaths	15.6 (10.6-22.9)	18.0 (12.2-26.0)	157 (107-230)	326 (222-470)	226 (154-331)	316 (216-456)
	CVD Deaths	9.8 (6.6-14.6)	11.6 (8.0-16.6)	219 (149-326)	318 (216-455)	315 (214-469)	309 (210-442)
Tunisia	T2D Incidence	13.3 (9.4-19.0)	21.8 (15.8-30.1)	1463 (1047-2090)	13363 (9807-18657)	338 (242-483)	1637 (1202-2286)
	CVD Incidence	6.7 (4.5-10.4)	11.2 (7.8-16.9)	3029 (2100-4740)	13306 (9247-20207)	700 (485-1095)	1630 (1133-2476)
	T2D DALYs	9.5 (6.7-14.0)	16.2 (11.7-23.2)	2365 (1672-3487)	22844 (16423-32657)	547 (386-806)	2799 (2012-4002)
	CVD DALYs	6.4 (4.5-9.6)	10.1 (7.0-15.1)	16388 (11295-24385)	50499 (35357-74720)	3787 (2610-5635)	6188 (4332-9156)
	T2D Deaths	7.1 (4.9-10.7)	11.9 (8.3-17.5)	31.2 (21.5-47.4)	236 (164-358)	7.2 (5.0-11.0)	28.9 (20.1-43.8)
	CVD Deaths	5.1 (3.5-7.8)	7.8 (5.4-11.8)	581 (396-888)	1994 (1355-3057)	134 (91.4-205)	244 (166-375)
Turkey	T2D Incidence	9.6 (7.1-13.1)	1.0 (0.5-3.0)	6287 (4584-8609)	3222 (1412-9544)	217 (158-297)	56.2 (24.6-166)
	CVD Incidence	5.4 (3.9-7.9)	0.5 (0.2-1.5)	17600 (12720-25833)	3479 (1618-9963)	607 (438-890)	60.7 (28.2-174)
	T2D DALYs	7.0 (5.1-9.8)	0.7 (0.3-2.0)	22222 (16219-31615)	6728 (3080-18669)	766 (559-1090)	117 (53.7-326)
	CVD DALYs	5.2 (3.8-7.4)	0.4 (0.2-1.3)	99088 (72434-141305)	12508 (5808-35634)	3416 (2497-4871)	218 (101-622)
	T2D Deaths	5.4 (3.9-7.8)	0.5 (0.2-1.4)	577 (413-825)	112 (52.7-311)	19.9 (14.2-28.4)	2.0 (0.9-5.4)
	CVD Deaths	4.0 (2.9-5.6)	0.3 (0.2-0.9)	3299 (2402-4669)	511 (237-1352)	114 (82.8-161)	8.9 (4.1-23.6)
Turkmenistan	T2D Incidence	44.7 (35.8-54.1)	2.6 (1.6-4.8)	867 (697-1049)	216 (132-398)	478 (384-578)	58.5 (35.7-108)
	CVD Incidence	18.7 (14.0-24.9)	0.8 (0.5-1.4)	2725 (2039-3623)	252 (163-436)	1501 (1123-1997)	68.3 (44.1-118)
	T2D DALYs	30.5 (23.6-40.1)	1.5 (0.9-2.7)	2324 (1794-3050)	571 (351-1034)	1280 (989-1681)	155 (95.2-280)

Supplementary Data 4. Proportional and absolute T2D and CVD burdens attributable to SSBs in 1990 and 2020 globally, regionally, and nationally (continued).

		Proportional burden % §		Absolute burden §		Absolute burden per 1M population §, ƒ	
Location	Outcome	1990	2020	1990	2020	1990	2020
	CVD DALYs	20.4 (15.6-27.3)	0.8 (0.5-1.4)	34992 (26891-46847)	2593 (1672-4471)	19282 (14817-25813)	703 (453-1212)
	T2D Deaths	25.1 (18.6-34.1)	1.2 (0.7-2.2)	45.1 (33.4-61.4)	9.6 (6.1-18.2)	24.8 (18.4-33.8)	2.6 (1.6-4.9)
	CVD Deaths	15.7 (11.8-21.4)	0.6 (0.4-1.0)	1182 (885-1617)	85.0 (55.2-147)	651 (488-891)	23.1 (15.0-39.7)
Uganda	T2D Incidence	0.5 (0.3-1.4)	30.5 (22.2-39.2)	45.1 (27.6-120)	9515 (6886-12288)	6.3 (3.8-16.6)	490 (354-632)
	CVD Incidence	0.3 (0.2-0.5)	15.5 (10.5-22.0)	71.7 (42.9-136)	8419 (5668-12082)	10.0 (5.9-18.9)	433 (292-622)
	T2D DALYs	0.4 (0.3-0.9)	25.0 (17.6-33.7)	287 (174-575)	44804 (31585-60946)	39.8 (24.1-79.8)	2305 (1625-3136)
	CVD DALYs	0.3 (0.2-0.5)	15.6 (10.4-22.3)	377 (225-665)	47280 (31740-67874)	52.3 (31.2-92.3)	2433 (1633-3492)
	T2D Deaths	0.3 (0.2-0.7)	19.0 (12.7-26.8)	7.9 (4.6-14.6)	1043 (700-1502)	1.1 (0.6-2.0)	53.7 (36.0-77.3)
	CVD Deaths	0.2 (0.1-0.4)	11.7 (7.5-17.2)	12.0 (6.9-22.0)	1404 (921-2101)	1.7 (1.0-3.1)	72.2 (47.4-108)
Ukraine	T2D Incidence	7.1 (5.0-10.1)	4.9 (3.5-7.3)	4144 (2971-5965)	4712 (3405-7212)	112 (80.5-162)	136 (98.0-207)
	CVD Incidence	2.2 (1.6-3.1)	1.4 (1.1-2.0)	13074 (9605-18869)	10403 (7537-14817)	354 (260-511)	299 (217-426)
	T2D DALYs	4.5 (3.3-6.7)	2.9 (2.1-4.4)	7649 (5652-11356)	7482 (5426-11270)	207 (153-308)	215 (156-324)
	CVD DALYs	2.1 (1.6-3.0)	1.4 (1.0-2.0)	122690 (90294-175000)	87329 (63517-125659)	3324 (2446-4741)	2512 (1827-3615)
	T2D Deaths	3.5 (2.6-5.3)	2.2 (1.6-3.2)	85.2 (63.2-130)	48.7 (35.7-71.7)	2.3 (1.7-3.5)	1.4 (1.0-2.1)
	CVD Deaths	1.6 (1.2-2.3)	1.0 (0.8-1.5)	4962 (3613-7086)	3730 (2712-5319)	134 (97.9-192)	107 (78.0-153)
United Arab Emirates	T2D Incidence	17.3 (11.5-26.4)	7.3 (4.4-14.0)	518 (344-787)	4334 (2543-8440)	460 (306-700)	539 (316-1050)
	CVD Incidence	10.3 (6.8-16.6)	4.6 (2.8-9.4)	664 (431-1074)	2934 (1789-6057)	591 (383-956)	365 (223-754)
	T2D DALYs	13.1 (8.7-19.8)	6.3 (3.8-11.9)	796 (519-1195)	4451 (2686-8317)	708 (461-1063)	554 (334-1035)
	CVD DALYs	9.8 (6.5-15.6)	4.3 (2.6-8.1)	3345 (2193-5246)	4768 (2890-8996)	2975 (1951-4667)	593 (360-1119)
	T2D Deaths	10.1 (6.6-15.8)	4.7 (2.9-9.0)	13.6 (8.9-21.3)	34.3 (21.1-64.2)	12.1 (7.9-19.0)	4.3 (2.6-8.0)
	CVD Deaths	7.9 (5.1-12.4)	3.7 (2.3-7.0)	89.3 (57.7-141)	118 (72.1-222)	79.5 (51.3-125)	14.7 (9.0-27.6)
United Kingdom	T2D Incidence	15.7 (13.5-19.5)	16.2 (13.9-20.3)	15011 (12815-18650)	35514 (30378-44443)	354 (302-440)	680 (582-851)
	CVD Incidence	4.7 (4.0-6.3)	4.5 (3.9-6.3)	16467 (14085-22346)	11190 (9624-15919)	388 (332-527)	214 (184-305)
	T2D DALYs	8.7 (7.6-11.2)	10.2 (8.8-13.1)	24835 (21582-31902)	53341 (45829-68834)	585 (509-752)	1022 (878-1319)
	CVD DALYs	4.8 (4.2-6.6)	4.1 (3.5-5.8)	210319 (182613-287409)	67233 (58282-95099)	4957 (4304-6774)	1288 (1117-1822)
	T2D Deaths	4.8 (4.2-6.5)	3.7 (3.2-4.8)	397 (349-538)	227 (200-295)	9.4 (8.2-12.7)	4.4 (3.8-5.6)
	CVD Deaths	3.5 (3.0-4.8)	2.7 (2.3-3.6)	8530 (7405-11548)	2704 (2344-3613)	201 (175-272)	51.8 (44.9-69.2)
United States	T2D Incidence	19.0 (16.6-24.5)	15.7 (13.7-20.8)	94388 (82001-123108)	298151 (259888-401299)	526 (457-686)	1198 (1044-1612)

Supplementary Data 4. Proportional and absolute T2D and CVD burdens attributable to SSBs in 1990 and 2020 globally, regionally, and nationally (continued).

		Proportional burden % §		Absolute burden §		Absolute burden per 1M population §, ƒ	
Location	Outcome	1990	2020	1990	2020	1990	2020
	CVD Incidence	6.8 (6.0-9.2)	5.4 (4.8-7.4)	116705 (102117-158932)	66911 (59461-92061)	650 (569-885)	269 (239-370)
	T2D DALYs	10.5 (9.3-14.0)	9.8 (8.8-13.8)	177124 (159142-238967)	455890 (404162-635601)	987 (886-1331)	1831 (1623-2553)
	CVD DALYs	6.4 (5.7-8.8)	5.8 (5.1-8.4)	806389 (717414-1106898)	611902 (542285-882916)	4492 (3996-6166)	2458 (2178-3547)
	T2D Deaths	7.1 (6.4-9.5)	6.7 (6.0-9.9)	3328 (3011-4425)	4615 (4160-6851)	18.5 (16.8-24.6)	18.5 (16.7-27.5)
	CVD Deaths	4.4 (3.9-6.0)	3.8 (3.4-5.3)	30550 (27362-41370)	22499 (20115-31526)	170 (152-230)	90.4 (80.8-127)
Uruguay	T2D Incidence	26.1 (18.4-37.3)	31.3 (22.4-43.1)	1146 (802-1649)	3667 (2616-5001)	562 (393-808)	1454 (1038-1983)
	CVD Incidence	10.5 (7.1-16.1)	12.4 (8.5-18.3)	1553 (1050-2365)	1845 (1266-2769)	761 (514-1159)	732 (502-1098)
	T2D DALYs	17.1 (11.7-25.2)	20.1 (14.0-28.6)	3279 (2239-4818)	7215 (4972-10269)	1607 (1097-2361)	2861 (1972-4072)
	CVD DALYs	10.8 (7.3-16.2)	11.5 (7.9-17.0)	17414 (11880-26070)	11026 (7677-16210)	8533 (5821-12774)	4372 (3044-6428)
	T2D Deaths	13.3 (8.9-20.2)	14.0 (9.4-20.7)	87.0 (57.8-132)	124 (83.9-185)	42.6 (28.3-64.9)	49.3 (33.3-73.4)
	CVD Deaths	8.1 (5.5-12.7)	8.1 (5.5-12.2)	709 (478-1103)	468 (319-702)	347 (234-540)	186 (127-278)
Uzbekistan	T2D Incidence	16.9 (11.8-24.3)	10.0 (6.5-15.7)	2280 (1599-3280)	9193 (6062-14509)	227 (159-327)	432 (285-681)
	CVD Incidence	5.4 (3.8-8.4)	3.1 (2.1-5.1)	4306 (2963-6691)	9084 (6053-14911)	429 (295-667)	427 (284-700)
	T2D DALYs	10.2 (6.9-15.4)	6.2 (4.0-10.4)	4964 (3376-7507)	18771 (12259-31660)	495 (337-748)	881 (576-1487)
	CVD DALYs	6.2 (4.3-9.3)	3.9 (2.6-6.2)	51801 (35914-77842)	66965 (44086-106239)	5165 (3581-7761)	3145 (2070-4989)
	T2D Deaths	7.9 (5.3-12.6)	4.7 (3.1-8.3)	81.6 (54.8-132)	289 (186-501)	8.1 (5.5-13.1)	13.6 (8.7-23.5)
	CVD Deaths	4.2 (2.9-6.7)	2.8 (1.8-4.7)	1674 (1155-2629)	2157 (1421-3649)	167 (115-262)	101 (66.7-171)
Vanuatu	T2D Incidence	5.1 (3.0-8.7)	8.5 (5.0-13.4)	11.3 (6.8-19.5)	96.5 (56.8-152)	168 (101-288)	606 (357-952)
	CVD Incidence	1.5 (0.9-2.8)	2.3 (1.4-3.8)	4.5 (2.8-8.2)	20.1 (12.4-33.0)	67.2 (41.9-122)	126 (78.0-207)
	T2D DALYs	2.7 (1.6-4.7)	4.4 (2.7-7.2)	38.0 (22.6-68.4)	247 (150-402)	562 (334-1012)	1553 (941-2523)
	CVD DALYs	2.1 (1.3-4.2)	3.4 (2.1-6.1)	119 (72.5-239)	498 (299-886)	1766 (1073-3538)	3127 (1879-5559)
	T2D Deaths	1.9 (1.2-3.5)	3.0 (1.8-4.9)	0.8 (0.5-1.3)	4.1 (2.5-6.8)	11.1 (6.9-19.7)	25.5 (15.7-42.6)
	CVD Deaths	1.6 (1.0-3.0)	2.5 (1.5-4.3)	3.0 (1.8-5.7)	12.4 (7.6-21.9)	44.2 (27.1-85.0)	78.0 (48.0-137)
Venezuela	T2D Incidence	30.3 (23.6-38.6)	17.7 (12.9-24.5)	7688 (5926-9819)	17167 (12598-23655)	757 (583-966)	944 (693-1301)
	CVD Incidence	14.6 (10.9-19.9)	8.1 (5.8-12.0)	6133 (4548-8379)	8829 (6260-13197)	603 (448-824)	486 (344-726)
	T2D DALYs	21.4 (16.4-28.1)	12.0 (8.8-17.4)	24400 (18830-32115)	54565 (39705-78798)	2401 (1853-3160)	3001 (2184-4334)
	CVD DALYs	15.3 (11.6-20.8)	8.2 (5.9-12.3)	61153 (45883-83310)	77431 (55886-117383)	6017 (4515-8197)	4258 (3074-6456)
	T2D Deaths	16.3 (12.0-22.5)	8.7 (6.2-13.1)	497 (366-684)	1041 (738-1554)	48.9 (36.0-67.3)	57.3 (40.6-85.5)
	CVD Deaths	11.3 (8.4-15.9)	5.9 (4.2-9.0)	1948 (1443-2749)	2704 (1936-4101)	192 (142-270)	149 (106-226)
Vietnam	T2D Incidence	3.0 (2.1-4.8)	4.0 (3.0-6.2)	1955 (1382-3158)	11213 (8226-17281)	55.2 (39.1-89.2)	164 (121-253)

Supplementary Data 4. Proportional and absolute T2D and CVD burdens attributable to SSBs in 1990 and 2020 globally, regionally, and nationally (continued).

		Proportional burden % §		Absolute burden §		Absolute burden per 1M population §, ƒ	
Location	Outcome	1990	2020	1990	2020	1990	2020
	CVD Incidence	1.1 (0.8-1.8)	1.7 (1.3-2.8)	1330 (968-2148)	5347 (4029-8854)	37.6 (27.4-60.7)	78.3 (59.0-130)
	T2D DALYs	1.8 (1.3-3.1)	2.8 (2.1-4.5)	5931 (4300-10171)	27755 (20831-45242)	168 (121-287)	407 (305-663)
	CVD DALYs	1.0 (0.7-1.7)	1.5 (1.1-2.4)	11103 (8043-18121)	41579 (31325-66470)	314 (227-512)	609 (459-974)
	T2D Deaths	1.2 (0.9-2.2)	1.9 (1.4-3.3)	137 (101-242)	556 (422-967)	3.9 (2.9-6.8)	8.1 (6.2-14.2)
	CVD Deaths	0.7 (0.5-1.0)	1.0 (0.8-1.5)	347 (258-538)	1362 (1030-2108)	9.8 (7.3-15.2)	20.0 (15.1-30.9)
Yemen, Rep.	T2D Incidence	32.1 (18.9-49.4)	41.7 (26.0-60.4)	2997 (1775-4600)	23905 (14745-34158)	676 (400-1037)	1589 (980-2271)
	CVD Incidence	17.2 (9.5-29.3)	22.3 (12.9-36.3)	9091 (4999-15602)	31844 (18250-52141)	2050 (1127-3518)	2117 (1213-3466)
	T2D DALYs	23.4 (13.6-38.1)	31.5 (19.2-48.6)	5704 (3264-9250)	35696 (21688-55091)	1286 (736-2086)	2373 (1442-3662)
	CVD DALYs	17.3 (9.6-29.5)	21.6 (12.7-34.9)	77488 (42727-131764)	213799 (124888-350366)	17474 (9635-29714)	14213 (8302-23292)
	T2D Deaths	18.9 (10.8-32.3)	23.8 (13.7-38.8)	95.6 (52.3-165)	400 (231-664)	21.6 (11.8-37.2)	26.6 (15.4-44.1)
	CVD Deaths	14.5 (8.0-24.8)	17.5 (10.0-28.6)	2462 (1384-4295)	7020 (3981-11616)	555 (312-969)	467 (265-772)
Zambia	T2D Incidence	38.4 (29.2-47.6)	17.7 (12.1-24.1)	2155 (1640-2680)	4009 (2741-5462)	645 (491-802)	491 (336-669)
	CVD Incidence	21.3 (15.2-28.8)	8.1 (5.3-11.8)	2469 (1736-3353)	2251 (1484-3288)	738 (519-1003)	276 (182-402)
	T2D DALYs	34.7 (25.7-44.3)	14.2 (9.5-19.4)	13119 (9829-16872)	15466 (10263-21064)	3923 (2940-5046)	1893 (1256-2578)
	CVD DALYs	19.9 (13.9-26.9)	7.8 (5.0-11.5)	11999 (8342-16369)	15353 (9956-22719)	3589 (2495-4896)	1879 (1219-2781)
	T2D Deaths	28.6 (20.3-38.0)	10.6 (6.8-15.3)	332 (234-449)	286 (185-418)	99.4 (69.9-134)	34.9 (22.6-51.1)
	CVD Deaths	15.5 (10.5-22.1)	5.8 (3.6-8.8)	378 (255-538)	463 (290-696)	113 (76.2-161)	56.7 (35.4-85.2)
Zimbabwe	T2D Incidence	11.9 (8.1-17.2)	28.8 (20.7-38.5)	786 (531-1139)	6198 (4417-8319)	176 (119-255)	886 (631-1189)
	CVD Incidence	6.3 (4.0-9.7)	15.3 (10.5-21.9)	1096 (708-1691)	4919 (3329-7029)	245 (158-378)	703 (476-1005)
	T2D DALYs	8.9 (5.7-13.7)	22.0 (14.9-31.0)	3419 (2219-5174)	28533 (19232-40818)	765 (496-1158)	4079 (2749-5835)
	CVD DALYs	5.0 (3.2-7.8)	14.1 (9.7-20.3)	4274 (2692-6690)	32142 (22107-47312)	956 (602-1497)	4595 (3161-6764)
	T2D Deaths	7.0 (4.3-11.4)	18.2 (12.1-26.6)	89.6 (54.8-145)	712 (474-1048)	20.0 (12.3-32.4)	102 (67.8-150)
	CVD Deaths	3.9 (2.4-6.3)	11.0 (7.3-16.5)	148 (91.5-243)	1040 (680-1555)	33.0 (20.5-54.3)	149 (97.2-222)

§ Data represent the central estimate (median) and 95% UI derived from the 2.5th and 97.5th percentiles of 1,000 multiway probabilistic Monte Carlo model simulations. The values shown are the direct and BMI mediated burdens combined using proportional multiplication.

Supplementary Data 4. Proportional and absolute T2D and CVD burdens attributable to SSBs in 1990 and 2020 globally, regionally, and nationally (continued).

		Proportional burden % §		Absolute burden §		Absolute burden per 1M population §, ƒ	
Location	Outcome	1990	2020	1990	2020	1990	2020

ƒ The absolute burden per 1 million adults was calculated by dividing the absolute number of cases by the global/region/country adult population (20+ year) in that year and multiplying by 1 million.

† In prior GDD reports, the region Central/ Eastern Europe and Central Asia was referred as Former Soviet Union, and Southeast and East Asia was referred as Asia.

CVD, cardiovascular disease; DALYs, disability-adjusted life years; T2D, type 2 diabetes; UIs, uncertainty intervals.
