# Supplementary material for: Burdens of type 2 diabetes and cardiovascular disease attributable to sugar-sweetened beverages in 184 countries
Source: Nat Med. 2025 Jan 6;31(2):552–64. doi: 10.1038/s41591-024-03345-4 (PMC11835746; doi:10.1038/s41591-024-03345-4)
Supplement: Supplementary file 4 — Differences in proportional and absolute CVD and T2D burdens attributable to SSBs from 1990 to 2020 globally, regionally and nationally. [file 41591_2024_3345_MOESM4_ESM.pdf]

# Burdens of type 2 diabetes and cardiovascular disease burdens to sugar-sweetened beverages in 184 countries

Supplementary Data 2 | Differences in proportional and absolute CVD and T2D burdens attributable to SSBs from 1990 to 2020 globally, regionally, and nationally.

Supplementary Data 2. Differences in proportional and absolute T2D and CVD burdens attributable to SSBs from 1990 to 2020 globally, regionally, and nationally.

| | | Difference in proportional burden (1990-2020) % § | | | Difference in absolute burden per 1M adults (1990-2020) §, ƒ | | |
|---|---|---|---|---|---|---|---|
| Location | Disease | Incidence | DALYs | Deaths | Incidence | DALYs | Deaths |
| World | T2D | 1.3 (0.9,1.7) | 0.6 (0.3,0.8) | 0.2 (0.0,0.5) | 221 (200,242) | 452 (403,502) | 5.4 (4.6,6.5) |
| | CVD | -0.1 (-0.3,0.0) | -0.2 (-0.3,0.0) | -0.3 (-0.4,-0.2) | 21.7 (8.7,33.8) | -251 (-327,-177) | -9.4 (-12.2,-7.0) |
| World region | | | | | | | |
| Centr/Eastern Europe and Centr Asia | T2D | 1.5 (1.0,2.3) | 0.5 (0.2,1.0) | 0.0 (-0.2,0.4) | 154 (127,194) | 303 (249,403) | 4.4 (3.4,6.1) |
| | CVD | 0.5 (0.3,0.8) | 0.3 (0.1,0.5) | 0.2 (0.1,0.3) | 103 (76.8,155) | -131 (-291,126) | 3.0 (-2.7,11.9) |
| High-Income Countries | T2D | -0.2 (-0.7,1.0) | 0.6 (0.4,1.4) | -0.2 (-0.4,0.2) | 385 (344,508) | 476 (420,648) | -1.0 (-1.5,0.2) |
| | CVD | -0.9 (-1.3,-0.7) | -0.3 (-0.5,0.0) | -0.4 (-0.6,-0.3) | -187 (-240,-164) | -1684 (-1934,-1548) | -65.6 (-75.9,-60.4) |
| Latin Amer/Caribbean | T2D | -3.2 (-4.1,-2.2) | -2.6 (-3.3,-1.9) | -2.5 (-3.1,-1.9) | 401 (339,473) | 836 (670,1031) | 8.0 (5.1,11.7) |
| | CVD | -1.1 (-1.6,-0.6) | -1.6 (-2.2,-1.0) | -1.2 (-1.7,-0.8) | 4.4 (-21.9,35.0) | -1438 (-1715,-1227) | -41.6 (-50.7,-33.9) |
| Mid. East/North Africa | T2D | 0.9 (0.1,1.8) | 0.5 (-0.3,1.2) | -0.7 (-1.4,-0.1) | 649 (540,764) | 834 (689,1011) | 4.4 (2.6,6.5) |
| | CVD | 0.2 (-0.3,0.7) | -0.1 (-0.5,0.5) | -0.3 (-0.6,0.1) | 84.9 (24.2,152) | -1037 (-1388,-710) | -29.8 (-40.7,-19.3) |
| South Asia | T2D | 1.1 (0.8,1.7) | 0.5 (0.3,0.7) | 0.1 (-0.1,0.2) | 94.1 (66.1,134) | 191 (131,275) | 2.5 (1.6,3.9) |
| | CVD | 0.2 (0.1,0.4) | 0.3 (0.2,0.6) | 0.2 (0.1,0.3) | 29.1 (18.1,50.6) | 246 (155,397) | 7.3 (4.4,11.7) |
| Southeast and East Asia | T2D | 0.4 (-0.2,0.9) | 0.4 (0.1,0.8) | 0.4 (0.2,0.8) | 54.3 (40.7,73.4) | 140 (112,190) | 2.0 (1.5,2.9) |
| | CVD | -0.1 (-0.2,0.0) | 0.0 (-0.1,0.1) | -0.1 (-0.2,0.0) | 17.6 (13.5,24.8) | 119 (77.9,179) | 4.1 (2.9,6.0) |
| Sub-Saharan Africa | T2D | 8.8 (6.8,11.0) | 7.1 (5.4,9.2) | 5.5 (4.0,7.3) | 373 (300,458) | 1058 (810,1344) | 19.4 (14.3,25.9) |
| | CVD | 4.4 (3.1,5.8) | 3.6 (2.5,5.0) | 2.8 (1.8,4.1) | 148 (103,200) | 635 (354,935) | 22.4 (12.6,34.1) |
| Country | | | | | | | |
| Afghanistan | T2D | 8.3 (4.1,15.8) | 6.0 (2.9,12.0) | 4.1 (1.9,8.9) | 529 (266,1049) | 743 (358,1516) | 6.9 (3.2,15.8) |
| | CVD | 3.1 (1.4,6.7) | 3.1 (1.5,6.8) | 2.3 (1.1,5.0) | 156 (71.8,357) | 1212 (563,2871) | 32.3 (14.6,72.6) |
| Albania | T2D | 47.3 (38.7,55.1) | 36.3 (27.9,44.5) | 26.0 (18.9,34.6) | 1477 (1214,1732) | 2812 (2166,3484) | 24.6 (17.8,33.0) |
| | CVD | 24.4 (18.2,31.6) | 21.4 (15.7,27.8) | 16.9 (11.9,23.0) | 1870 (1386,2466) | 13601 (9914,17779) | 650 (463,887) |
| Algeria | T2D | 0.4 (-0.9,2.2) | 0.3 (-0.7,1.6) | -0.8 (-2.1,0.1) | 871 (575,1280) | 1286 (839,1912) | 8.4 (5.4,13.3) |
| | CVD | 0.0 (-1.1,1.1) | -1.4 (-2.6,-0.7) | -1.5 (-2.8,-0.9) | 18.8 (-138,182) | -1995 (-3172,-1263) | -50.7 (-89.9,-29.8) |
| Angola | T2D | 12.6 (8.0,17.9) | 9.7 (5.8,13.9) | 6.9 (3.8,11.0) | 673 (471,925) | 1704 (1085,2439) | 22.2 (12.0,36.2) |
| | CVD | 5.9 (3.3,9.3) | 5.3 (2.7,8.8) | 4.1 (2.0,7.2) | 193 (98.5,322) | 1288 (594,2288) | 41.6 (18.5,75.4) |
| Antigua and Barbuda | T2D | 3.5 (1.7,5.7) | 4.4 (2.8,6.4) | 2.7 (1.6,4.2) | 1305 (912,1835) | 2045 (1369,2925) | -5.5 (-15.9,2.2) |
| | CVD | 3.7 (2.3,5.9) | 1.5 (0.8,2.6) | 1.2 (0.7,2.0) | 237 (128,403) | -2210 (-3301,-1436) | -92.9 (-141,-59.1) |
| Argentina | T2D | 6.6 (5.0,8.4) | 4.1 (3.2,5.7) | 2.5 (1.9,3.9) | 544 (432,682) | 736 (583,1026) | 5.6 (3.4,9.4) |
| | CVD | 2.8 (2.1,4.0) | 1.9 (1.5,2.9) | 1.3 (1.0,2.0) | 49.5 (0.8,106) | -1179 (-1824,-896) | -39.1 (-63.4,-29.8) |
| Armenia | T2D | 3.5 (2.2,6.1) | 2.1 (1.3,3.6) | 1.4 (0.9,2.4) | 175 (113,297) | 340 (218,606) | 4.5 (2.9,8.1) |
| | CVD | 1.2 (0.8,2.1) | 1.0 (0.7,1.9) | 0.7 (0.5,1.3) | 181 (116,334) | 1070 (686,1910) | 45.7 (29.5,80.9) |
| Australia | T2D | -3.8 (-5.3,-2.6) | -2.3 (-3.5,-1.6) | -2.0 (-3.6,-1.6) | 85.1 (57.8,167) | 36.4 (-4.5,131) | -2.7 (-5.1,-2.0) |
| | CVD | -1.8 (-2.8,-1.3) | -2.0 (-3.4,-1.5) | -1.7 (-2.9,-1.3) | -171 (-259,-128) | -2497 (-3758,-1937) | -96.8 (-150,-74.0) |
| Austria | T2D | 0.5 (0.0,1.3) | 0.0 (-0.5,0.4) | -0.7 (-1.3,-0.5) | 99.8 (79.5,138) | 69.5 (39.1,119) | -2.3 (-4.2,-1.7) |
| | CVD | -0.1 (-0.4,0.1) | -0.6 (-1.0,-0.5) | -0.5 (-0.7,-0.4) | -3.3 (-19.1,16.1) | -1411 (-1924,-1165) | -54.2 (-74.4,-45.3) |
| Azerbaijan | T2D | 1.6 (0.3,3.5) | 1.0 (0.4,2.4) | 0.7 (0.1,1.9) | 138 (89.7,235) | 251 (162,454) | 3.2 (2.0,6.6) |
| | CVD | 0.5 (0.2,1.2) | 0.4 (-0.1,1.2) | 0.3 (0.0,0.9) | 91.1 (52.2,183) | 165 (-309,756) | 10.8 (-1.1,31.4) |
| Bahamas, The | T2D | -35.0 (-40.0,-27.7) | -27.1 (-33.7,-20.0) | -22.9 (-30.3,-16.1) | -565 (-759,-237) | -3602 (-4461,-2605) | -109 (-145,-76.5) |

Supplementary Data 2. Differences in proportional and absolute T2D and CVD burdens attributable to SSBs from 1990 to 2020 globally, regionally, and nationally (continued).

| | | Difference in proportional burden (1990-2020) % § | | | Difference in absolute burden per 1M adults (1990-2020) §, f | | |
|---|---|---|---|---|---|---|---|
| Location | Disease | Incidence | DALYs | Deaths | Incidence | DALYs | Deaths |
| | CVD | -20.5 (-26.7,-14.9) | -21.8 (-28.4,-15.7) | -17.2 (-23.3,-12.0) | -814 (-1083,-582) | -9302 (-12175,-6589) | -302 (-411,-208) |
| Bahrain | T2D | 2.7 (-1.3,8.0) | 3.0 (-0.1,6.9) | 1.7 (-0.8,4.8) | 1137 (724,1791) | 1511 (841,2466) | 12.5 (0.3,27.8) |
| | CVD | 1.9 (-0.9,5.9) | 1.7 (-0.7,4.7) | 1.1 (-0.7,3.3) | 206 (-8.2,549) | -2446 (-3983,-1460) | -74.5 (-125,-43.8) |
| Bangladesh | T2D | 0.2 (0.1,0.6) | 0.1 (0.0,0.3) | 0.0 (0.0,0.1) | 26.8 (16.1,59.4) | 49.1 (29.4,101) | 0.5 (0.3,1.1) |
| | CVD | 0.1 (0.1,0.2) | 0.1 (0.0,0.2) | 0.0 (0.0,0.1) | 10.6 (5.8,23.3) | 30.0 (6.3,79.6) | 1.4 (0.6,3.2) |
| Barbados | T2D | 8.8 (6.7,11.0) | 7.2 (5.7,9.1) | 4.2 (3.0,5.5) | 2129 (1737,2596) | 5814 (4532,7164) | 71.5 (51.8,94.6) |
| | CVD | 5.4 (3.9,7.3) | 3.4 (2.5,4.6) | 2.6 (1.9,3.6) | 935 (691,1240) | -360 (-751,40.3) | -19.7 (-40.6,-5.3) |
| Belarus | T2D | -3.1 (-5.4,-1.4) | -1.8 (-3.5,-0.8) | -1.5 (-3.3,-0.8) | -7.5 (-36.0,29.3) | -15.9 (-77.6,46.7) | -0.7 (-1.8,-0.2) |
| | CVD | -1.1 (-1.9,-0.6) | -1.1 (-2.1,-0.6) | -0.8 (-1.5,-0.4) | -106 (-210,-37.9) | -1278 (-2604,-446) | -40.4 (-86.7,-10.4) |
| Belgium | T2D | 1.4 (0.5,2.8) | 1.4 (0.8,2.4) | -0.5 (-1.2,-0.2) | 273 (220,361) | 341 (263,490) | -4.0 (-6.5,-3.2) |
| | CVD | -0.4 (-1.1,-0.1) | -0.9 (-1.5,-0.7) | -0.7 (-1.2,-0.6) | -166 (-262,-129) | -2194 (-3267,-1844) | -83.8 (-127,-71.2) |
| Belize | T2D | 9.3 (6.6,12.1) | 7.2 (5.0,9.8) | 5.5 (3.7,7.6) | 1248 (895,1693) | 3061 (2103,4291) | 42.1 (28.6,59.2) |
| | CVD | 5.0 (3.4,7.3) | 3.7 (2.5,5.2) | 2.6 (1.7,3.7) | 312 (210,469) | -397 (-765,-201) | -17.9 (-33.6,-10.6) |
| Benin | T2D | 10.7 (6.7,15.6) | 8.2 (5.1,12.3) | 5.2 (2.9,8.2) | 646 (434,929) | 1504 (968,2233) | 17.4 (10.0,27.8) |
| | CVD | 5.3 (3.1,8.5) | 4.3 (2.5,6.8) | 3.0 (1.7,5.1) | 178 (91.6,306) | 814 (432,1369) | 24.4 (12.4,42.6) |
| Bhutan | T2D | 5.9 (2.9,10.9) | 3.5 (1.6,7.0) | 2.0 (0.7,4.6) | 248 (125,452) | 542 (267,1050) | 9.6 (4.2,21.3) |
| | CVD | 2.4 (1.0,5.3) | 2.3 (1.0,4.6) | 1.5 (0.6,3.4) | 194 (87.4,429) | 775 (326,1637) | 28.8 (12.7,64.8) |
| Bolivia | T2D | -6.0 (-7.6,-4.3) | -5.7 (-7.4,-4.1) | -5.5 (-7.4,-3.9) | 703 (505,924) | 923 (588,1411) | 7.9 (0.3,20.1) |
| | CVD | -3.9 (-5.3,-2.7) | -5.5 (-7.1,-4.1) | -4.3 (-5.6,-3.1) | 16.8 (-30.8,75.3) | -2798 (-3708,-2061) | -79.8 (-107,-57.6) |
| Bosnia and Herzegovina | T2D | 4.2 (2.7,7.2) | 2.3 (1.5,4.0) | 1.6 (1.1,2.9) | 357 (238,597) | 835 (570,1386) | 16.9 (11.5,28.5) |
| | CVD | 1.6 (1.0,2.7) | 1.2 (0.8,2.1) | 0.9 (0.6,1.5) | 192 (130,313) | 1390 (911,2308) | 64.8 (42.6,106) |
| Botswana | T2D | 24.2 (17.2,30.8) | 18.9 (12.6,25.5) | 14.9 (9.2,21.7) | 1269 (912,1623) | 3787 (2544,5113) | 84.8 (51.7,126) |
| | CVD | 14.0 (9.3,19.3) | 12.6 (8.1,17.4) | 9.8 (5.8,14.3) | 830 (550,1152) | 2725 (1585,3905) | 93.0 (52.8,139) |
| Brazil | T2D | -9.7 (-11.8,-7.8) | -7.9 (-9.4,-6.5) | -6.6 (-8.1,-5.6) | -30.3 (-97.4,96.2) | -487 (-662,-233) | -13.8 (-17.3,-10.1) |
| | CVD | -5.0 (-6.3,-4.0) | -5.0 (-6.1,-4.1) | -3.8 (-4.7,-3.2) | -140 (-185,-96.5) | -3073 (-3689,-2611) | -95.1 (-116,-80.1) |
| Brunei | T2D | 8.3 (5.6,12.0) | 5.6 (3.8,8.6) | 3.7 (2.5,5.8) | 1289 (877,1846) | 1742 (1158,2670) | 15.2 (9.8,24.0) |
| | CVD | 2.8 (1.8,4.4) | 3.9 (2.6,6.1) | 3.0 (2.0,4.7) | 61.3 (39.8,96.3) | 760 (459,1268) | 20.6 (12.9,34.6) |
| Bulgaria | T2D | 0.4 (-0.1,1.5) | 0.0 (-0.4,0.3) | -0.2 (-0.5,-0.1) | 92.8 (64.9,188) | 171 (115,339) | 1.5 (0.7,2.5) |
| | CVD | -0.1 (-0.3,0.0) | -0.2 (-0.4,-0.1) | -0.2 (-0.4,-0.1) | 16.5 (-2.0,45.1) | -261 (-602,-129) | -3.0 (-13.6,1.5) |
| Burkina Faso | T2D | 9.4 (3.2,22.1) | 7.1 (2.4,17.4) | 5.1 (1.7,13.0) | 279 (96.7,677) | 818 (271,2016) | 13.3 (4.6,34.8) |
| | CVD | 3.9 (1.3,10.4) | 3.2 (1.1,8.7) | 2.4 (0.8,6.7) | 139 (44.8,382) | 771 (250,2091) | 26.3 (8.7,75.5) |
| Burundi | T2D | 9.0 (4.8,14.1) | 6.4 (2.8,11.1) | 4.5 (1.5,8.8) | 229 (143,344) | 439 (-11.6,961) | 3.1 (-11.1,15.4) |
| | CVD | 4.3 (1.6,8.4) | 4.3 (1.6,8.1) | 3.2 (1.2,6.7) | 97.4 (-17.9,249) | 160 (-865,1055) | -0.1 (-34.1,29.3) |
| Cambodia | T2D | 11.5 (8.4,15.8) | 7.9 (5.8,11.6) | 5.8 (4.2,8.9) | 474 (347,657) | 1121 (817,1666) | 19.9 (14.6,31.8) |
| | CVD | 4.5 (3.3,7.0) | 5.2 (3.7,7.8) | 3.5 (2.6,5.5) | 167 (121,261) | 1948 (1396,3018) | 57.0 (41.9,90.7) |
| Cameroon | T2D | 18.3 (12.7,24.3) | 14.3 (9.9,19.8) | 10.7 (6.9,15.7) | 751 (521,998) | 2615 (1811,3665) | 49.2 (32.3,73.9) |
| | CVD | 8.9 (5.9,13.0) | 7.9 (5.2,11.8) | 6.0 (3.8,9.5) | 383 (251,564) | 2644 (1730,3945) | 84.2 (53.8,134) |
| Canada | T2D | -1.3 (-2.2,-0.4) | -0.5 (-1.1,0.1) | -0.9 (-1.5,-0.6) | 309 (241,471) | 300 (229,472) | -2.0 (-3.2,-1.4) |
| | CVD | -0.9 (-1.4,-0.5) | -1.3 (-1.9,-1.0) | -1.1 (-1.6,-0.9) | -134 (-189,-104) | -1540 (-2081,-1317) | -56.7 (-78.3,-49.1) |
| Cape Verde | T2D | 3.9 (1.7,7.5) | 2.4 (0.9,5.2) | 1.2 (0.1,2.9) | 337 (208,561) | 891 (550,1495) | 14.0 (8.1,24.7) |
| | CVD | 1.6 (0.5,3.4) | 1.1 (0.2,2.6) | 0.7 (0.1,1.8) | 57.4 (-14.0,165) | 532 (172,1220) | 19.8 (6.2,47.1) |
| Central African Republic | T2D | 2.3 (0.4,5.0) | 1.6 (0.2,3.7) | 1.0 (-0.2,2.9) | 300 (190,516) | 754 (410,1400) | 8.2 (2.2,19.6) |

Supplementary Data 2. Differences in proportional and absolute T2D and CVD burdens attributable to SSBs from 1990 to 2020 globally, regionally, and nationally (continued).

| | | Difference in proportional burden (1990-2020) % § | | | Difference in absolute burden per 1M adults (1990-2020) §, ƒ | | |
|---|---|---|---|---|---|---|---|
| Location | Disease | Incidence | DALYs | Deaths | Incidence | DALYs | Deaths |
| | CVD | 0.8 (-0.2,2.4) | 0.8 (-0.4,2.7) | 0.6 (-0.3,2.1) | 54.8 (10.9,135) | 395 (-100,1338) | 9.8 (-5.7,34.5) |
| Chad | T2D | 7.3 (4.0,11.2) | 5.5 (3.0,8.6) | 3.8 (1.9,6.6) | 305 (200,428) | 790 (492,1192) | 10.5 (5.6,18.7) |
| | CVD | 3.2 (1.4,6.1) | 2.8 (1.2,5.1) | 2.0 (0.7,4.2) | 48.3 (-35.1,154) | 524 (24.8,1144) | 12.2 (-5.2,36.3) |
| Chile | T2D | 6.6 (4.6,9.2) | 4.9 (3.5,6.5) | 1.7 (0.6,2.7) | 997 (762,1318) | 1187 (903,1580) | 4.2 (2.2,6.7) |
| | CVD | 3.9 (2.9,5.5) | 3.6 (2.7,4.9) | 2.2 (1.6,3.1) | 108 (70.8,159) | -263 (-516,-123) | -14.7 (-26.8,-9.0) |
| China | T2D | -0.3 (-1.2,-0.2) | -0.2 (-0.5,-0.1) | -0.1 (-0.3,-0.1) | -0.2 (-16.9,13.7) | 12.2 (-0.5,37.9) | 0.0 (-0.1,0.0) |
| | CVD | -0.1 (-0.2,-0.1) | -0.1 (-0.3,-0.1) | -0.1 (-0.2,-0.1) | 4.6 (0.6,8.1) | -7.6 (-59.4,4.0) | 0.5 (-0.3,0.9) |
| Colombia | T2D | 4.2 (2.4,6.2) | 3.1 (1.5,4.7) | 0.6 (-0.6,2.1) | 793 (627,972) | 1507 (1147,1949) | 2.9 (0.2,6.4) |
| | CVD | 1.8 (0.4,3.3) | -0.1 (-1.1,1.2) | -0.5 (-1.3,0.6) | 216 (125,334) | -1653 (-2204,-1119) | -19.9 (-35.2,-5.1) |
| Comoros | T2D | 7.1 (3.8,11.1) | 4.4 (1.5,8.0) | 2.4 (-0.2,5.6) | 348 (239,490) | 806 (365,1351) | 9.6 (-0.5,21.6) |
| | CVD | 3.3 (1.0,6.1) | 3.0 (0.8,5.7) | 2.0 (0.3,4.2) | 142 (34.5,289) | 352 (-281,1030) | 15.0 (-5.3,37.9) |
| Congo, Dem. Rep. | T2D | 2.3 (0.8,6.0) | 1.4 (0.5,3.9) | 0.8 (0.2,2.4) | 136 (46.7,368) | 259 (85.7,713) | 1.8 (-0.2,6.4) |
| | CVD | 0.7 (0.2,2.3) | 0.6 (0.1,2.1) | 0.4 (0.1,1.2) | 9.7 (-8.2,52.3) | -9.8 (-276,178) | -0.7 (-9.6,4.9) |
| Congo, Rep. | T2D | 20.6 (14.6,27.1) | 16.4 (11.2,22.6) | 13.0 (8.7,19.1) | 941 (665,1257) | 3437 (2331,4810) | 65.0 (42.1,96.8) |
| | CVD | 10.5 (6.9,15.4) | 10.1 (6.6,14.5) | 7.9 (5.1,12.1) | 491 (320,727) | 3594 (2249,5311) | 113 (69.4,180) |
| Costa Rica | T2D | -8.9 (-12.1,-5.9) | -7.0 (-9.7,-4.5) | -6.6 (-9.3,-4.3) | 276 (137,485) | 568 (276,1048) | 1.2 (-1.2,5.6) |
| | CVD | -4.6 (-6.8,-2.8) | -4.0 (-5.8,-2.5) | -3.1 (-4.7,-2.0) | -89.7 (-169,6.4) | -1787 (-2542,-1209) | -66.5 (-96.3,-43.6) |
| Cote d'Ivoire | T2D | 8.9 (5.3,13.7) | 7.1 (4.1,11.1) | 4.9 (2.4,8.2) | 524 (353,741) | 1467 (967,2147) | 22.3 (13.9,35.0) |
| | CVD | 4.5 (2.2,7.6) | 4.0 (1.9,6.9) | 2.9 (1.4,5.3) | 217 (117,351) | 1487 (817,2517) | 46.5 (24.9,80.8) |
| Croatia | T2D | 0.9 (0.4,1.8) | 0.1 (-0.6,0.4) | -0.3 (-0.9,0.0) | 139 (101,210) | 306 (223,457) | 5.1 (3.6,7.8) |
| | CVD | 0.1 (-0.2,0.2) | -0.1 (-0.5,0.0) | -0.1 (-0.3,0.0) | 9.5 (-16.3,30.8) | -701 (-1299,-486) | -16.4 (-33.1,-10.1) |
| Cuba | T2D | -10.1 (-15.4,-4.7) | -6.7 (-11.1,-2.6) | -6.7 (-11.1,-3.0) | 338 (105,616) | 41.7 (-522,686) | -31.2 (-45.3,-20.1) |
| | CVD | -5.2 (-8.4,-2.3) | -6.1 (-9.2,-3.3) | -4.7 (-7.3,-2.4) | -225 (-460,16.4) | -4954 (-7070,-3070) | -177 (-269,-97.1) |
| Cyprus | T2D | 3.1 (1.5,5.7) | 1.5 (0.7,2.8) | 0.2 (0.0,0.5) | 226 (118,427) | 122 (41.1,255) | -7.4 (-16.6,-3.5) |
| | CVD | 0.3 (0.1,1.0) | 0.5 (0.2,1.1) | 0.2 (0.1,0.5) | 5.9 (-5.9,27.7) | -744 (-1528,-367) | -30.6 (-64.4,-15.1) |
| Czech Republic | T2D | -1.2 (-2.1,-0.7) | -0.9 (-1.7,-0.7) | -0.9 (-1.5,-0.7) | 47.1 (25.1,95.3) | 83.9 (41.7,156) | 1.0 (0.0,1.9) |
| | CVD | -0.5 (-0.8,-0.3) | -0.7 (-1.1,-0.5) | -0.5 (-0.8,-0.4) | -104 (-155,-77.9) | -2098 (-3011,-1654) | -78.1 (-112,-60.5) |
| Denmark | T2D | 1.3 (0.6,2.3) | 0.6 (0.2,1.0) | 0.0 (-0.4,0.1) | 131 (102,197) | 155 (122,223) | 1.4 (0.9,2.3) |
| | CVD | 0.6 (0.5,1.1) | 0.2 (0.0,0.3) | 0.0 (-0.1,0.1) | 14.7 (6.9,32.3) | -1051 (-1689,-855) | -43.9 (-63.7,-36.0) |
| Djibouti | T2D | 17.0 (10.6,23.4) | 15.0 (8.6,21.6) | 13.0 (6.8,19.2) | 907 (680,1107) | 4110 (2913,5359) | 93.9 (63.0,128) |
| | CVD | 11.4 (5.8,17.8) | 11.6 (6.0,17.8) | 9.8 (5.0,15.6) | 950 (630,1312) | 5896 (3922,8242) | 181 (115,261) |
| Dominica | T2D | -7.2 (-9.7,-4.6) | -4.1 (-6.2,-2.4) | -3.7 (-5.6,-2.2) | 397 (205,712) | 300 (-158,1105) | -25.8 (-44.2,-10.3) |
| | CVD | -3.5 (-5.2,-2.1) | -2.9 (-4.3,-1.7) | -2.4 (-3.7,-1.5) | -132 (-232,-24.1) | -2523 (-3667,-1607) | -116 (-171,-74.2) |
| Dominican Republic | T2D | 11.6 (7.2,16.8) | 8.3 (5.3,12.5) | 6.2 (3.8,9.5) | 1221 (898,1654) | 2987 (2181,4142) | 38.7 (27.2,55.3) |
| | CVD | 4.7 (2.7,7.3) | 5.0 (2.9,8.2) | 3.9 (2.3,6.2) | 470 (320,693) | 3982 (2766,5889) | 143 (98.1,209) |
| Ecuador | T2D | -13.8 (-18.8,-9.1) | -13.2 (-17.4,-9.1) | -12.4 (-16.4,-8.6) | 901 (598,1282) | 1224 (579,2082) | 4.4 (-5.9,18.1) |
| | CVD | -9.3 (-12.5,-6.2) | -10.1 (-12.9,-7.1) | -7.7 (-10.4,-5.4) | -131 (-244,7.8) | -2742 (-3492,-1977) | -83.1 (-115,-54.1) |
| Egypt, Arab Rep. | T2D | -0.5 (-1.1,0.3) | -0.8 (-1.3,-0.3) | -0.9 (-1.5,-0.4) | 398 (299,555) | 638 (476,931) | 9.1 (6.5,14.1) |
| | CVD | -0.4 (-0.9,0.0) | -0.7 (-1.2,-0.4) | -0.4 (-0.7,-0.2) | 38.8 (-10.3,122) | -915 (-1500,-549) | -21.7 (-35.9,-11.6) |

Supplementary Data 2. Differences in proportional and absolute T2D and CVD burdens attributable to SSBs from 1990 to 2020 globally, regionally, and nationally (continued).

| | | Difference in proportional burden (1990-2020) % § | | | Difference in absolute burden per 1M adults (1990-2020) §, f | | |
|---|---|---|---|---|---|---|---|
| Location | Disease | Incidence | DALYs | Deaths | Incidence | DALYs | Deaths |
| El Salvador | T2D | 9.6 (6.8,12.9) | 5.7 (3.9,8.4) | 4.1 (2.7,6.5) | 877 (636,1214) | 3057 (2132,4469) | 63.0 (43.4,94.7) |
| | CVD | 3.5 (2.1,5.5) | 2.9 (1.6,4.6) | 1.9 (1.1,3.2) | 265 (170,406) | 952 (506,1579) | 49.4 (32.1,78.0) |
| Equatorial Guinea | T2D | 11.6 (6.9,17.7) | 8.5 (4.9,13.4) | 5.4 (2.6,9.3) | 540 (351,796) | 1077 (563,1763) | 10.6 (-1.4,24.1) |
| | CVD | 5.9 (3.4,10.0) | 5.2 (2.7,8.6) | 3.5 (1.7,6.5) | 105 (15.3,217) | 132 (-753,799) | 3.2 (-28.8,28.0) |
| Eritrea | T2D | 12.0 (7.3,17.1) | 9.0 (4.8,13.5) | 6.1 (2.4,10.6) | 1079 (751,1439) | 3777 (2468,5185) | 68.3 (39.9,106) |
| | CVD | 5.4 (2.2,9.2) | 5.6 (2.0,9.9) | 4.0 (1.0,7.3) | 487 (270,764) | 3585 (1910,5785) | 104 (56.4,170) |
| Estonia | T2D | 1.2 (0.5,2.1) | 0.3 (-0.2,0.8) | 0.1 (-0.4,0.3) | 105 (80.3,155) | 241 (191,367) | 3.9 (3.1,6.3) |
| | CVD | 0.3 (0.2,0.7) | 0.0 (-0.3,0.2) | 0.0 (-0.2,0.1) | 63.8 (34.8,116) | -1386 (-2498,-1071) | -50.7 (-83.5,-38.6) |
| Ethiopia (excludes Eritrea) | T2D | 11.8 (9.2,14.7) | 8.1 (6.1,10.8) | 4.6 (2.8,6.9) | 204 (157,260) | -234 (-647,-28.9) | -13.6 (-26.8,-7.8) |
| | CVD | 5.0 (3.4,7.2) | 4.3 (2.7,6.1) | 2.6 (1.3,4.1) | 69.6 (13.0,122) | -229 (-706,-1.7) | -4.5 (-17.5,2.1) |
| Fiji | T2D | 6.6 (4.5,9.4) | 3.8 (2.6,5.8) | 2.9 (2.0,4.7) | 1149 (804,1600) | 5158 (3679,7808) | 122 (86.2,187) |
| | CVD | 2.4 (1.6,3.8) | 3.0 (2.0,4.7) | 2.2 (1.5,3.6) | 190 (134,297) | 2904 (1935,4557) | 86.0 (59.9,135) |
| Finland | T2D | -0.5 (-1.3,0.1) | -0.4 (-0.9,-0.1) | -0.2 (-0.4,-0.1) | 103 (78.4,166) | 146 (113,245) | -0.3 (-0.5,-0.1) |
| | CVD | -0.9 (-1.7,-0.7) | -0.8 (-1.3,-0.6) | -0.6 (-1.0,-0.5) | -147 (-251,-117) | -1487 (-2319,-1233) | -49.7 (-77.6,-41.6) |
| France | T2D | 2.3 (1.7,3.3) | 1.1 (0.9,1.7) | 0.2 (0.0,0.4) | 135 (113,187) | 198 (166,275) | 1.6 (1.3,2.4) |
| | CVD | 0.1 (-0.2,0.4) | 0.1 (-0.1,0.3) | 0.0 (-0.1,0.1) | 23.8 (4.4,51.2) | -358 (-554,-303) | -13.6 (-20.5,-11.7) |
| Gabon | T2D | -6.9 (-11.8,-2.0) | -6.8 (-12.1,-2.4) | -6.1 (-11.0,-2.0) | 16.6 (-133,244) | -1688 (-2878,-670) | -65.8 (-107,-35.1) |
| | CVD | -4.4 (-8.3,-1.4) | -4.5 (-8.3,-1.6) | -3.9 (-7.4,-1.5) | -422 (-686,-239) | -3899 (-6022,-2318) | -147 (-231,-86.9) |
| Gambia, The | T2D | -27.7 (-33.9,-20.6) | -25.9 (-32.3,-18.8) | -22.8 (-30.1,-15.9) | -39.2 (-184,149) | -621 (-1096,-20.0) | -21.7 (-33.0,-7.4) |
| | CVD | -18.8 (-26.0,-13.0) | -16.8 (-23.7,-11.3) | -14.3 (-21.4,-9.1) | -663 (-946,-431) | -3915 (-5925,-2330) | -134 (-208,-77.5) |
| Georgia | T2D | 6.4 (4.3,9.9) | 3.4 (2.4,5.6) | 2.4 (1.6,3.8) | 247 (165,387) | 560 (379,920) | 8.7 (5.7,14.2) |
| | CVD | 1.9 (1.3,3.1) | 1.7 (1.2,2.8) | 1.2 (0.8,1.9) | 260 (176,426) | 1533 (1022,2545) | 62.6 (41.0,97.9) |
| Germany | T2D | 1.6 (1.0,2.5) | 0.9 (0.6,1.5) | -0.1 (-0.4,0.2) | 223 (182,295) | 249 (200,365) | -0.6 (-1.7,0.2) |
| | CVD | -0.1 (-0.3,0.1) | -0.3 (-0.5,-0.1) | -0.2 (-0.4,-0.1) | -90.4 (-142,-67.4) | -1567 (-2271,-1311) | -58.3 (-82.8,-49.3) |
| Ghana | T2D | 8.7 (5.8,12.9) | 6.1 (3.6,9.5) | 4.4 (2.4,7.5) | 613 (448,832) | 2050 (1424,2857) | 38.1 (25.6,58.4) |
| | CVD | 4.8 (3.0,7.4) | 3.2 (1.7,5.3) | 2.3 (1.1,4.1) | 254 (162,400) | 622 (27.9,1297) | 21.5 (3.1,45.6) |
| Greece | T2D | 1.6 (1.0,2.5) | 0.6 (0.2,1.2) | 0.1 (-0.1,0.4) | 106 (80.4,153) | 250 (197,360) | 1.2 (0.9,2.1) |
| | CVD | -0.1 (-0.3,0.1) | 0.1 (-0.1,0.3) | -0.1 (-0.2,0.0) | -16.7 (-36.8,-4.9) | -498 (-738,-393) | -16.9 (-24.8,-13.2) |
| Grenada | T2D | -10.0 (-14.7,-5.0) | -6.2 (-10.0,-2.3) | -4.1 (-7.5,-1.0) | 604 (259,1034) | -1079 (-2450,331) | -85.3 (-132,-45.5) |
| | CVD | -3.0 (-6.0,-0.2) | -4.5 (-7.2,-1.7) | -2.8 (-5.2,-0.7) | -323 (-553,-120) | -6823 (-9168,-5063) | -275 (-389,-197) |
| Guatemala | T2D | 15.8 (12.6,19.7) | 10.1 (7.8,13.4) | 8.0 (6.2,10.7) | 1285 (1024,1600) | 4238 (3335,5411) | 81.6 (63.2,105) |
| | CVD | 6.5 (5.0,8.7) | 6.0 (4.7,7.9) | 3.8 (2.8,5.1) | 252 (189,337) | 897 (606,1255) | 35.5 (26.2,49.3) |
| Guinea | T2D | 5.0 (3.0,7.9) | 3.6 (2.1,5.8) | 2.4 (1.2,4.2) | 167 (107,256) | 516 (316,840) | 8.9 (5.0,15.9) |
| | CVD | 1.9 (1.0,3.7) | 1.7 (0.9,3.3) | 1.2 (0.6,2.4) | 69.1 (26.3,144) | 532 (259,1050) | 16.7 (7.8,34.8) |
| Guinea-Bissau | T2D | 26.9 (19.0,35.5) | 21.5 (14.8,29.1) | 16.7 (11.1,23.8) | 888 (624,1185) | 3088 (2107,4198) | 56.0 (37.1,81.0) |
| | CVD | 12.6 (8.4,18.2) | 11.9 (7.9,17.5) | 9.4 (6.0,14.1) | 465 (306,681) | 4502 (2921,6678) | 135 (86.1,207) |
| Guyana | T2D | -8.9 (-11.2,-6.7) | -7.4 (-9.8,-5.4) | -6.4 (-8.8,-4.5) | 807 (475,1284) | 1580 (815,2804) | 2.0 (-7.3,16.9) |

Supplementary Data 2. Differences in proportional and absolute T2D and CVD burdens attributable to SSBs from 1990 to 2020 globally, regionally, and nationally (continued).

| | | Difference in proportional burden (1990-2020) % § | | | Difference in absolute burden per 1M adults (1990-2020) §, f | | |
|---|---|---|---|---|---|---|---|
| Location | Disease | Incidence | DALYs | Deaths | Incidence | DALYs | Deaths |
| | CVD | -4.9 (-6.8,-3.2) | -5.4 (-7.3,-3.7) | -4.1 (-5.8,-2.9) | -135 (-231,-37.6) | -5353 (-7374,-3715) | -162 (-233,-110) |
| Haiti | T2D | 3.1 (1.7,5.2) | 2.0 (1.0,3.5) | 1.0 (0.3,2.4) | 679 (467,1020) | 1069 (689,1784) | 7.2 (1.2,18.4) |
| | CVD | 1.4 (0.5,2.9) | 1.1 (0.1,2.4) | 0.7 (0.2,1.8) | 85.3 (32.1,172) | 198 (-665,900) | 5.1 (-18.5,30.8) |
| Honduras | T2D | 12.1 (9.8,14.6) | 7.5 (5.8,9.4) | 4.7 (3.4,6.6) | 1182 (959,1445) | 2403 (1907,3010) | 30.5 (23.0,40.6) |
| | CVD | 5.1 (3.8,7.0) | 3.5 (2.2,5.0) | 3.0 (2.1,4.3) | 308 (233,424) | 2359 (1701,3213) | 101 (75.8,140) |
| Hungary | T2D | -0.5 (-1.3,0.1) | -0.7 (-1.2,-0.5) | -0.8 (-1.4,-0.6) | 77.8 (52.1,135) | 140 (90.8,229) | 1.2 (0.4,2.2) |
| | CVD | -0.5 (-0.9,-0.4) | -0.8 (-1.3,-0.6) | -0.6 (-0.8,-0.4) | -117 (-180,-85.6) | -2187 (-3100,-1683) | -62.6 (-85.6,-47.4) |
| Iceland | T2D | 1.3 (0.0,2.8) | 1.4 (0.7,2.4) | 0.1 (-0.3,0.4) | 276 (214,373) | 356 (276,497) | 0.3 (-0.1,0.7) |
| | CVD | 0.3 (-0.3,0.9) | 0.2 (-0.1,0.5) | -0.1 (-0.3,0.1) | -16.4 (-56.3,18.3) | -1184 (-1677,-976) | -45.2 (-66.7,-37.2) |
| India | T2D | 0.1 (0.0,0.3) | 0.0 (0.0,0.0) | 0.0 (-0.1,0.0) | 14.0 (8.1,37.6) | 30.6 (17.9,67.0) | 0.5 (0.3,0.9) |
| | CVD | 0.0 (0.0,0.1) | 0.0 (0.0,0.1) | 0.0 (0.0,0.1) | 7.5 (3.9,16.7) | 46.6 (23.5,99.7) | 1.8 (1.0,3.5) |
| Indonesia | T2D | 1.0 (0.6,2.0) | 0.7 (0.4,1.4) | 0.5 (0.3,1.0) | 75.8 (53.5,133) | 156 (113,274) | 2.5 (1.9,4.6) |
| | CVD | 0.4 (0.3,0.8) | 0.5 (0.2,0.9) | 0.3 (0.2,0.6) | 28.4 (20.1,50.8) | 394 (269,721) | 11.6 (8.3,20.3) |
| Iran, Islamic Rep. | T2D | 0.3 (-0.6,1.5) | 0.2 (-0.5,0.8) | -0.2 (-0.9,0.3) | 310 (256,428) | 510 (425,708) | 6.1 (4.9,8.9) |
| | CVD | 0.1 (-0.7,0.8) | -0.3 (-0.9,0.1) | -0.5 (-1.1,-0.2) | 76.2 (-10.4,182) | -1057 (-1663,-817) | -25.4 (-45.2,-18.3) |
| Iraq | T2D | -2.9 (-4.8,-1.2) | -1.4 (-2.7,-0.2) | -1.7 (-3.2,-0.8) | 971 (620,1531) | 1002 (613,1648) | -2.9 (-8.5,2.6) |
| | CVD | -1.0 (-2.1,0.0) | -1.7 (-2.8,-0.9) | -1.2 (-2.1,-0.7) | -107 (-264,35.5) | -2367 (-3723,-1488) | -75.7 (-124,-45.1) |
| Ireland | T2D | -1.0 (-1.7,-0.3) | 0.3 (-0.2,0.9) | -1.0 (-1.7,-0.8) | 124 (91.6,169) | 41.2 (8.3,87.0) | -4.4 (-6.6,-3.4) |
| | CVD | 0.0 (-0.4,0.4) | -0.7 (-1.1,-0.5) | -0.8 (-1.3,-0.6) | -138 (-213,-103) | -3103 (-4369,-2445) | -121 (-173,-95.8) |
| Israel | T2D | -3.8 (-5.2,-2.3) | -3.6 (-5.1,-2.8) | -4.0 (-6.0,-3.3) | 154 (98.7,270) | -144 (-267,-4.9) | -9.9 (-16.4,-7.6) |
| | CVD | -1.9 (-2.8,-1.1) | -3.4 (-4.7,-2.8) | -3.1 (-4.5,-2.6) | -285 (-377,-221) | -5115 (-6603,-4236) | -222 (-297,-182) |
| Italy | T2D | 0.1 (-0.4,0.7) | 0.1 (-0.2,0.2) | -0.4 (-0.6,-0.3) | 51.8 (37.0,96.1) | 35.6 (13.5,64.7) | -1.9 (-3.1,-1.5) |
| | CVD | 0.1 (-0.1,0.3) | -0.4 (-0.6,-0.3) | -0.3 (-0.5,-0.2) | -17.6 (-34.6,-3.1) | -518 (-752,-444) | -17.4 (-25.8,-14.8) |
| Jamaica | T2D | -2.1 (-3.5,-0.5) | -1.9 (-3.2,-0.9) | -3.3 (-4.5,-2.4) | 628 (461,848) | 474 (135,868) | -18.2 (-29.3,-10.1) |
| | CVD | -1.4 (-2.4,-0.4) | -1.1 (-1.7,-0.6) | -1.2 (-1.7,-0.7) | 4.7 (-67.0,96.6) | -952 (-1299,-715) | -43.3 (-59.2,-32.0) |
| Japan | T2D | -1.4 (-2.4,-0.8) | -1.2 (-1.8,-0.8) | -1.0 (-1.7,-0.8) | 35.9 (10.4,85.7) | 99.3 (61.9,202) | -1.9 (-2.8,-1.6) |
| | CVD | -0.7 (-1.1,-0.5) | -0.6 (-0.9,-0.5) | -0.4 (-0.6,-0.3) | 3.1 (-7.3,12.8) | -308 (-419,-254) | -9.5 (-12.9,-7.6) |
| Jordan | T2D | 5.6 (3.5,8.6) | 4.5 (3.0,6.8) | 2.2 (0.9,4.3) | 2117 (1580,2815) | 2215 (1631,3064) | 3.7 (-2.8,11.7) |
| | CVD | 3.0 (1.7,5.1) | 1.7 (0.7,3.2) | 1.4 (0.6,2.6) | 628 (410,1011) | -1643 (-2335,-1108) | -46.6 (-66.2,-30.0) |
| Kazakhstan | T2D | 2.4 (1.1,4.5) | 1.6 (0.9,2.9) | 0.8 (0.4,1.7) | 182 (113,298) | 260 (168,434) | 1.4 (0.9,2.6) |
| | CVD | 0.8 (0.5,1.5) | 0.4 (0.1,1.0) | 0.4 (0.2,0.8) | 76.8 (42.4,150) | 92.6 (-228,492) | 9.0 (-0.6,25.1) |
| Kenya | T2D | -1.5 (-2.8,-0.8) | -1.4 (-2.6,-0.7) | -1.0 (-2.0,-0.5) | -4.3 (-14.4,4.5) | -27.2 (-69.1,25.1) | -1.0 (-2.2,0.2) |
| | CVD | -0.8 (-1.7,-0.3) | -0.5 (-1.3,-0.2) | -0.4 (-0.8,-0.1) | -33.5 (-74.9,-14.7) | -20.6 (-84.4,37.0) | -0.7 (-2.3,0.9) |
| Kiribati | T2D | 3.9 (2.3,6.2) | 2.1 (1.3,3.7) | 1.5 (0.9,2.7) | 560 (350,898) | 1770 (1119,3066) | 32.5 (19.8,58.3) |
| | CVD | 1.1 (0.7,1.9) | 1.6 (1.0,3.1) | 1.2 (0.8,2.3) | 58.8 (35.3,102) | 1262 (784,2355) | 30.6 (19.0,58.3) |
| Korea, Rep. | T2D | -0.5 (-1.6,-0.2) | -0.2 (-0.7,-0.1) | -0.5 (-1.0,-0.4) | 119 (100,196) | 147 (121,240) | -0.8 (-1.8,-0.5) |
| | CVD | -0.1 (-0.2,0.0) | -0.2 (-0.5,-0.1) | -0.1 (-0.3,-0.1) | -6.4 (-12.1,-3.8) | -118 (-210,-98.0) | -2.5 (-4.5,-2.0) |
| Kuwait | T2D | 22.1 (16.0,28.8) | 18.6 (13.5,24.9) | 11.0 (7.5,16.2) | 3926 (2795,5293) | 4719 (3358,6492) | 26.0 (17.6,38.9) |
| | CVD | 13.3 (9.2,19.0) | 13.5 (9.3,19.2) | 10.2 (6.9,14.8) | 1750 (1199,2495) | 4048 (2797,5906) | 115 (77.7,170) |
| Kyrgyz Republic | T2D | 4.3 (1.3,7.8) | 2.8 (1.0,5.3) | 1.9 (0.3,4.2) | 297 (197,425) | 419 (274,653) | 3.0 (1.7,5.4) |
| | CVD | 1.4 (0.5,2.8) | 1.0 (0.1,2.4) | 0.9 (0.2,1.9) | 67.4 (-18.2,172) | -127 (-946,747) | 3.0 (-24.5,34.7) |
| Lao PDR | T2D | 2.5 (1.8,4.2) | 1.7 (1.2,3.0) | 1.2 (0.9,2.2) | 142 (105,229) | 267 (197,476) | 3.8 (2.7,7.2) |

Supplementary Data 2. Differences in proportional and absolute T2D and CVD burdens attributable to SSBs from 1990 to 2020 globally, regionally, and nationally (continued).

| | | Difference in proportional burden (1990-2020) % § | | | Difference in absolute burden per 1M adults (1990-2020) §, ƒ | | |
|---|---|---|---|---|---|---|---|
| Location | Disease | Incidence | DALYs | Deaths | Incidence | DALYs | Deaths |
| | CVD | 0.9 (0.7,1.5) | 1.3 (0.9,2.2) | 0.8 (0.6,1.3) | 31.3 (23.0,53.1) | 495 (252,959) | 13.1 (7.6,23.5) |
| Latvia | T2D | 0.0 (-0.6,0.4) | -0.2 (-0.7,-0.1) | -0.3 (-0.7,-0.2) | 71.0 (53.5,111) | 196 (153,304) | 3.6 (2.8,5.2) |
| | CVD | -0.1 (-0.3,0.0) | -0.3 (-0.7,-0.2) | -0.2 (-0.4,-0.2) | 3.6 (-14.3,17.2) | -868 (-1538,-677) | -24.3 (-43.1,-18.9) |
| Lebanon | T2D | 2.6 (1.4,4.6) | 0.3 (-0.8,1.3) | -1.9 (-3.4,-0.9) | 569 (427,766) | 254 (-13.9,560) | -16.9 (-26.7,-10.7) |
| | CVD | -0.5 (-1.8,0.5) | -1.9 (-3.4,-1.2) | -2.2 (-3.5,-1.5) | -349 (-642,-151) | -7250 (-9839,-5817) | -227 (-316,-183) |
| Lesotho | T2D | 16.2 (11.3,22.1) | 12.6 (8.4,18.3) | 11.1 (6.9,16.6) | 727 (521,961) | 4155 (2852,5770) | 118 (74.4,174) |
| | CVD | 8.9 (5.8,13.7) | 8.8 (5.6,13.5) | 7.1 (4.5,11.2) | 294 (175,480) | 3044 (1964,4605) | 100 (63.5,158) |
| Liberia | T2D | 21.2 (15.2,28.1) | 17.2 (12.1,23.4) | 12.4 (8.3,18.0) | 819 (583,1105) | 2377 (1672,3255) | 35.8 (23.7,52.4) |
| | CVD | 10.2 (6.6,15.2) | 9.1 (6.0,13.6) | 6.6 (4.2,10.5) | 328 (204,512) | 2112 (1358,3310) | 61.0 (37.7,98.0) |
| Libya | T2D | 2.5 (-1.0,7.4) | 2.5 (-0.2,6.1) | 1.9 (-0.7,5.0) | 1265 (858,1905) | 1877 (1295,2891) | 17.6 (11.3,28.7) |
| | CVD | 1.7 (-0.3,4.9) | 1.4 (-0.4,3.9) | 1.3 (-0.2,3.3) | 431 (194,832) | 2358 (1287,4146) | 70.4 (36.6,131) |
| Lithuania | T2D | 1.1 (0.4,1.9) | 0.4 (0.0,1.0) | 0.2 (-0.1,0.6) | 81.7 (61.3,121) | 213 (164,353) | 3.6 (2.8,5.8) |
| | CVD | 0.1 (0.0,0.3) | 0.0 (-0.3,0.2) | 0.0 (-0.1,0.2) | 53.5 (29.7,97.5) | -109 (-576,118) | 6.8 (-5.6,17.6) |
| Luxembourg | T2D | 2.7 (1.7,4.1) | 2.1 (1.4,3.2) | -0.1 (-0.5,0.2) | 227 (165,325) | 241 (171,357) | -1.8 (-2.9,-1.2) |
| | CVD | 0.9 (0.5,1.8) | 0.1 (-0.2,0.3) | -0.1 (-0.3,0.1) | -2.4 (-23.5,19.9) | -1501 (-2237,-1102) | -57.7 (-82.8,-42.1) |
| Macedonia, FYR | T2D | 12.4 (9.3,16.6) | 7.5 (5.5,10.3) | 5.3 (3.8,7.9) | 1002 (757,1356) | 2008 (1495,2766) | 34.4 (25.3,51.0) |
| | CVD | 4.9 (3.6,6.7) | 3.7 (2.7,5.2) | 2.8 (2.1,4.1) | 550 (404,762) | 4055 (2996,5689) | 178 (131,260) |
| Madagascar | T2D | 2.8 (-0.6,6.4) | 1.5 (-1.6,4.6) | 1.0 (-1.6,3.7) | 120 (64.8,190) | 33.3 (-256,307) | -6.3 (-15.3,-0.2) |
| | CVD | 0.9 (-1.6,3.4) | 1.1 (-1.2,3.8) | 1.1 (-0.9,3.2) | -32.7 (-164,61.9) | -88.5 (-909,715) | -7.4 (-36.7,12.3) |
| Malawi | T2D | -4.2 (-6.9,-2.6) | -3.8 (-6.6,-2.4) | -3.1 (-5.7,-1.9) | -38.0 (-66.1,-20.8) | -364 (-623,-219) | -10.4 (-18.4,-6.4) |
| | CVD | -2.4 (-4.4,-1.5) | -2.3 (-4.1,-1.4) | -1.8 (-3.3,-1.1) | -97.0 (-182,-57.4) | -463 (-845,-268) | -14.0 (-26.9,-8.4) |
| Malaysia | T2D | -0.3 (-1.2,0.7) | 0.2 (-0.5,0.9) | 0.2 (-0.3,0.9) | 140 (95.8,226) | 136 (76.9,272) | -0.7 (-2.0,0.5) |
| | CVD | 0.1 (-0.1,0.7) | 0.1 (-0.3,0.9) | 0.1 (-0.2,0.7) | 26.3 (15.4,64.9) | -14.5 (-209,352) | -0.2 (-5.8,10.1) |
| Maldives | T2D | -13.3 (-17.5,-9.0) | -12.1 (-16.7,-7.7) | -12.5 (-17.2,-7.7) | -149 (-221,-68.4) | -1635 (-2255,-1015) | -46.6 (-66.4,-27.6) |
| | CVD | -4.9 (-8.0,-2.9) | -6.7 (-10.2,-4.0) | -6.5 (-9.9,-3.9) | -188 (-305,-114) | -5268 (-8007,-3194) | -150 (-229,-88.9) |
| Mali | T2D | 1.9 (-0.6,5.3) | 1.3 (-0.5,3.8) | 0.6 (-0.9,2.6) | 246 (125,498) | 485 (188,1034) | 3.8 (-0.8,11.5) |
| | CVD | 0.7 (-0.6,2.5) | 0.5 (-0.8,1.9) | 0.3 (-0.6,1.3) | 13.8 (-48.6,93.1) | 51.7 (-345,393) | 1.2 (-9.2,11.2) |
| Malta | T2D | 9.1 (6.9,12.1) | 5.3 (3.9,7.4) | 1.9 (1.2,3.0) | 768 (590,1017) | 1134 (856,1557) | 6.0 (3.4,10.0) |
| | CVD | 2.1 (1.3,3.5) | 1.9 (1.3,2.9) | 1.0 (0.6,1.6) | 93.9 (58.2,159) | -964 (-1866,-642) | -28.5 (-68.1,-17.3) |
| Marshall Islands | T2D | 8.4 (5.3,13.3) | 4.5 (2.9,7.7) | 3.3 (2.1,6.1) | 1351 (920,2022) | 3837 (2645,6190) | 59.5 (39.3,105) |
| | CVD | 2.4 (1.6,4.0) | 3.5 (2.1,5.9) | 2.8 (1.9,4.8) | 109 (72.8,172) | 3113 (2063,5169) | 78.1 (53.2,131) |
| Mauritania | T2D | 20.9 (15.0,27.5) | 15.1 (10.3,21.1) | 10.6 (6.7,15.7) | 451 (320,605) | 1393 (942,1941) | 29.1 (18.0,45.4) |
| | CVD | 10.8 (7.0,16.1) | 8.5 (5.5,12.9) | 6.4 (4.0,10.0) | 382 (241,572) | 1497 (905,2398) | 57.0 (32.2,94.4) |
| Mauritius | T2D | -13.1 (-15.0,-10.3) | -12.2 (-13.4,-10.3) | -13.0 (-14.4,-11.3) | 2968 (2243,3801) | 13597 (9878,18497) | 340 (239,479) |
| | CVD | -6.8 (-8.4,-5.4) | -7.7 (-8.8,-6.6) | -8.1 (-9.4,-6.4) | -91.4 (-191,35.6) | -14146 (-16848,-11306) | -459 (-564,-344) |
| Mexico | T2D | -2.2 (-3.6,-0.7) | -1.9 (-2.7,-1.1) | -1.5 (-2.1,-0.8) | 320 (176,513) | 1586 (1197,2158) | 29.8 (22.6,42.6) |
| | CVD | -1.5 (-2.6,-0.3) | -0.4 (-0.9,0.2) | -0.4 (-0.7,0.1) | 70.6 (-2.4,162) | 1003 (784,1396) | 37.3 (29.7,51.5) |
| Micronesia, Fed. Sts. | T2D | 7.7 (4.8,11.4) | 4.4 (2.9,7.1) | 3.3 (2.1,5.5) | 849 (575,1231) | 2374 (1613,3745) | 40.2 (26.6,66.7) |
| | CVD | 2.4 (1.6,4.2) | 3.4 (2.1,5.7) | 2.6 (1.7,4.5) | 107 (67.0,184) | 2621 (1528,4547) | 67.7 (42.8,121) |

Supplementary Data 2. Differences in proportional and absolute T2D and CVD burdens attributable to SSBs from 1990 to 2020 globally, regionally, and nationally (continued).

| | | Difference in proportional burden (1990-2020) % § | | | Difference in absolute burden per 1M adults (1990-2020) §, ƒ | | |
|---|---|---|---|---|---|---|---|
| Location | Disease | Incidence | DALYs | Deaths | Incidence | DALYs | Deaths |
| Moldova | T2D | -1.8 (-3.6,-0.5) | -1.2 (-2.6,-0.5) | -1.0 (-2.4,-0.4) | 9.3 (-25.5,59.6) | 24.0 (-48.8,115) | -0.1 (-1.2,1.0) |
| | CVD | -0.7 (-1.3,-0.3) | -0.7 (-1.4,-0.3) | -0.5 (-1.0,-0.2) | -59.3 (-121,-20.1) | -898 (-1743,-485) | -33.0 (-62.9,-17.1) |
| Mongolia | T2D | -0.3 (-1.4,1.3) | 0.6 (-0.1,1.9) | 0.6 (0.0,1.9) | 214 (144,316) | 365 (249,560) | 2.7 (1.8,4.9) |
| | CVD | 0.4 (0.0,1.2) | 0.3 (-0.2,1.3) | 0.2 (-0.1,0.9) | 6.2 (-31.0,63.0) | -653 (-1111,-314) | -28.4 (-47.3,-16.8) |
| Montenegro | T2D | 1.8 (0.8,4.4) | 1.0 (0.4,2.5) | 0.6 (0.2,1.7) | 170 (71.4,407) | 345 (142,833) | 5.2 (2.2,13.3) |
| | CVD | 0.6 (0.2,1.8) | 0.3 (-0.1,1.0) | 0.3 (0.1,0.9) | 93.8 (39.7,241) | 754 (307,2026) | 40.0 (17.0,101) |
| Morocco | T2D | 2.8 (1.4,4.7) | 2.1 (1.2,3.7) | 1.4 (0.7,2.7) | 1154 (794,1668) | 1921 (1318,2849) | 16.9 (11.4,27.3) |
| | CVD | 1.2 (0.5,2.4) | 0.4 (-0.4,1.3) | 0.5 (0.0,1.2) | 369 (228,639) | 325 (-438,1368) | 37.4 (12.7,75.3) |
| Mozambique | T2D | 4.4 (2.5,7.4) | 3.2 (1.8,5.8) | 2.4 (1.3,4.5) | 110 (62.6,183) | 457 (253,808) | 8.0 (4.0,15.2) |
| | CVD | 1.6 (0.8,3.1) | 1.5 (0.8,2.9) | 1.1 (0.6,2.1) | 26.2 (-5.6,69.1) | 301 (145,590) | 8.2 (4.1,17.0) |
| Myanmar | T2D | 7.1 (5.0,10.4) | 4.8 (3.4,7.5) | 3.5 (2.4,5.6) | 522 (364,772) | 1317 (928,2066) | 24.3 (17.0,39.2) |
| | CVD | 2.7 (1.9,4.2) | 3.1 (2.1,4.8) | 2.1 (1.4,3.2) | 119 (82.2,188) | 1508 (1005,2386) | 46.5 (32.3,75.7) |
| Namibia | T2D | 6.0 (3.3,9.2) | 2.9 (0.3,5.8) | 1.2 (-1.2,3.7) | 538 (393,699) | 1621 (970,2380) | 32.2 (13.7,58.2) |
| | CVD | 2.2 (0.1,4.3) | 2.2 (0.2,4.3) | 0.9 (-0.8,2.5) | 22.9 (-119,141) | 353 (-478,1116) | 13.3 (-12.1,39.4) |
| Nepal | T2D | 4.8 (2.4,9.0) | 3.0 (1.5,6.1) | 1.9 (0.9,4.2) | 236 (120,449) | 539 (270,1092) | 7.9 (3.8,17.3) |
| | CVD | 1.8 (0.9,3.9) | 1.8 (0.9,3.9) | 1.4 (0.6,3.1) | 131 (63.4,289) | 850 (407,1820) | 28.5 (13.5,64.3) |
| Netherlands | T2D | -10.5 (-13.5,-8.3) | -5.5 (-7.5,-4.4) | -3.5 (-4.8,-2.9) | -196 (-256,-131) | -461 (-634,-354) | -11.4 (-16.0,-9.8) |
| | CVD | -3.9 (-5.4,-3.1) | -3.9 (-5.4,-3.4) | -2.8 (-3.8,-2.3) | -299 (-418,-250) | -2599 (-3521,-2230) | -98.1 (-134,-85.8) |
| New Zealand | T2D | 1.9 (1.0,3.1) | 1.4 (1.1,2.3) | 0.6 (0.3,1.0) | 142 (109,219) | 204 (158,329) | 0.9 (0.5,1.7) |
| | CVD | 0.7 (0.5,1.2) | 0.4 (0.3,0.8) | 0.2 (0.1,0.4) | 17.3 (-2.9,47.1) | -403 (-698,-309) | -14.2 (-22.8,-10.9) |
| Nicaragua | T2D | 18.6 (13.7,24.2) | 13.6 (9.6,18.0) | 10.4 (7.1,14.5) | 1463 (1123,1842) | 3296 (2460,4228) | 45.0 (32.6,60.8) |
| | CVD | 9.0 (6.2,12.5) | 8.6 (6.0,11.6) | 6.3 (4.2,8.7) | 443 (313,618) | 1919 (1346,2592) | 69.4 (48.0,97.7) |
| Niger | T2D | -6.4 (-10.7,-3.9) | -5.0 (-8.7,-3.0) | -3.7 (-6.6,-2.2) | -95.3 (-160,-55.5) | -291 (-504,-171) | -5.2 (-9.4,-3.1) |
| | CVD | -2.9 (-5.5,-1.7) | -2.5 (-4.5,-1.4) | -1.9 (-3.6,-1.1) | -104 (-201,-60.2) | -431 (-784,-247) | -13.6 (-26.2,-7.7) |
| Nigeria | T2D | 13.9 (9.5,19.7) | 10.6 (7.0,15.4) | 7.4 (4.6,11.6) | 362 (247,515) | 1082 (711,1601) | 20.7 (12.7,32.4) |
| | CVD | 6.9 (4.5,10.6) | 5.9 (3.8,9.2) | 4.3 (2.7,6.9) | 291 (188,464) | 1528 (973,2424) | 49.4 (30.3,80.4) |
| Norway | T2D | 0.7 (-0.1,2.0) | 0.6 (0.2,1.3) | -0.1 (-0.3,0.0) | 68.8 (43.4,125) | 89.3 (56.3,160) | -0.4 (-1.0,-0.3) |
| | CVD | 0.3 (0.1,0.9) | -0.2 (-0.5,-0.1) | -0.2 (-0.5,-0.2) | -51.7 (-84.7,-24.8) | -1582 (-2522,-1313) | -65.2 (-97.9,-54.9) |
| Oman | T2D | 8.9 (5.9,14.1) | 7.1 (4.6,11.3) | 4.6 (2.9,8.0) | 439 (287,710) | 563 (363,918) | 5.8 (3.1,11.1) |
| | CVD | 5.1 (3.3,8.4) | 4.4 (2.9,7.1) | 3.2 (2.0,5.3) | 219 (127,390) | -329 (-940,-27.8) | -11.7 (-31.3,-2.1) |
| Pakistan | T2D | -0.4 (-1.3,0.5) | -0.1 (-0.8,0.6) | -0.3 (-1.1,0.4) | 398 (242,638) | 739 (412,1207) | 7.0 (3.6,13.1) |
| | CVD | 1.0 (0.3,2.3) | 1.3 (0.5,2.4) | 0.9 (0.4,1.7) | 52.3 (-16.7,146) | 977 (518,1731) | 21.1 (9.7,38.4) |
| Palestine | T2D | 4.3 (2.7,6.5) | 3.4 (2.2,4.9) | 2.2 (1.4,3.4) | 646 (440,930) | 748 (501,1103) | 3.6 (1.1,7.6) |
| | CVD | 2.3 (1.4,3.7) | 2.0 (1.3,3.1) | 1.6 (1.0,2.4) | 159 (76.7,299) | -813 (-1472,-495) | -36.0 (-60.0,-22.1) |
| Panama | T2D | 5.2 (0.1,10.1) | 3.1 (-0.8,7.1) | 1.9 (-1.2,5.2) | 1102 (805,1440) | 2932 (2115,3989) | 44.4 (31.0,62.4) |
| | CVD | 2.4 (-0.4,5.5) | 2.1 (-0.4,4.6) | 1.0 (-0.8,3.0) | 296 (144,488) | -193 (-878,412) | -5.8 (-33.6,20.3) |
| Papua New Guinea | T2D | -3.7 (-7.1,-2.4) | -2.0 (-4.0,-1.4) | -1.4 (-2.9,-1.0) | -125 (-236,-79.0) | -478 (-926,-321) | -9.6 (-18.7,-6.6) |
| | CVD | -1.1 (-2.2,-0.8) | -1.4 (-3.0,-0.9) | -1.1 (-2.3,-0.7) | -33.2 (-62.9,-22.2) | -506 (-1076,-332) | -12.8 (-26.1,-8.6) |
| Paraguay | T2D | 5.5 (1.7,10.2) | 3.2 (0.7,6.9) | 2.8 (0.7,5.8) | 763 (532,1095) | 2503 (1725,3675) | 57.7 (38.4,86.5) |
| | CVD | 2.9 (0.9,5.5) | 2.5 (0.7,4.9) | 1.9 (0.6,3.8) | 112 (50.9,206) | 590 (25.5,1368) | 24.1 (3.7,53.9) |
| Peru | T2D | -2.6 (-4.0,-1.4) | -1.9 (-3.1,-1.0) | -1.7 (-2.7,-0.9) | 253 (171,383) | 568 (378,898) | 9.6 (6.0,16.2) |
| | CVD | -0.2 (-0.9,0.6) | -1.8 (-2.7,-1.1) | -1.3 (-2.0,-0.8) | 50.3 (15.8,113) | -736 (-1094,-478) | -19.1 (-29.3,-12.0) |
| Philippines | T2D | -1.0 (-1.7,-0.4) | -1.5 (-2.1,-1.1) | -0.8 (-1.1,-0.6) | 67.2 (46.6,106) | 214 (158,349) | 6.2 (5.0,9.3) |
| | CVD | -0.5 (-0.8,-0.2) | -1.2 (-1.7,-0.9) | -0.6 (-0.8,-0.4) | 8.6 (0.1,22.4) | -196 (-349,-40.9) | 0.3 (-2.3,5.1) |
| Poland | T2D | -0.1 (-0.8,0.3) | -0.2 (-0.5,-0.1) | -0.4 (-0.8,-0.3) | 56.0 (41.8,95.8) | 98.9 (76.1,170) | 0.6 (0.1,1.1) |

Supplementary Data 2. Differences in proportional and absolute T2D and CVD burdens attributable to SSBs from 1990 to 2020 globally, regionally, and nationally (continued).

| | | Difference in proportional burden (1990-2020) % § | | | Difference in absolute burden per 1M adults (1990-2020) §, ƒ | | |
|---|---|---|---|---|---|---|---|
| Location | Disease | Incidence | DALYs | Deaths | Incidence | DALYs | Deaths |
| | CVD | -0.2 (-0.3,-0.1) | -0.5 (-1.0,-0.4) | -0.4 (-0.6,-0.3) | -51.1 (-82.8,-38.6) | -1365 (-2225,-1164) | -41.2 (-62.6,-34.8) |
| Portugal | T2D | 0.2 (-0.4,1.1) | 0.0 (-0.5,0.4) | -1.0 (-1.7,-0.8) | 250 (208,350) | 240 (184,357) | -3.2 (-6.4,-2.6) |
| | CVD | -0.1 (-0.3,0.2) | -0.1 (-0.2,0.2) | -0.2 (-0.3,-0.1) | -61.6 (-90.6,-50.0) | -1122 (-1512,-976) | -43.7 (-61.2,-37.6) |
| Qatar | T2D | 2.3 (0.9,5.0) | 3.1 (1.8,5.3) | 1.5 (0.7,3.4) | 1196 (770,1877) | 972 (620,1588) | -0.5 (-3.4,2.5) |
| | CVD | 1.5 (0.5,3.9) | 1.5 (0.6,3.6) | 1.3 (0.6,2.9) | 133 (28.1,314) | -1761 (-2750,-1120) | -51.1 (-81.8,-32.8) |
| Romania | T2D | 0.2 (-0.5,1.1) | -0.5 (-1.2,0.0) | -0.9 (-1.7,-0.5) | 129 (94.2,183) | 231 (167,321) | 1.3 (0.6,2.5) |
| | CVD | -0.3 (-0.7,-0.1) | -0.6 (-0.9,-0.4) | -0.5 (-0.8,-0.3) | 4.7 (-35.6,37.7) | -988 (-1482,-739) | -18.6 (-35.7,-10.1) |
| Russian Federation | T2D | 3.5 (2.6,5.3) | 1.7 (1.3,2.8) | 1.0 (0.7,1.7) | 189 (145,276) | 421 (330,636) | 7.8 (6.1,11.8) |
| | CVD | 1.3 (1.0,2.0) | 1.2 (0.9,1.8) | 0.8 (0.6,1.2) | 274 (213,414) | 1137 (824,1874) | 49.7 (37.4,78.0) |
| Rwanda | T2D | 62.4 (56.7,67.5) | 57.8 (51.4,63.3) | 51.0 (43.4,57.2) | 1006 (910,1095) | 5626 (4890,6374) | 155 (130,182) |
| | CVD | 44.3 (37.5,51.0) | 41.0 (34.3,47.4) | 34.5 (27.4,41.6) | 1512 (1240,1805) | 7105 (5655,8556) | 249 (193,315) |
| Samoa | T2D | 17.0 (12.0,23.1) | 9.1 (6.2,13.3) | 5.8 (3.8,8.8) | 2129 (1489,2980) | 5265 (3557,7915) | 78.9 (51.2,121) |
| | CVD | 4.5 (3.0,6.9) | 6.2 (4.1,9.3) | 4.3 (2.8,6.8) | 312 (197,483) | 5410 (3564,8268) | 152 (98.6,237) |
| Sao Tome and Principe | T2D | 7.9 (4.3,12.9) | 6.6 (3.6,10.6) | 3.9 (1.9,7.5) | 417 (265,623) | 894 (554,1369) | 6.8 (3.6,12.8) |
| | CVD | 3.9 (1.9,7.0) | 3.5 (1.7,6.2) | 2.5 (1.3,4.7) | 165 (57.6,333) | 1143 (523,2075) | 32.6 (15.0,65.2) |
| Saudi Arabia | T2D | 9.0 (4.7,15.4) | 8.8 (5.1,14.0) | 7.3 (4.1,12.0) | 1446 (967,2146) | 2026 (1344,3055) | 18.1 (11.2,28.4) |
| | CVD | 6.2 (3.4,10.6) | 6.7 (3.7,11.1) | 6.1 (3.6,10.0) | 677 (389,1109) | 3554 (2000,6008) | 76.3 (37.6,136) |
| Senegal | T2D | 16.7 (11.0,22.0) | 13.2 (8.1,18.2) | 9.8 (5.0,15.1) | 1305 (977,1612) | 3434 (2497,4477) | 55.8 (36.9,80.4) |
| | CVD | 10.0 (5.8,14.6) | 7.9 (4.1,12.1) | 6.1 (2.9,10.2) | 356 (162,587) | 1881 (513,3467) | 76.5 (21.0,139) |
| Serbia | T2D | -1.5 (-3.0,-0.6) | -1.1 (-2.5,-0.5) | -0.9 (-2.0,-0.4) | -0.5 (-24.5,45.9) | 13.5 (-45.8,88.5) | 0.2 (-1.1,1.3) |
| | CVD | -0.9 (-1.9,-0.4) | -0.8 (-1.6,-0.4) | -0.5 (-1.1,-0.2) | -58.9 (-126,-24.4) | -948 (-1957,-439) | -27.3 (-57.6,-12.5) |
| Seychelles | T2D | 2.4 (1.1,4.3) | 2.4 (1.3,3.8) | 1.6 (0.4,2.9) | 1876 (1472,2417) | 3201 (2525,4156) | 21.6 (15.6,30.1) |
| | CVD | 2.0 (1.2,3.1) | 1.8 (1.1,2.9) | 1.4 (0.8,2.1) | 88.8 (38.5,166) | -1888 (-2763,-1229) | -75.1 (-113,-50.4) |
| Sierra Leone | T2D | 20.7 (14.9,27.3) | 16.3 (11.2,22.0) | 11.7 (7.7,17.0) | 770 (543,1025) | 2127 (1479,2909) | 33.1 (21.8,48.4) |
| | CVD | 9.7 (6.2,14.5) | 8.8 (5.8,13.1) | 6.7 (4.3,10.6) | 410 (254,623) | 3140 (1982,4813) | 100 (61.9,164) |
| Singapore | T2D | -0.9 (-2.7,0.2) | 0.4 (-0.1,1.4) | -0.5 (-1.2,-0.3) | 104 (48.7,204) | 132 (66.1,282) | -4.5 (-7.4,-3.6) |
| | CVD | 0.0 (-0.3,0.4) | -0.2 (-0.5,0.1) | -0.2 (-0.4,-0.1) | 9.0 (-2.5,34.6) | -857 (-1357,-669) | -24.0 (-38.8,-19.0) |
| Slovak Republic | T2D | 2.4 (1.1,3.9) | 1.4 (0.7,2.4) | 0.7 (0.2,1.5) | 184 (134,260) | 280 (198,418) | 1.1 (0.0,2.6) |
| | CVD | 0.6 (0.2,1.2) | 0.1 (-0.3,0.6) | 0.1 (-0.2,0.5) | 28.5 (-16.7,80.1) | -1324 (-2276,-890) | -32.5 (-62.3,-16.3) |
| Slovenia | T2D | 2.2 (1.4,3.4) | 1.0 (0.5,1.8) | 0.2 (-0.3,0.7) | 145 (105,209) | 277 (200,429) | 2.6 (1.6,4.5) |
| | CVD | 0.7 (0.4,1.3) | 0.0 (-0.4,0.3) | 0.0 (-0.3,0.2) | 79.5 (47.5,139) | -578 (-1007,-413) | -16.6 (-30.7,-11.0) |
| Solomon Islands | T2D | -2.9 (-4.7,-1.3) | -1.2 (-2.7,-0.2) | -1.1 (-2.4,-0.2) | 276 (148,475) | 619 (207,1363) | 5.7 (-2.7,19.8) |
| | CVD | -1.0 (-2.1,-0.4) | -1.0 (-2.3,-0.1) | -0.9 (-2.1,-0.3) | -31.9 (-80.3,2.6) | -977 (-2218,-235) | -28.4 (-63.5,-10.4) |
| South Africa | T2D | -4.2 (-5.9,-2.1) | -5.4 (-6.7,-4.1) | -4.1 (-5.0,-3.0) | 476 (347,666) | 1810 (1243,2642) | 50.6 (34.2,74.8) |
| | CVD | -2.5 (-3.6,-1.3) | -5.4 (-6.9,-4.2) | -3.6 (-4.5,-2.8) | -202 (-304,-111) | -1432 (-1845,-1045) | -23.0 (-32.2,-13.2) |
| Spain | T2D | 2.1 (1.5,3.2) | 1.5 (1.1,2.2) | -0.3 (-0.7,-0.2) | 251 (196,348) | 298 (227,424) | -3.2 (-5.2,-2.5) |

Supplementary Data 2. Differences in proportional and absolute T2D and CVD burdens attributable to SSBs from 1990 to 2020 globally, regionally, and nationally (continued).

| | | Difference in proportional burden (1990-2020) % § | | | Difference in absolute burden per 1M adults (1990-2020) §, f | | |
|---|---|---|---|---|---|---|---|
| Location | Disease | Incidence | DALYs | Deaths | Incidence | DALYs | Deaths |
| | CVD | 0.9 (0.6,1.6) | 0.3 (0.1,0.6) | 0.1 (0.0,0.2) | 4.1 (-14.9,27.7) | -609 (-886,-487) | -22.6 (-33.0,-18.0) |
| Sri Lanka | T2D | 9.2 (6.9,12.2) | 7.1 (4.9,10.2) | 5.7 (3.7,8.5) | 1615 (1204,2119) | 3951 (2824,5467) | 66.6 (43.5,98.1) |
| | CVD | 4.7 (3.2,6.9) | 4.6 (3.2,6.6) | 3.6 (2.3,5.4) | 361 (247,523) | 2477 (1715,3605) | 97.2 (62.3,148) |
| St. Lucia | T2D | 2.7 (-2.2,7.9) | 2.3 (-1.2,6.0) | 0.5 (-2.5,3.1) | 1244 (829,1822) | 1784 (489,3279) | -16.4 (-50.2,8.1) |
| | CVD | 1.0 (-1.7,3.6) | 0.1 (-2.2,2.1) | -0.2 (-2.0,1.2) | 300 (105,546) | -2043 (-3259,-1198) | -77.8 (-130,-43.5) |
| St. Vincent and the Grenadines | T2D | 36.1 (30.4,41.1) | 31.4 (26.2,36.2) | 25.5 (21.1,30.4) | 4736 (4148,5334) | 16666 (13956,19141) | 306 (250,363) |
| | CVD | 22.9 (18.8,27.2) | 19.2 (15.6,23.0) | 14.9 (11.8,18.5) | 2016 (1653,2424) | 7013 (5436,8626) | 316 (245,401) |
| Sudan | T2D | 3.3 (1.9,5.1) | 2.9 (1.6,4.5) | 1.0 (0.0,2.2) | 733 (473,1074) | 849 (536,1259) | 4.4 (2.3,7.9) |
| | CVD | 1.3 (0.4,2.6) | 0.8 (-0.3,1.9) | 0.2 (-0.7,0.9) | -115 (-325,16.7) | -3828 (-6437,-2082) | -121 (-205,-70.0) |
| Suriname | T2D | 13.3 (9.7,16.9) | 9.8 (6.8,13.3) | 7.5 (5.1,10.5) | 2381 (1697,3191) | 6521 (4486,9137) | 89.5 (60.9,129) |
| | CVD | 6.3 (4.1,8.8) | 5.7 (3.8,8.1) | 4.3 (2.9,6.2) | 707 (472,1024) | 2340 (1498,3393) | 87.0 (56.3,127) |
| Swaziland | T2D | 6.0 (3.9,8.3) | 4.8 (3.2,6.8) | 4.3 (2.7,6.2) | 867 (615,1193) | 4219 (2852,6045) | 106 (68.8,158) |
| | CVD | 3.0 (1.6,4.9) | 4.1 (2.5,6.4) | 3.4 (2.0,5.1) | 194 (113,296) | 2361 (1494,3586) | 69.0 (41.7,108) |
| Sweden | T2D | -0.8 (-1.7,0.1) | -0.6 (-1.0,-0.3) | -0.7 (-1.0,-0.5) | 78.6 (52.6,141) | 60.9 (28.9,126) | -1.1 (-1.7,-0.7) |
| | CVD | -0.5 (-0.7,-0.3) | -0.7 (-1.0,-0.5) | -0.5 (-0.8,-0.5) | -71.8 (-102,-56.5) | -1578 (-2191,-1347) | -69.6 (-96.2,-59.8) |
| Switzerland | T2D | 0.5 (-0.2,1.4) | 1.0 (0.6,1.5) | -0.4 (-0.7,-0.3) | 257 (203,351) | 252 (192,359) | -4.1 (-6.0,-3.4) |
| | CVD | -0.1 (-0.4,0.1) | -0.7 (-1.1,-0.5) | -0.5 (-0.8,-0.4) | -53.6 (-87.5,-38.0) | -1382 (-1943,-1090) | -52.8 (-74.3,-42.8) |
| Syrian Arab Republic | T2D | 1.7 (0.5,3.5) | 1.7 (1.0,3.0) | 1.4 (0.8,2.8) | 372 (259,549) | 587 (420,895) | 4.6 (3.0,8.1) |
| | CVD | 1.5 (0.9,2.7) | 0.7 (0.0,1.7) | 0.9 (0.5,1.9) | 313 (209,547) | 168 (-573,1098) | 32.8 (13.7,72.5) |
| Taiwan | T2D | -4.7 (-6.0,-3.4) | -2.0 (-2.8,-1.5) | -2.1 (-3.2,-1.6) | 197 (141,296) | 210 (123,397) | -3.1 (-6.0,-1.1) |
| | CVD | -1.6 (-2.2,-1.1) | -1.0 (-1.4,-0.5) | -0.9 (-1.2,-0.6) | 26.3 (-4.2,76.3) | -459 (-600,-343) | -13.7 (-18.7,-10.5) |
| Tajikistan | T2D | 9.5 (6.2,14.2) | 6.3 (4.2,9.7) | 4.7 (3.0,7.9) | 365 (246,534) | 601 (407,940) | 7.0 (4.5,12.2) |
| | CVD | 3.3 (2.2,5.2) | 3.4 (2.3,5.3) | 2.5 (1.7,4.0) | 285 (194,459) | 1538 (994,2503) | 49.5 (31.8,85.8) |
| Tanzania | T2D | 19.9 (13.8,26.8) | 16.4 (10.9,23.1) | 12.3 (8.0,18.0) | 349 (244,473) | 1585 (1054,2243) | 36.1 (23.3,53.8) |
| | CVD | 10.5 (6.9,15.2) | 10.5 (6.7,15.3) | 7.8 (4.9,12.0) | 401 (260,598) | 2836 (1817,4186) | 87.4 (54.5,137) |
| Thailand | T2D | 8.1 (3.9,15.2) | 6.0 (3.0,11.7) | 4.4 (2.1,8.8) | 512 (249,982) | 1282 (625,2529) | 21.3 (10.1,43.0) |
| | CVD | 3.4 (1.6,6.9) | 3.9 (1.9,7.6) | 2.5 (1.2,5.1) | 166 (79.6,340) | 1221 (606,2455) | 37.7 (18.2,79.1) |
| Timor-Leste | T2D | 29.3 (23.4,36.4) | 21.4 (16.3,27.9) | 16.4 (12.0,22.2) | 1393 (1113,1746) | 2778 (2130,3659) | 42.4 (31.1,58.5) |
| | CVD | 12.9 (9.7,17.5) | 15.3 (11.6,20.5) | 11.0 (8.2,15.1) | 489 (365,674) | 8025 (6104,10929) | 255 (190,353) |
| Togo | T2D | 59.1 (53.5,63.7) | 53.5 (46.7,58.6) | 47.0 (39.0,53.0) | 1423 (1267,1543) | 5697 (4918,6334) | 126 (104,145) |
| | CVD | 39.7 (32.4,46.0) | 36.2 (29.6,42.2) | 31.0 (24.0,37.3) | 1692 (1374,1994) | 11854 (9444,14164) | 405 (311,493) |
| Tonga | T2D | 14.5 (10.0,21.1) | 7.3 (4.7,11.0) | 4.5 (2.9,7.3) | 1536 (1043,2261) | 4217 (2694,6437) | 77.9 (49.4,127) |
| | CVD | 3.7 (2.4,5.9) | 4.9 (3.2,7.8) | 3.2 (2.0,5.2) | 259 (164,420) | 2650 (1685,4275) | 79.2 (50.2,128) |
| Trinidad and Tobago | T2D | 6.5 (4.4,8.7) | 4.1 (2.6,5.7) | 2.3 (1.1,3.7) | 1903 (1380,2548) | 5593 (3842,7693) | 88.3 (57.9,130) |
| | CVD | 3.1 (1.8,4.7) | 2.5 (1.6,3.7) | 1.7 (1.0,2.6) | 563 (358,840) | -575 (-1429,-45.1) | -7.6 (-34.4,11.1) |
| Tunisia | T2D | 8.5 (4.8,13.0) | 6.7 (3.9,10.4) | 4.7 (2.4,8.3) | 1305 (928,1827) | 2261 (1573,3264) | 21.5 (14.5,33.8) |

Supplementary Data 2. Differences in proportional and absolute T2D and CVD burdens attributable to SSBs from 1990 to 2020 globally, regionally, and nationally (continued).

| | | Difference in proportional burden (1990-2020) % § | | | Difference in absolute burden per 1M adults (1990-2020) §, ƒ | | |
|---|---|---|---|---|---|---|---|
| Location | Disease | Incidence | DALYs | Deaths | Incidence | DALYs | Deaths |
| | CVD | 4.5 (1.9,7.9) | 3.6 (1.7,6.6) | 2.6 (1.1,5.2) | 918 (549,1497) | 2429 (1211,4230) | 108 (59.2,200) |
| Turkey | T2D | -8.5 (-11.7,-5.9) | -6.2 (-8.7,-4.3) | -4.9 (-6.9,-3.4) | -156 (-234,-59.7) | -641 (-914,-405) | -17.7 (-25.8,-11.9) |
| | CVD | -4.9 (-7.0,-3.4) | -4.7 (-6.7,-3.3) | -3.6 (-5.2,-2.6) | -541 (-789,-378) | -3188 (-4534,-2246) | -105 (-149,-74.1) |
| Turkmenistan | T2D | -42.0 (-50.6,-34.0) | -28.9 (-37.7,-22.4) | -23.8 (-32.4,-17.7) | -416 (-501,-328) | -1127 (-1448,-865) | -22.1 (-29.9,-16.2) |
| | CVD | -17.8 (-23.8,-13.4) | -19.6 (-26.0,-15.0) | -15.1 (-20.5,-11.2) | -1428 (-1902,-1050) | -18579 (-24613,-14204) | -627 (-855,-464) |
| Uganda | T2D | 29.9 (21.6,38.3) | 24.5 (17.3,32.9) | 18.6 (12.5,26.3) | 483 (349,624) | 2268 (1591,3102) | 52.6 (35.1,76.2) |
| | CVD | 15.2 (10.2,21.6) | 15.3 (10.3,21.9) | 11.5 (7.4,16.9) | 424 (283,609) | 2387 (1593,3446) | 70.6 (46.2,106) |
| Ukraine | T2D | -2.1 (-3.5,-1.2) | -1.5 (-2.6,-1.0) | -1.3 (-2.2,-0.9) | 22.6 (5.8,62.3) | 8.6 (-24.7,55.4) | -0.9 (-1.5,-0.6) |
| | CVD | -0.7 (-1.2,-0.5) | -0.7 (-1.1,-0.5) | -0.5 (-0.8,-0.4) | -53.8 (-110,-18.5) | -783 (-1307,-527) | -26.5 (-46.9,-17.4) |
| United Arab Emirates | T2D | -9.8 (-14.4,-6.3) | -6.6 (-10.3,-3.9) | -5.3 (-8.5,-3.1) | 79.3 (-30.7,445) | -142 (-292,109) | -7.7 (-12.4,-4.7) |
| | CVD | -5.5 (-8.8,-3.3) | -5.4 (-8.4,-3.4) | -4.2 (-6.6,-2.6) | -207 (-374,-62.9) | -2358 (-3628,-1524) | -64.3 (-99.2,-41.4) |
| United Kingdom | T2D | 0.5 (-0.5,2.1) | 1.5 (0.8,2.6) | -1.1 (-1.8,-0.9) | 326 (266,435) | 433 (352,603) | -5.0 (-7.0,-4.3) |
| | CVD | -0.2 (-0.6,0.4) | -0.7 (-1.1,-0.5) | -0.8 (-1.2,-0.7) | -172 (-245,-133) | -3671 (-5004,-3157) | -149 (-198,-129) |
| United States | T2D | -3.3 (-5.1,-2.1) | -0.6 (-1.1,0.1) | -0.4 (-0.9,0.4) | 671 (576,985) | 843 (726,1290) | 0.0 (-1.0,2.7) |
| | CVD | -1.3 (-2.2,-1.0) | -0.6 (-1.0,-0.4) | -0.6 (-0.9,-0.4) | -382 (-535,-322) | -2030 (-2642,-1798) | -79.8 (-104,-71.1) |
| Uruguay | T2D | 5.2 (3.2,7.4) | 3.0 (1.7,4.7) | 0.7 (-0.4,1.6) | 891 (627,1223) | 1256 (836,1763) | 6.9 (3.1,11.5) |
| | CVD | 1.7 (0.8,3.1) | 0.6 (-0.1,1.4) | 0.0 (-0.8,0.5) | -33.7 (-140,58.7) | -4158 (-6492,-2760) | -161 (-261,-106) |
| Uzbekistan | T2D | -6.7 (-10.8,-3.6) | -3.9 (-7.0,-1.9) | -3.1 (-5.8,-1.4) | 204 (103,389) | 381 (184,786) | 5.4 (2.0,12.4) |
| | CVD | -2.3 (-3.9,-1.3) | -2.3 (-4.0,-1.2) | -1.4 (-2.7,-0.7) | -2.2 (-102,125) | -1993 (-3439,-1054) | -64.9 (-116,-33.6) |
| Vanuatu | T2D | 3.4 (2.0,5.2) | 1.7 (1.0,2.8) | 1.1 (0.6,2.0) | 438 (257,689) | 989 (589,1602) | 14.1 (8.5,25.6) |
| | CVD | 0.8 (0.4,1.3) | 1.2 (0.7,2.2) | 0.9 (0.5,1.5) | 58.5 (34.9,98.5) | 1345 (763,2392) | 33.3 (19.6,59.1) |
| Venezuela | T2D | -12.3 (-17.6,-7.1) | -9.2 (-13.5,-5.4) | -7.4 (-11.3,-4.2) | 190 (-1.4,453) | 602 (-17.7,1564) | 8.5 (-4.4,27.7) |
| | CVD | -6.4 (-9.7,-3.4) | -7.1 (-10.5,-4.0) | -5.3 (-8.3,-3.0) | -113 (-254,75.5) | -1707 (-3062,-249) | -42.8 (-91.1,6.3) |
| Vietnam | T2D | 1.0 (0.2,2.1) | 0.9 (0.5,1.7) | 0.6 (0.3,1.2) | 108 (77.6,173) | 239 (172,404) | 4.3 (2.9,7.6) |
| | CVD | 0.6 (0.3,1.2) | 0.5 (0.2,1.0) | 0.3 (0.2,0.6) | 40.7 (27.7,74.1) | 292 (189,546) | 10.1 (6.8,17.4) |
| Yemen, Rep. | T2D | 9.5 (6.1,12.5) | 7.9 (4.7,11.2) | 4.9 (1.9,8.2) | 909 (576,1278) | 1081 (662,1621) | 5.2 (1.1,11.0) |
| | CVD | 5.2 (2.6,8.2) | 4.3 (2.0,7.0) | 2.9 (1.0,5.0) | 80.3 (-357,364) | -3117 (-7679,-736) | -86.9 (-235,-16.6) |
| Zambia | T2D | -20.5 (-25.9,-15.4) | -20.4 (-25.8,-14.9) | -18.0 (-23.8,-12.8) | -152 (-246,-46.2) | -2028 (-2661,-1444) | -64.4 (-86.6,-45.1) |
| | CVD | -13.1 (-18.3,-9.1) | -11.9 (-17.0,-8.3) | -9.7 (-14.1,-6.3) | -457 (-648,-307) | -1690 (-2530,-1012) | -55.8 (-86.6,-33.1) |
| Zimbabwe | T2D | 16.7 (11.9,22.0) | 13.0 (8.8,18.3) | 11.1 (7.2,16.1) | 711 (508,948) | 3317 (2256,4679) | 82.0 (54.8,120) |
| | CVD | 9.0 (5.9,12.8) | 9.0 (6.1,12.7) | 7.1 (4.6,10.5) | 455 (300,655) | 3640 (2493,5325) | 116 (75.4,173) |

§ Data represent the central estimate (median) and 95% UI derived from the 2.5th and 97.5th percentiles of 1,000 multiway probabilistic Monte Carlo model simulations. The values shown are the direct and BMI mediated burdens combined using proportional multiplication.

ƒ The absolute burden per 1 million adults was calculated by dividing the absolute number of cases by the global/region/country adult population (20+ years) in that year and multiplying by 1 million.

† In prior GDD reports, the region Central/ Eastern Europe and Central Asia was referred as Former Soviet Union, and Southeast and East Asia was referred as Asia.

CVD, cardiovascular disease; DALYs, disability-adjusted life years; T2D, type 2 diabetes; UIs, uncertainty intervals.

Supplementary Data 2. Differences in proportional and absolute T2D and CVD burdens attributable to SSBs from 1990 to 2020 globally, regionally, and nationally (continued).

| | | Difference in proportional burden (1990-2020) % § | | | Difference in absolute burden per 1M adults (1990-2020) §, ƒ | | |
|---|---|---|---|---|---|---|---|
| Location | Disease | Incidence | DALYs | Deaths | Incidence | DALYs | Deaths |
