# Supplementary material for: Burdens of type 2 diabetes and cardiovascular disease attributable to sugar-sweetened beverages in 184 countries
Source: Nat Med. 2025 Jan 6;31(2):552–64. doi: 10.1038/s41591-024-03345-4 (PMC11835746; doi:10.1038/s41591-024-03345-4)
Supplement: Supplementary file 5 — Sociodemographic development index and proportional T2D and CVD burdens attributable to SSBs in 1990 and 2020. [file 41591_2024_3345_MOESM5_ESM.pdf]

Burdens of type 2 diabetes and cardiovascular disease burdens to sugar-sweetened beverages in 184 countries

Supplementary Data 3 | Sociodemographic development index and proportional T2D and CVD burdens attributable to SSBs in 1990 and 2020

Supplementary Data 3. Sociodemographic development index (SDI) and proportional T2D and CVD burdens attributable to SSBs in 1990 and 2020 (continued).

World Region	Country	Year	SDI*	T2D incidence	CVD incidence	T2D deaths	CVD deaths	T2D DALYs	CVD DALYs
Centr/Eastern Eur Centr Asia	Albania	1990	0.5577733	4.5 (3.0-7.3)	2.0 (1.3-3.3)	1.7 (1.1-3.0)	1.2 (0.8-2.0)	2.7 (1.8-4.6)	1.7 (1.1-2.8)
		2020	0.70379025	51.9 (42.6-60.6)	26.5 (19.7-33.9)	27.8 (20.3-36.7)	18.1 (13.1-24.4)	39.1 (30.2-48.1)	23.2 (17.1-29.9)
	Armenia	1990	0.54441453	1.8 (1.2-3.7)	0.7 (0.4-1.2)	0.9 (0.6-1.5)	0.5 (0.3-0.9)	1.1 (0.7-2.0)	0.7 (0.5-1.4)
		2020	0.6982689	5.5 (3.4-8.9)	1.9 (1.2-3.4)	2.2 (1.4-4.1)	1.3 (0.8-2.3)	3.2 (2.1-5.6)	1.8 (1.2-3.2)
	Azerbaijan	1990	0.59598603	3.3 (2.2-5.6)	1.1 (0.8-2.0)	1.4 (1.0-2.7)	0.9 (0.6-1.7)	1.9 (1.3-3.3)	1.3 (0.9-2.4)
		2020	0.69260519	4.9 (3.3-7.9)	1.7 (1.2-2.8)	2.2 (1.5-3.9)	1.3 (0.9-2.2)	3.0 (2.0-5.1)	1.8 (1.2-3.0)
	Belarus	1990	0.62244658	7.3 (4.9-11.0)	2.3 (1.6-3.7)	3.2 (2.1-5.4)	1.8 (1.2-2.8)	4.3 (2.9-6.6)	2.5 (1.7-4.0)
		2020	0.78238156	4.1 (2.8-6.7)	1.3 (0.9-2.1)	1.6 (1.1-2.9)	1.0 (0.7-1.6)	2.5 (1.7-4.3)	1.3 (0.9-2.2)
	Bosnia and Herzegovina	1990	0.54113254	1.2 (0.8-2.4)	0.6 (0.4-0.9)	0.6 (0.4-1.1)	0.4 (0.3-0.7)	0.8 (0.5-1.6)	0.5 (0.3-0.9)
		2020	0.72020206	5.5 (3.8-8.9)	2.1 (1.5-3.4)	2.3 (1.6-3.8)	1.3 (0.9-2.1)	3.2 (2.2-5.2)	1.8 (1.2-2.8)
	Bulgaria	1990	0.6334465	3.8 (2.7-6.1)	1.4 (1.1-2.2)	1.9 (1.4-3.2)	1.2 (0.9-1.8)	2.4 (1.8-4.1)	1.5 (1.1-2.4)
		2020	0.76508971	4.2 (3.0-7.0)	1.3 (1.0-2.1)	1.7 (1.3-2.7)	1.0 (0.7-1.4)	2.4 (1.8-4.2)	1.3 (1.0-2.2)
	Croatia	1990	0.66890636	5.4 (4.0-7.9)	2.0 (1.5-3.2)	2.5 (1.9-4.0)	1.4 (1.1-2.2)	3.5 (2.6-5.4)	2.0 (1.5-3.1)
		2020	0.79546227	6.3 (4.7-9.3)	2.1 (1.6-3.0)	2.2 (1.7-3.2)	1.4 (1.0-2.0)	3.5 (2.7-5.2)	1.9 (1.4-2.7)
	Czech Republic	1990	0.68184802	5.0 (3.9-7.4)	1.9 (1.5-2.7)	2.2 (1.7-3.3)	1.4 (1.1-2.0)	3.0 (2.4-4.4)	1.9 (1.5-2.6)
		2020	0.82662663	3.8 (3.0-5.9)	1.4 (1.1-2.0)	1.3 (1.0-1.9)	0.8 (0.7-1.2)	2.1 (1.7-3.1)	1.2 (0.9-1.7)
	Estonia	1990	0.67496763	4.2 (3.2-6.6)	1.3 (1.1-2.2)	1.8 (1.4-3.0)	1.0 (0.8-1.4)	2.5 (2.0-4.4)	1.4 (1.1-2.3)
		2020	0.84181714	5.4 (4.2-7.9)	1.7 (1.3-2.6)	1.9 (1.5-3.0)	1.0 (0.8-1.4)	2.8 (2.3-4.5)	1.4 (1.1-2.1)
	Georgia	1990	0.65613604	0.3 (0.2-0.6)	0.1 (0.1-0.1)	0.1 (0.1-0.2)	0.1 (0.1-0.1)	0.2 (0.1-0.3)	0.1 (0.1-0.2)
		2020	0.72912589	6.7 (4.5-10.4)	2.0 (1.4-3.2)	2.5 (1.7-4.0)	1.3 (0.9-2.0)	3.6 (2.5-5.9)	1.8 (1.3-3.0)
	Hungary	1990	0.64941999	7.0 (5.5-10.0)	2.8 (2.2-3.9)	3.4 (2.6-4.9)	2.1 (1.6-2.9)	4.5 (3.5-6.5)	2.9 (2.3-4.0)
		2020	0.78762039	6.5 (5.1-9.4)	2.2 (1.7-3.1)	2.5 (1.9-3.6)	1.5 (1.2-2.1)	3.7 (2.9-5.3)	2.1 (1.6-2.9)
	Kazakhstan	1990	0.5894358	2.9 (1.9-5.1)	0.9 (0.6-1.6)	1.1 (0.8-2.0)	0.7 (0.5-1.2)	1.7 (1.1-2.9)	1.0 (0.7-1.8)
		2020	0.72278877	5.4 (3.5-8.6)	1.8 (1.2-2.9)	2.0 (1.4-3.3)	1.1 (0.7-1.7)	3.3 (2.2-5.2)	1.5 (1.0-2.4)
	Kyrgyz Republic	1990	0.51940765	11.8 (8.2-16.8)	3.7 (2.6-5.7)	5.1 (3.4-8.2)	2.8 (1.9-4.3)	7.1 (4.9-10.4)	4.0 (2.8-6.0)
		2020	0.60049955	16.1 (11.5-22.0)	5.2 (3.7-7.8)	6.9 (4.9-10.9)	3.7 (2.6-5.7)	9.9 (7.0-14.4)	5.1 (3.6-7.6)
	Latvia	1990	0.68019364	4.0 (3.1-6.0)	1.1 (0.9-1.7)	1.7 (1.4-2.8)	0.9 (0.7-1.4)	2.3 (1.8-3.9)	1.3 (1.0-2.2)
		2020	0.82790045	4.0 (3.1-5.9)	1.0 (0.8-1.5)	1.5 (1.1-2.2)	0.7 (0.6-1.0)	2.1 (1.7-3.3)	1.0 (0.8-1.5)

Supplementary Data 3. Sociodemographic development index (SDI) and proportional T2D and CVD burdens attributable to SSBs in 1990 and 2020 (continued).

World Region	Country	Year	SDI*	T2D incidence	CVD incidence	T2D deaths	CVD deaths	T2D DALYs	CVD DALYs
	Lithuania	1990	0.66850394	3.6 (2.8-5.8)	1.1 (0.8-1.6)	1.5 (1.2-2.7)	0.9 (0.7-1.3)	2.1 (1.6-3.6)	1.2 (0.9-2.0)
		2020	0.85275239	4.8 (3.7-6.9)	1.2 (0.9-1.8)	1.8 (1.4-2.9)	0.9 (0.7-1.4)	2.6 (2.0-4.2)	1.2 (1.0-2.0)
	Macedonia, FYR	1990	0.60902609	2.6 (1.9-4.7)	1.1 (0.8-1.7)	1.2 (0.9-2.0)	0.7 (0.5-1.1)	1.6 (1.2-2.9)	1.0 (0.7-1.6)
		2020	0.7478499	15.1 (11.5-20.0)	6.0 (4.4-8.2)	6.6 (4.8-9.5)	3.6 (2.6-5.0)	9.2 (6.8-12.6)	4.8 (3.5-6.5)
	Moldova	1990	0.60425176	5.2 (3.6-8.4)	1.7 (1.2-2.7)	2.4 (1.6-4.3)	1.3 (0.9-2.1)	3.2 (2.2-5.2)	1.8 (1.2-2.8)
		2020	0.72771054	3.4 (2.3-5.6)	1.0 (0.7-1.7)	1.4 (1.0-2.5)	0.8 (0.6-1.4)	2.0 (1.4-3.4)	1.1 (0.7-1.8)
	Mongolia	1990	0.46655012	14.1 (9.6-20.0)	4.5 (3.0-6.6)	5.4 (3.7-8.2)	3.4 (2.3-5.1)	8.1 (5.5-11.7)	4.8 (3.2-6.9)
		2020	0.61462964	13.9 (9.5-19.8)	4.9 (3.4-7.2)	6.1 (4.1-9.4)	3.6 (2.5-5.5)	8.7 (5.9-12.7)	5.1 (3.5-7.7)
	Montenegro	1990	0.67422572	3.5 (1.4-8.2)	1.6 (0.7-3.8)	1.5 (0.7-3.7)	1.1 (0.5-2.6)	2.1 (0.9-5.1)	1.6 (0.7-3.8)
		2020	0.79255439	5.4 (2.2-12.5)	2.2 (0.9-5.2)	2.1 (0.9-5.2)	1.4 (0.6-3.3)	3.1 (1.3-7.5)	1.9 (0.8-4.5)
	Poland	1990	0.62722789	4.1 (3.4-6.2)	1.6 (1.4-2.3)	1.9 (1.6-2.9)	1.2 (1.0-1.7)	2.5 (2.1-3.7)	1.7 (1.5-2.7)
		2020	0.80879532	3.9 (3.3-5.9)	1.5 (1.2-2.1)	1.5 (1.2-2.2)	0.8 (0.7-1.2)	2.2 (1.9-3.4)	1.2 (1.0-1.7)
	Romania	1990	0.61929886	11.0 (8.7-14.3)	3.9 (3.1-5.3)	5.6 (4.4-7.8)	2.9 (2.3-4.0)	7.1 (5.6-9.8)	4.0 (3.1-5.3)
		2020	0.76427644	11.3 (8.7-14.8)	3.6 (2.8-4.8)	4.7 (3.6-6.6)	2.4 (1.9-3.3)	6.7 (5.2-8.9)	3.4 (2.7-4.6)
	Russian Federation	1990	0.67160058	3.5 (2.7-5.8)	1.3 (1.0-1.9)	1.6 (1.2-2.5)	0.9 (0.7-1.3)	2.2 (1.7-3.5)	1.2 (1.0-1.9)
		2020	0.80601147	7.1 (5.5-10.2)	2.6 (2.0-3.7)	2.7 (2.1-3.9)	1.7 (1.3-2.5)	4.0 (3.1-5.7)	2.4 (1.9-3.5)
	Serbia	1990	0.63051102	4.4 (2.1-8.9)	1.9 (0.9-3.9)	2.1 (1.0-4.4)	1.1 (0.5-2.3)	2.8 (1.4-6.1)	1.6 (0.8-3.2)
		2020	0.78918333	2.9 (1.4-6.3)	1.0 (0.5-2.0)	1.2 (0.6-2.6)	0.6 (0.3-1.3)	1.7 (0.8-3.6)	0.8 (0.4-1.7)
	Slovak Republic	1990	0.65385351	7.7 (6.0-10.6)	2.7 (2.2-3.9)	3.4 (2.7-4.9)	2.2 (1.8-3.2)	4.6 (3.6-6.7)	3.0 (2.4-4.4)
		2020	0.80830597	10.1 (7.9-13.3)	3.4 (2.7-4.6)	4.1 (3.2-5.9)	2.3 (1.8-3.4)	6.0 (4.8-8.3)	3.2 (2.5-4.4)
	Slovenia	1990	0.72746393	5.8 (4.3-8.6)	2.4 (1.8-3.5)	2.6 (1.9-4.1)	1.5 (1.1-2.3)	3.6 (2.7-5.7)	2.1 (1.6-3.2)
		2020	0.84037385	8.1 (6.0-11.3)	3.1 (2.3-4.6)	2.9 (2.1-4.4)	1.5 (1.1-2.2)	4.7 (3.5-7.0)	2.2 (1.6-3.2)
	Tajikistan	1990	0.46615541	3.6 (2.2-6.5)	1.1 (0.7-1.9)	1.5 (1.0-2.8)	0.8 (0.5-1.4)	2.0 (1.3-3.7)	1.2 (0.8-2.0)
		2020	0.53769053	13.4 (9.0-19.4)	4.4 (3.1-6.9)	6.2 (4.1-10.1)	3.3 (2.3-5.1)	8.4 (5.6-12.8)	4.6 (3.2-7.1)
	Turkmenistan	1990	0.56312689	44.7 (35.8-54.1)	18.7 (14.0-24.9)	25.1 (18.6-34.1)	15.7 (11.8-21.4)	30.5 (23.6-40.1)	20.4 (15.6-27.3)
		2020	0.67829818	2.6 (1.6-4.8)	0.8 (0.5-1.4)	1.2 (0.7-2.2)	0.6 (0.4-1.0)	1.5 (0.9-2.7)	0.8 (0.5-1.4)
	Ukraine	1990	0.64746147	7.1 (5.0-10.1)	2.2 (1.6-3.1)	3.5 (2.6-5.3)	1.6 (1.2-2.3)	4.5 (3.3-6.7)	2.1 (1.6-3.0)
		2020	0.75992987	4.9 (3.5-7.3)	1.4 (1.1-2.0)	2.2 (1.6-3.2)	1.0 (0.8-1.5)	2.9 (2.1-4.4)	1.4 (1.0-2.0)
	Uzbekistan	1990	0.50024174	16.9 (11.8-24.3)	5.4 (3.8-8.4)	7.9 (5.3-12.6)	4.2 (2.9-6.7)	10.2 (6.9-15.4)	6.2 (4.3-9.3)
		2020	0.6591242	10.0 (6.5-15.7)	3.1 (2.1-5.1)	4.7 (3.1-8.3)	2.8 (1.8-4.7)	6.2 (4.0-10.4)	3.9 (2.6-6.2)

Supplementary Data 3. Sociodemographic development index (SDI) and proportional T2D and CVD burdens attributable to SSBs in 1990 and 2020 (continued).

World Region	Country	Year	SDI*	T2D incidence	CVD incidence	T2D deaths	CVD deaths	T2D DALYs	CVD DALYs
High-Income Countries	Australia	1990	0.72598252	13.9 (10.9-18.5)	5.5 (4.3-8.0)	5.1 (4.0-8.2)	3.6 (2.8-5.4)	7.8 (6.2-11.6)	5.1 (4.0-7.7)
		2020	0.84205131	9.9 (7.8-14.6)	3.7 (2.8-5.8)	3.0 (2.4-4.6)	1.9 (1.5-2.7)	5.5 (4.3-8.6)	3.1 (2.4-4.8)
	Austria	1990	0.74985369	8.2 (6.7-10.5)	2.8 (2.3-3.8)	2.9 (2.4-4.1)	2.0 (1.6-2.7)	4.4 (3.7-6.0)	2.8 (2.3-3.9)
		2020	0.85202039	8.7 (7.1-11.3)	2.6 (2.2-3.7)	2.2 (1.8-3.1)	1.5 (1.2-2.0)	4.4 (3.7-5.9)	2.2 (1.8-3.2)
	Belgium	1990	0.73739066	15.1 (12.7-19.1)	5.4 (4.6-8.0)	4.4 (3.8-6.3)	3.3 (2.8-4.9)	8.3 (7.1-11.1)	4.8 (4.1-7.1)
		2020	0.85134665	16.6 (14.0-20.8)	5.0 (4.2-7.1)	3.9 (3.4-5.5)	2.6 (2.2-3.6)	9.7 (8.2-12.9)	3.9 (3.3-5.6)
	Canada	1990	0.78197786	10.3 (8.7-13.3)	4.3 (3.7-5.8)	4.1 (3.6-5.9)	3.0 (2.6-4.1)	5.8 (5.0-8.1)	4.3 (3.7-5.9)
		2020	0.87199263	8.9 (7.3-12.4)	3.4 (2.8-4.8)	3.1 (2.7-4.6)	1.9 (1.6-2.7)	5.3 (4.4-7.6)	3.0 (2.5-4.3)
	Cyprus	1990	0.64823087	6.7 (3.4-13.3)	3.0 (1.5-6.3)	2.6 (1.3-5.4)	2.0 (1.0-4.1)	3.8 (1.9-7.8)	2.8 (1.4-5.8)
		2020	0.83305952	9.7 (5.0-18.4)	3.3 (1.7-6.8)	2.7 (1.4-5.7)	2.2 (1.1-4.5)	5.3 (2.7-10.5)	3.3 (1.7-6.8)
	Denmark	1990	0.80115466	5.3 (4.2-8.1)	1.3 (1.1-2.0)	1.8 (1.5-2.8)	1.1 (0.9-1.6)	2.9 (2.3-4.7)	1.6 (1.3-2.4)
		2020	0.8943683	6.7 (5.3-9.8)	2.0 (1.6-3.1)	1.8 (1.5-2.5)	1.1 (0.9-1.5)	3.5 (2.8-5.1)	1.7 (1.4-2.6)
	Finland	1990	0.75622151	7.1 (5.8-9.9)	2.5 (2.1-3.9)	1.9 (1.7-2.7)	1.6 (1.3-2.4)	4.1 (3.4-6.0)	2.2 (1.9-3.5)
		2020	0.85765555	6.5 (5.4-9.3)	1.6 (1.3-2.3)	1.7 (1.4-2.4)	1.0 (0.8-1.4)	3.7 (3.0-5.4)	1.5 (1.2-2.1)
	France	1990	0.73074747	6.8 (5.7-9.1)	2.5 (2.1-3.5)	1.9 (1.6-2.7)	1.3 (1.1-1.8)	3.5 (3.0-4.9)	2.0 (1.8-2.8)
		2020	0.83604944	9.1 (7.7-12.1)	2.6 (2.2-3.7)	2.0 (1.8-2.8)	1.3 (1.1-1.8)	4.7 (4.0-6.3)	2.2 (1.9-3.0)
	Germany	1990	0.81707767	8.4 (7.0-10.6)	3.0 (2.5-4.3)	2.7 (2.3-3.6)	2.0 (1.7-2.7)	4.5 (3.8-6.0)	2.9 (2.5-4.1)
		2020	0.90143861	9.9 (8.3-12.7)	2.9 (2.5-4.1)	2.6 (2.3-3.7)	1.8 (1.5-2.4)	5.4 (4.6-7.1)	2.6 (2.2-3.7)
	Greece	1990	0.67418647	7.1 (5.6-9.7)	2.0 (1.6-2.9)	2.1 (1.7-3.1)	1.4 (1.2-2.0)	4.2 (3.3-6.0)	2.2 (1.8-3.3)
		2020	0.78963222	8.7 (6.9-11.6)	1.9 (1.6-2.9)	2.2 (1.8-3.2)	1.4 (1.1-1.9)	4.8 (3.8-6.6)	2.3 (1.8-3.4)
	Iceland	1990	0.76421252	12.9 (10.5-16.5)	4.0 (3.2-5.9)	3.2 (2.7-4.8)	2.4 (2.0-3.6)	7.1 (5.9-9.4)	3.6 (3.0-5.2)
		2020	0.87432315	14.2 (11.3-18.2)	4.2 (3.5-6.5)	3.3 (2.7-4.7)	2.3 (1.9-3.4)	8.5 (7.0-11.6)	3.7 (3.0-5.4)
	Ireland	1990	0.71989182	13.3 (10.5-16.6)	4.0 (3.1-5.7)	4.0 (3.2-5.8)	3.1 (2.4-4.4)	7.0 (5.6-9.2)	4.2 (3.3-5.9)
		2020	0.87195882	12.3 (9.6-15.7)	3.9 (3.1-5.6)	2.9 (2.3-4.1)	2.3 (1.8-3.1)	7.3 (5.7-9.5)	3.5 (2.8-4.9)
	Italy	1990	0.70625522	5.1 (4.3-7.2)	1.8 (1.5-2.5)	1.7 (1.5-2.6)	1.1 (0.9-1.5)	2.6 (2.3-3.8)	1.6 (1.4-2.3)
		2020	0.80363568	5.3 (4.4-7.7)	1.9 (1.6-2.7)	1.3 (1.1-1.7)	0.8 (0.7-1.0)	2.7 (2.3-3.8)	1.2 (1.1-1.7)
	Luxembourg	1990	0.78105161	9.8 (7.3-13.7)	3.0 (2.2-4.5)	2.9 (2.2-4.3)	2.0 (1.4-2.8)	5.4 (4.1-7.8)	2.9 (2.1-4.2)
		2020	0.88249532	12.5 (9.4-17.4)	3.9 (2.9-6.0)	2.9 (2.1-4.1)	1.9 (1.4-2.6)	7.5 (5.6-10.5)	3.0 (2.2-4.4)
	Malta	1990	0.65650458	11.5 (8.6-16.1)	4.4 (3.3-6.6)	4.0 (3.0-6.1)	3.1 (2.3-4.9)	6.3 (4.7-9.2)	4.3 (3.2-6.5)
		2020	0.79837517	20.7 (15.8-27.1)	6.5 (4.8-9.4)	5.9 (4.5-8.6)	4.2 (3.1-5.9)	11.6 (8.9-15.9)	6.3 (4.7-8.9)
	Netherlands	1990	0.79461212	11.8 (10.2-14.7)	4.3 (3.7-5.8)	3.7 (3.3-5.2)	2.9 (2.6-4.0)	6.2 (5.5-8.4)	4.2 (3.7-5.7)

Supplementary Data 3. Sociodemographic development index (SDI) and proportional T2D and CVD burdens attributable to SSBs in 1990 and 2020 (continued).

World Region	Country	Year	SDI*	T2D incidence	CVD incidence	T2D deaths	CVD deaths	T2D DALYs	CVD DALYs
		2020	0.88655857	1.1 (0.5-3.3)	0.4 (0.2-1.0)	0.3 (0.1-0.7)	0.2 (0.1-0.4)	0.6 (0.3-1.8)	0.3 (0.1-0.7)
	New Zealand	1990	0.75232165	4.1 (3.1-6.8)	1.4 (1.1-2.2)	1.5 (1.2-2.5)	1.0 (0.8-1.4)	2.2 (1.8-3.7)	1.3 (1.0-2.1)
		2020	0.84739874	6.1 (4.7-9.1)	2.1 (1.7-3.3)	2.1 (1.7-3.3)	1.1 (0.9-1.6)	3.7 (2.9-5.7)	1.8 (1.4-2.7)
	Norway	1990	0.79588728	7.8 (6.2-10.6)	2.1 (1.8-3.1)	2.1 (1.8-3.2)	1.5 (1.3-2.3)	4.3 (3.6-6.2)	2.2 (1.8-3.4)
		2020	0.91452992	8.4 (6.8-11.7)	2.4 (2.0-4.0)	2.0 (1.7-3.0)	1.3 (1.1-1.8)	4.9 (4.0-7.4)	2.0 (1.6-3.1)
	Portugal	1990	0.59977776	8.7 (7.4-11.3)	2.3 (2.0-3.2)	3.0 (2.7-4.6)	1.6 (1.4-2.1)	4.8 (4.2-6.6)	2.3 (2.0-3.1)
		2020	0.74103738	8.9 (7.6-11.8)	2.3 (2.0-3.2)	2.1 (1.8-2.9)	1.4 (1.2-1.8)	4.8 (4.1-6.4)	2.3 (2.0-3.1)
	Spain	1990	0.63667317	7.9 (6.4-11.2)	2.6 (2.2-3.9)	2.5 (2.1-3.5)	1.7 (1.4-2.3)	4.4 (3.6-6.1)	2.5 (2.1-3.7)
		2020	0.76650603	10.1 (8.2-13.9)	3.5 (2.8-5.2)	2.2 (1.8-2.9)	1.7 (1.4-2.4)	5.9 (4.8-8.0)	2.8 (2.3-4.1)
	Sweden	1990	0.78553579	8.2 (6.9-11.0)	2.3 (1.9-3.1)	2.4 (2.0-3.2)	1.6 (1.4-2.2)	4.5 (3.8-6.2)	2.2 (1.9-3.0)
		2020	0.88506201	7.3 (6.1-10.3)	1.8 (1.5-2.6)	1.7 (1.4-2.2)	1.1 (0.9-1.4)	3.8 (3.3-5.4)	1.6 (1.3-2.2)
	Switzerland	1990	0.86276684	9.9 (7.9-13.0)	3.2 (2.5-4.5)	2.7 (2.2-3.6)	2.0 (1.6-2.7)	5.3 (4.3-7.1)	2.9 (2.3-4.1)
		2020	0.93202764	10.5 (8.5-13.7)	3.1 (2.5-4.4)	2.3 (1.9-3.1)	1.4 (1.2-1.9)	6.3 (5.1-8.3)	2.2 (1.8-3.1)
	United Kingdom	1990	0.74433413	15.7 (13.5-19.5)	4.7 (4.0-6.3)	4.8 (4.2-6.5)	3.5 (3.0-4.8)	8.7 (7.6-11.2)	4.8 (4.2-6.6)
		2020	0.85692029	16.2 (13.9-20.3)	4.5 (3.9-6.3)	3.7 (3.2-4.8)	2.7 (2.3-3.6)	10.2 (8.8-13.1)	4.1 (3.5-5.8)
	United States	1990	0.76364769	19.0 (16.6-24.5)	6.8 (6.0-9.2)	7.1 (6.4-9.5)	4.4 (3.9-6.0)	10.5 (9.3-14.0)	6.4 (5.7-8.8)
		2020	0.86079277	15.7 (13.7-20.8)	5.4 (4.8-7.4)	6.7 (6.0-9.9)	3.8 (3.4-5.3)	9.8 (8.8-13.8)	5.8 (5.1-8.4)
Latin Amer/Caribbean	Antigua and Barbuda	1990	0.61210459	27.3 (19.0-37.7)	11.9 (7.9-17.3)	12.5 (8.3-18.5)	7.6 (5.1-11.5)	17.1 (11.5-24.6)	10.7 (7.2-15.8)
		2020	0.74634533	30.9 (21.6-42.5)	15.6 (10.6-23.0)	15.2 (10.2-22.1)	8.8 (5.8-13.2)	21.5 (14.8-30.6)	12.2 (8.2-17.8)
	Argentina	1990	0.58739728	12.2 (9.6-16.2)	5.5 (4.3-7.9)	6.1 (4.9-8.9)	4.0 (3.2-5.8)	7.9 (6.3-11.1)	5.6 (4.4-7.9)
		2020	0.7212943	18.8 (15.0-24.2)	8.3 (6.6-11.5)	8.7 (7.0-12.3)	5.4 (4.3-7.5)	12.1 (9.7-16.2)	7.6 (6.0-10.5)
	Bahamas, The	1990	0.69350927	56.7 (42.8-69.8)	30.8 (21.6-41.8)	34.0 (23.3-46.5)	24.4 (16.6-33.8)	42.7 (30.4-55.7)	31.6 (22.0-42.6)
		2020	0.80294802	21.7 (14.1-31.4)	10.2 (6.4-15.7)	11.1 (7.0-17.0)	7.1 (4.4-10.9)	15.5 (9.8-23.2)	9.8 (6.0-14.9)
	Barbados	1990	0.65358252	32.5 (26.2-39.8)	15.2 (11.8-19.2)	16.2 (12.4-21.0)	9.2 (6.9-12.0)	21.4 (16.7-26.9)	12.6 (9.7-16.0)
		2020	0.74436665	41.3 (33.7-49.8)	20.7 (16.0-25.8)	20.4 (15.8-26.0)	11.8 (9.0-15.2)	28.7 (22.8-35.2)	16.0 (12.2-20.0)
	Belize	1990	0.42372699	21.1 (14.5-29.5)	9.8 (6.6-14.3)	10.3 (6.8-15.0)	6.6 (4.5-10.1)	14.1 (9.5-20.1)	9.4 (6.3-13.7)
		2020	0.60706005	30.4 (21.7-41.4)	14.9 (10.1-21.4)	15.7 (10.7-22.4)	9.3 (6.3-13.6)	21.4 (14.7-29.7)	13.1 (9.0-18.8)
	Bolivia	1990	0.42391796	35.2 (27.1-43.3)	19.1 (14.3-24.6)	21.4 (15.9-27.9)	14.0 (10.4-18.4)	26.0 (19.6-33.2)	18.9 (14.1-24.4)
		2020	0.59485444	29.1 (22.0-36.9)	15.2 (11.1-20.1)	15.7 (11.4-21.4)	9.7 (7.1-13.4)	20.3 (14.9-26.4)	13.2 (9.8-17.6)
	Brazil	1990	0.50007051	24.2 (20.5-28.8)	11.5 (9.7-14.0)	13.2 (11.3-16.5)	8.5 (7.2-10.5)	17.4 (14.9-21.1)	11.5 (9.7-14.2)

Supplementary Data 3. Sociodemographic development index (SDI) and proportional T2D and CVD burdens attributable to SSBs in 1990 and 2020 (continued).

World Region	Country	Year	SDI*	T2D incidence	CVD incidence	T2D deaths	CVD deaths	T2D DALYs	CVD DALYs
		2020	0.64920157	14.2 (11.8-18.6)	6.3 (5.3-8.8)	6.6 (5.6-9.1)	4.6 (3.9-6.3)	9.5 (8.0-12.6)	6.5 (5.4-8.8)
	Chile	1990	0.5864951	18.3 (13.9-24.5)	6.9 (5.1-9.5)	9.0 (6.7-12.6)	5.0 (3.8-6.9)	11.9 (8.9-16.3)	6.8 (5.0-9.1)
		2020	0.76921368	25.0 (19.1-32.7)	10.8 (8.1-14.7)	10.7 (8.1-14.4)	7.3 (5.5-9.8)	16.8 (12.7-22.0)	10.3 (7.8-13.9)
	Colombia	1990	0.48072005	43.7 (35.6-52.4)	21.3 (16.6-27.0)	24.1 (18.5-30.6)	16.1 (12.1-20.8)	32.6 (25.9-40.0)	21.3 (16.5-26.9)
		2020	0.65085535	48.1 (39.3-57.3)	23.0 (18.0-29.2)	24.7 (19.0-31.6)	15.5 (11.6-20.4)	35.6 (28.3-44.0)	21.2 (16.4-27.1)
	Costa Rica	1990	0.53412518	30.7 (21.7-40.3)	14.0 (9.5-19.8)	15.6 (10.5-22.1)	9.1 (6.1-13.2)	21.7 (15.2-29.4)	12.8 (8.6-18.1)
		2020	0.69619461	21.7 (14.6-29.7)	9.4 (6.2-13.7)	8.9 (5.7-13.3)	5.9 (3.9-9.0)	14.6 (9.7-20.8)	8.8 (5.8-12.9)
	Cuba	1990	0.55801907	37.8 (30.2-46.1)	17.8 (13.6-23.1)	20.2 (14.8-27.2)	13.1 (9.8-17.2)	27.1 (20.5-34.6)	17.4 (13.2-22.5)
		2020	0.66521075	27.6 (20.9-35.5)	12.8 (9.5-17.3)	13.5 (9.8-18.5)	8.4 (6.1-11.6)	20.2 (15.2-26.8)	11.3 (8.4-15.4)
	Dominica	1990	0.56360259	26.8 (18.8-36.9)	12.5 (8.4-18.0)	12.9 (8.5-18.5)	7.7 (5.1-11.2)	17.2 (11.6-24.4)	10.1 (6.6-14.7)
		2020	0.74441994	19.9 (13.4-27.9)	9.0 (5.9-13.2)	9.2 (6.0-13.6)	5.3 (3.4-7.8)	13.1 (8.6-19.0)	7.2 (4.6-10.6)
	Dominican Republic	1990	0.44265408	13.3 (9.9-18.3)	6.1 (4.5-8.8)	6.7 (5.0-9.5)	4.4 (3.3-6.3)	9.4 (7.1-12.9)	6.4 (4.8-9.2)
		2020	0.61563563	25.0 (19.1-33.0)	10.9 (8.1-15.0)	13.0 (9.6-17.7)	8.4 (6.1-11.5)	17.8 (13.5-23.9)	11.6 (8.4-15.9)
	Ecuador	1990	0.51843061	48.6 (40.5-56.4)	27.2 (21.9-33.3)	30.5 (24.3-38.0)	19.2 (15.2-24.1)	38.0 (30.7-45.7)	26.8 (21.8-32.5)
		2020	0.65671446	34.7 (27.1-43.3)	17.8 (13.3-23.3)	18.0 (13.6-24.1)	11.4 (8.5-15.4)	24.6 (18.6-32.0)	16.7 (12.7-22.0)
	El Salvador	1990	0.37305797	18.5 (13.0-26.2)	7.5 (5.2-11.3)	9.2 (6.3-13.7)	5.8 (4.0-8.6)	12.6 (8.7-18.4)	8.5 (5.9-12.5)
		2020	0.55891989	28.3 (20.3-38.6)	11.1 (7.6-16.4)	13.5 (9.2-19.6)	7.7 (5.3-11.6)	18.6 (12.9-26.3)	11.4 (8.0-16.7)
	Grenada	1990	0.43673442	35.7 (28.7-43.4)	15.7 (12.1-20.3)	17.5 (13.4-23.0)	11.3 (8.6-15.2)	23.9 (18.8-30.5)	15.4 (12.0-20.0)
		2020	0.66508635	25.8 (19.9-32.8)	12.7 (9.4-16.9)	13.4 (9.9-18.2)	8.5 (6.2-11.9)	17.9 (13.5-23.5)	11.0 (8.2-15.1)
	Guatemala	1990	0.31179246	10.9 (8.2-14.5)	4.7 (3.5-6.9)	5.7 (4.4-7.8)	3.7 (2.8-5.3)	7.9 (6.0-10.6)	5.4 (4.0-7.5)
		2020	0.53457141	26.8 (21.4-33.2)	11.3 (8.7-14.6)	13.8 (10.7-17.8)	7.6 (5.8-9.8)	18.2 (14.3-23.0)	11.5 (8.9-14.8)
	Guyana	1990	0.46043013	37.6 (27.6-50.2)	17.6 (12.2-24.7)	21.3 (14.7-29.8)	13.3 (9.1-19.2)	27.0 (19.2-37.1)	17.4 (12.0-24.6)
		2020	0.64228464	28.7 (20.4-40.1)	12.7 (8.6-18.6)	15.0 (10.1-21.7)	9.1 (6.1-13.8)	19.6 (13.5-28.1)	12.0 (8.0-17.6)
	Haiti	1990	0.31033463	11.4 (7.6-17.4)	5.3 (3.5-8.7)	6.1 (4.1-9.6)	3.9 (2.6-6.5)	7.7 (5.2-11.9)	5.0 (3.4-8.3)
		2020	0.44639061	14.6 (9.9-21.2)	6.8 (4.5-10.5)	7.1 (4.9-11.4)	4.7 (3.2-7.6)	9.7 (6.6-14.6)	6.2 (4.2-9.6)
	Honduras	1990	0.33204289	20.2 (16.1-25.6)	9.0 (6.8-12.1)	10.8 (8.3-14.4)	6.2 (4.7-8.7)	14.6 (11.3-18.9)	8.7 (6.5-11.6)
		2020	0.50866925	32.5 (26.3-39.7)	14.1 (10.9-18.4)	15.6 (12.0-20.3)	9.3 (6.9-12.3)	22.1 (17.4-27.8)	12.1 (9.3-15.8)
	Jamaica	1990	0.53478123	28.4 (22.7-35.1)	13.8 (10.8-17.5)	15.1 (11.5-19.6)	7.4 (5.7-9.7)	19.3 (15.0-24.5)	10.3 (8.0-13.3)
		2020	0.68056718	26.4 (20.7-33.0)	12.3 (9.5-15.8)	11.8 (8.9-15.5)	6.2 (4.7-8.2)	17.3 (13.4-22.2)	9.1 (7.0-11.8)
	Mexico	1990	0.50499608	32.3 (28.7-36.6)	15.0 (13.0-17.8)	16.5 (14.4-19.6)	9.6 (8.3-11.5)	22.0 (19.5-25.8)	13.9 (12.2-16.5)

Supplementary Data 3. Sociodemographic development index (SDI) and proportional T2D and CVD burdens attributable to SSBs in 1990 and 2020 (continued).

World Region	Country	Year	SDI*	T2D incidence	CVD incidence	T2D deaths	CVD deaths	T2D DALYs	CVD DALYs
		2020	0.66011906	30.0 (26.4-35.0)	13.5 (11.5-16.7)	14.9 (12.9-18.3)	9.2 (8.0-11.4)	20.2 (17.6-24.0)	13.4 (11.7-16.5)
	Nicaragua	1990	0.34603523	13.1 (9.4-18.0)	5.7 (4.1-8.4)	6.7 (4.8-9.8)	3.8 (2.7-5.7)	9.2 (6.7-13.0)	5.7 (4.1-8.0)
		2020	0.52029267	31.8 (25.0-40.1)	14.8 (11.0-19.5)	17.2 (12.8-22.8)	10.1 (7.5-13.6)	23.0 (17.5-29.4)	14.3 (10.7-18.9)
	Panama	1990	0.54604812	32.5 (25.6-40.4)	15.4 (11.8-20.1)	17.0 (13.1-22.5)	10.1 (7.7-13.8)	23.7 (18.6-30.3)	13.7 (10.7-18.1)
		2020	0.70378849	37.7 (30.4-46.4)	17.8 (13.6-23.2)	18.9 (14.5-25.0)	11.1 (8.4-14.8)	26.8 (20.8-34.3)	15.8 (12.2-20.7)
	Paraguay	1990	0.46952779	17.5 (13.0-23.2)	7.8 (5.5-10.8)	8.7 (6.2-12.1)	5.2 (3.6-7.3)	12.2 (9.0-16.7)	7.4 (5.3-10.2)
		2020	0.63105769	23.0 (16.9-31.1)	10.7 (7.5-15.0)	11.5 (8.0-16.2)	7.1 (5.0-10.3)	15.4 (11.1-21.5)	9.9 (7.0-14.2)
	Peru	1990	0.51041985	21.3 (15.1-30.4)	10.1 (7.1-15.1)	11.5 (8.0-17.3)	7.1 (4.9-10.6)	15.4 (10.8-22.6)	10.7 (7.5-15.6)
		2020	0.65867224	18.7 (13.0-27.2)	9.9 (6.9-15.0)	9.8 (6.7-15.1)	5.8 (4.0-8.9)	13.5 (9.4-20.1)	8.9 (6.2-13.2)
	St. Lucia	1990	0.49629657	27.2 (21.4-34.6)	12.8 (9.9-17.4)	13.8 (10.3-18.6)	7.3 (5.5-10.1)	18.3 (14.1-24.0)	10.2 (7.8-13.8)
		2020	0.66972049	29.6 (23.1-38.1)	13.8 (10.2-18.6)	14.3 (10.7-18.9)	7.1 (5.2-9.9)	20.7 (15.7-26.6)	10.4 (7.7-13.9)
	St. Vincent and the Grenadines	1990	0.47593019	25.2 (19.8-31.8)	12.0 (9.1-16.0)	12.7 (9.6-17.7)	8.0 (6.0-11.3)	17.1 (13.0-22.6)	11.0 (8.4-14.7)
		2020	0.6328609	61.5 (53.4-68.9)	35.0 (29.1-41.4)	38.4 (31.9-45.8)	23.0 (18.6-28.4)	48.4 (41.1-56.1)	30.3 (25.0-36.2)
	Suriname	1990	0.5020543	22.0 (14.9-31.6)	10.1 (6.7-15.0)	11.6 (7.7-17.5)	7.5 (4.9-11.2)	15.4 (10.3-22.7)	10.2 (6.7-15.1)
		2020	0.63068371	35.5 (25.0-47.9)	16.4 (11.1-23.7)	19.1 (13.0-28.0)	11.8 (7.9-17.0)	25.3 (17.5-35.7)	15.9 (10.8-22.8)
	Trinidad and Tobago	1990	0.62397015	27.9 (19.6-38.3)	13.6 (9.2-19.6)	15.6 (10.6-22.9)	9.8 (6.6-14.6)	19.3 (13.4-27.9)	13.1 (9.0-19.1)
		2020	0.76642208	34.4 (24.8-46.0)	16.7 (11.5-23.6)	18.0 (12.2-26.0)	11.6 (8.0-16.6)	23.5 (16.3-32.8)	15.7 (10.9-22.4)
	Uruguay	1990	0.58192186	26.1 (18.4-37.3)	10.5 (7.1-16.1)	13.3 (8.9-20.2)	8.1 (5.5-12.7)	17.1 (11.7-25.2)	10.8 (7.3-16.2)
		2020	0.71616977	31.3 (22.4-43.1)	12.4 (8.5-18.3)	14.0 (9.4-20.7)	8.1 (5.5-12.2)	20.1 (14.0-28.6)	11.5 (7.9-17.0)
	Venezuela	1990	0.51689122	30.3 (23.6-38.6)	14.6 (10.9-19.9)	16.3 (12.0-22.5)	11.3 (8.4-15.9)	21.4 (16.4-28.1)	15.3 (11.6-20.8)
		2020	0.60003478	17.7 (12.9-24.5)	8.1 (5.8-12.0)	8.7 (6.2-13.1)	5.9 (4.2-9.0)	12.0 (8.8-17.4)	8.2 (5.9-12.3)
Mid. East/North Africa	Algeria	1990	0.46048691	19.2 (13.1-27.7)	9.6 (6.2-14.6)	10.6 (6.9-16.2)	7.8 (5.1-11.9)	14.2 (9.5-20.9)	10.1 (6.8-15.1)
		2020	0.65365147	19.6 (13.3-28.5)	9.6 (6.2-14.6)	9.8 (6.3-14.7)	6.3 (4.1-9.5)	14.5 (9.5-21.3)	8.6 (5.6-12.8)
	Bahrain	1990	0.58457885	16.0 (10.9-23.0)	9.4 (6.3-14.4)	9.1 (6.2-13.7)	7.5 (5.0-11.3)	11.5 (7.8-16.9)	9.3 (6.3-14.1)
		2020	0.74810308	18.6 (12.5-27.3)	11.4 (7.5-17.5)	10.8 (7.6-16.2)	8.7 (5.8-12.7)	14.5 (9.8-20.9)	11.1 (7.3-16.2)
	Egypt, Arab Rep.	1990	0.41718274	10.0 (7.6-13.0)	5.0 (3.8-7.7)	6.1 (4.6-8.3)	4.2 (3.2-6.0)	7.4 (5.6-9.8)	5.5 (4.2-7.8)
		2020	0.59736334	9.5 (7.2-12.9)	4.7 (3.5-7.3)	5.2 (3.9-7.4)	3.8 (2.8-5.4)	6.5 (5.0-9.1)	4.8 (3.6-6.8)
	Iran, Islamic Rep.	1990	0.45379994	8.9 (7.4-11.4)	4.5 (3.8-6.7)	4.8 (4.0-6.7)	3.6 (3.0-5.2)	6.3 (5.3-8.4)	4.6 (3.8-6.4)
		2020	0.69191876	9.2 (7.6-12.0)	4.7 (3.9-6.6)	4.6 (3.8-6.4)	3.2 (2.6-4.4)	6.5 (5.5-8.7)	4.3 (3.6-5.8)

Supplementary Data 3. Sociodemographic development index (SDI) and proportional T2D and CVD burdens attributable to SSBs in 1990 and 2020 (continued).

World Region	Country	Year	SDI*	T2D incidence	CVD incidence	T2D deaths	CVD deaths	T2D DALYs	CVD DALYs
	Iraq	1990	0.41204417	20.9 (14.1-30.3)	9.4 (6.3-14.8)	10.7 (7.1-16.7)	7.1 (4.8-11.2)	14.2 (9.5-21.4)	9.1 (6.1-14.0)
		2020	0.65330038	18.0 (12.0-26.8)	8.4 (5.5-13.4)	8.9 (6.0-14.1)	5.8 (3.9-9.4)	12.6 (8.4-19.2)	7.3 (4.9-11.4)
	Israel	1990	0.70917835	20.0 (17.0-24.5)	8.5 (7.0-11.0)	10.6 (8.8-14.2)	7.5 (6.1-9.9)	13.8 (11.7-17.6)	9.1 (7.5-11.7)
		2020	0.80635159	16.1 (13.5-20.8)	6.6 (5.5-8.9)	6.5 (5.4-8.7)	4.3 (3.5-5.6)	10.2 (8.5-13.3)	5.7 (4.7-7.5)
	Jordan	1990	0.53914747	20.0 (15.5-25.5)	10.6 (7.9-14.3)	11.7 (8.7-16.1)	8.3 (6.2-11.0)	14.5 (11.1-19.0)	10.7 (8.1-14.1)
		2020	0.72006824	25.7 (19.8-33.5)	13.6 (10.1-18.7)	13.9 (10.5-18.9)	9.7 (7.3-13.4)	19.2 (14.6-25.2)	12.5 (9.4-16.7)
	Kuwait	1990	0.6645179	14.3 (9.4-21.5)	7.3 (4.8-12.5)	7.4 (4.9-12.0)	6.3 (4.1-10.7)	10.8 (7.2-16.8)	8.1 (5.3-13.3)
		2020	0.84345981	36.7 (26.1-49.2)	20.7 (14.3-30.1)	18.5 (12.7-27.0)	16.5 (11.2-24.8)	29.6 (21.0-40.7)	21.6 (14.8-31.7)
	Lebanon	1990	0.53671897	20.1 (16.8-24.7)	10.6 (8.6-14.3)	11.6 (9.4-15.0)	8.7 (7.1-11.6)	14.5 (12.0-18.3)	11.1 (9.1-14.7)
		2020	0.74200972	22.7 (18.8-28.4)	10.1 (8.2-13.4)	9.7 (7.7-12.9)	6.5 (5.2-8.5)	14.8 (12.3-18.5)	9.1 (7.5-11.8)
	Libya	1990	0.52799817	16.8 (11.8-23.9)	8.2 (5.5-12.3)	8.8 (6.1-13.3)	6.4 (4.5-9.5)	11.8 (8.3-16.9)	8.9 (6.2-12.9)
		2020	0.72027039	19.4 (13.4-28.1)	10.0 (6.8-14.8)	10.8 (7.5-16.2)	7.8 (5.4-11.5)	14.4 (10.2-21.0)	10.4 (7.2-15.1)
	Morocco	1990	0.35807287	14.0 (9.4-20.1)	6.5 (4.4-10.1)	7.2 (4.9-11.1)	5.3 (3.6-7.9)	9.8 (6.6-14.3)	6.9 (4.7-10.2)
		2020	0.55524607	16.9 (11.6-24.0)	7.8 (5.2-11.9)	8.6 (5.9-13.7)	5.8 (3.9-8.9)	11.9 (8.2-17.4)	7.3 (4.9-11.0)
	Oman	1990	0.42927095	6.3 (4.0-10.5)	3.0 (2.0-5.2)	3.2 (2.0-5.7)	2.5 (1.6-4.2)	4.1 (2.7-7.1)	3.1 (1.9-5.2)
		2020	0.76885422	15.4 (10.2-23.9)	8.2 (5.3-13.5)	7.9 (5.1-12.9)	5.7 (3.7-9.4)	11.3 (7.4-17.9)	7.5 (4.9-12.2)
	Palestine	1990	0.40179221	10.2 (6.9-14.5)	5.1 (3.4-7.9)	5.2 (3.5-7.9)	3.6 (2.4-5.5)	6.8 (4.6-9.9)	4.8 (3.2-7.2)
		2020	0.62381002	14.5 (10.0-20.6)	7.4 (4.9-11.2)	7.4 (5.0-11.0)	5.2 (3.4-7.8)	10.3 (6.9-14.7)	6.9 (4.6-10.1)
	Qatar	1990	0.65120838	18.7 (12.2-28.6)	11.5 (7.4-18.4)	10.8 (7.3-16.7)	8.8 (5.7-13.8)	14.4 (9.5-22.4)	11.1 (7.1-17.3)
		2020	0.8414674	21.2 (13.9-32.4)	13.2 (8.4-21.2)	12.4 (8.1-19.5)	10.2 (6.5-16.2)	17.6 (11.7-27.0)	12.7 (8.1-20.2)
	Saudi Arabia	1990	0.53895452	14.2 (10.1-20.5)	7.0 (4.9-10.8)	7.8 (5.3-12.1)	5.5 (3.8-8.6)	10.2 (7.2-15.3)	7.2 (5.0-10.7)
		2020	0.81054186	23.4 (16.3-33.6)	13.3 (9.1-20.0)	15.1 (10.6-22.2)	11.6 (8.1-17.5)	19.1 (13.2-27.5)	13.9 (9.5-20.6)
	Syrian Arab Republic	1990	0.43049264	9.2 (6.4-12.7)	4.1 (2.9-6.7)	4.6 (3.3-6.9)	3.6 (2.6-5.6)	6.3 (4.5-9.1)	5.1 (3.6-7.3)
		2020	0.61772759	10.9 (7.7-15.3)	5.6 (3.9-8.9)	6.1 (4.3-9.3)	4.6 (3.2-7.1)	8.1 (5.8-11.7)	5.7 (4.1-9.0)
	Tunisia	1990	0.47113852	13.3 (9.4-19.0)	6.7 (4.5-10.4)	7.1 (4.9-10.7)	5.1 (3.5-7.8)	9.5 (6.7-14.0)	6.4 (4.5-9.6)
		2020	0.67761515	21.8 (15.8-30.1)	11.2 (7.8-16.9)	11.9 (8.3-17.5)	7.8 (5.4-11.8)	16.2 (11.7-23.2)	10.1 (7.0-15.1)
	Turkey	1990	0.46160698	9.6 (7.1-13.1)	5.4 (3.9-7.9)	5.4 (3.9-7.8)	4.0 (2.9-5.6)	7.0 (5.1-9.8)	5.2 (3.8-7.4)
		2020	0.70579974	1.0 (0.5-3.0)	0.5 (0.2-1.5)	0.5 (0.2-1.4)	0.3 (0.2-0.9)	0.7 (0.3-2.0)	0.4 (0.2-1.3)
	United Arab Emirates	1990	0.64441227	17.3 (11.5-26.4)	10.3 (6.8-16.6)	10.1 (6.6-15.8)	7.9 (5.1-12.4)	13.1 (8.7-19.8)	9.8 (6.5-15.6)
		2020	0.84615198	7.3 (4.4-14.0)	4.6 (2.8-9.4)	4.7 (2.9-9.0)	3.7 (2.3-7.0)	6.3 (3.8-11.9)	4.3 (2.6-8.1)

Supplementary Data 3. Sociodemographic development index (SDI) and proportional T2D and CVD burdens attributable to SSBs in 1990 and 2020 (continued).

World Region	Country	Year	SDI*	T2D incidence	CVD incidence	T2D deaths	CVD deaths	T2D DALYs	CVD DALYs
	Yemen, Rep.	1990	0.21566459	32.1 (18.9-49.4)	17.2 (9.5-29.3)	18.9 (10.8-32.3)	14.5 (8.0-24.8)	23.4 (13.6-38.1)	17.3 (9.6-29.5)
		2020	0.44689307	41.7 (26.0-60.4)	22.3 (12.9-36.3)	23.8 (13.7-38.8)	17.5 (10.0-28.6)	31.5 (19.2-48.6)	21.6 (12.7-34.9)
South Asia	Afghanistan	1990	0.17383217	0.5 (0.2-1.7)	0.2 (0.1-0.5)	0.3 (0.1-0.8)	0.2 (0.1-0.4)	0.4 (0.2-1.0)	0.2 (0.1-0.5)
		2020	0.32983007	8.8 (4.4-17.0)	3.3 (1.5-7.1)	4.4 (2.1-9.6)	2.5 (1.1-5.4)	6.4 (3.1-12.8)	3.3 (1.6-7.3)
	Bangladesh	1990	0.22854893	0.8 (0.5-1.6)	0.3 (0.2-0.5)	0.3 (0.2-0.6)	0.2 (0.1-0.4)	0.5 (0.3-1.0)	0.3 (0.2-0.5)
		2020	0.48307917	1.0 (0.6-2.2)	0.4 (0.2-0.7)	0.4 (0.2-0.7)	0.3 (0.2-0.5)	0.6 (0.4-1.3)	0.4 (0.2-0.7)
	Bhutan	1990	0.21503985	4.4 (2.2-8.4)	1.4 (0.7-3.3)	2.0 (1.0-4.4)	1.2 (0.6-2.7)	2.8 (1.4-5.8)	1.5 (0.8-3.5)
		2020	0.46871448	10.3 (5.3-18.1)	3.9 (1.9-7.9)	4.0 (1.9-8.5)	2.8 (1.3-5.7)	6.5 (3.3-12.1)	3.9 (1.9-7.7)
	India	1990	0.3325936	0.7 (0.4-1.6)	0.2 (0.2-0.4)	0.3 (0.2-0.6)	0.2 (0.1-0.4)	0.5 (0.3-0.9)	0.3 (0.2-0.6)
		2020	0.56813815	0.7 (0.4-1.8)	0.3 (0.2-0.6)	0.3 (0.2-0.6)	0.2 (0.1-0.4)	0.5 (0.3-1.0)	0.3 (0.2-0.6)
	Maldives	1990	0.33160154	25.7 (16.8-35.1)	10.1 (6.0-15.7)	18.9 (11.2-27.7)	10.1 (6.0-15.5)	21.7 (13.5-30.7)	12.4 (7.6-18.8)
		2020	0.64760859	12.2 (6.9-19.3)	5.1 (2.8-8.7)	6.4 (3.5-11.0)	3.7 (2.0-6.4)	9.5 (5.3-15.3)	5.7 (3.2-9.4)
	Nepal	1990	0.19956065	1.2 (0.6-2.8)	0.4 (0.2-1.0)	0.6 (0.3-1.3)	0.4 (0.2-0.8)	0.8 (0.4-1.8)	0.5 (0.2-1.0)
		2020	0.42741709	6.1 (3.1-11.4)	2.2 (1.1-4.8)	2.5 (1.2-5.4)	1.7 (0.9-3.8)	3.9 (2.0-7.9)	2.3 (1.1-4.8)
	Pakistan	1990	0.31046762	17.3 (10.7-26.1)	6.1 (3.4-10.9)	8.5 (4.7-14.7)	5.0 (2.8-8.7)	11.8 (6.9-19.0)	6.5 (3.7-11.1)
		2020	0.49736441	16.8 (10.4-26.1)	7.3 (4.1-12.5)	8.2 (4.6-14.0)	5.9 (3.3-10.0)	11.8 (6.8-18.7)	7.8 (4.5-13.3)
	Sri Lanka	1990	0.52262255	9.6 (6.9-13.5)	3.5 (2.4-5.4)	5.7 (3.8-8.9)	2.9 (1.9-4.5)	7.5 (5.3-10.9)	4.0 (2.8-5.9)
		2020	0.69764854	18.8 (14.1-24.5)	8.3 (5.6-12.0)	11.5 (7.7-17.0)	6.5 (4.3-9.8)	14.7 (10.4-20.4)	8.7 (6.1-12.4)
Southeast and East Asia	Brunei	1990	0.66608192	6.5 (4.2-9.5)	2.1 (1.4-3.2)	2.5 (1.7-3.8)	2.1 (1.4-3.0)	3.8 (2.5-5.6)	3.4 (2.2-5.2)
		2020	0.80742409	14.8 (10.0-20.9)	4.9 (3.3-7.3)	6.2 (4.1-9.6)	5.1 (3.4-7.6)	9.4 (6.4-14.1)	7.3 (4.9-10.9)
	Cambodia	1990	0.28907506	0.3 (0.2-0.5)	0.1 (0.1-0.2)	0.1 (0.1-0.2)	0.1 (0.1-0.1)	0.2 (0.1-0.3)	0.1 (0.1-0.2)
		2020	0.46887605	11.8 (8.7-16.2)	4.6 (3.4-7.2)	5.9 (4.4-9.0)	3.6 (2.7-5.6)	8.0 (5.9-11.8)	5.3 (3.9-8.0)
	China	1990	0.45866894	1.2 (0.9-2.4)	0.3 (0.3-0.6)	0.4 (0.3-0.6)	0.2 (0.2-0.4)	0.7 (0.5-1.2)	0.3 (0.3-0.6)
		2020	0.71336458	0.8 (0.6-1.7)	0.2 (0.2-0.3)	0.2 (0.2-0.3)	0.1 (0.1-0.2)	0.5 (0.4-0.9)	0.2 (0.2-0.3)
	Fiji	1990	0.53464891	4.2 (2.9-6.6)	1.5 (1.1-2.5)	1.9 (1.3-3.2)	1.6 (1.1-2.9)	2.5 (1.7-4.3)	2.1 (1.5-3.8)
		2020	0.67143149	10.9 (7.7-15.2)	3.9 (2.8-5.9)	4.8 (3.4-7.4)	3.9 (2.8-6.0)	6.3 (4.5-9.5)	5.2 (3.7-7.9)
	Indonesia	1990	0.45713495	1.3 (1.0-2.7)	0.5 (0.4-0.9)	0.6 (0.5-1.1)	0.4 (0.3-0.9)	0.9 (0.6-1.6)	0.6 (0.5-1.4)
		2020	0.65192665	2.4 (1.7-4.0)	0.9 (0.7-1.5)	1.2 (0.8-1.9)	0.8 (0.6-1.3)	1.6 (1.2-2.6)	1.1 (0.8-2.0)
	Japan	1990	0.79025352	7.4 (6.1-10.5)	2.2 (1.9-3.0)	2.7 (2.3-3.9)	1.4 (1.2-1.8)	4.6 (3.9-6.7)	2.1 (1.8-2.8)
		2020	0.86910688	5.9 (4.9-8.8)	1.5 (1.2-2.0)	1.7 (1.4-2.3)	1.0 (0.8-1.2)	3.4 (2.9-5.1)	1.5 (1.3-2.1)
	Kiribati	1990	0.41038982	3.9 (2.5-6.9)	1.2 (0.7-2.2)	1.5 (1.0-2.9)	1.2 (0.8-2.4)	2.1 (1.3-3.9)	1.6 (1.0-3.3)

Supplementary Data 3. Sociodemographic development index (SDI) and proportional T2D and CVD burdens attributable to SSBs in 1990 and 2020 (continued).

World Region	Country	Year	SDI*	T2D incidence	CVD incidence	T2D deaths	CVD deaths	T2D DALYs	CVD DALYs
		2020	0.52345411	7.9 (5.0-12.5)	2.3 (1.5-3.9)	3.0 (1.9-5.2)	2.5 (1.6-4.4)	4.2 (2.7-7.2)	3.3 (2.1-5.9)
	Korea, Rep.	1990	0.69232931	3.5 (3.0-6.2)	0.9 (0.8-1.3)	1.4 (1.2-2.2)	0.6 (0.5-0.9)	2.0 (1.8-3.5)	0.9 (0.8-1.5)
		2020	0.88371757	3.0 (2.6-5.0)	0.8 (0.7-1.1)	0.9 (0.8-1.2)	0.5 (0.4-0.6)	1.8 (1.6-2.9)	0.7 (0.6-1.0)
	Lao PDR	1990	0.26428316	1.2 (0.8-2.6)	0.4 (0.3-0.8)	0.6 (0.4-1.1)	0.4 (0.3-0.8)	0.8 (0.5-1.6)	0.6 (0.4-1.3)
		2020	0.48419296	3.8 (2.8-5.9)	1.4 (1.0-2.1)	1.8 (1.3-3.0)	1.2 (0.9-1.9)	2.5 (1.9-4.2)	1.9 (1.4-3.1)
	Malaysia	1990	0.54579941	6.1 (4.5-8.5)	2.0 (1.5-2.9)	2.5 (1.9-3.8)	1.7 (1.3-2.6)	3.7 (2.8-5.3)	2.5 (1.9-3.8)
		2020	0.73874399	5.7 (4.2-8.2)	2.1 (1.6-3.5)	2.7 (2.1-4.3)	1.8 (1.4-3.0)	3.8 (2.9-5.9)	2.6 (2.0-4.3)
	Marshall Islands	1990	0.43083929	5.1 (3.3-8.3)	1.3 (0.9-2.2)	1.7 (1.2-2.9)	1.3 (0.9-2.5)	2.6 (1.8-4.3)	1.9 (1.3-3.3)
		2020	0.56883751	13.7 (9.5-19.7)	3.8 (2.7-5.8)	5.0 (3.5-8.5)	4.2 (2.9-6.8)	7.1 (5.1-11.3)	5.4 (3.7-8.6)
	Micronesia, Fed. Sts.	1990	0.46251162	4.3 (2.9-7.0)	1.2 (0.8-1.9)	1.6 (1.1-2.6)	1.2 (0.9-2.1)	2.3 (1.6-3.8)	1.7 (1.2-3.1)
		2020	0.58452723	12.1 (8.4-17.1)	3.6 (2.6-5.7)	4.9 (3.5-7.5)	3.9 (2.7-6.2)	6.7 (4.8-9.9)	5.2 (3.6-8.1)
	Myanmar	1990	0.31921972	1.1 (0.7-2.3)	0.4 (0.3-0.7)	0.6 (0.4-1.0)	0.4 (0.3-0.7)	0.8 (0.5-1.4)	0.6 (0.4-1.1)
		2020	0.5294347	8.3 (5.9-12.4)	3.1 (2.2-4.8)	4.1 (2.9-6.5)	2.4 (1.7-3.8)	5.6 (4.0-8.6)	3.7 (2.6-5.6)
	Papua New Guinea	1990	0.31066863	3.7 (2.4-7.1)	1.1 (0.8-2.2)	1.4 (1.0-2.9)	1.1 (0.7-2.3)	2.0 (1.4-4.0)	1.4 (0.9-3.0)
		2020	0.41518688	0.0 (0.0-0.0)	0.0 (0.0-0.0)	0.0 (0.0-0.0)	0.0 (0.0-0.0)	0.0 (0.0-0.0)	0.0 (0.0-0.0)
	Philippines	1990	0.5100118	9.1 (7.4-11.1)	3.9 (3.1-5.0)	5.2 (4.2-6.8)	3.7 (3.0-4.8)	7.3 (5.9-9.1)	5.9 (4.7-7.6)
		2020	0.64486723	8.0 (6.5-10.1)	3.4 (2.7-4.4)	4.3 (3.5-5.8)	3.1 (2.5-4.1)	5.8 (4.6-7.6)	4.6 (3.7-6.1)
	Samoa	1990	0.48749143	8.5 (5.6-13.3)	2.3 (1.5-3.7)	2.8 (1.9-4.7)	2.0 (1.3-3.4)	4.2 (2.7-6.9)	2.9 (1.8-5.0)
		2020	0.5900753	25.5 (17.8-36.1)	6.8 (4.5-10.4)	8.7 (5.7-13.3)	6.3 (4.1-9.8)	13.4 (9.0-20.0)	9.1 (5.9-13.9)
	Singapore	1990	0.68640444	9.8 (7.5-15.2)	2.9 (2.3-4.6)	3.3 (2.6-5.4)	2.4 (1.9-3.8)	5.5 (4.3-8.5)	3.4 (2.7-5.5)
		2020	0.85429601	8.8 (6.8-13.5)	2.8 (2.2-4.9)	2.7 (2.2-4.4)	2.2 (1.7-3.5)	5.9 (4.6-9.4)	3.1 (2.5-5.4)
	Solomon Islands	1990	0.30121717	15.6 (10.0-22.7)	4.8 (3.1-8.0)	6.8 (4.3-11.0)	4.8 (3.0-8.1)	9.0 (5.8-14.2)	6.4 (4.0-10.7)
		2020	0.42611243	12.6 (7.9-19.2)	3.8 (2.4-6.4)	5.7 (3.6-9.3)	3.9 (2.4-6.6)	7.7 (4.8-12.4)	5.3 (3.3-8.9)
	Taiwan	1990	0.66763385	17.5 (14.1-21.7)	6.3 (5.0-8.1)	6.4 (5.1-8.7)	3.8 (3.1-5.1)	9.4 (7.6-12.1)	5.5 (4.4-7.0)
		2020	0.87124795	12.7 (10.1-16.4)	4.7 (3.8-6.2)	4.2 (3.4-5.9)	3.0 (2.4-4.0)	7.4 (5.9-9.7)	4.5 (3.5-5.9)
	Thailand	1990	0.50664486	2.3 (1.0-5.1)	0.8 (0.4-1.8)	1.1 (0.5-2.5)	0.6 (0.3-1.5)	1.5 (0.7-3.4)	1.0 (0.5-2.3)
		2020	0.67911999	10.5 (5.1-20.0)	4.3 (2.1-8.6)	5.5 (2.7-11.2)	3.2 (1.5-6.6)	7.6 (3.7-14.8)	4.9 (2.4-9.8)
	Timor-Leste	1990	0.26246808	1.6 (1.0-3.3)	0.6 (0.4-1.3)	0.7 (0.5-1.6)	0.5 (0.3-1.1)	1.0 (0.6-2.3)	0.8 (0.5-1.7)
		2020	0.44234934	31.0 (24.6-38.9)	13.5 (10.2-18.6)	17.2 (12.6-23.4)	11.5 (8.6-15.8)	22.4 (17.2-29.5)	16.1 (12.2-21.7)
	Tonga	1990	0.49180684	5.7 (3.5-9.9)	1.7 (1.1-3.1)	2.1 (1.4-3.9)	1.5 (1.0-3.0)	3.0 (1.9-5.5)	2.1 (1.3-4.1)

Supplementary Data 3. Sociodemographic development index (SDI) and proportional T2D and CVD burdens attributable to SSBs in 1990 and 2020 (continued).

World Region	Country	Year	SDI*	T2D incidence	CVD incidence	T2D deaths	CVD deaths	T2D DALYs	CVD DALYs
		2020	0.62210446	20.3 (13.6-30.0)	5.4 (3.4-8.9)	6.7 (4.3-10.6)	4.8 (3.0-7.7)	10.3 (6.6-16.0)	7.1 (4.5-11.7)
	Vanuatu	1990	0.35310025	5.1 (3.0-8.7)	1.5 (0.9-2.8)	1.9 (1.2-3.5)	1.6 (1.0-3.0)	2.7 (1.6-4.7)	2.1 (1.3-4.2)
		2020	0.47003839	8.5 (5.0-13.4)	2.3 (1.4-3.8)	3.0 (1.8-4.9)	2.5 (1.5-4.3)	4.4 (2.7-7.2)	3.4 (2.1-6.1)
	Vietnam	1990	0.40763005	3.0 (2.1-4.8)	1.1 (0.8-1.8)	1.2 (0.9-2.2)	0.7 (0.5-1.0)	1.8 (1.3-3.1)	1.0 (0.7-1.7)
		2020	0.62309032	4.0 (3.0-6.2)	1.7 (1.3-2.8)	1.9 (1.4-3.3)	1.0 (0.8-1.5)	2.8 (2.1-4.5)	1.5 (1.1-2.4)
Sub-Saharan Africa	Angola	1990	0.27073694	13.8 (9.6-20.2)	6.7 (4.4-10.4)	8.9 (5.7-13.9)	5.3 (3.4-8.8)	11.0 (7.2-16.4)	6.5 (4.2-10.3)
		2020	0.44728399	26.6 (18.6-36.1)	12.8 (8.3-18.7)	15.9 (10.4-23.2)	9.5 (6.0-14.5)	20.7 (14.2-28.9)	11.9 (7.8-17.5)
	Benin	1990	0.21890715	14.1 (9.0-21.6)	5.8 (3.6-9.9)	7.5 (4.6-12.7)	3.4 (2.1-6.1)	10.6 (6.6-17.2)	4.6 (2.8-7.9)
		2020	0.36696449	25.1 (17.0-36.1)	11.2 (6.9-17.6)	12.8 (8.0-20.3)	6.6 (3.9-10.8)	19.1 (12.3-28.3)	8.9 (5.6-14.3)
	Botswana	1990	0.41807775	16.6 (11.6-23.0)	8.8 (5.7-12.8)	10.6 (6.6-16.7)	6.5 (4.1-10.1)	12.8 (8.3-19.1)	8.1 (5.3-12.3)
		2020	0.6392753	40.9 (29.9-52.4)	23.0 (15.9-30.9)	25.8 (16.6-36.6)	16.2 (10.4-23.3)	32.0 (22.1-43.0)	20.9 (14.1-28.7)
	Burkina Faso	1990	0.12969562	2.0 (0.6-5.9)	1.0 (0.3-3.0)	1.3 (0.4-3.9)	0.7 (0.2-2.0)	1.7 (0.5-5.1)	0.9 (0.3-2.6)
		2020	0.27987132	11.4 (3.9-27.9)	4.9 (1.6-13.1)	6.3 (2.1-16.7)	3.1 (1.0-8.9)	8.9 (3.0-22.3)	4.1 (1.3-11.0)
	Burundi	1990	0.20586736	11.9 (7.8-17.8)	5.9 (3.8-10.2)	8.0 (5.1-12.9)	4.6 (2.9-8.2)	10.1 (6.5-15.6)	6.0 (3.8-10.2)
		2020	0.286496	21.0 (14.5-30.1)	10.4 (6.7-16.8)	12.7 (8.2-19.8)	8.0 (5.0-13.5)	16.6 (11.1-24.8)	10.5 (6.9-16.7)
	Cameroon	1990	0.30305533	6.5 (4.1-10.0)	3.1 (2.0-5.1)	3.8 (2.4-6.3)	2.0 (1.3-3.4)	5.1 (3.2-8.1)	2.7 (1.7-4.4)
		2020	0.47228585	24.9 (17.3-33.1)	12.0 (8.0-17.5)	14.6 (9.5-21.3)	8.0 (5.2-12.7)	19.4 (13.4-27.1)	10.6 (7.0-15.6)
	Cape Verde	1990	0.27672337	7.1 (4.7-11.1)	3.0 (1.9-5.1)	3.7 (2.3-6.0)	2.0 (1.2-3.3)	5.5 (3.6-8.4)	2.8 (1.8-4.6)
		2020	0.52846159	11.1 (7.2-17.4)	4.7 (2.9-7.9)	4.8 (3.0-8.2)	2.7 (1.7-4.6)	8.0 (5.2-12.5)	4.0 (2.5-6.7)
	Central African Republic	1990	0.21682519	6.7 (4.3-10.4)	2.9 (1.8-4.8)	4.2 (2.7-6.9)	2.4 (1.5-4.1)	5.1 (3.4-8.2)	2.9 (1.8-4.7)
		2020	0.3054263	8.9 (5.9-14.1)	3.7 (2.4-6.6)	5.2 (3.4-8.9)	2.9 (1.9-5.2)	6.7 (4.4-11.0)	3.7 (2.4-6.5)
	Chad	1990	0.11463883	11.3 (7.6-16.5)	5.0 (3.1-8.4)	6.4 (4.0-10.4)	3.3 (2.0-5.6)	8.7 (5.6-13.5)	4.3 (2.7-7.3)
		2020	0.23610308	18.9 (13.0-26.2)	8.3 (5.3-13.0)	10.4 (6.7-15.8)	5.4 (3.4-8.9)	14.4 (9.5-20.1)	7.1 (4.6-11.1)
	Comoros	1990	0.27004812	15.2 (10.6-21.0)	7.7 (5.0-11.8)	10.7 (7.0-16.0)	5.8 (3.7-9.1)	13.3 (9.1-19.4)	7.4 (5.0-11.6)
		2020	0.47200313	22.4 (16.2-30.0)	10.9 (7.4-16.3)	13.2 (8.9-19.5)	7.8 (5.2-12.0)	17.8 (12.6-25.1)	10.5 (7.2-15.4)
	Congo, Dem. Rep.	1990	0.28984321	5.5 (1.9-15.3)	2.4 (0.8-7.5)	3.4 (1.2-10.1)	1.9 (0.6-5.8)	4.3 (1.5-11.9)	2.3 (0.8-7.0)
		2020	0.37671227	7.8 (2.7-21.1)	3.2 (1.1-9.4)	4.2 (1.5-11.8)	2.3 (0.8-6.7)	5.7 (2.0-15.9)	2.9 (1.0-8.5)
	Congo, Rep.	1990	0.42065466	7.3 (4.8-11.2)	3.4 (2.2-5.6)	4.8 (3.1-8.0)	2.9 (1.8-4.8)	5.9 (3.9-9.5)	3.5 (2.3-5.8)
		2020	0.57845155	28.1 (19.9-37.4)	14.0 (9.5-20.4)	17.9 (12.0-26.2)	10.9 (7.0-16.5)	22.5 (15.5-31.4)	13.7 (9.2-19.9)
	Cote d'Ivoire	1990	0.27932035	12.8 (8.7-18.7)	6.2 (4.1-9.8)	7.7 (5.0-11.9)	4.4 (2.8-7.1)	10.1 (6.8-15.1)	5.6 (3.7-8.9)

Supplementary Data 3. Sociodemographic development index (SDI) and proportional T2D and CVD burdens attributable to SSBs in 1990 and 2020 (continued).

World Region	Country	Year	SDI*	T2D incidence	CVD incidence	T2D deaths	CVD deaths	T2D DALYs	CVD DALYs
		2020	0.4190401	21.9 (15.2-30.2)	10.8 (7.2-15.7)	12.7 (8.4-18.4)	7.4 (4.8-11.2)	17.3 (11.7-24.4)	9.6 (6.5-14.3)
	Djibouti	1990	0.33778179	31.2 (22.3-41.4)	18.4 (12.1-26.7)	22.8 (15.1-32.9)	14.2 (9.1-21.6)	27.5 (19.2-37.9)	17.8 (11.8-26.5)
		2020	0.48187995	48.5 (36.1-59.0)	30.0 (20.5-40.3)	35.9 (24.6-46.8)	24.4 (16.2-33.7)	43.0 (31.1-54.0)	29.5 (20.4-39.6)
	Equatorial Guinea	1990	0.26878363	11.6 (7.7-16.5)	5.1 (3.2-8.2)	7.5 (4.9-11.8)	4.2 (2.6-7.1)	9.2 (6.1-13.9)	5.1 (3.3-8.4)
		2020	0.6521249	23.2 (15.8-32.6)	11.1 (7.1-16.7)	13.1 (8.5-19.8)	7.9 (5.0-12.4)	17.9 (12.0-25.6)	10.5 (6.6-15.7)
	Eritrea	1990	0.21602824	18.0 (12.5-24.9)	9.0 (5.9-14.1)	13.1 (8.6-19.9)	7.9 (5.1-12.9)	15.5 (10.2-22.4)	9.5 (6.1-15.2)
		2020	0.40045941	30.2 (21.8-39.2)	14.5 (9.6-20.9)	19.4 (12.6-27.3)	12.0 (7.7-17.5)	24.6 (16.9-33.3)	15.3 (10.1-21.7)
	Ethiopia (excludes Eritrea)	1990	0.14803388	11.9 (8.8-16.0)	6.0 (4.3-9.2)	8.3 (5.8-12.0)	5.2 (3.6-7.9)	10.1 (7.4-14.3)	6.4 (4.6-9.7)
		2020	0.35321635	23.8 (18.7-29.8)	11.0 (8.2-15.2)	13.0 (9.3-17.6)	7.9 (5.6-11.0)	18.3 (13.9-23.4)	10.8 (8.0-14.7)
	Gabon	1990	0.45542119	28.3 (20.3-37.3)	14.7 (9.8-21.4)	19.0 (12.2-27.7)	11.5 (7.2-17.7)	23.4 (15.9-32.7)	14.3 (9.3-21.0)
		2020	0.62760965	21.2 (14.3-29.6)	10.3 (6.6-15.8)	12.8 (7.9-19.7)	7.5 (4.6-12.0)	16.5 (10.8-24.2)	9.7 (6.1-15.1)
	Gambia, The	1990	0.23871485	49.8 (38.9-59.8)	28.7 (20.0-38.6)	34.5 (24.6-45.6)	20.8 (13.9-30.3)	42.4 (31.4-53.1)	25.6 (17.5-34.8)
		2020	0.40452419	21.8 (14.8-31.1)	9.7 (6.1-14.8)	11.7 (7.4-18.1)	6.4 (3.9-10.3)	16.4 (10.8-24.0)	8.6 (5.5-13.2)
	Ghana	1990	0.373112	17.7 (12.4-24.5)	7.6 (5.0-11.4)	10.8 (7.1-16.1)	5.7 (3.6-8.7)	14.3 (9.8-20.4)	7.3 (4.8-10.9)
		2020	0.55846217	26.5 (19.3-35.4)	12.4 (8.5-17.9)	15.2 (10.3-22.4)	8.0 (5.2-12.1)	20.4 (14.5-28.1)	10.6 (7.2-15.3)
	Guinea	1990	0.17829542	4.4 (2.8-6.8)	1.9 (1.2-3.3)	2.5 (1.5-4.2)	1.3 (0.7-2.2)	3.4 (2.1-5.5)	1.6 (1.0-2.8)
		2020	0.329878	9.6 (6.2-13.9)	3.9 (2.4-6.5)	4.9 (3.1-7.7)	2.5 (1.5-4.2)	7.1 (4.5-10.6)	3.4 (2.1-5.6)
	Guinea-Bissau	1990	0.20761484	0.2 (0.1-0.3)	0.1 (0.0-0.1)	0.1 (0.1-0.2)	0.1 (0.0-0.1)	0.1 (0.1-0.2)	0.1 (0.0-0.1)
		2020	0.347799	27.1 (19.2-35.7)	12.7 (8.4-18.3)	16.8 (11.2-23.9)	9.5 (6.0-14.2)	21.7 (14.9-29.3)	12.0 (7.9-17.6)
	Kenya	1990	0.33385029	5.0 (2.8-8.7)	2.5 (1.4-4.9)	3.1 (1.7-5.9)	1.6 (0.8-3.1)	4.2 (2.3-7.6)	2.2 (1.2-4.1)
		2020	0.51613657	3.5 (1.9-6.2)	1.7 (0.9-3.5)	2.1 (1.1-4.2)	1.2 (0.6-2.3)	2.8 (1.5-5.2)	1.6 (0.9-3.1)
	Lesotho	1990	0.33915513	13.5 (9.2-19.8)	6.8 (4.4-11.3)	8.7 (5.4-13.7)	4.0 (2.4-6.7)	10.5 (6.7-16.2)	5.3 (3.3-8.7)
		2020	0.50674665	29.8 (21.5-39.7)	15.9 (10.9-23.0)	19.8 (12.8-29.3)	11.2 (7.3-17.4)	23.3 (16.0-32.8)	14.3 (9.5-21.1)
	Liberia	1990	0.23529685	8.2 (5.1-12.8)	3.4 (2.1-5.9)	4.6 (2.7-7.9)	2.3 (1.3-4.3)	6.2 (3.8-10.2)	3.0 (1.8-5.1)
		2020	0.34813389	29.5 (20.9-39.3)	13.6 (9.1-20.3)	17.2 (11.4-25.3)	9.0 (5.8-14.1)	23.6 (16.2-32.7)	12.2 (8.1-18.3)
	Madagascar	1990	0.27988946	16.7 (11.3-23.8)	8.2 (5.2-13.1)	11.5 (7.4-17.6)	6.4 (4.1-10.5)	14.7 (9.7-21.3)	8.5 (5.4-13.4)
		2020	0.39277871	19.6 (13.8-27.7)	9.2 (5.8-14.2)	12.6 (8.4-18.8)	7.5 (4.9-11.8)	16.2 (11.1-23.3)	9.8 (6.4-15.1)
	Malawi	1990	0.20401024	7.3 (4.8-11.4)	3.9 (2.5-6.9)	5.2 (3.3-8.7)	2.9 (1.8-5.0)	6.5 (4.2-10.3)	3.9 (2.5-6.5)
		2020	0.37787902	3.1 (2.0-5.4)	1.5 (1.0-2.8)	2.0 (1.2-3.4)	1.1 (0.7-2.0)	2.6 (1.6-4.5)	1.5 (1.0-2.8)

Supplementary Data 3. Sociodemographic development index (SDI) and proportional T2D and CVD burdens attributable to SSBs in 1990 and 2020 (continued).

World Region	Country	Year	SDI*	T2D incidence	CVD incidence	T2D deaths	CVD deaths	T2D DALYs	CVD DALYs
	Mali	1990	0.12652643	5.8 (3.6-9.9)	2.7 (1.6-4.9)	3.3 (2.1-6.0)	1.8 (1.1-3.4)	4.5 (2.8-7.9)	2.4 (1.4-4.6)
		2020	0.26262922	7.8 (4.7-13.5)	3.4 (2.0-6.1)	4.0 (2.4-7.2)	2.1 (1.3-3.9)	5.9 (3.6-10.2)	2.9 (1.8-5.3)
	Mauritania	1990	0.33578094	12.0 (7.8-18.1)	5.3 (3.3-8.7)	7.0 (4.3-11.4)	3.7 (2.3-6.4)	9.3 (5.8-14.3)	4.7 (2.9-8.0)
		2020	0.4913656	32.9 (23.0-44.9)	16.1 (10.5-24.6)	17.6 (11.1-26.8)	10.1 (6.2-16.2)	24.3 (16.2-34.7)	13.4 (8.6-20.3)
	Mauritius	1990	0.54458653	60.6 (52.9-68.1)	29.9 (22.4-38.1)	46.5 (36.3-56.3)	28.8 (21.2-37.1)	51.5 (41.8-60.6)	35.4 (27.5-43.7)
		2020	0.7146298	47.7 (38.8-57.2)	22.9 (16.6-31.3)	33.1 (24.7-43.7)	20.6 (14.8-28.2)	39.2 (30.1-49.8)	27.6 (21.0-35.5)
	Mozambique	1990	0.17306472	4.3 (2.4-7.7)	2.3 (1.3-4.8)	3.1 (1.7-6.1)	1.3 (0.7-2.4)	3.9 (2.1-7.6)	1.7 (0.9-3.2)
		2020	0.32029118	8.8 (4.9-14.2)	4.0 (2.2-7.2)	5.6 (3.1-9.9)	2.4 (1.3-4.3)	7.1 (4.0-12.3)	3.2 (1.8-5.7)
	Namibia	1990	0.45004023	38.5 (28.4-50.8)	22.0 (15.2-31.8)	26.6 (17.4-39.7)	16.2 (10.5-24.8)	31.0 (21.2-43.7)	20.0 (13.3-29.1)
		2020	0.61443589	44.7 (33.2-58.0)	24.3 (16.6-34.6)	27.8 (18.2-40.7)	17.2 (11.1-25.8)	34.1 (23.7-47.1)	22.2 (15.0-32.1)
	Niger	1990	0.08086848	6.4 (3.9-10.7)	2.9 (1.7-5.5)	3.7 (2.2-6.6)	1.9 (1.1-3.6)	5.0 (3.0-8.7)	2.5 (1.4-4.5)
		2020	0.16453926	0.0 (0.0-0.0)	0.0 (0.0-0.0)	0.0 (0.0-0.0)	0.0 (0.0-0.0)	0.0 (0.0-0.0)	0.0 (0.0-0.0)
	Nigeria	1990	0.30586805	1.6 (1.0-3.1)	0.7 (0.4-1.4)	0.9 (0.6-1.7)	0.5 (0.3-0.9)	1.2 (0.8-2.4)	0.6 (0.4-1.1)
		2020	0.49620474	15.5 (10.7-22.1)	7.7 (5.0-11.8)	8.4 (5.2-13.0)	4.8 (3.0-7.7)	11.8 (7.8-17.3)	6.6 (4.2-10.1)
	Rwanda	1990	0.27509719	3.7 (2.2-7.0)	1.8 (1.1-3.6)	2.6 (1.5-4.9)	1.4 (0.8-2.8)	3.2 (1.9-6.1)	1.9 (1.1-3.8)
		2020	0.4298966	66.5 (59.9-71.8)	46.5 (38.8-53.3)	53.8 (45.5-60.6)	36.2 (28.4-43.6)	61.4 (54.0-67.4)	43.1 (35.7-50.0)
	Sao Tome and Principe	1990	0.30954285	9.3 (6.0-13.6)	3.8 (2.3-6.1)	4.9 (3.0-8.0)	2.4 (1.5-4.0)	7.0 (4.4-10.6)	3.2 (2.0-5.3)
		2020	0.49786141	17.2 (11.5-24.6)	7.7 (4.8-12.2)	8.8 (5.5-14.2)	4.9 (3.0-8.1)	13.6 (8.9-20.0)	6.7 (4.2-10.7)
	Senegal	1990	0.23804761	28.2 (20.3-37.5)	14.9 (9.8-21.8)	17.5 (11.7-26.2)	10.4 (6.6-16.2)	23.0 (16.1-31.7)	13.3 (8.9-19.8)
		2020	0.40119752	45.3 (34.8-55.6)	24.9 (17.7-33.9)	27.5 (19.2-37.9)	16.7 (10.9-24.3)	36.2 (26.8-46.5)	21.3 (15.0-29.3)
	Seychelles	1990	0.5755265	22.9 (18.5-28.4)	9.8 (7.5-13.3)	13.9 (10.4-19.2)	8.9 (6.6-12.4)	17.8 (14.0-22.8)	12.6 (9.8-16.4)
		2020	0.72666377	25.4 (20.1-31.7)	11.8 (9.0-15.8)	15.5 (11.8-20.9)	10.3 (7.8-14.0)	20.1 (15.9-25.8)	14.4 (11.2-18.7)
	Sierra Leone	1990	0.21156934	8.5 (5.6-13.2)	3.7 (2.3-6.3)	4.6 (2.8-8.1)	2.5 (1.5-4.5)	6.4 (4.1-10.2)	3.2 (2.0-5.7)
		2020	0.35241202	29.4 (20.9-39.1)	13.5 (8.9-19.8)	16.4 (10.8-24.2)	9.3 (5.9-14.6)	22.7 (16.0-31.1)	12.2 (8.1-18.0)
	South Africa	1990	0.54157144	31.8 (26.2-38.7)	17.2 (13.5-21.8)	20.5 (15.9-26.4)	13.2 (10.2-17.0)	26.3 (21.2-33.2)	18.7 (15.0-23.5)
		2020	0.6771661	27.6 (22.1-34.6)	14.6 (11.4-19.2)	16.4 (12.3-22.3)	9.6 (7.1-13.0)	20.9 (16.3-27.4)	13.1 (10.2-17.3)
	Sudan	1990	0.29217864	23.2 (15.0-33.7)	10.4 (6.3-16.3)	12.6 (7.6-19.7)	8.6 (5.2-13.6)	16.6 (10.5-24.7)	11.2 (7.0-17.1)
		2020	0.53345542	26.5 (17.1-38.3)	11.7 (7.2-18.2)	13.7 (8.4-21.2)	8.8 (5.4-13.9)	19.5 (12.4-28.7)	12.0 (7.4-18.3)
	Swaziland	1990	0.39942095	24.3 (16.9-33.4)	13.5 (8.9-20.1)	15.8 (10.0-23.7)	9.3 (5.9-14.4)	19.0 (12.5-27.8)	12.1 (7.9-18.3)
		2020	0.58165307	30.3 (21.4-41.2)	16.6 (11.1-24.0)	20.1 (13.0-29.5)	12.7 (8.3-18.9)	23.9 (16.1-33.8)	16.3 (10.8-23.7)

Supplementary Data 3. Sociodemographic development index (SDI) and proportional T2D and CVD burdens attributable to SSBs in 1990 and 2020 (continued).

World Region	Country	Year	SDI*	T2D incidence	CVD incidence	T2D deaths	CVD deaths	T2D DALYs	CVD DALYs
	Tanzania	1990	0.25930607	1.4 (0.9-2.8)	0.7 (0.4-1.4)	1.0 (0.6-2.1)	0.6 (0.3-1.1)	1.3 (0.8-2.6)	0.7 (0.5-1.4)
		2020	0.43874724	21.4 (14.9-29.0)	11.3 (7.4-16.3)	13.4 (8.6-19.6)	8.3 (5.3-12.8)	17.7 (11.7-24.8)	11.3 (7.4-16.4)
	Togo	1990	0.26969227	7.0 (4.4-11.0)	3.0 (1.8-5.2)	4.0 (2.5-6.9)	2.0 (1.2-3.5)	5.4 (3.4-8.8)	2.7 (1.6-4.6)
		2020	0.40235692	66.4 (58.9-71.9)	42.8 (34.3-50.1)	51.2 (42.0-58.3)	33.1 (25.5-40.1)	59.2 (50.9-65.5)	38.9 (31.5-46.3)
	Uganda	1990	0.1870011	0.5 (0.3-1.4)	0.3 (0.2-0.5)	0.3 (0.2-0.7)	0.2 (0.1-0.4)	0.4 (0.3-0.9)	0.3 (0.2-0.5)
		2020	0.41684608	30.5 (22.2-39.2)	15.5 (10.5-22.0)	19.0 (12.7-26.8)	11.7 (7.5-17.2)	25.0 (17.6-33.7)	15.6 (10.4-22.3)
	Zambia	1990	0.30400855	38.4 (29.2-47.6)	21.3 (15.2-28.8)	28.6 (20.3-38.0)	15.5 (10.5-22.1)	34.7 (25.7-44.3)	19.9 (13.9-26.9)
		2020	0.49748556	17.7 (12.1-24.1)	8.1 (5.3-11.8)	10.6 (6.8-15.3)	5.8 (3.6-8.8)	14.2 (9.5-19.4)	7.8 (5.0-11.5)
	Zimbabwe	1990	0.39855934	11.9 (8.1-17.2)	6.3 (4.0-9.7)	7.0 (4.3-11.4)	3.9 (2.4-6.3)	8.9 (5.7-13.7)	5.0 (3.2-7.8)
		2020	0.47157317	28.8 (20.7-38.5)	15.3 (10.5-21.9)	18.2 (12.1-26.6)	11.0 (7.3-16.5)	22.0 (14.9-31.0)	14.1 (9.7-20.3)

*SDI, Sociodemographic development index is a measure of a nation's development expressed on a scale of 0 to 1 sourced from the Global Burden of Disease study, based on a compositive average of the rankings of income per capita, average educational attainment and fertility rates.

CVD, cardiovascular disease; SSBs, Sugar-sweetened beverages; T2D, type 2 diabetes
