# Supplementary material for: Burdens of type 2 diabetes and cardiovascular disease attributable to sugar-sweetened beverages in 184 countries
Source: Nat Med. 2025 Jan 6;31(2):552–64. doi: 10.1038/s41591-024-03345-4 (PMC11835746; doi:10.1038/s41591-024-03345-4)
Supplement: Supplementary file 6 — Age-at-event calculation for the etiologic effect of BMI on ischemic heart disease. [file 41591_2024_3345_MOESM6_ESM.pdf]

Burdens of type 2 diabetes and cardiovascular disease burdens to sugar-sweetened beverages in 184 countries

Supplementary Data 4 | Age at event calculation for the etiologic effect of BMI on ischemic heart disease.

Supplementary Data 4. Age at event calculation for the etiologic effect of BMI on ischemic heart disease*.

Study	Weighting	Weighting rescaled	Weighting fraction	Age at baseline¶ (y)	Type of follow-up time	Follow-up time (y)	Mean or 2/3 follow-up time§	Age at event‡ (y)	Weighted age at event# (y)
BMI on IHD (Lu *et al.*, 2014)									
Abdominal Aortic Aneurysm Screening Program	0.016	NA	0.016	74.5	mean follow-up	3.2	2	76.6	1.23
The age 40-programme linked with the National Cause of Death Register	0.0228	NA	0.023	42.0	max follow-up	24	12.0	54.0	1.23
Aito Town Study	0.0003	NA	0.000	47.0	mean follow-up	16.4	10.9	57.9	0.02
Akabane Study	0.0029	NA	0.003	54.5	mean follow-up	11.2	7.5	62.0	0.18
Atherosclerosis Risk in Communities Study (ARIC)	0.0223	NA	0.022	54.5	mean follow-up	17	11.3	65.8	1.47
Australia Longitudinal Study of Ageing (ALSA)	0.0031	NA	0.003	81.5	mean follow-up	5.7	3.8	85.3	0.26
Busselton Health Study	0.0204	NA	0.020	57.0	mean follow-up	24.1	16.1	73.1	1.49
Busselton Health Stud, phase II	0.0188	NA	0.019	57.5	mean follow-up	14	9.3	66.8	1.26
Canada Nutrition Database	0.0198	NA	0.020	58.5	mean follow-up	22	14.7	73.2	1.45
Capital Iron and Steel Company Hospital Cohort (CISCH)	0.0021	NA	0.002	49.0	mean follow-up	3.3	2.2	51.2	0.11
CVDFACTS	0.0134	NA	0.013	60.0	max follow-up	11	5.5	65.5	0.88
Cardiovascular Health Study (CHS)	0.0222	NA	0.022	82.5	max follow-up	13	6.5	89.0	1.98
Chinese Multi-Provincial Cohort Study (CMCS)	0.012	NA	0.012	49.5	mean follow-up	11	7.3	56.8	0.68
Cohort of Norway (CONOR) (except Troms? and HUNT studies)	0.0204	NA	0.020	58.5	max follow-up	12	6.0	64.5	1.32
Cohort study from Porto Alegre, southern Brazil	0.0032	NA	0.003	53.0	mean follow-up	6	4.0	57.0	0.18

Supplementary Data 4. Age at event calculation for the etiologic effect of BMI on ischemic heart disease * (continued).

Study	Weighting	Weighting rescaled	Weighting fraction	Age at baseline¶ (y)	Type of follow-up time	Follow-up time (y)	Mean or 2/3 follow-up time§ (y)	Age at event‡ (y)	Weighted age at event# (y)
Danish Diet, Cancer and Health study	0.0219	NA	0.022	57.0	max follow-up	11	5.5	62.5	1.37
DETECT	0.0115	NA	0.012	56.5	max follow-up	5	2.5	59.0	0.68
Diabetes Intervention Study	0.0099	NA	0.010	42.0	mean follow-up	11	7.3	49.3	0.49
Dubbo Study of the Elderly	0.0244	NA	0.024	79.0	mean follow-up	16	10.7	89.7	2.19
East and West Finland Cohort, Seven Countries Study, Phase II	0.0055	NA	0.006	79.0	mean follow-up	10	6.7	85.7	0.47
Electrical Generating Authority of Thailand Study (EGAT)	0.0024	NA	0.002	44.5	mean follow-up	11.4	7.6	52.1	0.13
EURODIAB Prospective Complications Study	0.0084	NA	0.008	39.0	mean follow-up	7	4.7	43.7	0.37
Finnish Mobile Clinic Health Examination Survey (FMC)	0.0235	NA	0.024	49.5	max follow-up	28	14.0	63.5	1.49
Fletcher Challenge Heart and Health Study	0.0169	NA	0.017	54.5	mean follow-up	5.9	3.9	58.4	0.99
Framingham Heart Study-Cohort	0.0217	NA	0.022	45.5	max follow-up	59	29.5	75.0	1.63
Framingham Heart Study-Offspring	0.0204	NA	0.020	40.0	max follow-up	36	18.0	58.0	1.18
General Post Office Study (GPO)	0.0119	NA	0.012	52.5	max follow-up	41	20.5	73.0	0.87
Goteborg BEDA study	0.0095	NA	0.010	51.0	max follow-up	19	9.5	60.5	0.57
Health Risks and Quality of Life in the Hong Kong Elderly	0.0061	NA	0.006	85.0	mean follow-up	3	2.0	87.0	0.53
The Hisayama Study	0.0078	NA	0.008	65.5	mean follow-up	25.1	16.7	82.2	0.64

Supplementary Data 4. Age at event calculation for the etiologic effect of BMI on ischemic heart disease [*] (continued).

Study	Weighting	Weighting rescaled	Weighting fraction	Age at baseline[¶] (y)	Type of follow-up time	Follow-up time (y)	Mean or 2/3 follow-up time[§]	Age at event[‡] (y)	Weighted age at event[#] (y)
Honolulu Heart Program (HHP)	0.0217	NA	0.022	52.0	max follow-up	29	14.5	66.5	1.44
Kaunas Rotterdam Intervention Study (KRIS)	0.0203	NA	0.020	47.0	max follow-up	38	19.0	66.0	1.34
The Kinmen Neurological Disorders Survey (KINDS)	0.0007	NA	0.001	70.5	mean follow-up	2.9	1.9	72.4	0.05
Korean Medical Insurance Corporation Study (KMIC)	0.01	NA	0.010	47.0	mean follow-up	4	2.7	49.7	0.50
LIFE Study	0.0183	NA	0.018	67.5	mean follow-up	4	2.7	70.2	1.28
MEGA Study	0.0082	NA	0.008	55.0	mean follow-up	5.3	3.5	58.5	0.48
Melbourne Collaborative Study (MCCS)	0.0177	NA	0.018	51.5	mean follow-up	13.3	8.9	60.4	1.07
Multi-Ethnic Study of Atherosclerosis (MESA)	0.0177	NA	0.018	64.5	max follow-up	8	4.0	68.5	1.21
Multifactor Primary Prevention Study	0.02	NA	0.020	51.0	max follow-up	28	14.0	65.0	1.30
Multiple Risk Factor Intervention Trial (MRFIT)	0.0225	NA	0.023	46.0	max follow-up	12	6.0	52.0	1.17
National Heart foundation Risk Factor Prevalence Study	0.0134	NA	0.013	44.5	mean follow-up	15	10.0	54.5	0.73
Newcastle Study	0.0034	NA	0.003	49.0	mean follow-up	5.7	3.8	52.8	0.18
NHANES I Epidemiologic Follow-up Study (NHEFS)	0.0231	NA	0.023	49.5	max follow-up	21	10.5	60.0	1.39
NIPPON DATA80	0.0069	NA	0.007	61.5	mean follow-up	14	9.3	70.8	0.49
NIPPON DATA90	0.0034	NA	0.0034	65.0	mean follow-up	10	6.7	71.7	0.24
The Nord-Trondelag Health Study 2 (HUNT 2)	0.0218	NA	0.0218	56.5	max follow-up	13	6.5	63.0	1.37

Supplementary Data 4. Age at event calculation for the etiologic effect of BMI on ischemic heart disease [*] (continued).

Study	Weighting	Weighting rescaled	Weighting fraction	Age at baseline[¶] (y)	Type of follow-up time	Follow-up time (y)	Mean or 2/3 follow-up time[§]	Age at event[‡] (y)	Weighted age at event[#] (y)
The Northern Manhattan Study (NOMAS)	0.0171	NA	0.0171	70.0	mean follow-up	10	6.7	76.7	1.31
Norwegian Counties Study (NCS)	0.0236	NA	0.0236	35.0	max follow-up	35	17.5	52.5	1.24
Perth Risk Factors Survey (Perth MONICA)	0.0118	NA	0.0118	55.0	mean follow-up	14.4	9.6	64.6	0.76
Prospective Cardiovascular Munster Study (PROCAM)	0.018	NA	0.0180	42.5	mean follow-up	12	8.0	50.5	0.91
Prospective Study of Women in Gothenburg	0.0143	NA	0.0143	49.0	max follow-up	40	20.0	69.0	0.99
Puerto Rico Heart Health Program (PRHHP)	0.0164	NA	0.0164	54.5	mean follow-up	8.3	5.5	60.0	0.98
Risk of ischaemic heart disease in Zaragoza (ZACARIS)	0.0139	NA	0.0139	62.0	mean follow-up	5	3.3	65.3	0.91
Rotterdam Study (RS)	0.0179	NA	0.0179	77.0	mean follow-up	17	11.3	88.3	1.58
Saitama Cohort Study	0.0005	NA	0.0005	57.0	mean follow-up	11	7.3	64.3	0.03
Seven Cities Cohort Study	0.0021	NA	0.0021	62.5	mean follow-up	2.7	1.8	64.3	0.14
Shibata Cohort Study	0.0045	NA	0.0045	64.5	mean follow-up	20	13.3	77.8	0.35
Singapore Cardiovascular Cohort Study	0.011	NA	0.0110	53.5	mean follow-up	8.5	5.7	59.2	0.65
Study of Multifactorial Prevention of Ischemic Heart Disease	0.0213	NA	0.0213	49.5	max follow-up	33	16.5	66.0	1.41
SENECA	0.0077	NA	0.0077	73.5	mean follow-up	10	6.7	80.2	0.62
Tanno - Soubetsu Study	0.0014	NA	0.0014	52.0	mean follow-up	16.4	10.9	62.9	0.09

Supplementary Data 4. Age at event calculation for the etiologic effect of BMI on ischemic heart disease * (continued).

Study	Weighting	Weighting rescaled	Weighting fraction	Age at baseline¶ (y)	Type of follow-up time	Follow-up time (y)	Mean or 2/3 follow-up time§	Age at event‡ (y)	Weighted age at event# (y)
Tehran Lipid and Glucose Study (TLGS)	0.0173	NA	0.0173	60.0	mean follow-up	7.6	5.1	65.1	1.13
The Troms? Study (4th cohort)	0.023	NA	0.0230	61.0	max follow-up	16	8.0	69.0	1.59
Turkish Adult Risk Factor Study (TARF)	0.0191	NA	0.0191	50.0	max follow-up	12	6.0	56.0	1.07
Uppsala Longitudinal Study of Adult Men (ULSAM)	0.012	NA	0.0120	70.0.	max follow-up	17	8.5	78.5	0.94
Ventimiglia di Sicilia Heart Study	0.0056	NA	0.0056	55.0	mean follow-up	15	10.0	65.0	0.36
Health Monitoring & Promotion Programme (VHM&PP)	0.0232	NA	0.0232	57.0	mean follow-up	14.4	9.6	66.6	1.55
Western Collaborative Group Study (WCGS)	0.0127	NA	0.0127	49.0	mean follow-up	33	22.0	71.0	0.90
Whitehall I Study	0.0223	NA	0.0223	54.5	max follow-up	43	21.5	76.0	1.69
Whitehall II Study	0.0076	NA	0.0076	51.0	max follow-up	19	9.5	60.5	0.46
Women's Health Initiative Study (WHI-OS)	0.0241	NA	0.0241	64.5	max follow-up	12	6.0	70.5	1.70
Women's Health Initiative study, Clinical Trial (WHI-CT)	0.0158	NA	0.0158	64.5	mean follow-up	7	4.7	69.2	1.09
Zutphen cohort, Seven Countries Study, Phase II	0.0032	NA	0.0032	79.0	mean follow-up	10	6.7	85.7	0.27
Total	0.998	NA	0.998						**66.28**

* Ischemic heart disease is also referred as coronary heart disease

¶ Age at age at baseline was calculated as the mean of the range of age at baseline. For example, in the Abdominal Aortic Aneurysm Screening Program, the range age at baseline was 65 to 84, hence the age at baseline was calculated as (65+84)/2 = 74.5

§ The follow-up time was calculated as half of the max follow-up time, or as 2/3 of the mean or median follow-up, depending on the type of follow-up time reported.

‡ Age at event corresponds to the sum of the age at baseline plus the mean or 2/3 follow-up time.

[#] Age at event was then weighted by its corresponding weighting fraction. The individual weighted age at event where were added up to obtain the final age at event for the metanalysis.

BMI, body-mass index; SSBs, sugar-sweetened beverages
