# Supplementary material for: Burdens of type 2 diabetes and cardiovascular disease attributable to sugar-sweetened beverages in 184 countries
Source: Nat Med. 2025 Jan 6;31(2):552–64. doi: 10.1038/s41591-024-03345-4 (PMC11835746; doi:10.1038/s41591-024-03345-4)
Supplement: Supplementary file 7 — Study characteristics and effect estimates from studies assessing the association between education level with T2D and CVD risk for disaggregating GBD disease estimates. [file 41591_2024_3345_MOESM7_ESM.pdf]

Burdens of type 2 diabetes and cardiovascular disease burdens to sugar-sweetened beverages in 184 countries

Supplementary Data 5 | Study characteristics and effect estimates from studies assessing the association between education level with T2D and CVD risk for disaggregating GBD disease estimates.

Supplementary Data 5. Study characteristics and effect estimates from studies assessing the association between education level with diabetes and CVD risk for disaggregating GBD disease estimates.

Study	Study design	Disease outcome[*]	Outcome ascertainment	Education definition	Original effect estimate (RR (95% CI))	Covariates[¶]	Country income[¥]	Sample size	Calculated effect high vs. low education[†] (RR (95% CI))
**Type 2 diabetes**									
Dagenais *et al.*, 2016	Prospective cohort (Prospective Urban Rural Epidemiology study – PURE)	T2D prevalence	Blood glucose or self-report	Primary (0-6 years); secondary (7-11 years); college, trade, or university (>11 years)	Primary vs. college, trade, or university [HIC: 1.28 (1.01, 1.60); UMIC: 1.21 (1.04, 1.41); LMIC: 1.04 (0.93, 1.17); LIC: .098 (0.79, 1.21)]	Age, sex, residency location, BMI, waist-hip ratio, physical activity level, diet quality score (AHEI), smoking, family history of T2D, ethnicity	HIC<br>UMIC<br>LMIC<br>LIC	3 countries; n=14,757<br>7 countries; n=26,088<br>4 countries; n=55,430<br>4 countries; n=23,391	0.78 (0.63, 0.99)<br>0.83, (0.71, 0.96)<br>0.96 (0.85, 1.08)<br>1.02 (0.83, 1.27)
Seiglie *et al.*, 2020	Pooled analysis of 29 nationally representative surveys	T2D prevalence	Blood glucose or medication usage	No formal schooling; less than primary school or primary school completed (less than or up to grade 6 completed); and secondary school (grade 7–12) or above[#]	Primary vs. no education [UMIC: 1.21 (0.96, 1.52); LMIC: 1.29 (1.18, 1.41); LIC: 1.53 (1.29, 1.82)]<br><br>Secondary vs. no education [UMIC: 1.42 (1.12, 1.79); LMIC: 1.65 (1.54, 1.77); LIC: 2.15 (1.77, 2.61)]	Age and sex only	UMIC<br>LMIC<br>LIC	11 countries; n=25,439<br>8 countries; n=510,377<br>10 countries; n=26,629	1.34 (1.10, 1.62)[#]<br>1.54 (1.45, 1.63)[#]<br>1.86 (1.59, 2.17)[#]
Agardh *et al.*, 2011	Meta-analysis	T2D incidence	Varied by study (self-report, blood glucose, or medical records)	Varied by study. Low educational level included: no schooling, not having graduated from primary school, no university/academic degree; high educational level included having graduated from primary school, university/academic degree	Lowest vs. highest education level [HIC: 1.45 (1.28, 1.63)]	The model used the most adjusted estimate reported: 6 studies with crude estimates; 1 study adjusted only for age; 1 study adjusted only for age and sex; and 10 studies adjusted for age, and two or more variables including sex, BMI, ethnicity, waist-to-hip ratio, smoking, physical activity, diet score, and other related variables	HIC	18 studies; 9 countries n=~337,996	0.69 (0.61, 0.78)[£]

Supplementary Data 5. Study characteristics and effect estimates from studies assessing the association between education level with T2D and CVD risk for disaggregating GBD disease estimates (continued).

Study	Study design	Disease outcome*	Outcome ascertainment	Education definition	Original effect estimate (RR (95% CI))	Covariates¶	Country income¥	Sample size	Calculated effect high vs. low education† (RR (95% CI))
**Cardiovascular disease**									
Yusuf *et al.*, 2020	Prospective cohort (Prospective Urban Rural Epidemiology study – PURE)	CVD prevalence	Self-reported history of CVD	Primary (0-6 years); secondary (7-11 years); college, trade, or university (>11 years)	Primary vs. trade, college, or university [HIC: 1.18 (0.89, 1.58); MIC: 1.35 (1.19, 1.53); 2.25 (1.59, 3.19)]	Age, sex, area of residence, tobacco use, alcohol use, diet score, physical activity, hypertension, T2D, non-HDL cholesterol, waist to hip ratio, depression, grip strength, household air pollution	HIC	4 countries; n=17,249	0.85 (0.63, 1.12)
							MIC	12 countries; n=102,680	0.74 (0.65, 0.84)
							LIC	5 countries; n=35,793	0.44 (0.31, 0.63)
Khaing *et al.*, 2017	Meta-analysis	CVD prevalence and mortality	Varied by study (medical records or self-report of medical diagnosis)	Low (<9 years: illiteracy, no education, basic, primary education); medium (10–12 years: secondary, high school, intermediate, technical, apprenticed, trade, vocation); high (>12 years: university, college, associates, master, professional, PhD)	Low vs. high education level [coronary artery disease: 1.36 (1.11, 1.66); cardiovascular events 1.50 (1.17, 1.92); stroke 1.23 (1.06, 1.43)]	Age and sex at the minimum. Some studies were additionally adjusted by some cardiovascular risk factors	HICµ	144 studies; 17 countries (14 HIC; n range=128 to 4,157,202	0.76 (0.68, 0.85)
Jackson *et al.*, 2018	Prospective cohort	Stroke incidence	Hospital records	College or university degree; certificate, diploma, trade, apprenticeship, higher school; school or intermediate certificate; or no qualifications.	No qualifications vs. college or university degree [men: 1.25 (1.07, 1.46); women 1.41 (1.16, 1.71)]	Age, sex	HIC	n=253,657	0.76 (0.68, 0.86)
Hassen *et al.*, 2020	Prospective cohort	CVD prevalence	Self-reported history of CVD	Low (early childhood, primary educ, and ≤ lower	High vs. low [Overall: 0.82 (0.79, 0.90)]	Age, sex, household income, retirement	HIC	n=14,322	0.82 (0.79, 0.90)

Supplementary Data 5. Study characteristics and effect estimates from studies assessing the association between education level with T2D and CVD risk for disaggregating GBD disease estimates (continued).

Study	Study design	Disease outcome*	Outcome ascertainment	Education definition	Original effect estimate (RR (95% CI))	Covariates¶	Country income¥	Sample size	Calculated effect high vs. low education† (RR (95% CI))
				secondary education); medium (upper secondary education and post-secondary non-tertiary education), and high (tertiary education including bachelor's, master's, PhD)		status, marital status, and social support			
Min *et al.*, 2017	Prospective cohort	CVD prevalence	Self-reported history of CVD and blood pressure measurements	Less than high school (<12 years); high school graduate or GED equivalent; some college; and college graduate and above§	Less than high school vs. college [MI: 2.25 (1.57, 3.21); stroke: 2.49 (1.68, 3.70)]<br><br>Some college vs college [MI: 1.56 (1.07, 2.28); stroke 2.49 (1.68, 3.70)]	Age and sex	HIC	n=5,031	0.49 (0.39, 0.62)§
Liao *et al.*, 2021	Prospective cohort	Heart failure incidence	Medical records	Basic education (less than high school completion); intermediate education (high school degree or vocational school); and advanced education (attending or completed college of professional school)	Advanced vs. basic [Overall: 0.50 (0.45, 0.55)]	Age, gender, and race	HIC	n=12,315	0.50 (0.45, 0.55)
Reshetnyak *et al.*, 2020	Prospective cohort	Stroke incidence	Medical records	<high school and high school graduate or more	Low vs high education [Overall: 1.32 (1.14, 1.52)]	Age and gender	HIC	n=27,813	0.76 (0.66, 0.88)

Supplementary Data 5. Study characteristics and effect estimates from studies assessing the association between education level with T2D and CVD risk for disaggregating GBD disease estimates (continued).

Study	Study design	Disease outcome*	Outcome ascertainment	Education definition	Original effect estimate (RR (95% CI))	Covariates¶	Country income¥	Sample size	Calculated effect high vs. low education† (RR (95% CI))
Li *et al.*, 2022	Prospective cohort (Prospective Urban Rural Epidemiology study – PURE)	CVD incidence	Self-reported history of CVD	Primary (0-6 years); secondary (7-11 years); college, trade, or university (>11 years)	Primary vs. Trade, college, or university [CVD: 1.63 (1.40, 1.89); MI: 1.32 (0.99, 1.77); stroke: 1.73 (1.45, 2.06)]	Age, sex, are of residence, tobacco use, alcohol use, diet score, physical activity, hypertension, T2D, non-HDL cholesterol, waist to hip ratio, depression, grip strength, household air pollution, and a random intercept of centres	UMIC	1 country; n=47,262	0.62 (0.56, 0.69)
Joseph *et al.*, 2022	Prospective cohort (Prospective Urban Rural Epidemiology study – PURE)	CVD incidence	Self-reported history of CVD	Primary (0-6 years); secondary (7-11 years); college, trade, or university (>11 years)	Primary vs. Trade, college, or university [CVD: 2.00 (1.66, 2.41)]	Age, sex, are of residence, tobacco use, alcohol use, diet score, physical activity, hypertension, T2D, non-HDL cholesterol, waist to hip ratio, depression, grip strength, household air pollution	LMIC	3 countries; n=33,583	0.50 (0.41, 0.60)
Lopez-Jaramillo *et al.*, 2022	Prospective cohort (Prospective Urban Rural Epidemiology study – PURE)	CVD incidence	Self-reported history of CVD	Primary (0-6 years); secondary (7-11 years); college, trade, or university (>11 years)	Primary vs. Trade, college, or university [CVD: 1.05 (0.82, 1.33); MI: 0.99 (0.70, 1.41)]	Age, sex, are of residence, tobacco use, alcohol use, diet score, physical activity, hypertension, T2D, non-HDL cholesterol, waist to hip ratio, depression, grip strength, household air pollution	UMIC µ	4 countries; n=24,718	0.97 (0.80, 1.18)

*Relative risk difference estimates based on T2D and CVD prevalence or incidence are assumed to be generalizable to T2D and CVD incidence, mortality, and DALYs, the outcomes of interest in this comparative risk assessment analysis.

† Our desired exposure was the effect estimate for "high vs. low" education. Estimates for "high" and "low" education level from each study were matched as closely as possible with the GDD education level definitions as: "low" 0 to 6 years of education; and "high" >12 years of education. When estimates were given for "low vs. high" education instead of "high vs. low" education, estimates and 95% CI were inverted as 1/RR to obtain the effect for "high vs. low". In addition, estimates within studies were pooled together when: 1) multiple estimates for different CVD outcomes were reported, 2) males and females estimates were reported separately, 3) estimates for different locations were reported separately (except when reported by country income); or 4) an intermediate education level fell into the low or high GDD education level category. Some studies required a more complex methodology for pooling estimates together as described in detail for each of these studies.

For Seiglie *et al.*, the categories "no formal education" and "primary education" fell into the GDD's definition of low education, with "no formal education" being the reference category. To combine these categories, we first calculated the ratio of "secondary education vs. no formal education" to "primary education vs. no formal education" - for each country income level category separately - to obtain the effect of "secondary education vs. primary education" using the equation $ES_1/ES_2 = exp(ln(ES_1) - ln(ES_2))$, where ES_1 is the effect estimate for "secondary vs. no formal education" and ES_2

is the effect estimate for “primary vs. no formal education”. The 95% CI was calculated with the equations: (1) $Var(ES_1/ES_2) = ES_1/ES_2 \sqrt{Var(ES_1)^2/ES_1^2 + Var(ES_2)^2/ES_2^2}$, and (2) 95%CI = ($ES_1/ES$) ± $Var(ES_1/ES_2)$*1.959. Next, we pooled the calculated effect of “secondary education vs. primary education” with the effect of “secondary education vs. primary education” as given in the study to obtain the final estimate for high vs. low education for each country income level category separately.

£ We excluded the LIC estimate from Aghard *et al*., 2011 given that only one study was used to inform this estimate and we did not consider it representative for the whole category. We also excluded the MIC estimate from this study because we identified other studies that provided separate estimates for UMIC and LMIC, and we wanted to keep the most specific possible estimates for each income category.

µ Some studies pooled estimates from countries with a different country income classification. We discussed on a case-by-case basis whether or not to keep the estimate from that study, and if keeping under which country income category locate it. Spefically, in Khaing *et al.,* 2017, 93.1% of the studies were from HIC (17 HIC, 1 UMIC, and 2 LMIC), thus, we decided to classify the pooled estimate from this study as for HIC. Lopez-Jaramillo *et al*., 2021 included 3 UMIC and 1 HIC, thus we decided to categorize it as HIC.

§ For Min *et al.,* the categories “some college” and “college graduate and above” fell into the GDD’s definition of high education, with “college graduate and above” being the reference category. To combine these categories, we first pooled together the effect estimates for MI and stroke for each education category separately. Next, we calculated the ratio of “some college vs. college graduate and above” to “less than high school (<12 years) vs. college graduate and above” to obtain the effect of “some college vs. less than high school (<12 years)” using the equation $ES_1/ES_2 = exp(ln(ES_1) - ln(ES_2))$, where ES_1 is the effect estimate for “some college vs. college graduate and above” and ES_2 is the effect estimate for “less than high school (<12 years) vs. college graduate and above”. The 95% CI was calculated with the equations: (1) $Var(ES_1/ES_2) = ES_1/ES_2 \sqrt{Var(ES_1)^2/ES_1^2 + Var(ES_2)^2/ES_2^2}$, and (2) 95%CI = ($ES_1/ES$) ± $Var(ES_1/ES_2)$*1.959. Next, we inverted the effect estimate of “less than high school (<12 years) vs. college graduate and above” as 1/RR to obtain the effect for “college graduate and above vs. less than high school (<12 years)”. Finally, we pooled the calculated effects for “some college vs. less than high school (<12 years)” and “college graduate and above vs. less than high school (<12 years)” to obtain the final estimate of high vs. low education.

¥ Based on World Bank Country Income group classification.

¶ The ideal effect estimates for this analysis were those adjusted only for age and sex adjusted to avoid the attenuating effects of adjusting for additional covariates. The aim of collating these studies was to partition Global Burden of Disease study age-sex stratified CVD and T2D disease estimates further into education level and urban/rural residence finer stratifications, not to determine the causal association between education level and urban rural residence with T2D or CVD risk.

AHEI, alternative healthy eating index; BMI, body mass index; CI, confidence interval; CVD, cardiovascular disease; GDD, Global Dietary Database; HIC, high-income country; LIC, low-income country; LMIC, low middle-income country; MI, myocardial infraction; RR, relative risk; T2D, type 2 diabetes; UMIC, upper middle-income country
