# Supplementary material for: Burdens of type 2 diabetes and cardiovascular disease attributable to sugar-sweetened beverages in 184 countries
Source: Nat Med. 2025 Jan 6;31(2):552–64. doi: 10.1038/s41591-024-03345-4 (PMC11835746; doi:10.1038/s41591-024-03345-4)
Supplement: Supplementary file 8 — Study characteristics and effect estimates from studies assessing the association between area of residence with T2D and CVD risk for disaggregating GBD disease estimates. [file 41591_2024_3345_MOESM8_ESM.pdf]

Burdens of type 2 diabetes and cardiovascular disease burdens to sugar-sweetened beverages in 184 countries

Supplementary Data 6 | Study characteristics and effect estimates from studies assessing the association between area of residence with T2D and CVD risk for disaggregating GBD disease estimates.

Supplementary Data 6. Study characteristics and effect estimates from studies assessing the association between area of residence with diabetes and CVD risk for disaggregating GBD disease estimates.

Study	Study design	Disease outcome*	Outcome ascertainment	Area of residence definition†	Original effect estimate (RR (95% CI))	Covariates¶	Country income#	Sample size	Calculated effect urban vs. rural (RR (95% CI))†
**T2D**									
Dagenais *et al.*, 2016	Prospective cohort (Prospective Urban Rural Epidemiology study – PURE)	T2D prevalence	Blood glucose or self-report	Urban and rural area of residence (based on the national for urban and rural, or defined as rural if the community is isolated from urban centers (distance of >50 km or lack easy access to commuter transportation)	Urban vs. rural [HIC: 1.07 (0.81, 1.40); LMIC: 1.20 (1.02, 1.41); LIC: 1.65 (1.28, 2.12)]¥	Age, sex, residency location, BMI, waist-hip ratio, physical activity level, diet quality score (AHEI), smoking, family history of T2D, ethnicity	HIC<br>LMIC<br>LIC	3 countries; n=14,757<br>4 countries; n=55,430<br>4 countries; n=23,391	1.07 (0.81, 1.40)<br>1.20 (1.02, 1.41)<br>1.65 (1.28, 2.12)
den Braver *et al.*, 2018	Meta-analysis	T2D prevalence or incidence	Blood glucose or self-report	Urban and rural (varied by study)	Urban vs. rural [UMIC: 1.49 (1.16–1.92); LMIC: 1.45 (1.20–1.74)	All age-sex adjusted ; some studies also adjusted for BMI, phys. Activity, etc.	UMIC<br>LMIC	8 studies, 15 countries: n=~271,629<br>7 studies, 6 countries: n=~40,905	1.49 (1.16, 1.92)<br>1.45 (1.20, 1.74)
O'Connor *et al.*, 2012	Cross-sectional	T2D prevalence	Self-report	Rural and urban (based on the CDC metropolitan statistical codes to denote urban and rural populations)	Rural vs. urban [Overall: 0.94 (0.89, 0.99)]	Household income, education, age, sex, BMI, race/ethnicity	HIC	n=214,698	1.06 (1.01, 1.12)
Corsi *et al.*, 2019	Cross-sectional	T2D prevalence	Blood glucose or medication usage	Urban and rural (based on the census definitions including population size, and population density)	Urban vs. rural [Overall: 1.12 (1.01, 1.25)]	Age, sex, smoking and alcohol use, wealth, education, and social caste	LMIC	n=718,597	1.12 (1.01, 1.25)
**Cardiovascular disease**									
Hassen *et al.*, 2020	Prospective cohort	CVD prevalence	Self-reported history of CVD	Large town or city; small town; and rural or village	Urban vs. rural [Small city vs rural: 1.08 (0.99, 1.14); Large city/town vs. rural: 1.14 [1.07-1.18]	Age, sex, household income, retirement status, marital status, and social support	HIC	n=14,322	1.12 (1.08, 1.17)

Supplementary Data 6. Study characteristics and effect estimates from studies assessing the association between area of residence with T2D and CVD risk for disaggregating GBD disease estimates (continued).

Study	Study design	Disease outcome*	Outcome ascertainment	Area of residence definition†	Original effect estimate (RR (95% CI))	Covariates¶	Country income#	Sample size	Calculated effect urban vs. rural (RR (95% CI))†
Reshetnyak *et al*., 2020	Prospective cohort	Stroke incidence	Medical records	Rural and urban (based on a modification of the USDA's Rural Urban Commuting Area (RUCA) system)	Rural vs. urban [Overall: 1.02 (0.74, 1.42)]	Age and gender	HIC	n=27,813	0.98 (0.70, 1.35)
Kapral *et al*., 2019	Retrospective cohort	Stroke incidence	Medical records	Rural and urban (rural was defined as community outside of the commuting zone of census metropolitan and census agglomeration areas with a population size ≤10,000)	Rural vs. urban [Stroke: 1.11 (1.08, 1.13)]	Age and sex	HIC	n=6,207,032	0.90 (0.88, 0.93)
Bernabe-Ortiz *et al*., 2022	Pooled analysis of national cross-sectional surveys	CVD risk	Self-report risk factors including sex, age, smoking, and BMI	Rural and urban (rural setting is the area with up to 100 households contiguously grouped, not a district capital, or an area having ≥100 households, dispersed or scattered without forming blocks; the remainder settings were considered urban areas)	Rural vs. urban [Overall: 0.82 (0.75, 0.89)]	Adjusted by age, sex, education, wealth index, and survey year	UMIC	n=80,409	1.22 (1.12, 1.33)
Lu *et al*., 2019	Cross-sectional	CVD risk	Self-report and baseline measurements of risk factors	Urban and rural	Rural vs. urban [Overall: 0.95 (0.78, 1.17)]	Age, sex, geographic region, ethnic origin, occupation, annual household income, and education	UMIC	n=1,680,126	1.05 (0.85, 1.28)
O'Connor *et al*., 2012	Cross-sectional	CHD prevalence	Self-reported medical diagnosis of	Urban and rural (urban was defined as living in an area with 50,000 or	Rural vs. urban [Overall: 1.09 (1.02, 1.17)]	Annual household income, age, education, gender,	HIC	n=214,000	0.92 (0.85, 0.98)

Supplementary Data 6. Study characteristics and effect estimates from studies assessing the association between area of residence with T2D and CVD risk for disaggregating GBD disease estimates (continued).

Study	Study design	Disease outcome*	Outcome ascertainment	Area of residence definition†	Original effect estimate (RR (95% CI))	Covariates¶	Country income#	Sample size	Calculated effect urban vs. rural (RR (95% CI))†
			coronary heart disease	more inhabitants; whereas rural was defined as living outside of a metropolitan statistical area)		BMI, ethnicity, and smoking status			

¥ We excluded UMIC estimates from Dagenais *et al.,* 2016 as these were already included in den Braver *et al.,* 2018.

*Relative risk difference estimates based on T2D and CVD prevalence, incidence, or risk are assumed to be generalizable to T2D and CVD incidence, mortality, and DALYs, the outcomes of interest in this comparative risk assessment analysis.

† Our desired exposure was the effect estimate for "urban vs. rural" area of residence. When estimates were given for "rural vs. urban" instead of "urban vs. rural", estimates and 95% CI were inverted as 1/RR to obtain the effect for "urban vs. rural". For Hassen *et al*., 2020 the categories "Small city" and "Large city" were both considered as "urban", thus these estimates were pooled together.

¶ The ideal effect estimates for this analysis were those adjusted only for age and sex adjusted to avoid the attenuating effects of adjusting for additional covariates. The aim of collating these studies was to partition Global Burden of Disease study age-sex stratified CVD and T2D disease estimates further into education level and urban/rural residence finer stratifications, not to determine the causal association between education level and urban rural residence with T2D or CVD risk.

AHEI, alternative healthy eating index; BMI, body mass index; CI, confidence interval; CVD, cardiovascular disease; CHD, coronary heart disease; GDD, Global Dietary Database; HIC, high-income country; LIC, low-income country; LMIC, low middle-income country; MI, myocardial infraction; RR, relative risk; T2D, type 2 diabetes; UMIC, upper middle-income country; USDA, United States Department of Agriculture
